# A Novel Catalytic Route to Polymerizable Bicyclic Cyclic Carbonate Monomers from Carbon Dioxide

**DOI:** 10.1002/anie.202205053

**Published:** 2022-05-09

**Authors:** Chang Qiao, Wangyu Shi, Arianna Brandolese, Jordi Benet‐Buchholz, Eduardo C. Escudero‐Adán, Arjan W. Kleij

**Affiliations:** ^1^ Institute of Chemical Research of Catalonia (ICIQ) the Barcelona Institute of Science and Technology Av. Països Catalans 16 43007 Tarragona Spain; ^2^ Universitat Rovira i Virgili C/Marcel ⋅ lí Domingo s/n 43007 Tarragona Spain; ^3^ Catalan Institute of Research and Advanced Studies (ICREA) Pg. Lluís Companys 23 08010 Barcelona Spain

**Keywords:** Carbon Dioxide, Cyclic Carbonates, Homogeneous Catalysis, Monomers, Ring-Opening Polymerization

## Abstract

A new catalytic route has been developed for the coupling of epoxides and CO_2_ affording polymerizable six‐membered bicyclic carbonates. Cyclic epoxides equipped with a β‐positioned OH group can be transformed into structurally diverse bicyclic cyclic carbonates in good yields and with high selectivity. Key to the chemo‐selectivity is the difference between the reactivity of *syn*‐ and *anti*‐configured epoxy alcohols, with the latter leading to six‐membered ring carbonate formation in the presence of a binary Al^III^ aminotriphenolate complex/DIPEA catalyst. X‐ray analyses show that the conversion of the *syn*‐configured substrate evolves via a standard double inversion pathway providing a five‐membered carbonate product, whereas the *anti*‐isomer allows for activation of the oxirane unit of the substrate opposite to the pendent alcohol. The potential use of these bicyclic products is shown in ring‐opening polymerization offering access to rigid polycarbonates with improved thermal resistance.

## Graphical abstract

A novel catalytic approach for the preparation of polymerizable bicyclic carbonates allows the synthesis of a wide range of unique heterocycles. The stereochemical configuration of the epoxy alcohol substrate plays a crucial role, determining the chemoselectivity profile of this coupling manifold. The synthetic potential of these bicyclic six‐membered carbonates was examined, and their successful ring‐opening polymerization under mild conditions gives new incentives for relatively rigid polycarbonate architectures.

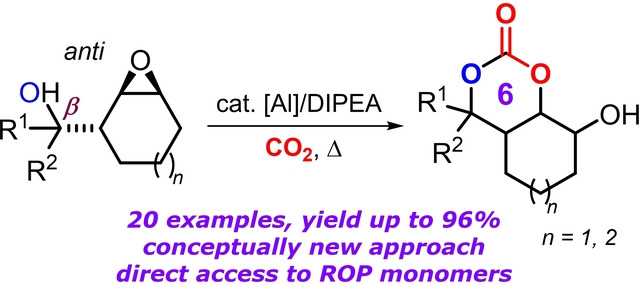

The catalytic recycling of carbon dioxide into valuable chemicals useful as intermediates in synthetic chemistry^[1]^ and polymer science^[2]^ represents a seminal approach within the context of a circular economy.^[3]^ The synthesis of cyclic carbonates through non‐reductive coupling methods represents a valuable carbon dioxide reutilization approach and has advanced greatly in the last decade. In this regard, modern methods build on the [3+2] cycloaddition between readily available cyclic ethers and carbon dioxide (CO_2_) under attractive process conditions.^[4]^ Unlike for this well‐established catalytic formation of 5‐membered cyclic carbonates, traditional methods that allow for larger ring carbonate formation rely on the use of CO or COCl_2_, which are extremely toxic.^[5]^ (Semi)stoichiometric methods (Scheme 1a) include the use of homoallylic alcohols reported by Johnston^[6]^ or diols as established by Buchard,[^7a^, ^7b^] Dyson^[7c]^ and Tomishige.^[7d]^ However, these entries to larger‐ring cyclic carbonates typically require the presence of sacrificial reagents such as alkyl halides, tosyl chloride or cyano pyridines. Transesterification of polyols with activated forms of CO_2_ ^[8]^ and the direct coupling of oxetanes and CO_2_ (Scheme 1a)^[9]^ also have shown potential to access larger carbonate heterocycles.

**Scheme 1:**
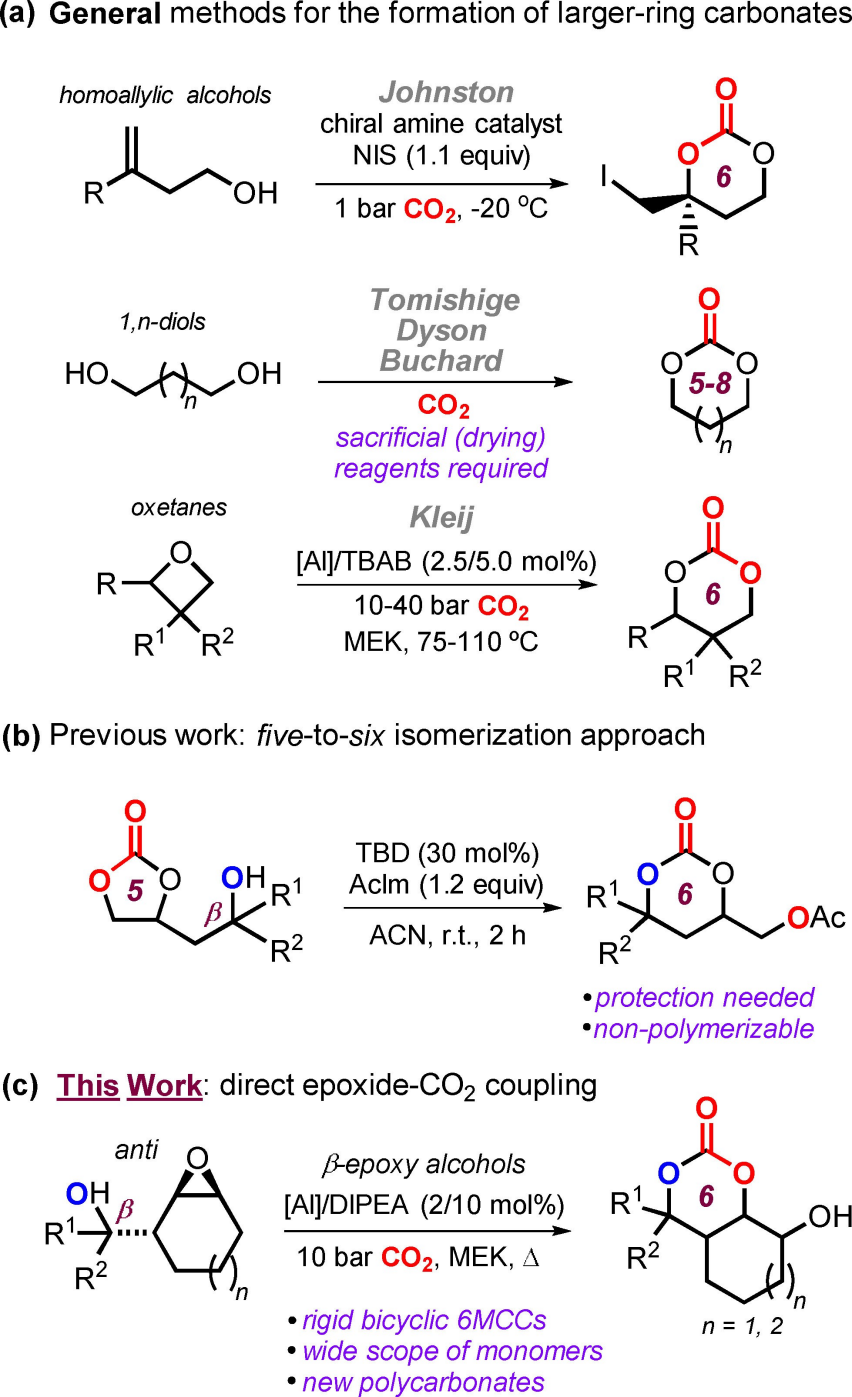
a) General approaches for six‐membered cyclic carbonate synthesis. b) Our previously reported synthesis of *O*‐protected six‐membered carbonates. c) A new and challenging direct coupling of an epoxide and CO_2_ providing bicyclic carbonate heterocycles (This Work).

Larger ring‐carbonates have important incentives in the area of polymer chemistry for the design of new types of functional macromolecules with tuneable mechanical and thermal properties.^[10]^ Therefore, conceptually new catalytic methods that enable a wider scope of such monomers while being created from a renewable carbon source can create important incentives for future low‐carbon emission polymers.

Recently we disclosed an unusual isomerization of five‐ to six‐membered cyclic carbonates (Scheme 1b).^[11a]^ A tertiary, β‐positioned alcohol group in the smaller‐sized heterocycles plays a crucial role as it acts as a pro‐nucleophile able to attack the carbonate C‐center thereby forming a larger‐ring cyclic carbonate. Key to the success of this ring‐expanding approach is the higher kinetic feasibility to intercept the primary alcohol present in the six‐membered compound. The protected six‐membered carbonates were examined under standard ring‐opening polymerization (ROP) conditions but failed to deliver a polycarbonate product as *O*‐deprotection and back‐isomerization to the thermodynamically more stable five‐membered carbonate occurs. This lack of polymerization potential motivated us to design a different strategy that could build on our previously established substrate‐directed CO_2_ activation manifold.[^11b^, ^11c^, ^11d^]

By rigidifying the substrate scaffold though preserving the presence of a β‐positioned alcohol, we discovered a salient difference between *syn*‐ and *anti*‐configured β‐epoxy alcohols in their coupling with CO_2_ leading to cyclic carbonates (Scheme 1c). The *anti* substrates deliver in one step six‐membered bicyclic carbonates in good yield and selectivity under binary catalysis. The mechanistic pathway towards the observed chemo‐selectivity is discussed and supported through X‐ray structural studies, and diversification studies show that these heterocycles have both utility and stability upon modification. ROP of representative bicyclic six‐membered carbonates is successfully demonstrated, illustrating the importance of backbone rigidity to substantially increase the thermal resistance of the resultant polycarbonate.

At the onset of our screening studies, we examined various conditions for the conversion of both *syn*‐ and *anti*‐**1 a** (Table 1).^[12]^ Based on our previous experience,^[11]^ various combinations of Al‐complexes **A** and **B** and additives (DBU, DIPEA and TBAB) were scrutinized to examine their effect on the chemo‐selectivity of this benchmark conversion.^[13]^ First, a low‐temperature approach was chosen (entry 1) with **A** and DIPEA as binary catalyst at relatively high CO_2_ pressure but this proved to be unproductive By increasing the reaction temperature and lowering the pressure to 10 bar, low conversion of **1 a** was noted but no carbonate products were detected (entry 2). We found that a reaction temperature of 100 °C was key towards carbonate formation (see Table S1 and below). In the presence of TBAB (entries 3 and 4), the five‐membered ring carbonate *syn*‐**P1^a^** was formed suggesting the occurrence of a standard double inversion pathway.^[14]^ Interestingly, in the presence of base catalyst (entry 5), a configurationally different five‐membered cyclic carbonate (*anti*‐**P1^b^**) was produced as the major reaction component as supported by X‐ray crystallography (see the Supporting Information).[^15^, ^16^] The presence of both TBAB and DBU (entry 6) leads to a mixture of five‐membered cyclic carbonates *syn*‐**P1^a^** and *anti*‐**P1^b^**.

**Table 1:** Trials conducted with epoxy alcohol substrate **1 a** using various catalysts under different conditions.^[a]^

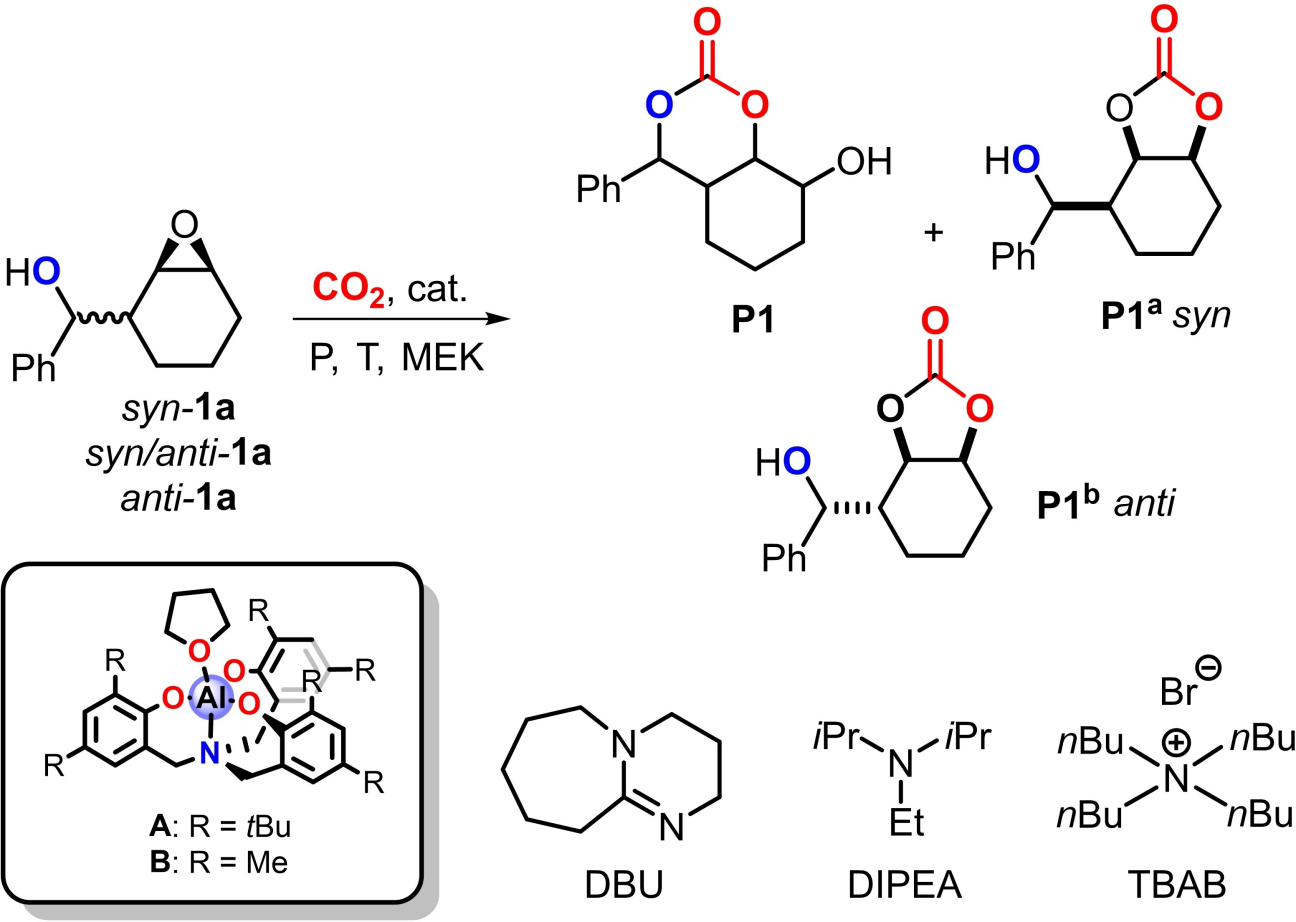							
Entry	**1 a**	Cat. [mol %]	*P*/*T* [bar/°C]	Conv [%]	P1 [%]	P1^[a]^ [%]	P1^[b]^ [%]
1	*syn*	**A**/DIPEA, 10	30/50	<1	–	–	–
2	*syn*	**A**/DIPEA, 10	10/100	18	0	0	0
3	*syn*	**A**/TBAB, 5	10/100	84	0	35	0
4	*syn*	TBAB, 5	10/100	74	0	**37**	0
5	*syn*	DBU, 10	10/100	94	0	0	26
6^[b]^	*syn*	TBAB/DBU	10/100	95	0	12	29
7^[d]^	^[c]^	**A**/DIPEA, 10	10/100	61	20	0	0
8^[d]^	^[c]^	**A**/TBAB, 5	10/100	>99	20	18	0
9^[d]^	*anti*	**A**/TBAB, 5	10/100	>99	83	0	0
10^[d]^	*anti*	**A**/DIPEA, 10	10/100	95	**85**	0	0
11	*anti*	**A**	10/100	36	11	0	0
12	*anti*	DIPEA, 10	10/100	14	0	0	0
13^[d]^	*anti*	**B**/DIPEA, 10	10/100	>99	77	0	0

[a] Reaction performed under the indicated pressure and temperature, MEK as solvent (0.4 mL), *syn*‐**1 a** or **1 a** (0.5 mmol) or *anti*‐**1 a** (0.2 mmol), Al‐complex **A** or **B** (2 mol %), additive (indicated), 22 h. The amount of **P1**, **P1^a^** and **P1^b^** and the overall conversion of **1 a** was determined by ^1^H NMR (CDCl_3_). [b] TBAB (5 mol %) and DBU (10 mol %). [c] A 3 : 1 mixture of s*yn*/*anti‐* **1 a** was used. [d] Yields of the isolated product are reported for these entries.

An important lead result was accomplished in the conversion of a 3 : 1 *syn*/*anti* substrate mixture (entries 7 and 8) leading to substantial formation (20 %, close to the amount of the *anti*‐isomer in *syn*/*anti*‐**1 a**) of the target six‐membered cyclic carbonate **P1** (see the Supporting Information). The selectivity towards **P1** could be further increased by using *anti*‐**1 a** (entries 9–13). Compared to the presence of TBAB, the use of DIPEA shows slightly higher selectivity for **P1** (entries 9 and 10, see Tables S1 and S2 for further details) but, more importantly does not necessitate the use of halide‐containing additives.^[17]^ In the presence of Al‐complex **A** only the reaction had low efficiency (entry 11; 11 % yield of **P1**) while DIPEA individually did not show any selectivity towards the formation of **P1** (entry 12). It therefore appears that a cooperative action of both catalyst components is required for efficient and selective substrate conversion. Though Al‐complex **B** also showed good potential towards the formation of the desired product (entry 13), a somewhat lower yield of **P1** was noted. In the screening studies (Table 1 and S1), we found that other products may also be formed, the details of which can be found in the Supporting Information.

The scope of this new CO_2_ transformative process (Scheme 2) was then examined using the conditions reported in entry 10 of Table 1. Aryl‐substituted bicyclic carbonates **P2**–**P7** could be prepared in good yields from their β‐epoxy alcohols precursors (**1 b**–**1 g**; having secondary alcohol groups) providing, in some cases, useful functional groups for post‐synthetic modifications. Then we also examined precursors comprising primary alcohol groups and this allowed us to prepare **P8** (91 %) and **P9** (57 %) in excellent and moderately high yield, respectively. The lower yield for **P9** is ascribed to the more flexible nature of the cycloheptyl ring and a higher energy requirement to produce a reactive conformation.

**Scheme 2:**
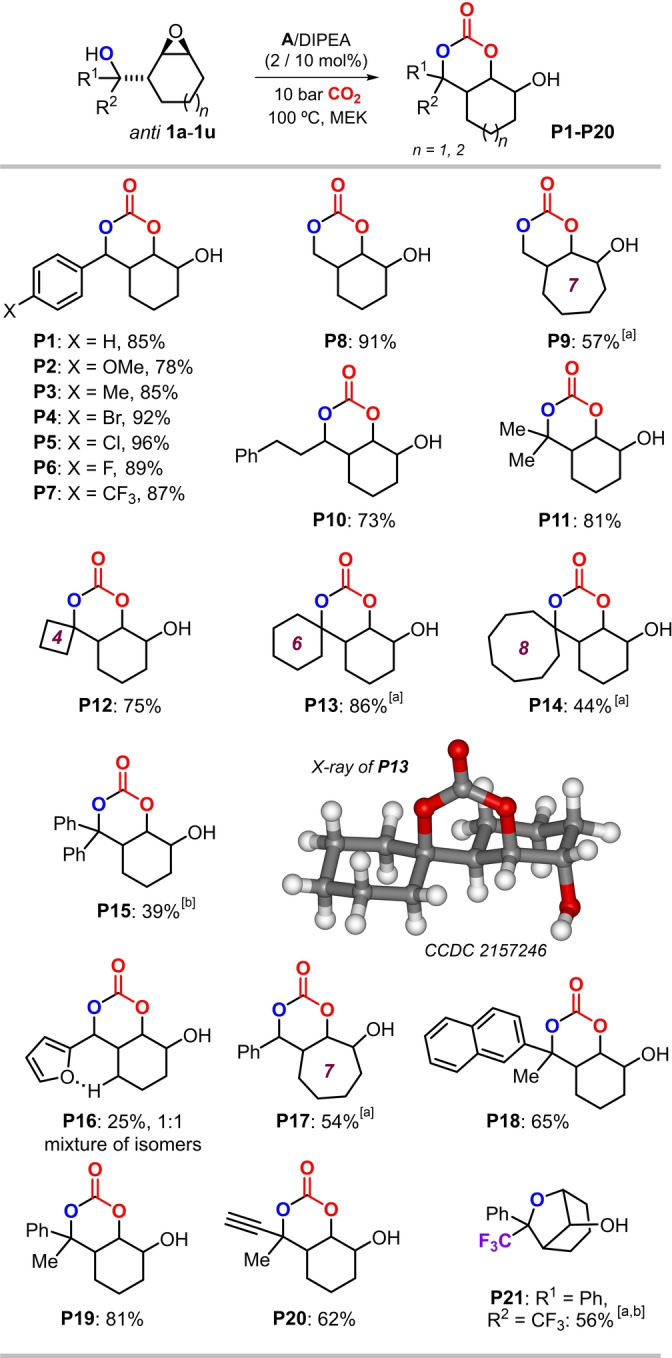
Scope of six‐membered bicyclic carbonates (**P1**–**P20**) by coupling of epoxy alcohols **1 a**–**1 u** and CO_2_ in the presence of Al‐complex **A** and DIPEA. [a] Reaction time was 72 h. [b] Using TBAB (5 mol %) instead of DIPEA.

In order to widen the scope, epoxy alcohol substrates with groups other than aryls were also tested, providing access to bicyclic carbonates **P10**–**P14** in good yields (except for **P14**: 44 %). In some of these cases, a longer reaction time was needed to reach higher substrate conversion such as for spiranes **P13** and **P14**. In the latter case the twisted nature of the cyclooctyl ring likely increases the steric impediment around the alcohol group, leading to slower intramolecular attack on the oxirane unit. A similar “steric” effect probably holds for the synthesis of **P15** (39 %), whereas the low yield of the furan‐derivative **P16** (25 %) is ascribed to (thermal) decomposition over time which likely involves the reactive furan group.^[18]^

**P16** was isolated as a mixture of rotamers as suggested by molecular modelling studies. The isomers relate to the relative positioning of the furan group to the bicyclic scaffold with CH⋅⋅⋅O interactions being competitive to HO⋅⋅⋅OC(O)O hydrogen bonding. Finally, we examined the use of an aryl‐substituted cycloheptane oxide and “mixed” substituted epoxy alcohols which allowed to prepare the carbonate products **P17**–**P20** in appreciable yields. Notably, **P20** (62 % yield) featuring a terminal alkyne offers a synthetic handle while building up molecular complexity. Substrate **1 u** having a strongly electron‐withdrawing CF_3_ group changed the chemo‐selectivity drastically. Only a trace amount of the desired product could be detected in the crude by ^1^H NMR. From the reaction mixture we were able to isolate and characterize bicycle **P21** (see the Supporting Information for details).^[19]^

The synthetic potential and stability of bicyclic carbonates **P8** and **P20** was then examined (Scheme 3). Scaling up the synthesis of **P8** (79 %) was straightforward providing gram‐quantity of this bicyclic carbonate (Scheme 3a). Dess–Martin oxidation of **P8** (Scheme 3b) gave access to the ketone product **P22** in 75 % yield as a mixture of isomers as the carbonyl fragment can have two relative orientations (*exo* and *endo*) with respect to the cyclic carbonate ring. *O*‐protection in **P8** was simple and straightforward (Scheme 3c, d) with both silylated **P23** (91 %) and phenylester **P24** (75 %) isolated in good yields. Acrylic ester derivative **P25** (76 %, Scheme 3e) was produced by coupling of **P8** with a propargylic ester, and a Cu‐catalyzed azide‐alkyne “click” coupling of **P20** resulted cleanly into the formation of 1,2,3‐triazole derivative **P26** (Scheme 3f, 85 %).

**Scheme 3:**
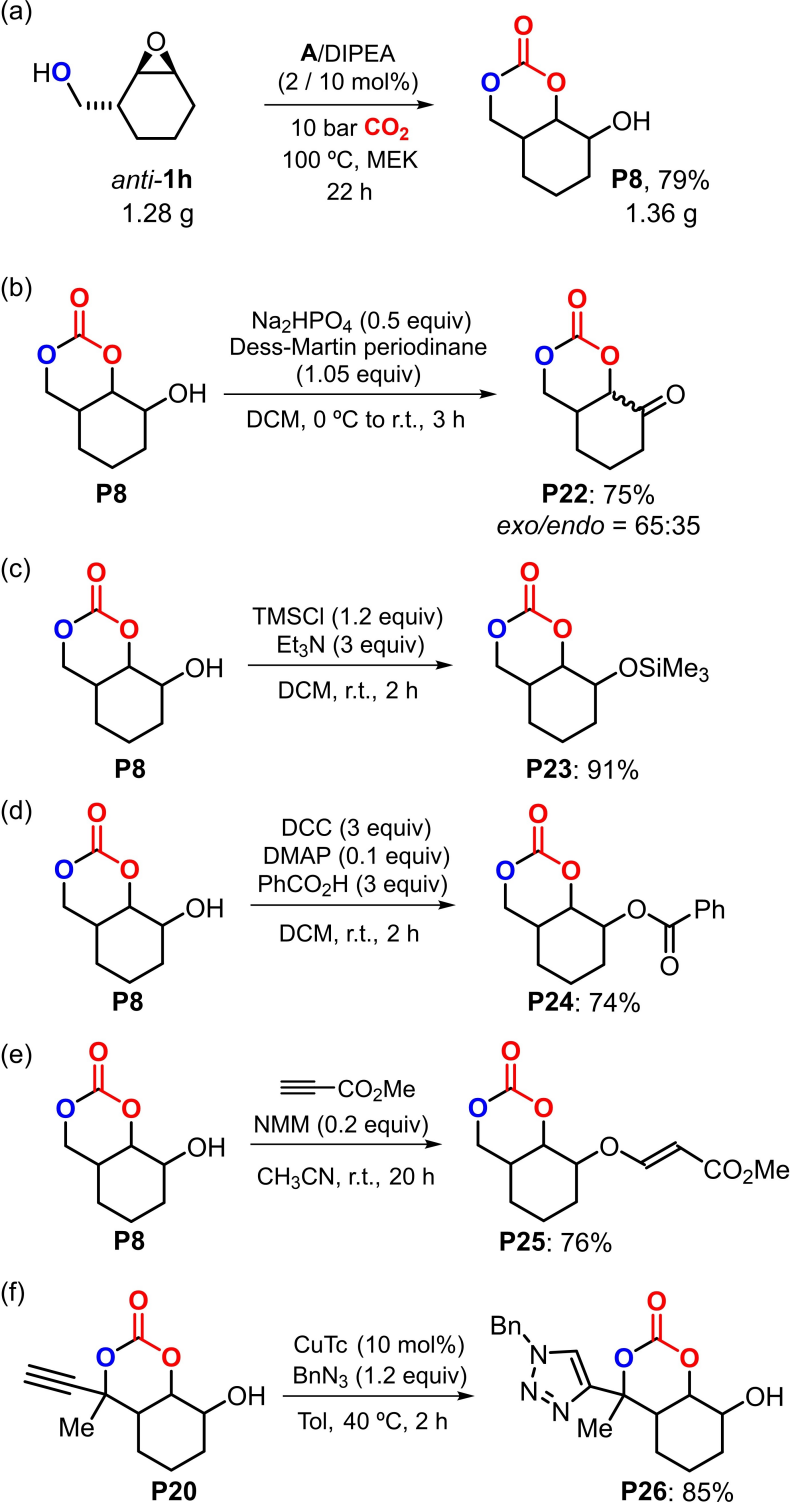
Scale up of **P8** and product diversification studies using both **P8** and **P20**.

Finally, we used monomers **P8** and **P23** to examine their ROP potential under standard conditions (Table 2).[^10a^, ^10e^, ^20^] Monomer **P8** could be oligomerized (entry 1, *M*_n_=1.7 kg mol^−1^, *Ð*=1.47) at incomplete conversion, and extension of the reaction time to 48 h (entry 2) led to (partial) degradation of this oligocarbonate. These data indicated that the free alcohol present in **P8** might interfere with the ROP process. We therefore then examined silyl‐protected **P23** (entry 3) and found that nearly full monomer conversion was achieved at r.t. after 20 h, with the polycarbonate having improved features (*M*_n_=5.9 kg mol^−1^, *Ð*=1.34). Scale up of this process (entry 4) further improved the efficiency (*M*_n_=7.8 kg mol^−1^, *Ð*=1.32) and the new polycarbonate could be isolated as a white solid in 80 %. Performing the ROP of **P23** at higher temperature (entry 5 versus 3) did not provoke any significant change in the polymer properties, which is in line with the non‐innocent nature of the free alcohol in **P8** during the polymerization process. A slightly higher molecular weight polymer was produced in DCM (entry 6 versus 3), while lowering the catalyst loading (entry 7) gave the polycarbonate with slightly improved molecular weight. The isolated polycarbonate from entry 4 was subjected to thermogravimetric analysis (TGA) and differential scanning calorimetry (DSC). The *T*_g_ of this new polycarbonate is substantially higher (at 52 °C) than the unsubstituted polycarbonate that is generated from the ROP of trimethylenecarbonate (*T*_g_=−26 °C for a sample having a molecular weight of around 7 kg mol^−1^).^[21]^ This more rigid polycarbonate also exhibits a high *T*_d_^5^ of 234 °C favourable to process the polymer beyond its glass transition.

**Table 2:** ROP studies using **P8** and **P23** as monomers, and TBD/BnOH as catalyst/initiator.^[a]^

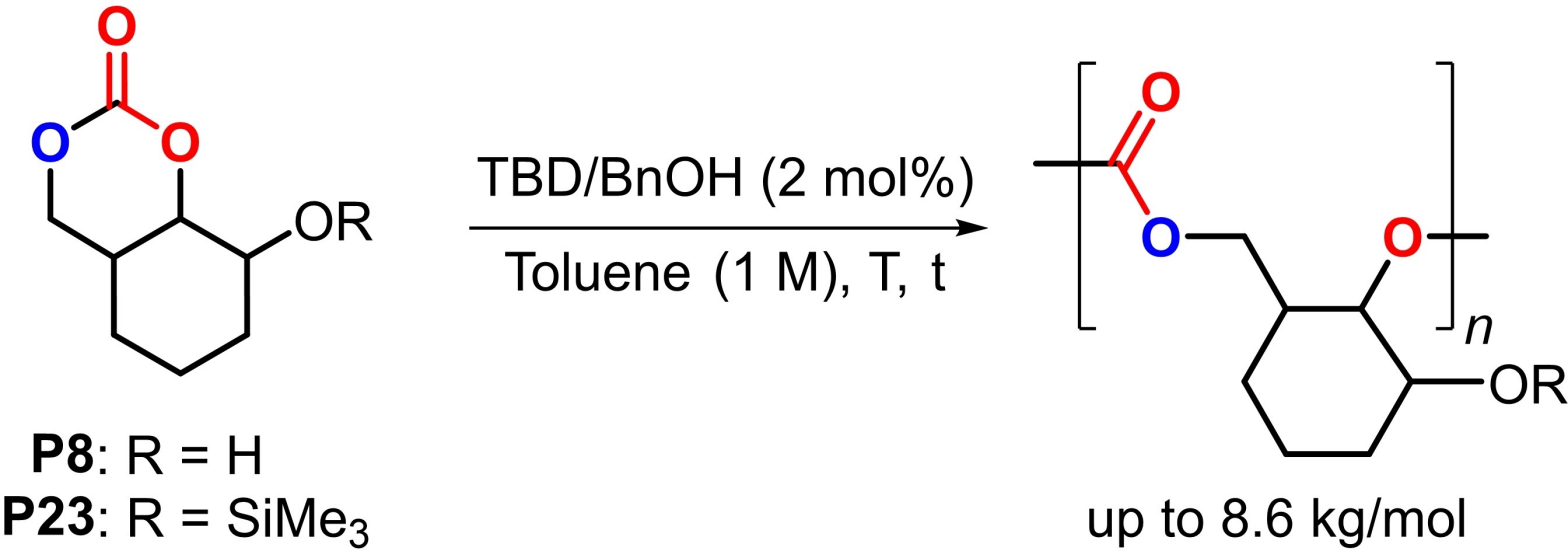						
Entry	Mon.	Solvent	*t*/*T* [h]/[° C]	Conv. [%]^[b]^	Mn^[c]^ [kg mol^−1^]	*Ð* ^[c]^
1^[d]^	**P8**	toluene	20, r.t.	–	1.7	1.47
2^[d]^	**P8**	toluene	48, r.t.	–	0.5	3.83
3	**P23**	toluene	20, r.t.	96	5.9	1.34
4^[e]^	**P23**	toluene	20, r.t.	>99^[f]^	7.8	1.32
5	**P23**	toluene	20, 100	94	5.5	1.20
6	**P23**	DCM	20, r.t.	88	6.4	1.27
7^[e,g]^	**P23**	toluene	20, r.t.	>99	8.6	1.27

[a] For monomer **P8**: 20 mg (1.17 ⋅ 10^−4^ mol), TBD/BnOH=1 : 1, 2 mol %, 117 μL of solvent. For monomer **P23**: 20 mg (8.2×10^−5^ mol), TBD/BnOH=1 : 1, 2 mol %, 82 μL of solvent. For both monomers: time and temperature indicated. Note that only one of the two possible regio‐isomers is shown. [b] Conversion determined by ^1^H NMR (CDCl_3_). [c] *M*_n_ and *Ð* values obtained through GPC analysis in THF using PS standards. [d] Incomplete conversion, accurate determination of monomer conversion not possible due to too much signal overlap. [e] **P23** (200 mg, 8.2×10^−4^ mol), TBD/BnOH=1 : 1, 2 mol %, 820 μL of solvent. [f] Yield of the isolated polycarbonate: 80 %. [g] TBD/BnOH=1 : 1, 1 mol %.

The marked difference in reactivity between the *syn* and *anti* isomer of **1 a** can be rationalized by a stereochemical model where the Al‐complex activates the oxirane at one side of the *anti*‐configured cyclic epoxide. The alcohol (in the presence of a suitable base) enables the activation of CO_2_ from the other face (Scheme 4, lower part; note, molecular structure of *anti*‐**1 o** as a structural model)^[22]^ allowing for ring‐opening and straightforward formation of bicyclic product **P1**. Such reactivity would not be possible with the *syn* isomer of **1 a** (cf., X‐ray of *syn*‐**1 a** and Table 1, entry 2) though a double inversion process is feasible in the presence of TBAB leading to the five‐membered cyclic carbonate **P1^a^**.

**Scheme 4:**
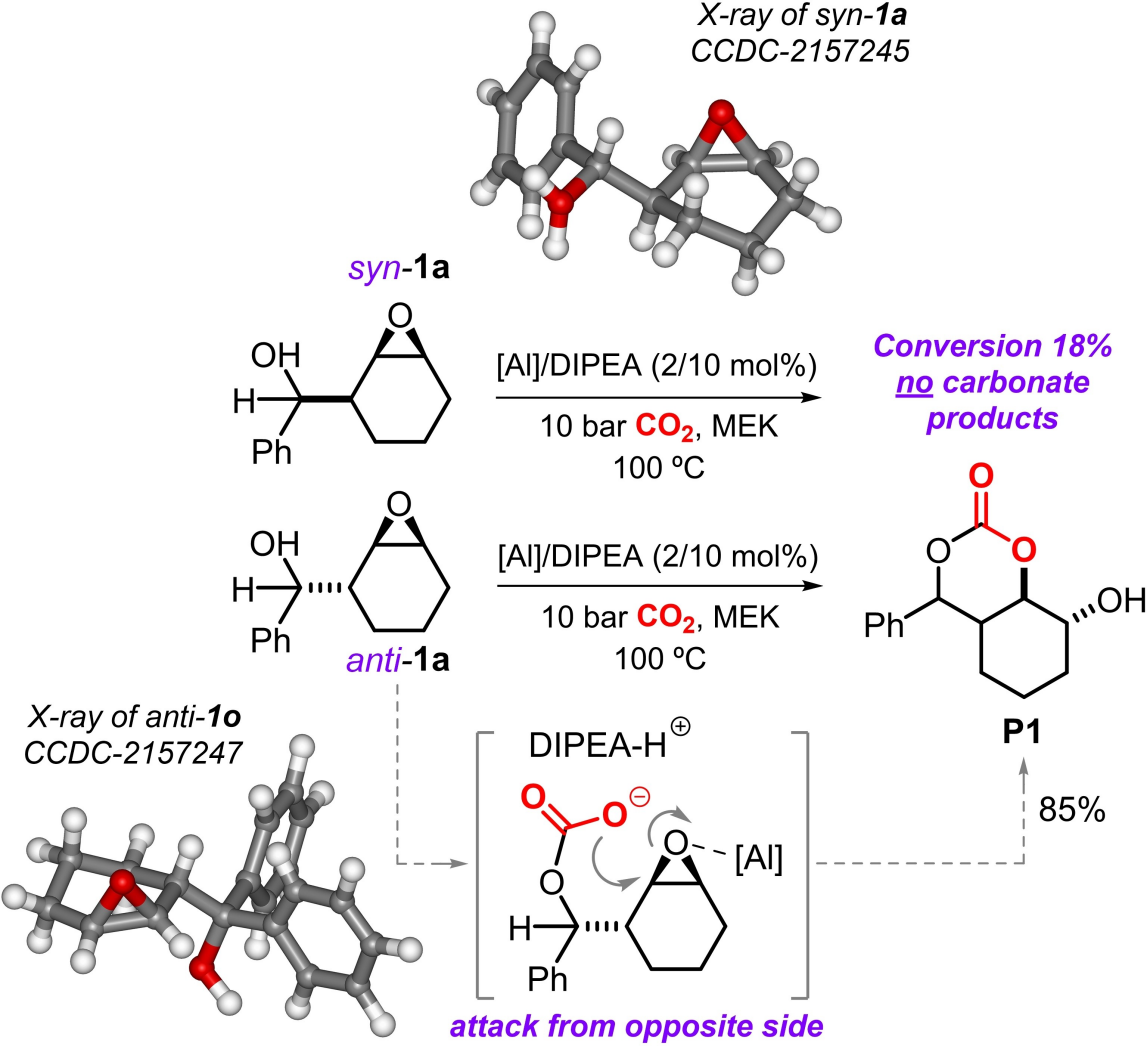
Reactivity comparison between both stereoisomers of **1 a** under similar conditions, and stereochemical model for the conversion of β‐epoxy alcohol *anti*‐**1 a** in the presence of binary catalyst **A**/DIPEA.

In summary, we here describe a novel catalytic approach that allows the coupling between β‐epoxy alcohols and CO_2_ leading to the direct formation of an unusual scope of larger‐ring bicyclic carbonates in good yields. Crucial in this manifold is the stereo‐configuration of the substrate with the *anti*‐isomer leading exclusively to a six‐membered bicyclic carbonate while the corresponding *syn*‐isomer only provides access to five‐membered ring carbonates. The potential of these bicyclic carbonates has been further illustrated in ROP experiments, and the substitution degree and functionality thus hold promise for the design and preparation of a whole new range of (functional) and above all rigidified polycarbonates obtained from CO_2_‐based monomers.

## Conflict of interest

The authors declare no conflict of interest.

## Supporting information

As a service to our authors and readers, this journal provides supporting information supplied by the authors. Such materials are peer reviewed and may be re‐organized for online delivery, but are not copy‐edited or typeset. Technical support issues arising from supporting information (other than missing files) should be addressed to the authors.

Supporting Information

Supporting Information

Supporting Information

Supporting Information

Supporting Information

Supporting Information

Supporting Information

Supporting Information

Supporting Information

## Data Availability

The data that support the findings of this study are available from the corresponding author upon reasonable request.
