# Supplementary material for: A Novel Catalytic Route to Polymerizable Bicyclic Cyclic Carbonate Monomers from Carbon Dioxide
Source: Angew Chem Int Ed Engl. 2022 May 9;61(27):e202205053. doi: 10.1002/anie.202205053 (PMC9323429; doi:10.1002/anie.202205053)

checkCIF/PLATON report

Structure factors have been supplied for datablock(s) qc4-173-c2

THIS REPORT IS FOR GUIDANCE ONLY. IF USED AS PART OF A REVIEW PROCEDURE FOR PUBLICATION, IT SHOULD NOT REPLACE THE EXPERTISE OF AN EXPERIENCED CRYSTALLOGRAPHIC REFEREE.

No syntax errors found. CIF dictionary Interpreting this report

Datablock: qc4-173-c2

```
Bond precision:   = 0.0000 A               Wavelength=0.71075

Cell:              a=18.4631(4)     b=10.33078(17)      c=17.5044(4)
                   alpha=90         beta=118.196(3)     gamma=90
Temperature:       100 K

                  Calculated                   Reported
Volume            2942.57(13)                  2942.58(13)
Space group       P 21/c                       P 21/c
Hall group        -P 2ybc                      -P 2ybc
Moiety formula    C19 H20 O2                   ?
Sum formula       C19 H20 O2                   C38 H40 O4
Mr                280.35                       560.70
Dx,g cm-3         1.266                        1.266
Z                 8                            4
Mu (mm-1)         0.080                        0.080
F000              1200.0                       1200.0
F000'             1200.53
h,k,lmax          25,14,24                     25,14,23
Nref              8277                         7163
Tmin,Tmax         0.995,0.996                  0.464,1.000
Tmin'             0.984

Correction method= # Reported T Limits: Tmin=0.464 Tmax=1.000
AbsCorr = MULTI-SCAN

Data completeness= 0.865           Theta(max)= 29.566

                                                       wR2(reflections)=
R(reflections)= 0.0489( 6335)                          0.2053( 7163)

S = 1.787                    Npar= 710
```

The following ALERTS were generated. Each ALERT has the format
test-name_ALERT_alert-type_alert-level.
Click on the hyperlinks for more details of the test.

Alert level C

SHFSU01_ALERT_2_C The absolute value of parameter shift to su ratio > 0.05
Absolute value of the parameter shift to su ratio given 0.056
Additional refinement cycles may be required.
PLAT080_ALERT_2_C Maximum Shift/Error .... 0.06 Why ?
PLAT085_ALERT_2_C SHELXL Default Weighting Scheme is not Optimized Please Check
PLAT213_ALERT_2_C Atom C5' has ADP max/min Ratio ..... 3.7 prolat
PLAT220_ALERT_2_C NonSolvent Resd 4 C Ueq(max)/Ueq(min) Range 4.0 Ratio
PLAT329_ALERT_4_C Carbon Atom Hybridisation Unclear for .... C3B' Check
PLAT338_ALERT_4_C Small Aver Tau in Cyclohexane C2' -C7' 29.54 Degree
PLAT906_ALERT_3_C Large K Value in the Analysis of Variance ...... 2.715 Check
PLAT911_ALERT_3_C Missing FCF Refl Between Thmin & STh/L= 0.600 63 Report
PLAT918_ALERT_3_C Reflection(s) with I(obs) much Smaller I(calc) . 1 Check

Alert level G

PLAT002_ALERT_2_G Number of Distance or Angle Restraints on AtSite 42 Note
PLAT003_ALERT_2_G Number of Uiso or Uij Restrained non-H Atoms ... 49 Report
PLAT007_ALERT_5_G Number of Unrefined Donor-H Atoms .... 1 Report
PLAT045_ALERT_1_G Calculated and Reported Z Differ by a Factor ... 2 Check
PLAT171_ALERT_4_G The CIF-Embedded .res File Contains EADP Records 3 Report
PLAT175_ALERT_4_G The CIF-Embedded .res File Contains SAME Records 1 Report
PLAT177_ALERT_4_G The CIF-Embedded .res File Contains DELU Records 2 Report
PLAT178_ALERT_4_G The CIF-Embedded .res File Contains SIMU Records 4 Report
PLAT186_ALERT_4_G The CIF-Embedded .res File Contains ISOR Records 4 Report
PLAT230_ALERT_2_G Hirshfeld Test Diff for O1A --C4A . 5.5 s.u.
PLAT230_ALERT_2_G Hirshfeld Test Diff for C16B --C17B . 5.5 s.u.
PLAT300_ALERT_4_G Atom Site Occupancy of O1A Constrained at 0.7 Check
PLAT300_ALERT_4_G Atom Site Occupancy of O2A Constrained at 0.7 Check
PLAT300_ALERT_4_G Atom Site Occupancy of C1A Constrained at 0.7 Check
PLAT300_ALERT_4_G Atom Site Occupancy of C2A Constrained at 0.7 Check
PLAT300_ALERT_4_G Atom Site Occupancy of C3A Constrained at 0.7 Check
PLAT300_ALERT_4_G Atom Site Occupancy of C4A Constrained at 0.7 Check
PLAT300_ALERT_4_G Atom Site Occupancy of C5A Constrained at 0.7 Check
PLAT300_ALERT_4_G Atom Site Occupancy of C6A Constrained at 0.7 Check
PLAT300_ALERT_4_G Atom Site Occupancy of C7A Constrained at 0.7 Check
PLAT300_ALERT_4_G Atom Site Occupancy of C8A Constrained at 0.7 Check
PLAT300_ALERT_4_G Atom Site Occupancy of C9A Constrained at 0.7 Check
PLAT300_ALERT_4_G Atom Site Occupancy of C10A Constrained at 0.7 Check
PLAT300_ALERT_4_G Atom Site Occupancy of C11A Constrained at 0.7 Check
PLAT300_ALERT_4_G Atom Site Occupancy of C12A Constrained at 0.7 Check
PLAT300_ALERT_4_G Atom Site Occupancy of C13A Constrained at 0.7 Check
PLAT300_ALERT_4_G Atom Site Occupancy of C14A Constrained at 0.7 Check
PLAT300_ALERT_4_G Atom Site Occupancy of C15A Constrained at 0.7 Check
PLAT300_ALERT_4_G Atom Site Occupancy of C16A Constrained at 0.7 Check
PLAT300_ALERT_4_G Atom Site Occupancy of C17A Constrained at 0.7 Check
PLAT300_ALERT_4_G Atom Site Occupancy of C18A Constrained at 0.7 Check
PLAT300_ALERT_4_G Atom Site Occupancy of C19A Constrained at 0.7 Check
PLAT300_ALERT_4_G Atom Site Occupancy of H2A Constrained at 0.7 Check
PLAT300_ALERT_4_G Atom Site Occupancy of H3A Constrained at 0.7 Check
PLAT300_ALERT_4_G Atom Site Occupancy of H4A Constrained at 0.7 Check

```
PLAT300_ALERT_4_G Atom Site Occupancy of H9A        Constrained at         0.7 Check
PLAT300_ALERT_4_G Atom Site Occupancy of H10A       Constrained at         0.7 Check
PLAT300_ALERT_4_G Atom Site Occupancy of H11A       Constrained at         0.7 Check
PLAT300_ALERT_4_G Atom Site Occupancy of H12A       Constrained at         0.7 Check
PLAT300_ALERT_4_G Atom Site Occupancy of H13A       Constrained at         0.7 Check
PLAT300_ALERT_4_G Atom Site Occupancy of H15A       Constrained at         0.7 Check
PLAT300_ALERT_4_G Atom Site Occupancy of H16A       Constrained at         0.7 Check
PLAT300_ALERT_4_G Atom Site Occupancy of H17A       Constrained at         0.7 Check
PLAT300_ALERT_4_G Atom Site Occupancy of H18A       Constrained at         0.7 Check
PLAT300_ALERT_4_G Atom Site Occupancy of H2AA       Constrained at         0.7 Check
PLAT300_ALERT_4_G Atom Site Occupancy of H19A       Constrained at         0.7 Check
PLAT300_ALERT_4_G Atom Site Occupancy of H5AA       Constrained at         0.7 Check
PLAT300_ALERT_4_G Atom Site Occupancy of H5AB       Constrained at         0.7 Check
PLAT300_ALERT_4_G Atom Site Occupancy of H6AA       Constrained at         0.7 Check
PLAT300_ALERT_4_G Atom Site Occupancy of H6AB       Constrained at         0.7 Check
PLAT300_ALERT_4_G Atom Site Occupancy of H7AA       Constrained at         0.7 Check
PLAT300_ALERT_4_G Atom Site Occupancy of H7AB       Constrained at         0.7 Check
PLAT300_ALERT_4_G Atom Site Occupancy of O1B        Constrained at         0.6 Check
PLAT300_ALERT_4_G Atom Site Occupancy of O2B        Constrained at         0.6 Check
PLAT300_ALERT_4_G Atom Site Occupancy of C1B        Constrained at         0.6 Check
PLAT300_ALERT_4_G Atom Site Occupancy of C2B        Constrained at         0.6 Check
PLAT300_ALERT_4_G Atom Site Occupancy of C3B        Constrained at         0.6 Check
PLAT300_ALERT_4_G Atom Site Occupancy of C4B        Constrained at         0.6 Check
PLAT300_ALERT_4_G Atom Site Occupancy of C5B        Constrained at         0.6 Check
PLAT300_ALERT_4_G Atom Site Occupancy of C6B        Constrained at         0.6 Check
PLAT300_ALERT_4_G Atom Site Occupancy of C7B        Constrained at         0.6 Check
PLAT300_ALERT_4_G Atom Site Occupancy of C8B        Constrained at         0.6 Check
PLAT300_ALERT_4_G Atom Site Occupancy of C9B        Constrained at         0.6 Check
PLAT300_ALERT_4_G Atom Site Occupancy of C10B       Constrained at         0.6 Check
PLAT300_ALERT_4_G Atom Site Occupancy of C11B       Constrained at         0.6 Check
PLAT300_ALERT_4_G Atom Site Occupancy of C12B       Constrained at         0.6 Check
PLAT300_ALERT_4_G Atom Site Occupancy of C13B       Constrained at         0.6 Check
PLAT300_ALERT_4_G Atom Site Occupancy of C14B       Constrained at         0.6 Check
PLAT300_ALERT_4_G Atom Site Occupancy of C15B       Constrained at         0.6 Check
PLAT300_ALERT_4_G Atom Site Occupancy of C16B       Constrained at         0.6 Check
PLAT300_ALERT_4_G Atom Site Occupancy of C17B       Constrained at         0.6 Check
PLAT300_ALERT_4_G Atom Site Occupancy of C18B       Constrained at         0.6 Check
PLAT300_ALERT_4_G Atom Site Occupancy of C19B       Constrained at         0.6 Check
PLAT300_ALERT_4_G Atom Site Occupancy of H2B        Constrained at         0.6 Check
PLAT300_ALERT_4_G Atom Site Occupancy of H3B        Constrained at         0.6 Check
PLAT300_ALERT_4_G Atom Site Occupancy of H4B        Constrained at         0.6 Check
PLAT300_ALERT_4_G Atom Site Occupancy of H9B        Constrained at         0.6 Check
PLAT300_ALERT_4_G Atom Site Occupancy of H10D       Constrained at         0.6 Check
PLAT300_ALERT_4_G Atom Site Occupancy of H11D       Constrained at         0.6 Check
PLAT300_ALERT_4_G Atom Site Occupancy of H12B       Constrained at         0.6 Check
PLAT300_ALERT_4_G Atom Site Occupancy of H13B       Constrained at         0.6 Check
PLAT300_ALERT_4_G Atom Site Occupancy of H15B       Constrained at         0.6 Check
PLAT300_ALERT_4_G Atom Site Occupancy of H16D       Constrained at         0.6 Check
PLAT300_ALERT_4_G Atom Site Occupancy of H17B       Constrained at         0.6 Check
PLAT300_ALERT_4_G Atom Site Occupancy of H18B       Constrained at         0.6 Check
PLAT300_ALERT_4_G Atom Site Occupancy of H19B       Constrained at         0.6 Check
PLAT300_ALERT_4_G Atom Site Occupancy of H2BA       Constrained at         0.6 Check
PLAT300_ALERT_4_G Atom Site Occupancy of H5BC       Constrained at         0.6 Check
PLAT300_ALERT_4_G Atom Site Occupancy of H5BD       Constrained at         0.6 Check
PLAT300_ALERT_4_G Atom Site Occupancy of H6BC       Constrained at         0.6 Check
PLAT300_ALERT_4_G Atom Site Occupancy of H6BD       Constrained at         0.6 Check
PLAT300_ALERT_4_G Atom Site Occupancy of H7BA       Constrained at         0.6 Check
```

```
PLAT300_ALERT_4_G Atom Site Occupancy of H7BB       Constrained at        0.6 Check
PLAT300_ALERT_4_G Atom Site Occupancy of O1'        Constrained at        0.3 Check
PLAT300_ALERT_4_G Atom Site Occupancy of O2'        Constrained at        0.3 Check
PLAT300_ALERT_4_G Atom Site Occupancy of C1'        Constrained at        0.3 Check
PLAT300_ALERT_4_G Atom Site Occupancy of C2'        Constrained at        0.3 Check
PLAT300_ALERT_4_G Atom Site Occupancy of C3'        Constrained at        0.3 Check
PLAT300_ALERT_4_G Atom Site Occupancy of C4'        Constrained at        0.3 Check
PLAT300_ALERT_4_G Atom Site Occupancy of C5'        Constrained at        0.3 Check
PLAT300_ALERT_4_G Atom Site Occupancy of C6'        Constrained at        0.3 Check
PLAT300_ALERT_4_G Atom Site Occupancy of C7'        Constrained at        0.3 Check
PLAT300_ALERT_4_G Atom Site Occupancy of C8'        Constrained at        0.3 Check
PLAT300_ALERT_4_G Atom Site Occupancy of C9'        Constrained at        0.3 Check
PLAT300_ALERT_4_G Atom Site Occupancy of C10'       Constrained at        0.3 Check
PLAT300_ALERT_4_G Atom Site Occupancy of C11'       Constrained at        0.3 Check
PLAT300_ALERT_4_G Atom Site Occupancy of C12'       Constrained at        0.3 Check
PLAT300_ALERT_4_G Atom Site Occupancy of C13'       Constrained at        0.3 Check
PLAT300_ALERT_4_G Atom Site Occupancy of C14'       Constrained at        0.3 Check
PLAT300_ALERT_4_G Atom Site Occupancy of C15'       Constrained at        0.3 Check
PLAT300_ALERT_4_G Atom Site Occupancy of C16'       Constrained at        0.3 Check
PLAT300_ALERT_4_G Atom Site Occupancy of C17'       Constrained at        0.3 Check
PLAT300_ALERT_4_G Atom Site Occupancy of C18'       Constrained at        0.3 Check
PLAT300_ALERT_4_G Atom Site Occupancy of C19'       Constrained at        0.3 Check
PLAT300_ALERT_4_G Atom Site Occupancy of H2'A       Constrained at        0.3 Check
PLAT300_ALERT_4_G Atom Site Occupancy of H2'        Constrained at        0.3 Check
PLAT300_ALERT_4_G Atom Site Occupancy of H5'A       Constrained at        0.3 Check
PLAT300_ALERT_4_G Atom Site Occupancy of H3'        Constrained at        0.3 Check
PLAT300_ALERT_4_G Atom Site Occupancy of H5'B       Constrained at        0.3 Check
PLAT300_ALERT_4_G Atom Site Occupancy of H4'        Constrained at        0.3 Check
PLAT300_ALERT_4_G Atom Site Occupancy of H6'A       Constrained at        0.3 Check
PLAT300_ALERT_4_G Atom Site Occupancy of H6'B       Constrained at        0.3 Check
PLAT300_ALERT_4_G Atom Site Occupancy of H7'A       Constrained at        0.3 Check
PLAT300_ALERT_4_G Atom Site Occupancy of H7'B       Constrained at        0.3 Check
PLAT300_ALERT_4_G Atom Site Occupancy of H9'        Constrained at        0.3 Check
PLAT300_ALERT_4_G Atom Site Occupancy of H10'       Constrained at        0.3 Check
PLAT300_ALERT_4_G Atom Site Occupancy of H11'       Constrained at        0.3 Check
PLAT300_ALERT_4_G Atom Site Occupancy of H12'       Constrained at        0.3 Check
PLAT300_ALERT_4_G Atom Site Occupancy of H13'       Constrained at        0.3 Check
PLAT300_ALERT_4_G Atom Site Occupancy of H15'       Constrained at        0.3 Check
PLAT300_ALERT_4_G Atom Site Occupancy of H16'       Constrained at        0.3 Check
PLAT300_ALERT_4_G Atom Site Occupancy of H17'       Constrained at        0.3 Check
PLAT300_ALERT_4_G Atom Site Occupancy of H18'       Constrained at        0.3 Check
PLAT300_ALERT_4_G Atom Site Occupancy of H19'       Constrained at        0.3 Check
PLAT300_ALERT_4_G Atom Site Occupancy of O2B'       Constrained at        0.4 Check
PLAT300_ALERT_4_G Atom Site Occupancy of O1B'       Constrained at        0.4 Check
PLAT300_ALERT_4_G Atom Site Occupancy of C1B'       Constrained at        0.4 Check
PLAT300_ALERT_4_G Atom Site Occupancy of C2B'       Constrained at        0.4 Check
PLAT300_ALERT_4_G Atom Site Occupancy of C3B'       Constrained at        0.4 Check
PLAT300_ALERT_4_G Atom Site Occupancy of C4B'       Constrained at        0.4 Check
PLAT300_ALERT_4_G Atom Site Occupancy of C5B'       Constrained at        0.4 Check
PLAT300_ALERT_4_G Atom Site Occupancy of C6B'       Constrained at        0.4 Check
PLAT300_ALERT_4_G Atom Site Occupancy of C7B'       Constrained at        0.4 Check
PLAT300_ALERT_4_G Atom Site Occupancy of C8B'       Constrained at        0.4 Check
PLAT300_ALERT_4_G Atom Site Occupancy of C9B'       Constrained at        0.4 Check
PLAT300_ALERT_4_G Atom Site Occupancy of C10C       Constrained at        0.4 Check
PLAT300_ALERT_4_G Atom Site Occupancy of C11C       Constrained at        0.4 Check
PLAT300_ALERT_4_G Atom Site Occupancy of C12C       Constrained at        0.4 Check
PLAT300_ALERT_4_G Atom Site Occupancy of C13C       Constrained at        0.4 Check
```

```
PLAT300_ALERT_4_G Atom Site Occupancy of C14C       Constrained at        0.4 Check
PLAT300_ALERT_4_G Atom Site Occupancy of C15C       Constrained at        0.4 Check
PLAT300_ALERT_4_G Atom Site Occupancy of C16C       Constrained at        0.4 Check
PLAT300_ALERT_4_G Atom Site Occupancy of C17C       Constrained at        0.4 Check
PLAT300_ALERT_4_G Atom Site Occupancy of C18C       Constrained at        0.4 Check
PLAT300_ALERT_4_G Atom Site Occupancy of C19C       Constrained at        0.4 Check
PLAT300_ALERT_4_G Atom Site Occupancy of H2BB       Constrained at        0.4 Check
PLAT300_ALERT_4_G Atom Site Occupancy of H2B'       Constrained at        0.4 Check
PLAT300_ALERT_4_G Atom Site Occupancy of H3BA       Constrained at        0.4 Check
PLAT300_ALERT_4_G Atom Site Occupancy of H10C       Constrained at        0.4 Check
PLAT300_ALERT_4_G Atom Site Occupancy of H3BB       Constrained at        0.4 Check
PLAT300_ALERT_4_G Atom Site Occupancy of H11C       Constrained at        0.4 Check
PLAT300_ALERT_4_G Atom Site Occupancy of H4B'       Constrained at        0.4 Check
PLAT300_ALERT_4_G Atom Site Occupancy of H12C       Constrained at        0.4 Check
PLAT300_ALERT_4_G Atom Site Occupancy of H5BA       Constrained at        0.4 Check
PLAT300_ALERT_4_G Atom Site Occupancy of H13C       Constrained at        0.4 Check
PLAT300_ALERT_4_G Atom Site Occupancy of H5BB       Constrained at        0.4 Check
PLAT300_ALERT_4_G Atom Site Occupancy of H6BA       Constrained at        0.4 Check
PLAT300_ALERT_4_G Atom Site Occupancy of H15C       Constrained at        0.4 Check
PLAT300_ALERT_4_G Atom Site Occupancy of H6BB       Constrained at        0.4 Check
PLAT300_ALERT_4_G Atom Site Occupancy of H16C       Constrained at        0.4 Check
PLAT300_ALERT_4_G Atom Site Occupancy of H7B'       Constrained at        0.4 Check
PLAT300_ALERT_4_G Atom Site Occupancy of H17C       Constrained at        0.4 Check
PLAT300_ALERT_4_G Atom Site Occupancy of H9B'       Constrained at        0.4 Check
PLAT300_ALERT_4_G Atom Site Occupancy of H18C       Constrained at        0.4 Check
PLAT300_ALERT_4_G Atom Site Occupancy of H19C       Constrained at        0.4 Check
PLAT301_ALERT_3_G Main Residue  Disorder ..............(Resd  1  )       100% Note
PLAT301_ALERT_3_G Main Residue  Disorder ..............(Resd  2  )       100% Note
PLAT301_ALERT_3_G Main Residue  Disorder ..............(Resd  3  )       100% Note
PLAT301_ALERT_3_G Main Residue  Disorder ..............(Resd  4  )       100% Note
PLAT304_ALERT_4_G Non-Integer Number of Atoms in ..... (Resd  1  )      28.70 Check
PLAT304_ALERT_4_G Non-Integer Number of Atoms in ..... (Resd  2  )      24.60 Check
PLAT304_ALERT_4_G Non-Integer Number of Atoms in ..... (Resd  3  )      12.30 Check
PLAT304_ALERT_4_G Non-Integer Number of Atoms in ..... (Resd  4  )      16.40 Check
PLAT398_ALERT_2_G Deviating  C-O-C   Angle From 120 for O1A      .       60.9 Degree
PLAT398_ALERT_2_G Deviating  C-O-C   Angle From 120 for O1B      .       61.4 Degree
PLAT398_ALERT_2_G Deviating  C-O-C   Angle From 120 for O1'      .       60.9 Degree
PLAT398_ALERT_2_G Deviating  C-O-C   Angle From 120 for O1B'     .       61.1 Degree
PLAT720_ALERT_4_G Number of Unusual/Non-Standard Labels ..........         43 Note
PLAT773_ALERT_2_G Check long C-C Bond in CIF: C2B'     --C3B'            1.83 Ang.
PLAT793_ALERT_4_G Model has Chirality at C2A         (Centro SPGR)          R Verify
PLAT793_ALERT_4_G Model has Chirality at C2B         (Centro SPGR)          S Verify
PLAT793_ALERT_4_G Model has Chirality at C3A         (Centro SPGR)          S Verify
PLAT793_ALERT_4_G Model has Chirality at C3B         (Centro SPGR)          R Verify
PLAT793_ALERT_4_G Model has Chirality at C4A         (Centro SPGR)          R Verify
PLAT793_ALERT_4_G Model has Chirality at C4B         (Centro SPGR)          S Verify
PLAT793_ALERT_4_G Model has Chirality at C2'         (Centro SPGR)          S Verify
PLAT793_ALERT_4_G Model has Chirality at C3'         (Centro SPGR)          R Verify
PLAT793_ALERT_4_G Model has Chirality at C4'         (Centro SPGR)          S Verify
PLAT793_ALERT_4_G Model has Chirality at C4B'        (Centro SPGR)          R Verify
PLAT793_ALERT_4_G Model has Chirality at C7B'        (Centro SPGR)          S Verify
PLAT860_ALERT_3_G Number of Least-Squares Restraints ............         522 Note
PLAT870_ALERT_4_G ALERTS Related to Twinning Effects Suppressed ..          ! Info
PLAT883_ALERT_1_G No Info/Value for _atom_sites_solution_primary .     Please Do !
PLAT910_ALERT_3_G Missing # of FCF Reflection(s) Below Theta(Min).          2 Note
PLAT912_ALERT_4_G Missing # of FCF Reflections Above STh/L=  0.600       1033 Note
PLAT933_ALERT_2_G Number of HKL-OMIT Records in Embedded .res File          3 Note
```

```
PLAT965_ALERT_2_G The SHELXL WEIGHT Optimisation has not Converged     Please Check
PLAT992_ALERT_5_G Repd & Actual _reflns_number_gt Values Differ by          2 Check
```

```
   0 ALERT level A = Most likely a serious problem - resolve or explain
   0 ALERT level B = A potentially serious problem, consider carefully
  10 ALERT level C = Check. Ensure it is not caused by an omission or oversight
 208 ALERT level G = General information/check it is not something unexpected

   2 ALERT type 1 CIF construction/syntax error, inconsistent or missing data
  16 ALERT type 2 Indicator that the structure model may be wrong or deficient
   9 ALERT type 3 Indicator that the structure quality may be low
 189 ALERT type 4 Improvement, methodology, query or suggestion
   2 ALERT type 5 Informative message, check
```

It is advisable to attempt to resolve as many as possible of the alerts in all categories. Often the minor alerts point to easily fixed oversights, errors and omissions in your CIF or refinement strategy, so attention to these fine details can be worthwhile. In order to resolve some of the more serious problems it may be necessary to carry out additional measurements or structure refinements. However, the purpose of your study may justify the reported deviations and the more serious of these should normally be commented upon in the discussion or experimental section of a paper or in the "special_details" fields of the CIF. checkCIF was carefully designed to identify outliers and unusual parameters, but every test has its limitations and alerts that are not important in a particular case may appear. Conversely, the absence of alerts does not guarantee there are no aspects of the results needing attention. It is up to the individual to critically assess their own results and, if necessary, seek expert advice.

Publication of your CIF in IUCr journals

A basic structural check has been run on your CIF. These basic checks will be run on all CIFs submitted for publication in IUCr journals (*Acta Crystallographica*, *Journal of Applied Crystallography*, *Journal of Synchrotron Radiation*); however, if you intend to submit to *Acta Crystallographica Section C* or *E* or *IUCrData*, you should make sure that full publication checks are run on the final version of your CIF prior to submission.

Publication of your CIF in other journals

Please refer to the *Notes for Authors* of the relevant journal for any special instructions relating to CIF submission.

PLATON version of 19/02/2022; check.def file version of 19/02/2022

Datablock qc4-173-c2 - ellipsoid plot

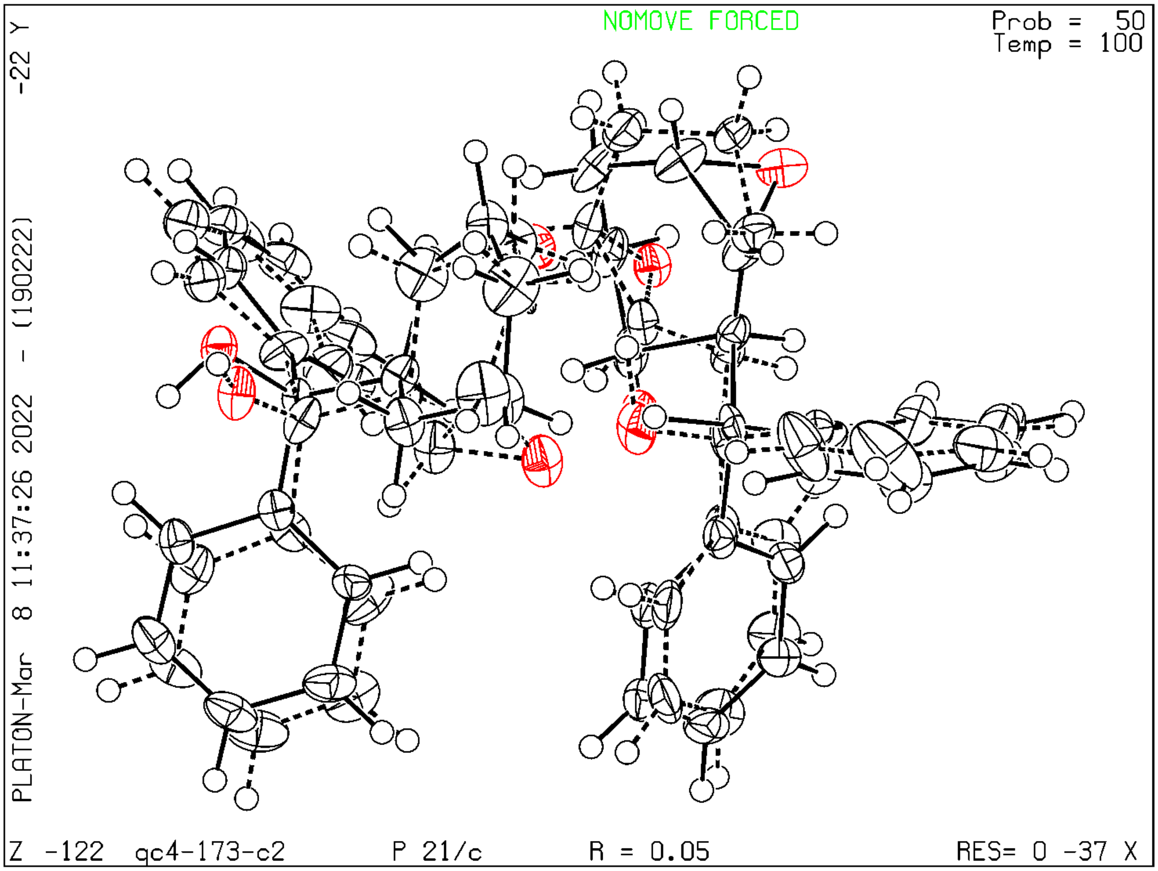

Supplement: Supplementary file 1 — Supporting Information [file ANIE-61-0-s006.pdf]
