# Supplementary material for: A Novel Catalytic Route to Polymerizable Bicyclic Cyclic Carbonate Monomers from Carbon Dioxide
Source: Angew Chem Int Ed Engl. 2022 May 9;61(27):e202205053. doi: 10.1002/anie.202205053 (PMC9323429; doi:10.1002/anie.202205053)

checkCIF/PLATON report

Structure factors have been supplied for datablock(s) mo_qc4131c_0m

THIS REPORT IS FOR GUIDANCE ONLY. IF USED AS PART OF A REVIEW PROCEDURE FOR PUBLICATION, IT SHOULD NOT REPLACE THE EXPERTISE OF AN EXPERIENCED CRYSTALLOGRAPHIC REFEREE.

No syntax errors found. CIF dictionary Interpreting this report

Datablock: mo_qc4131c_0m

Bond precision:	C-C = 0.0017 A		Wavelength=0.71073
Cell:	a=21.648(2)	b=9.2058(9)	c=10.7785(10)
	alpha=90	beta=93.321(3)	gamma=90
Temperature:	100 K		

	Calculated	Reported
Volume	2144.4(4)	2144.4(4)
Space group	C 2/c	C 2/c
Hall group	-C 2yc	-C 2yc
Moiety formula	C13 H16 O2	?
Sum formula	C13 H16 O2	C13 H16 O2
Mr	204.26	204.26
Dx,g cm-3	1.265	1.265
Z	8	8
Mu (mm-1)	0.084	0.084
F000	880.0	880.0
F000'	880.41	
h,k,lmax	30,13,15	30,13,15
Nref	3282	3245
Tmin,Tmax	0.990,0.998	0.594,0.746
Tmin'	0.983	

Correction method= # Reported T Limits: Tmin=0.594 Tmax=0.746
AbsCorr = MULTI-SCAN

Data completeness= 0.989 Theta(max)= 30.551

R(reflections)= 0.0484(2556) wR2(reflections)= 0.1447(3245)

S = 1.047 Npar= 144

The following ALERTS were generated. Each ALERT has the format
test-name_ALERT_alert-type_alert-level.
Click on the hyperlinks for more details of the test.

Alert level C

```
PLAT094_ALERT_2_C Ratio of Maximum / Minimum Residual Density ....       2.06 Report
PLAT911_ALERT_3_C Missing FCF Refl Between Thmin & STh/L=    0.600          5 Report
```

Alert level G

```
PLAT300_ALERT_4_G Atom Site Occupancy of H1O'       Constrained at        0.5 Check
PLAT300_ALERT_4_G Atom Site Occupancy of H1O        Constrained at        0.5 Check
PLAT398_ALERT_2_G Deviating  C-O-C   Angle From 120 for O1       .       60.7 Degree
PLAT414_ALERT_2_G Short Intra D-H..H-X       H1O      ..H7       .       2.11 Ang.
                                                     x,y,z  =      1_555 Check
PLAT720_ALERT_4_G Number of Unusual/Non-Standard Labels ..........          4 Note
PLAT793_ALERT_4_G Model has Chirality at C1          (Centro SPGR)          R Verify
PLAT793_ALERT_4_G Model has Chirality at C2          (Centro SPGR)          S Verify
PLAT793_ALERT_4_G Model has Chirality at C6          (Centro SPGR)          S Verify
PLAT793_ALERT_4_G Model has Chirality at C7          (Centro SPGR)          S Verify
PLAT883_ALERT_1_G No Info/Value for _atom_sites_solution_primary .     Please Do !
PLAT912_ALERT_4_G Missing # of FCF Reflections Above STh/L=  0.600         32 Note
PLAT941_ALERT_3_G Average HKL Measurement Multiplicity ...........        4.0 Low
PLAT978_ALERT_2_G Number C-C Bonds with Positive Residual Density.         13 Info
```

0 **ALERT level A** = Most likely a serious problem - resolve or explain
0 **ALERT level B** = A potentially serious problem, consider carefully
2 **ALERT level C** = Check. Ensure it is not caused by an omission or oversight
13 **ALERT level G** = General information/check it is not something unexpected

1 ALERT type 1 CIF construction/syntax error, inconsistent or missing data
4 ALERT type 2 Indicator that the structure model may be wrong or deficient
2 ALERT type 3 Indicator that the structure quality may be low
8 ALERT type 4 Improvement, methodology, query or suggestion
0 ALERT type 5 Informative message, check

It is advisable to attempt to resolve as many as possible of the alerts in all categories. Often the minor alerts point to easily fixed oversights, errors and omissions in your CIF or refinement strategy, so attention to these fine details can be worthwhile. In order to resolve some of the more serious problems it may be necessary to carry out additional measurements or structure refinements. However, the purpose of your study may justify the reported deviations and the more serious of these should normally be commented upon in the discussion or experimental section of a paper or in the "special_details" fields of the CIF. checkCIF was carefully designed to identify outliers and unusual parameters, but every test has its limitations and alerts that are not important in a particular case may appear. Conversely, the absence of alerts does not guarantee there are no aspects of the results needing attention. It is up to the individual to critically assess their own results and, if necessary, seek expert advice.

Publication of your CIF in IUCr journals

A basic structural check has been run on your CIF. These basic checks will be run on all CIFs submitted for publication in IUCr journals (*Acta Crystallographica*, *Journal of Applied Crystallography*, *Journal of Synchrotron Radiation*); however, if you intend to submit to *Acta Crystallographica Section C* or *E* or *IUCrData*, you should make sure that full publication checks are run on the final version of your CIF prior to submission.

Publication of your CIF in other journals

Please refer to the *Notes for Authors* of the relevant journal for any special instructions relating to CIF submission.

PLATON version of 19/02/2022; check.def file version of 19/02/2022

Datablock mo_qc4131c_0m - ellipsoid plot

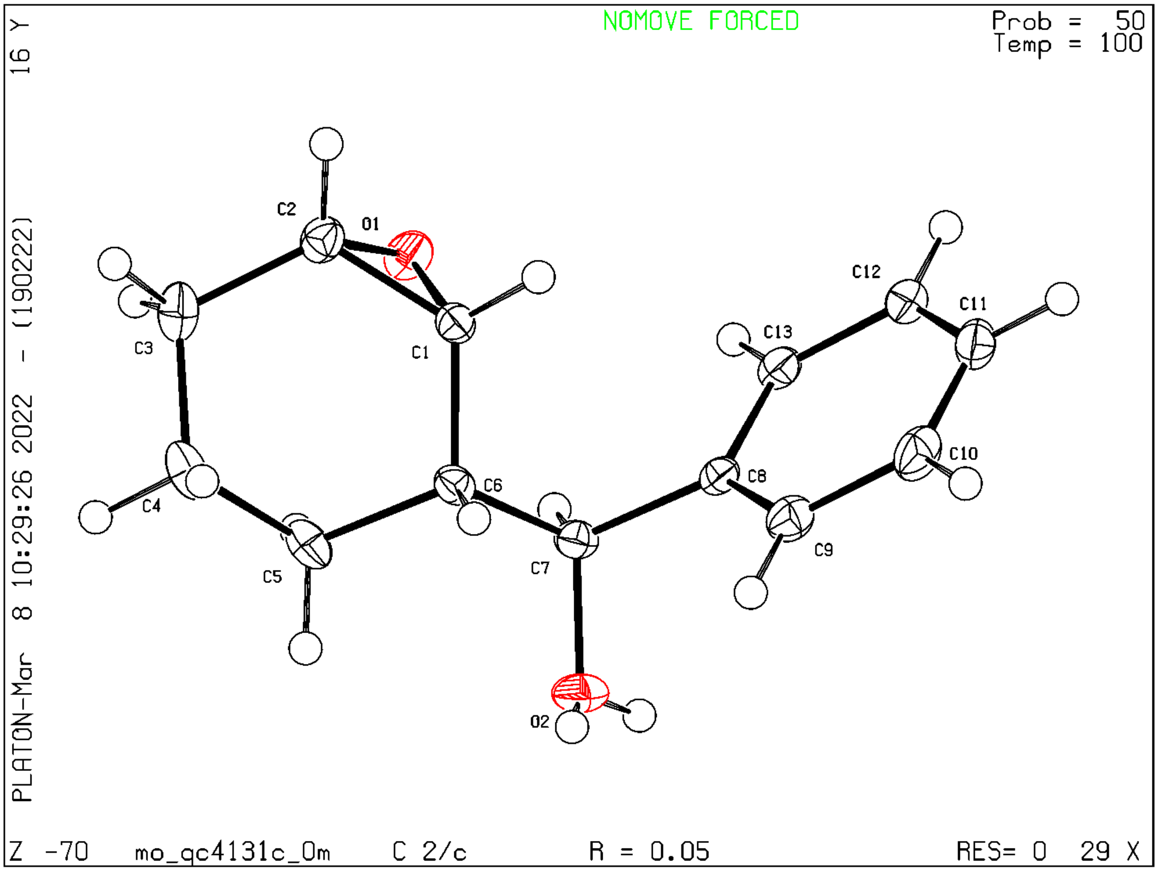

Supplement: Supplementary file 2 — Supporting Information [file ANIE-61-0-s005.pdf]
