# Supplementary material for: A Novel Catalytic Route to Polymerizable Bicyclic Cyclic Carbonate Monomers from Carbon Dioxide
Source: Angew Chem Int Ed Engl. 2022 May 9;61(27):e202205053. doi: 10.1002/anie.202205053 (PMC9323429; doi:10.1002/anie.202205053)

checkCIF/PLATON report

Structure factors have been supplied for datablock(s) mo_qc41552_0m

THIS REPORT IS FOR GUIDANCE ONLY. IF USED AS PART OF A REVIEW PROCEDURE FOR PUBLICATION, IT SHOULD NOT REPLACE THE EXPERTISE OF AN EXPERIENCED CRYSTALLOGRAPHIC REFEREE.

No syntax errors found. CIF dictionary Interpreting this report

Datablock: mo_qc41552_0m

Bond precision: C-C = 0.0053 A Wavelength=0.71073

Cell:	a=11.3607(17)	b=11.9291(18)	c=18.900(3)
	alpha=88.466(3)	beta=76.159(3)	gamma=89.416(3)
Temperature:	100 K		

	Calculated	Reported
Volume	2486.1(7)	2486.2(6)
Space group	P -1	P -1
Hall group	-P 1	-P 1
Moiety formula	C13 H19.41 O4, 3(C13 H20 O4), 1.196(H2 O)	?
Sum formula	C52 H81.80 O17.20	C52 H81.82 O17.18
Mr	982.11	981.81
Dx,g cm-3	1.312	1.312
Z	2	2
Mu (mm-1)	0.097	0.097
F000	1062.7	1062.0
F000'	1063.34	
h,k,lmax	14,15,23	14,15,23
Nref	10561	10474
Tmin,Tmax	0.996,0.999	0.596,0.745
Tmin'	0.996	

Correction method= # Reported T Limits: Tmin=0.596 Tmax=0.745
AbsCorr = MULTI-SCAN

Data completeness= 0.992 Theta(max)= 26.730

R(reflections)= 0.0860(7619) wR2(reflections)= 0.2423(10474)

S = 1.087 Npar= 743

The following ALERTS were generated. Each ALERT has the format
test-name_ALERT_alert-type_alert-level.
Click on the hyperlinks for more details of the test.

Alert level B

```
PLAT097_ALERT_2_B Large Reported Max.  (Positive) Residual Density       1.03 eA-3
```

Author Response: The large residual positive electron density is related to the disorder in the crystal which could not be refined properly for this part of the structure. This structure was considered suitable for publication despite the presence of electron density.

```
PLAT417_ALERT_2_B Short Inter D-H..H-D       H1WA     ..H40A     .       2.08 Ang.
                                               1-x,1-y,2-z  =      2_667 Check
```

Author Response: The hydrogen atoms of -OH groups and water molecules are forming a network of hydrogen bonds which result in a relatively short distance between H1WA and H40A. The mentioned distance was considered as correct.

Alert level C

```
DIFMX02_ALERT_1_C  The maximum difference density is > 0.1*ZMAX*0.75
            The relevant atom site should be identified.
PLAT041_ALERT_1_C Calc. and Reported SumFormula    Strings  Differ     Please Check
PLAT077_ALERT_4_C Unitcell Contains Non-integer Number of Atoms ..     Please Check
PLAT340_ALERT_3_C Low Bond Precision on  C-C Bonds ..............    0.00528 Ang.
PLAT355_ALERT_3_C Long   O-H (X0.82,N0.98A)  O4C      - H3P      .       1.06 Ang.
PLAT906_ALERT_3_C Large K Value in the Analysis of Variance ......      8.936 Check
PLAT906_ALERT_3_C Large K Value in the Analysis of Variance ......      2.241 Check
PLAT911_ALERT_3_C Missing FCF Refl Between Thmin & STh/L=    0.600         24 Report
PLAT975_ALERT_2_C Check Calcd Resid. Dens.  1.09Ang From O4B     .       0.58 eA-3
PLAT975_ALERT_2_C Check Calcd Resid. Dens.  1.01Ang From O3D     .       0.43 eA-3
PLAT975_ALERT_2_C Check Calcd Resid. Dens.  0.81Ang From O2C     .       0.42 eA-3
PLAT975_ALERT_2_C Check Calcd Resid. Dens.  0.86Ang From O4C     .       0.40 eA-3
```

Alert level G

```
FORMU01_ALERT_2_G  There is a discrepancy between the atom counts in the
            _chemical_formula_sum and the formula from the _atom_site* data.
            Atom count from _chemical_formula_sum:C52 H81.82 O17.18
            Atom count from the _atom_site data:  C52 H81.80399 O17.19599
CELLZ01_ALERT_1_G Difference between formula and atom_site contents detected.
CELLZ01_ALERT_1_G ALERT: check formula stoichiometry or atom site occupancies.
           From the CIF: _cell_formula_units_Z    2
           From the CIF: _chemical_formula_sum  C52 H81.82 O17.18
           TEST: Compare cell contents of formula and atom_site data

           atom    Z*formula  cif sites diff
```

```
          C       104.00    104.00    0.00
          H       163.64    163.61    0.03
          O        34.36     34.39   -0.03
PLAT002_ALERT_2_G Number of Distance or Angle Restraints on AtSite          3 Note
PLAT003_ALERT_2_G Number of Uiso or Uij Restrained non-H Atoms ...         28 Report
PLAT007_ALERT_5_G Number of Unrefined Donor-H Atoms .............           6 Report
PLAT068_ALERT_1_G Reported F000 Differs from Calcd (or Missing)...     Please Check
PLAT083_ALERT_2_G SHELXL Second Parameter in WGHT  Unusually Large       5.92 Why ?
PLAT154_ALERT_1_G The s.u.'s on the Cell Angles are Equal ..(Note)      0.003 Degree
PLAT172_ALERT_4_G The CIF-Embedded .res File Contains DFIX Records          3 Report
PLAT177_ALERT_4_G The CIF-Embedded .res File Contains DELU Records          1 Report
PLAT178_ALERT_4_G The CIF-Embedded .res File Contains SIMU Records          1 Report
PLAT186_ALERT_4_G The CIF-Embedded .res File Contains ISOR Records          1 Report
PLAT301_ALERT_3_G Main Residue  Disorder .............(Resd  1  )         65% Note
PLAT302_ALERT_4_G Anion/Solvent/Minor-Residue Disorder (Resd  6  )       100% Note
PLAT304_ALERT_4_G Non-Integer Number of Atoms in ..... (Resd  1  )      36.41 Check
PLAT304_ALERT_4_G Non-Integer Number of Atoms in ..... (Resd  6  )       0.59 Check
PLAT398_ALERT_2_G Deviating  C-O-C   Angle From 120 for O2'      .      132.5 Degree
PLAT410_ALERT_2_G Short Intra H...H Contact  H1D      ..H4DB     .       2.11 Ang.
                                                  x,y,z  =      1_555 Check
PLAT410_ALERT_2_G Short Intra H...H Contact  H8DB     ..H10'     .       2.13 Ang.
                                                  x,y,z  =      1_555 Check
PLAT415_ALERT_2_G Short Inter D-H..H-X       H6AA     ..H2WB     .       1.77 Ang.
                                                x,y,1+z  =      1_556 Check
PLAT415_ALERT_2_G Short Inter D-H..H-X       H8AB     ..H2WB     .       2.06 Ang.
                                                x,y,1+z  =      1_556 Check
PLAT415_ALERT_2_G Short Inter D-H..H-X       H1C      ..H2WA     .       1.69 Ang.
                                             1-x,-y,1-z  =      2_656 Check
PLAT720_ALERT_4_G Number of Unusual/Non-Standard Labels ..........         45 Note
PLAT790_ALERT_4_G Centre of Gravity not Within Unit Cell: Resd.  #          2 Note
             C13 H20 O4
PLAT793_ALERT_4_G Model has Chirality at C1A         (Centro SPGR)          R Verify
PLAT793_ALERT_4_G Model has Chirality at C1B         (Centro SPGR)          S Verify
PLAT793_ALERT_4_G Model has Chirality at C1C         (Centro SPGR)          S Verify
PLAT793_ALERT_4_G Model has Chirality at C1D         (Centro SPGR)          R Verify
PLAT793_ALERT_4_G Model has Chirality at C9A         (Centro SPGR)          R Verify
PLAT793_ALERT_4_G Model has Chirality at C9B         (Centro SPGR)          S Verify
PLAT793_ALERT_4_G Model has Chirality at C9C         (Centro SPGR)          S Verify
PLAT793_ALERT_4_G Model has Chirality at C9D         (Centro SPGR)          R Verify
PLAT793_ALERT_4_G Model has Chirality at C13A        (Centro SPGR)          R Verify
PLAT793_ALERT_4_G Model has Chirality at C13B        (Centro SPGR)          S Verify
PLAT793_ALERT_4_G Model has Chirality at C13C        (Centro SPGR)          S Verify
PLAT793_ALERT_4_G Model has Chirality at C13D        (Centro SPGR)          R Verify
PLAT860_ALERT_3_G Number of Least-Squares Restraints .............        653 Note
PLAT883_ALERT_1_G No Info/Value for _atom_sites_solution_primary .     Please Do !
PLAT912_ALERT_4_G Missing # of FCF Reflections Above STh/L=  0.600         64 Note
PLAT930_ALERT_2_G FCF-based   Twin Law         [ 0 1 0] Est.d BASF       0.19 Check
PLAT931_ALERT_5_G CIFcalcFCF  Twin Law         [ 0 1 0] Est.d BASF       0.19 Check
PLAT933_ALERT_2_G Number of HKL-OMIT Records in Embedded .res File          4 Note
PLAT941_ALERT_3_G Average HKL Measurement Multiplicity ...........        3.5 Low
PLAT978_ALERT_2_G Number C-C Bonds with Positive Residual Density.          5 Info
PLAT992_ALERT_5_G Repd & Actual _reflns_number_gt Values Differ by          2 Check
```

---

0 **ALERT level A** = Most likely a serious problem - resolve or explain
2 **ALERT level B** = A potentially serious problem, consider carefully
12 **ALERT level C** = Check. Ensure it is not caused by an omission or oversight

```
46 ALERT level G = General information/check it is not something unexpected

 7 ALERT type 1 CIF construction/syntax error, inconsistent or missing data
19 ALERT type 2 Indicator that the structure model may be wrong or deficient
 8 ALERT type 3 Indicator that the structure quality may be low
23 ALERT type 4 Improvement, methodology, query or suggestion
 3 ALERT type 5 Informative message, check
```

It is advisable to attempt to resolve as many as possible of the alerts in all categories. Often the minor alerts point to easily fixed oversights, errors and omissions in your CIF or refinement strategy, so attention to these fine details can be worthwhile. In order to resolve some of the more serious problems it may be necessary to carry out additional measurements or structure refinements. However, the purpose of your study may justify the reported deviations and the more serious of these should normally be commented upon in the discussion or experimental section of a paper or in the "special_details" fields of the CIF. checkCIF was carefully designed to identify outliers and unusual parameters, but every test has its limitations and alerts that are not important in a particular case may appear. Conversely, the absence of alerts does not guarantee there are no aspects of the results needing attention. It is up to the individual to critically assess their own results and, if necessary, seek expert advice.

Publication of your CIF in IUCr journals

A basic structural check has been run on your CIF. These basic checks will be run on all CIFs submitted for publication in IUCr journals (*Acta Crystallographica*, *Journal of Applied Crystallography*, *Journal of Synchrotron Radiation*); however, if you intend to submit to *Acta Crystallographica Section C* or *E* or *IUCrData*, you should make sure that full publication checks are run on the final version of your CIF prior to submission.

Publication of your CIF in other journals

Please refer to the *Notes for Authors* of the relevant journal for any special instructions relating to CIF submission.

PLATON version of 19/02/2022; check.def file version of 19/02/2022

Datablock mo_qc41552_0m - ellipsoid plot

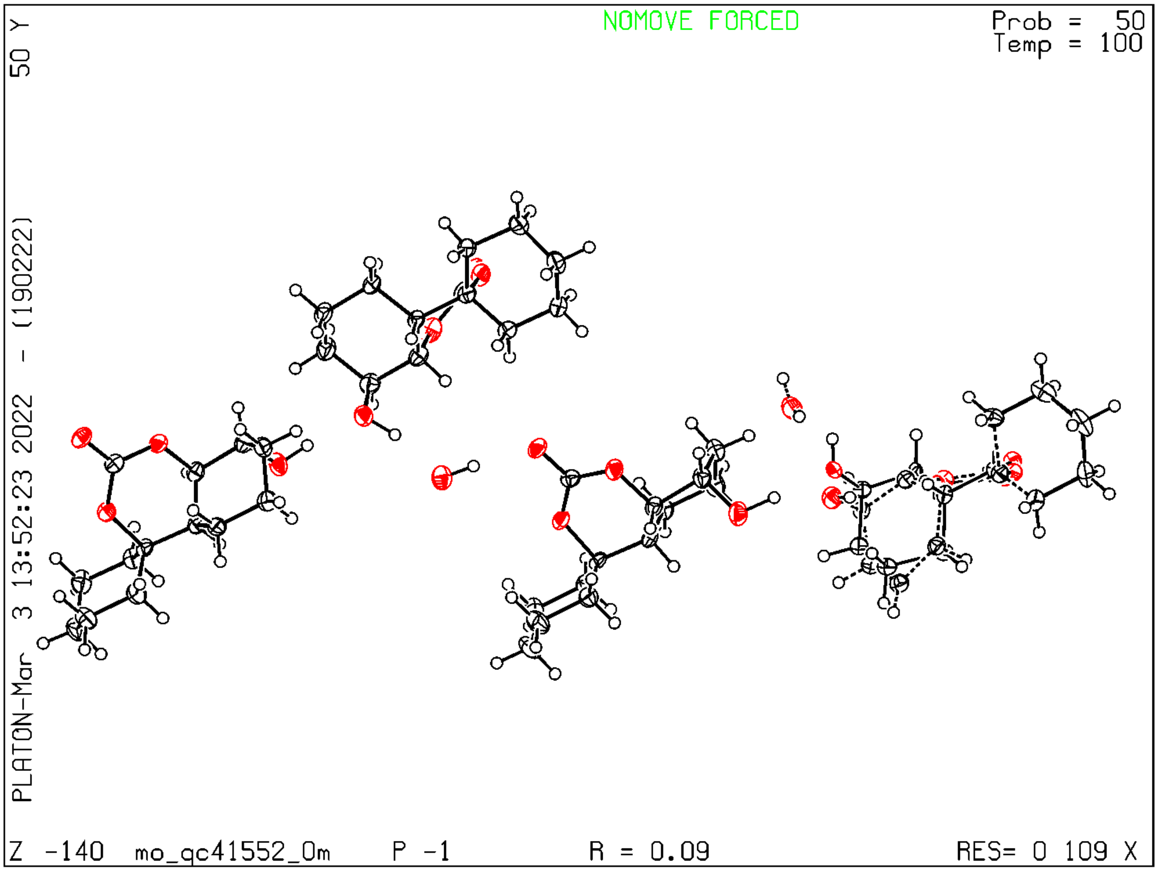

Supplement: Supplementary file 4 — Supporting Information [file ANIE-61-0-s004.pdf]
