# Supplementary material for: A Novel Catalytic Route to Polymerizable Bicyclic Cyclic Carbonate Monomers from Carbon Dioxide
Source: Angew Chem Int Ed Engl. 2022 May 9;61(27):e202205053. doi: 10.1002/anie.202205053 (PMC9323429; doi:10.1002/anie.202205053)
Supplement: Supplementary file 5 — Supporting Information [file ANIE-61-0-s008.pdf]

Angewandte Chemie
Eine Zeitschrift der Gesellschaft Deutscher Chemiker

Supporting Information

A Novel Catalytic Route to Polymerizable Bicyclic Cyclic Carbonate Monomers from Carbon Dioxide

*C. Qiao, W. Shi, A. Brandolese, J. Benet-Buchholz, E. C. Escudero-Adán, A. W. Kleij**

Supporting Information

Contents:

Page S2: General comments

Page S3: Experimental procedures for the synthesis of cycloalkenes

Page S5: Experimental procedures for the synthesis of cyclic epoxy alcohol

Page S6: Experimental procedures for the 6-membered cyclic carbonates

Page S7: Full screening Table S1

Page S9: Further screening results with substrate **1o** and **1u**

Page S10: Experimental procedures for the product diversification

Page S13: Characterization data for all new and relevant compounds

Page S35: References

Page S36: IR, ^{1}H NMR, ^{13}C NMR and ^{19}F NMR spectra

Page S127: ROP studies

Page S140: X-ray molecular structures

S2. General comments

All reagents were used as received from commercial suppliers (Aldrich, Acros or TCI) unless otherwise stated. Carbon dioxide was purchased from PRAXAIR and used without further purification. NMR-spectra were recorded on Bruker AV-400 or AV-500 spectrometers. The residual solvent signals were used as references for ^1H and ^{13}C NMR spectra ($CDCl_3$: δ^H = 7.26 ppm, δ^C = 77.16 ppm, $(CD_3)_2SO$: δ^H = 2.5 ppm, δ^C = 39.52 ppm). ^{19}F NMR spectra were not calibrated by an internal reference. FT-IR measurements were carried out on a Bruker Optics FTIR-ATR TR0 spectrometer. Exact mass analyses and X-ray diffraction studies were performed by the Research Support Area (RSA) at ICIQ. Solvents were dried using an Innovative Technology PURE SOLV solvent purification system.

S3. Experimental procedures for the synthesis of cycloalkenes

Non-commercial allyl bromides were synthesized using literature procedures.[1] These allyl bromides are thermally unstable and become brownish while decomposing when exposed to air and/or under light. They were therefore freshly prepared before use.

Method A: cycloalkenes with a primary alcohol group [1]

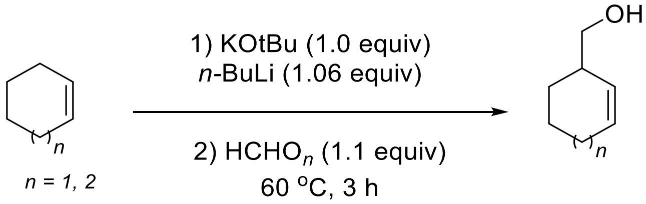

n-BuLi (1.6 M in hexanes, 20 mL, 32 mmol) was added to a degassed suspension of KO*t*Bu (3.39 g, 30.2 mmol) in cyclohexene (27 mL) or cycloheptene (30 mmol in 25 mL cyclohexane) under a nitrogen atmosphere. The reaction mixture was kept below 15 ºC over a period of 2 h, and then allowed to warm to r.t. over 16 h. The resultant suspension was cooled to 0 °C and $(HCHO)_n$ (1 g, 33.2 mmol) was added (**Caution**: exothermic!). The reaction mixture was heated to 60 ºC for 3 h, then cooled to 0 °C and quenched with a saturated aqueous $NaHCO_3$ solution (20 mL). The mixture was extracted with CH_2Cl_2 (3 × 20 mL). The combined organic extracts were washed sequentially with a saturated aqueous $NaHCO_3$ solution (20 mL) and brine (20 mL), dried and concentrated in vacuo to give the desired product. All the cycloalkenes with primary alcohol groups synthesized using this method were used directly for the next step without further purification.

Method B: cycloalkenes with a secondary alcohol group [2]

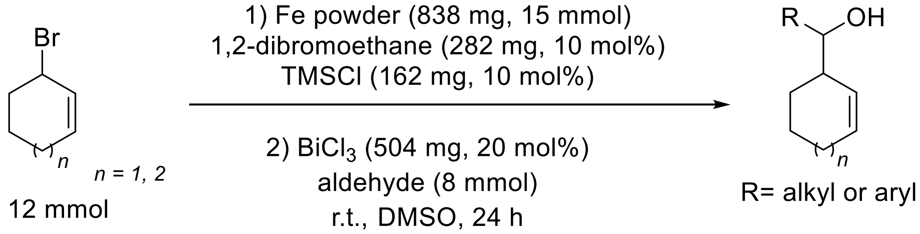

To a 10 mL Schlenk flask was sequentially added iron powder (838 mg, 15 mmol) and DMSO (10 mL). Then the iron was activated by the addition of 1,2-dibromoethane (282 mg, 10 mol%) and TMSCl (162 mg, 10 mol%). After stirring for 30 min, $BiCl_3$ (504 mg, 1.6 mmol), the cyclic allyl bromide (12 mmol) and aldehyde (8 mmol) were sequentially added to the reaction mixture. The suspension was vigorously stirred at r.t. for 24 h before quenching it with a saturated aqueous $NaHCO_3$ solution (30 mL) following extraction with ethyl acetate (3 × 20 mL). The combined extracts were washed with brine (30 mL), dried over anhydrous Na_2SO_4 and concentrated in vacuo.

The residue obtained was purified by silica gel column chromatography using ethyl acetate (EA)/hexane as eluent to give the pure products.

Method C: cycloalkenes with a tertiary alcohol group [3]

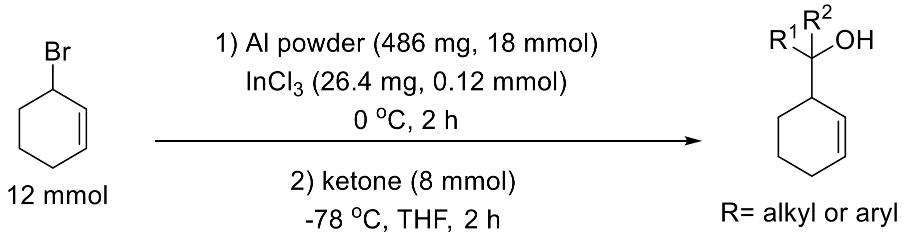

Al-powder (486 mg, 18 mmol) and $InCl_3$ (26.4 mg, 0.12 mmol) were placed in an argon-flushed dry flask. After THF (15 mL) was added, a solution of a cyclic allyl bromide (12 mmol) in THF (15 mL) was added with a syringe pump over 1 h at 0 °C and the resulting solution was stirred at 0 °C for another hour. A solution of the ketone reagent (8 mmol) in THF (5 mL) was added to the mixture at -78 °C via a syringe and the resulting mixture was stirred at -78 °C for 2 h. Standard work-up as under methods **A** and **B**, and purification by flash chromatography over silica was carried out to isolate the target compounds.

S5. Experimental procedures for the synthesis of cyclic epoxy alcohols

Anti-isomers

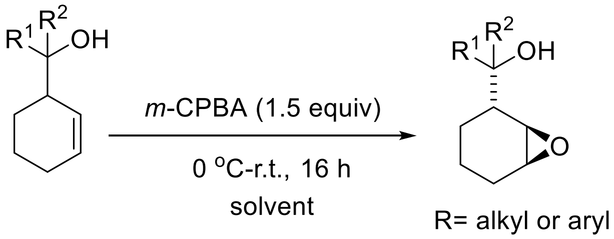

The respective cycloalkene alcohol (3 mmol) was dissolved (DCM/MeOH, 15 mL) at 0 °C and *m*-CPBA (1.5 equiv., 4.5 mmol, 1.0 g) was added. The mixture was allowed to warm to r.t. and was stirred for 16 h. The reaction mixture was washed with Na_2CO_3 (2 × 30 mL) and was dried over Na_2SO_4. The solvent was removed *in vacuo* and the crude product was purified by flash chromatography. **Note**: under this condition, *syn* and *anti*-isomers were obtained at the same time, in most cases, they can be separated by flash chromatography.

Syn-isomers [4]

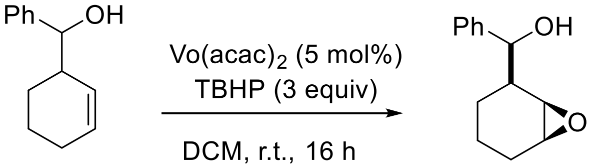

The respective epoxy alcohol (5 mmol, 1.0 equiv) and Vo(acac)$_2$ (5 mol%, 32 mg) were dissolved in DCM (50 mL) at 0 °C, then TBHP (*tert*-butyl hydroperoxide solution, 5.0-6.0 M in decane, 3 mL) was added. The mixture was stirred at r.t. for 16 h. After the reaction, the solvent was removed under vacuum and the crude product was purified by flash chromatography employing 10% EA in hexane as eluent.

S6. Experimental procedure for the 6-membered cyclic carbonates

The Al(III) aminotriphenolate complexes **A** and **B** were prepared according to a literature procedure.[5]

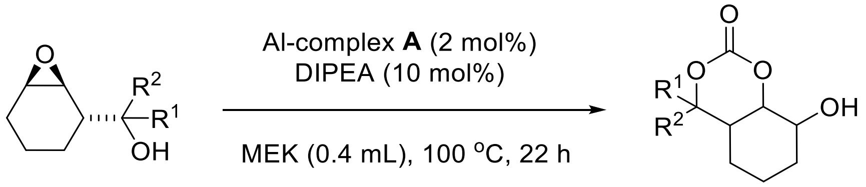

In a typical experiment, a stainless-steel HEL-multireactor was charged with the epoxide (0.1 mmol) dissolved in MEK (400 μL) and Al-complex **A** (0.002 mmol 2 mol%, 1.6 mg) and DIPEA (0.01 mmol 10 mol%, 1.3 mg) were added. The reactor was purged twice with CO_2 (10 bar) and then charged with CO_2 (10 bar). The mixture was stirred at 100 °C for 22 h, then cooled with an ice/water bath and carefully depressurized. The solvent was removed *in vacuo* and the resulting product was purified by flash chromatography employing 30-70% EA in hexane as a gradient eluent.

NB: **A scale-up reaction** for product **P8** was also performed on a 10 mmol scale (substrate-based) in 5 mL of MEK in a 30 mL stainless steel reactor. Yield 1.36 g (79%).

S7. Full screening Table S1

Table S1

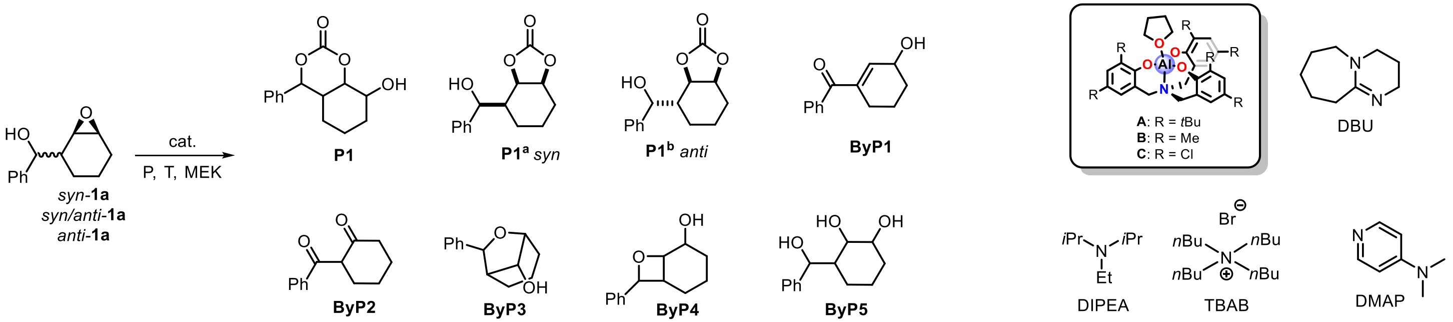

Entry	1a	Cat. [mol%]	P/T [bar/°C]	Conv [%]	**P1** [%][d]	**P1**[a] [%][d]	**P1**[b] [%][d]	**ByP1** [%]	**ByP2** [%]	**ByP3** [%][d]	**ByP4** [%][d]	**ByP5** [%]
1	*syn*	**A**/DIPEA, 10	30/50	<1	–	–	–	–	–	–	–	–
2	*syn*	**A**/DIPEA, 10	10/75	<5	–	–	–	–	–	–	–	–
3	*syn*	**A**/DIPEA, 10	10/100	18	0	0	0	5.4	0	0	0	8.6
4	*syn*	**A**/TBAB, 5	10/100	84	0	35 (29)	0	7	0	0	16	9
5	*syn*	TBAB, 5	10/100	74	0	**37**	0	7	0	0	7	10
6	*syn*	DBU, 10	10/100	94	0	0	26 (21)	3	0	0	0	13
7	*syn*	**B**/TBAB, 5	10/100	>99	0	0	0	5	0	0	22	10
8	*syn*	DMAP, 10	10/100	>99	0	0	**39 (37)**	trace	trace	0	0	10
9	*syn*	**A**/DBU, 10	10/100	>99	0	0	0	0	trace	46(46)	0	12
10[b]	*syn*	TBAB/DBU	10/100	95	0	12	29	0	7.3	0	0	7.3

Continuation of **Table S1**

11	[c]	**A**/DIPEA, 10	10/100	61	(20)	0	0	0	0	0	(23)	0
12	[c]	**A**/TBAB, 5	10/100	>99	(20)	18	0	0	0	0	61	0
13	*anti*	**A**/TBAB, 5	10/100	>99	(83)	0	0	0	0	0	0	trace
14	*anti*	**A**/DIPEA, 10	10/75	70	65	0	0	0	0	0	0	0
15	*anti*	**A**/DIPEA, 2	10/100	83	75	0	0	5	0	0	0	trace
16	*anti*	**A**/DIPEA, 6	10/100	93	76	0	0	4	0	0	0	9
17	*anti*	**A**/DIPEA, 10	10/100	95	**(85)**	0	0	0	0	0	0	0
18	*anti*	**B**/DIPEA, 10	10/100	>99	(77)	0	0	0	0	0	0	0
19	*anti*	**C**/DIPEA, 10	10/100	>99	trace	0	0	0	0	0	0	8
20	*anti*	TBAB, 5	10/100	74	11	0	21	0	0	0	0	0
21	*anti*	DMAP, 10	10/100	14	0	0	0	0	0	0	0	0
22	*anti*	DBU, 10	10/100	>99	0	0	0	0	0	0	0	74
23	*anti*	**A**	10/100	36	11	0	0	0	0	0	0	Trace
24	*anti*	DIPEA, 10	10/100	14	0	0	0	0	0	0	0	0

[a] Reaction performed under the indicated pressure and temperature, MEK as solvent (0.4 mL), *syn*-**1a** or **1a** (0.5 mmol) or *anti*-**1a** (0.2 mmol), Al-complex **A** or **B** (2 mol%), additive (indicated), 22 h. The amount of **P1**, **P1**$^{\mathbf{a}}$ and **P1**$^{\mathbf{b}}$ and the overall conversion of **1a** was determined by ^{1}H NMR ($CDCl_3$). [b] TBAB (5 mol%) and DBU (10 mol%). [c] *Syn/anti* (3:1 mixture) **1a** was used. [d] In brackets the isolate yield of product.

<u>Note</u>: all the by-products (**ByP1** to **ByP5**) reported in **Table S1** were isolated and characterized/identified by ^{1}H NMR, ^{13}C NMR, IR and HRMS. These data are provided below in the respective section from page S32 onwards.

S9. Further screening results with substrate 1o and 1u

Table S2. Further screening results with substrate **1o** [a]

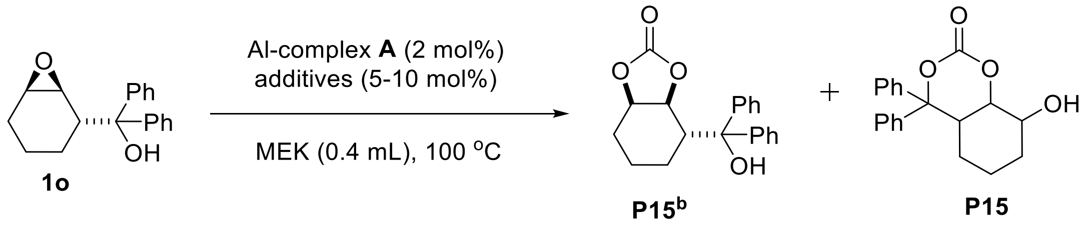

Entry	Additives (mol%)	t (h)	Yield of **P15** [b,c]	Yield of **P15** [c]
1	DIPEA (10)	22	/	trace
2	DIPEA (10)	72	/	19%
3	TBAB (5)	22	(18%)[b]	(39%)[b]
4	TBAB (5)	72	63%	35%

[a] Substrate **1o** (0.1 mmol), CO_2 (10 bar), Al-complex **A** (2 mol%), additive (5-10 mol%), MEK (0.4 mL). [b] Yield of the isolated product. [c] All reported yields and conversions are based on ^1H NMR ($CDCl_3$) measurements using mesitylene as an internal standard.

Table S3. Further screening results with substrate **1u** [a]

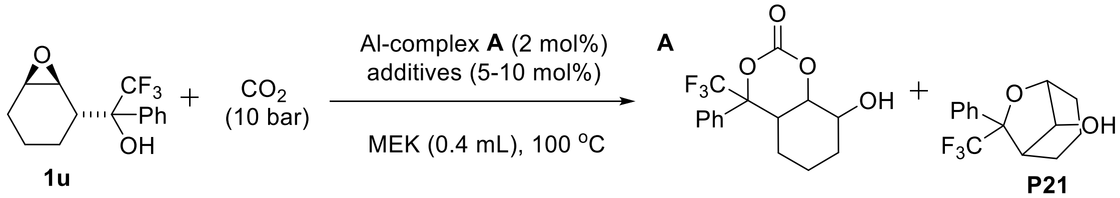

Entry	Additives (mol%)	t (h)	Yield of 6MCC [b]	**P21** [b]
1	DIPEA (10)	22 h	trace	(18%) [c]
2	DIPEA (10)	72 h	trace	22%
3	TBAB (5)	72 h	trace	(56%) [c]

[a] Substrate **1u** (0.1 mmol), CO_2 (10 bar), Al-complex **A** (2 mol%), additives (5-10 mol%), MEK (0.4 mL). [b] All reported yields and conversions are based on ^1H NMR ($CDCl_3$) measurements using mesitylene as an internal standard. [c] Yield of the isolated product.

S10. Experimental procedures for the product diversification

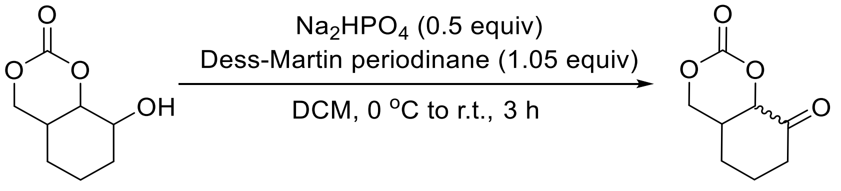

P8
0.2 mmol

P22 75%
exo/*endo* 65:35

In a 10 mL oven-dried glass vial, **P8** (0.2 mmol, 34.4 mg, 1.0 equiv) was dissolved in 1 mL of dry DCM under argon. The solution was cooled to 0 °C, and anhydrous Na_2HPO_4 (15.7 mg, 0.1 mmol, 0.5 equiv) was added. Dess-Martin periodinane (90 mg, 0.21 mmol, 1.05 equiv) was then added at once at 0 °C, and the resulting suspension was warmed to r.t. and stirred for 3 h. The reaction mixture was then quenched by successive addition of saturated aqueous $NaHCO_3$ and saturated aqueous Na_2SO_3 solutions (1.5 mL in both cases), extracted with DCM, dried over Na_2SO_4, filtered, and concentrated under reduced pressure. The residue was purified by silica gel column chromatography (hexane/EA= 10:1) to afford the desired **P22** (25.5 mg, 75%) as a white solid.

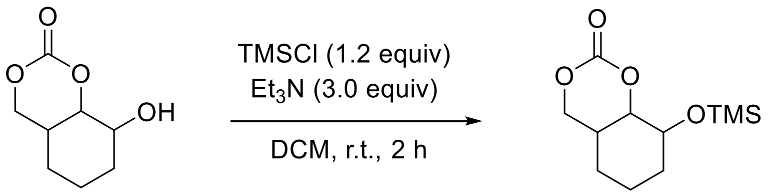

P8
0.2 mmol

P23 91%

In a 10 mL oven-dried glass vial, a mixture of **P8** (34.4 mg, 0.20 mmol, 1.0 equiv) and triethyl amine (84 μL, 0.6 mmol 3.0 equiv) in anhydrous DCM (2 mL) under N_2 was prepared. Subsequently, TMSCl (31.0 μL, 0.24 mmol, 1.2 equiv) was added slowly. After stirring for 2 h at r.t., the mixture was concentrated to dryness and directly purified by column chromatography (hexane/EA = 10:1) to give the product **P23** in 91% yield (44.5 mg) as white solid.

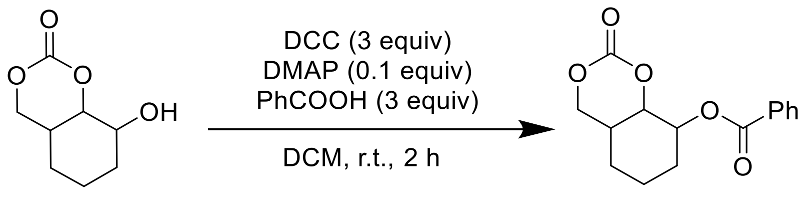

P8
0.2 mmol

P24 74%

To a stirred solution of **P8** (34.4 mg, 0.2 mmol, 1 equiv) in 1 mL of DCM was successively added DMAP (2.4 mg, 0.02 mmol, 0.1 equiv), benzoic acid (73.2 mg, 0.6 mmol, 3 equiv) and DCC (123.7 mg, 0.6 mmol, 3 equiv). After stirring for 2 h at r.t., the mixture was diluted with 2 mL of diethyl ether, and then filtered. The filtrate was concentrated to dryness and purified by column chromatography (eluent: hexane/EA = 4:1-2:1) to give the esterification product **P24** in 74% yield (41.0 mg) as a white solid.

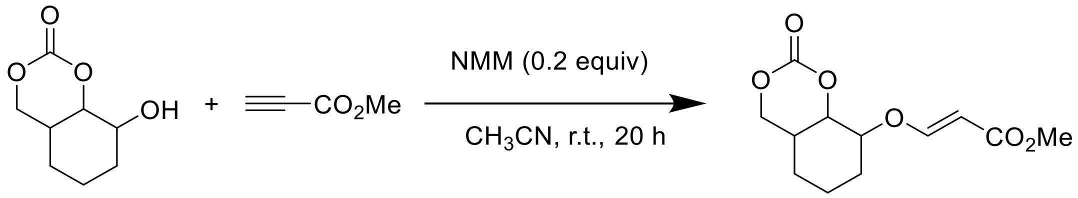

P8
0.2 mmol

P25 76%

To a stirred solution of **P8** (34.4 mg, 0.2 mmol, 1 equiv) in 1 mL of CH_3CN was successively added NMM (4.0 mg, 0.04 mmol, 0.2 equiv) and methyl propiolate (20.2 mg, 0.24 mmol, 1.2 equiv). After stirring for 20 h at r.t., the mixture was concentrated to dryness and directly purified by column chromatography (hexane/EA = 2:1) to give the ether product **P25** in 76% yield (38.9 mg) as a colorless oil.

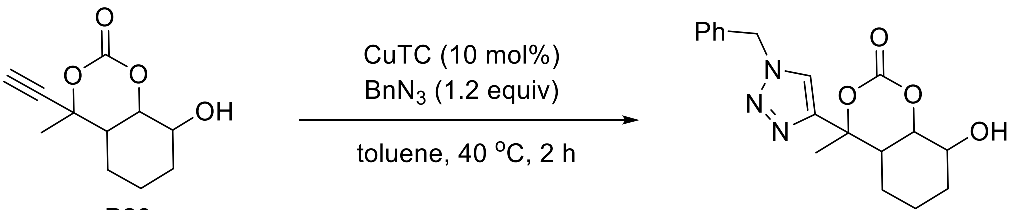

P20 0.2 mmol

P26 85%

In a 10 mL oven-dried glass vial, a mixture of **P20** (43.2 mg, 0.20 mmol, 1.0 equiv) and copper(I) thiophene-2-carboxylate (CuTC) (3.8 mg, 0.02 mmol, 0.1 equiv) in anhydrous toluene (1 mL) was cooled with an ice-water bath. Subsequently, benzyl azide (30.0 μL, 0.24 mmol, 1.2 equiv) was added slowly, and the reaction mixture was heated to 40 °C and stirred for 2 h while monitored by TLC. The reaction mixture was quenched by a saturated aqueous NH_4Cl solution, extracted with DCM (3 × 10 mL), dried over Na_2SO_4, filtered, and concentrated under reduced pressure. The residue was purified by silica gel column chromatography (hexane/EA= 1:1) to afford the desired triazole **P26** (58.4 mg, 85%) as a white solid.

S13. Characterization data for all new and relevant compounds

Cyclic epoxy alcohols

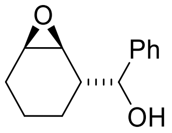

1a (*anti*)

Using methanol as solvent. The product was isolated as a colorless oil, eluent: 10% EA in hexane. Yield: 60%, 367.3 mg. **^{1}H NMR** (500 MHz, $CDCl_3$) δ 7.39 – 7.34 (m, 4H), 7.32 – 7.26 (m, 1H), 4.87 (d, *J* = 5.8 Hz, 1H), 3.18 (dt, *J* = 4.0, 2.0 Hz, 1H), 3.07 (d, *J* = 3.9 Hz, 1H), 2.22 (dt, *J* = 11.3, 5.6 Hz, 1H), 2.11 – 2.04 (m, 1H), 1.89 (s, 1H), 1.68 (dddd, *J* = 14.9, 11.6, 5.3, 2.0 Hz, 1H), 1.45 – 1.37 (m, 2H), 1.34 – 1.23 (m, 1H), 1.19 – 1.11 (m, 1H); **^{13}C NMR** (126 MHz, $CDCl_3$) δ 142.73, 128.62, 127.87, 126.39, 76.53, 54.85, 53.08, 41.92, 24.95, 21.52, 17.13; **HRMS** (ESI+; MeOH): *m/z* calcd. ($C_{13}H_{16}NaO_2$) 227.1043 $(M+Na)^+$: found: 227.1036.

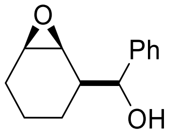

1a (*syn*)

The product was isolated as a white solid, eluent: 10% EA in hexane. Yield: 92%, 563 mg. **^{1}H NMR** (500 MHz, $CDCl_3$) δ 7.47 – 7.43 (m, 2H), 7.41 – 7.36 (m, 2H), 7.33 – 7.29 (m, 1H), 4.89 (dt, *J* = 6.7, 1.1 Hz, 1H), 3.15 (tdd, *J* = 4.1, 1.4, 0.6 Hz, 1H), 2.91 (dd, *J* = 4.1, 2.6 Hz, 1H), 2.45 (d, *J* = 2.2 Hz, 1H), 2.16 (dtd, *J* = 8.0, 6.6, 2.5 Hz, 1H), 1.93 – 1.81 (m, 2H), 1.65 – 1.58 (m, 1H), 1.50 – 1.44 (m, 2H), 1.30 – 1.17 (m, 1H); **^{13}C NMR** (126 MHz, $CDCl_3$) δ 143.03, 128.47, 127.68, 126.51, 76.54, 54.37, 52.48, 42.43, 23.83, 20.44, 19.73; **HRMS** (ESI+; MeOH): *m/z* calcd. ($C_{13}H_{16}NaO_2$) 227.1043 $(M+Na)^+$: found: 227.1038.

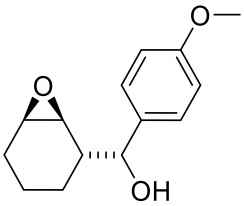

1b

Using dichloromethane as solvent. The product was isolated as a colorless oil, eluent: 30% EA in hexane. Yield: 13%, 91.5 mg. **^{1}H NMR** (400 MHz, $CDCl_3$) δ 7.31 – 7.26 (m, 2H), 6.92 – 6.86 (m, 2H), 4.77 (d, *J* = 6.3 Hz, 1H), 3.80 (s, 3H), 3.19 – 3.11 (m, 1H), 3.04 – 2.95 (m, 1H), 2.18 (dt,

J = 11.6, 6.0 Hz, 1H), 2.06 (ddd, *J* = 15.3, 3.4, 1.9 Hz, 1H), 1.91 (s, 1H), 1.71 – 1.63 (m, 1H), 1.54 – 1.37 (m, 2H), 1.31 (dddd, *J* = 15.0, 6.4, 4.6, 2.2 Hz, 1H), 1.12 (tdd, *J* = 12.5, 11.0, 2.8 Hz, 1H); **^{13}C NMR** (101 MHz, $CDCl_3$) δ 159.30, 134.88, 127.66, 114.00, 76.18, 55.43, 54.68, 53.07, 41.97, 24.95, 21.97, 17.16; **HRMS** (ESI+; MeOH): *m/z* calcd. ($C_{14}H_{18}NaO_3$) 257.1148 $(M+Na)^+$: found: 257.1149.

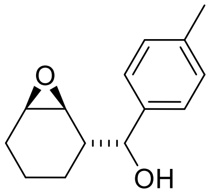

1c

Using dichloromethane as solvent. The product was isolated as a colorless oil, eluent: 30% EA in hexane. Yield: 16%, 104.8 mg. **^{1}H NMR** (500 MHz, $CDCl_3$) δ 7.25 (d, *J* = 8.0 Hz, 2H), 7.17 (d, *J* = 7.8 Hz, 2H), 4.81 (d, *J* = 6.0 Hz, 1H), 3.16 (dt, *J* = 4.1, 2.0 Hz, 1H), 3.04 (d, *J* = 3.9 Hz, 1H), 2.35 (s, 3H), 2.19 (dt, *J* = 11.6, 5.9 Hz, 1H), 1.94 (s, 1H), 1.72 – 1.58 (m, 2H), 1.49 – 1.37 (m, 2H), 1.34 – 1.23 (m, 1H), 1.18 – 1.08 (m, 1H); **^{13}C NMR** (126 MHz, $CDCl_3$) δ 139.76, 137.53, 129.27, 126.35, 76.39, 54.82, 53.08, 41.90, 24.95, 21.71, 21.23, 17.15; **HRMS** (ESI+; MeOH): *m/z* calcd. ($C_{14}H_{18}NaO_2$) 241.1199 $(M+Na)^+$: found: 241.1199.

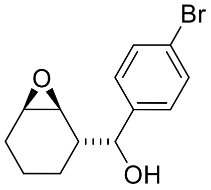

1d

Using dichloromethane as solvent. The product was isolated as a white solid, eluent: 30% EA in hexane. Yield: 15%, 127.5 mg. **^{1}H NMR** (500 MHz, $CDCl_3$) δ 7.50 – 7.45 (m, 2H), 7.24 – 7.20 (m, 2H), 4.82 (d, *J* = 5.4 Hz, 1H), 3.16 (dt, *J* = 4.1, 1.9 Hz, 1H), 3.05 – 3.02 (m, 1H), 2.22 (s, 1H), 2.13 (dt, *J* = 11.2, 5.6 Hz, 1H), 2.05 (dqd, *J* = 14.6, 2.9, 1.4 Hz, 1H), 1.65 (dddd, *J* = 14.9, 11.8, 5.4, 2.0 Hz, 1H), 1.41 – 1.19 (m, 3H), 1.09 (tdd, *J* = 12.8, 11.2, 2.7 Hz, 1H); **^{13}C NMR** (126 MHz, $CDCl_3$) δ 141.73, 131.62, 128.05, 121.54, 75.70, 54.77, 53.10, 41.86, 24.84, 21.21, 17.05; **HRMS** (ESI+; MeOH): *m/z* calcd. ($C_{13}H_{15}BrNaO_2$) 305.1048 $(M+Na)^+$: found: 305.1049.

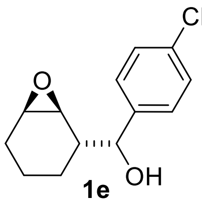

1e

Using dichloromethane as solvent. The product was isolated as a white solid, eluent: 30% EA in hexane. Yield: 13%, 93.1 mg. **^{1}H NMR** (400 MHz, $CDCl_3$) δ 7.35 – 7.28 (m, 4H), 4.87 (d, *J* = 5.5 Hz, 1H), 3.18 (dd, *J* = 3.9, 2.0 Hz, 1H), 3.05 (d, *J* = 3.7 Hz, 1H), 2.17 (dt, *J* = 10.9, 5.5 Hz, 1H), 2.11 – 2.03 (m, 1H), 1.66 (dddd, *J* = 14.9, 11.5, 5.4, 1.9 Hz, 1H), 1.45 – 1.24 (m, 3H), 1.18 – 1.06 (m, 1H); **^{13}C NMR** (101 MHz, $CDCl_3$) δ 141.19, 133.53, 128.74, 127.72, 75.80, 54.73, 53.07, 41.96, 24.89, 21.29, 17.08; **HRMS** (ESI+; MeOH): *m/z* calcd. ($C_{13}H_{15}ClNaO_2$) 261.0653 $(M+Na)^+$: found: 261.0649.

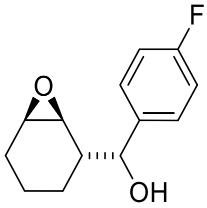

1f

Using dichloromethane as solvent. The product was isolated as a colorless oil, eluent: 30% EA in hexane. Yield: 10%, 66.7 mg. **^{1}H NMR** (500 MHz, $CDCl_3$) δ 7.36 – 7.30 (m, 2H), 7.07 – 7.02 (m, 2H), 4.84 (d, *J* = 5.8 Hz, 1H), 3.16 (dt, *J* = 4.2, 1.9 Hz, 1H), 3.03 (d, *J* = 3.9 Hz, 1H), 2.16 (dt, *J* = 11.2, 5.6 Hz, 1H), 2.09 – 2.00 (m, 2H), 1.66 (dddd, *J* = 14.9, 11.8, 5.3, 2.0 Hz, 1H), 1.45 – 1.37 (m, 2H), 1.33 – 1.23 (m, 1H), 1.16 – 1.07 (m, 1H); **^{13}C NMR** (126 MHz, $CDCl_3$) δ 162.37 (d, *J* = 245.9 Hz), 138.47 (d, *J* = 3.2 Hz), 128.00 (d, *J* = 7.9 Hz), 115.43 (d, *J* = 21.5 Hz), 75.83, 54.69, 53.06, 42.01, 24.90, 21.51, 17.09; **^{19}F NMR** (471 MHz, $CDCl_3$) δ -114.79; **HRMS** (ESI+; MeOH): *m/z* calcd. ($C_{13}H_{15}FNaO_2$) 245.0948 $(M+Na)^+$: found: 245.0940.

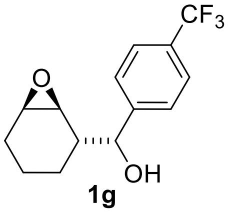

1g

Using dichloromethane as solvent. The product was isolated as a white solid, eluent: 30% EA in hexane. Yield: 25%, 204.2 mg. **^{1}H NMR** (400 MHz, $CDCl_3$) δ 7.65 – 7.59 (m, 2H), 7.48 (dq, *J* = 8.2, 0.9 Hz, 2H), 4.97 (d, *J* = 5.1 Hz, 1H), 3.20 (dd, *J* = 3.8, 2.0 Hz, 1H), 3.10 (dt, *J* = 4.0, 0.7 Hz, 1H), 2.20 (dt, *J* = 11.0, 5.4 Hz, 1H), 2.11 – 2.03 (m, 1H), 1.67 (dddd, *J* = 14.9, 11.5, 5.5, 1.9 Hz, 1H), 1.39 (dtd, *J* = 9.8, 5.2, 4.7, 2.6 Hz, 1H), 1.33 – 1.20 (m, 2H), 1.19 – 1.07 (m, 1H); **^{13}C NMR**

(101 MHz, $CDCl_3$) δ 146.72, 133.70, 130.49, 130.31, 130.17, 129.94, 129.85, 129.53, 128.34, 128.28, 126.61, 125.57, 125.53, 125.49, 125.45, 122.87, 120.17, 75.78, 54.87, 53.12, 41.95, 24.84, 20.94, 17.04; **^{19}F NMR** (376 MHz, $CDCl_3$) δ -62.61; **HRMS** (ESI+; MeOH): *m/z* calcd. ($C_{14}H_{15}F_3NaO_2$) 295.0916 $(M+Na)^+$: found: 295.0916.

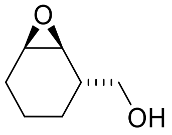

1h

Using methanol as solvent. The product was isolated as a colorless oil, eluent: 30% EA in hexane. Yield: 17%, 65.4 mg. **^{1}H NMR** (500 MHz, $CDCl_3$) δ 3.73 (dd, *J* = 10.7, 5.7 Hz, 1H), 3.64 (dd, *J* = 10.7, 7.0 Hz, 1H), 3.18 (dt, *J* = 4.1, 2.0 Hz, 1H), 3.09 (dd, *J* = 4.0, 1.0 Hz, 1H), 2.13 – 2.00 (m, 2H), 1.69 (dddd, *J* = 15.0, 10.4, 6.3, 2.1 Hz, 2H), 1.60 – 1.53 (m, 1H), 1.43 – 1.35 (m, 2H), 0.95 (dtd, *J* = 13.2, 11.1, 4.3 Hz, 1H); **^{13}C NMR** (126 MHz, $CDCl_3$) δ 65.43, 54.16, 52.83, 37.61, 24.93, 23.97, 17.20. **HRMS** (APCI+; MeOH): *m/z* calcd. ($C_7H_{13}O_2$) 129.0910 $(M+H)^+$: found: 129.0905.

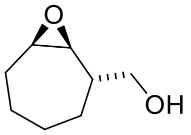

1i

Using dichloromethane as solvent. The product was isolated as a colorless oil, eluent: 30% EA in hexane. Yield: 46%, 196.2 mg. **^{1}H NMR** (400 MHz, $CDCl_3$) δ 3.69 (qd, *J* = 10.5, 6.6 Hz, 2H), 3.04 (ddd, *J* = 7.3, 5.5, 4.5 Hz, 1H), 2.87 (dd, *J* = 7.1, 4.7 Hz, 1H), 2.21 (ddd, *J* = 11.7, 7.5, 5.6 Hz, 1H), 1.86 – 1.72 (m, 2H), 1.71 – 1.64 (m, 1H), 1.59 (dq, *J* = 5.6, 3.1, 2.2 Hz, 2H), 1.46 – 1.33 (m, 4H); **^{13}C NMR** (101 MHz, $CDCl_3$) δ 66.18, 57.81, 54.76, 43.90, 30.16, 29.92, 28.19, 24.59; **HRMS** (APCI+; MeOH): *m/z* calcd. ($C_8H_{13}O$) 125.0961 $(M-OH)^+$: found: 125.0955.

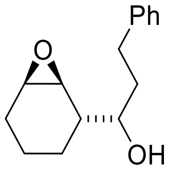

1j

Using methanol as solvent. The product was isolated as a colorless oil, eluent: 15% EA in hexane. Yield: 27%, 188.2 mg, *anti*:*syn* = 3:1. Only the data for the *anti* isomer is reported here. **^{1}H NMR** (500 MHz, $CDCl_3$) δ 7.29 (dt, *J* = 8.8, 6.8 Hz, 3H), 7.24 – 7.16 (m, 4H), 3.80 (dt, *J* = 8.4, 4.2 Hz, 1H), 3.20 (ddd, *J* = 10.5, 4.1, 1.6 Hz, 1H), 3.06 (dd, *J* = 4.0, 1.1 Hz, 1H), 2.83 (ddd, *J* = 13.6, 9.4, 5.9 Hz, 1H), 2.69 (tdd, *J* = 13.7, 9.5, 6.7 Hz, 1H), 2.09 (ddq, *J* = 14.8, 4.2, 2.2 Hz, 1H), 1.98 – 1.76 (m, 6H), 1.69 – 1.62 (m, 1H), 1.58 – 1.48 (m, 2H), 1.44 (dtt, *J* = 12.9, 5.2, 2.9 Hz, 1H), 1.40

– 1.23 (m, 2H), 1.08 (qd, J = 12.9, 3.1 Hz, 1H); ^{13}**C NMR** (126 MHz, $CDCl_3$) δ 142.31, 141.79, 128.61, 128.57, 128.53, 128.50, 126.12, 125.91, 74.26, 73.50, 55.30, 53.36, 53.01, 52.48, 40.36, 40.16, 37.02, 36.22, 32.63, 32.30, 24.99, 23.79, 22.41, 20.87, 19.86, 17.14; **HRMS** (ESI+; MeOH): *m/z* calcd. ($C_{15}H_{20}NaO_2$) 255.1356 $(M+Na)^+$: found: 255.1347.

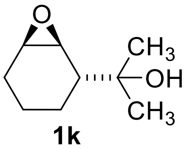

1k

Using methanol as solvent. The product was isolated as a colorless oil, eluent: 30% EA in hexane. Yield: 46%, 215.6 mg. ^{1}**H NMR** (500 MHz, $CDCl_3$) δ 3.23 (dd, J = 4.0, 1.3 Hz, 1H), 3.18 (q, J = 2.0 Hz, 1H), 2.11 (ddq, J = 14.8, 3.8, 1.9 Hz, 1H), 1.79 (dd, J = 12.2, 6.0 Hz, 1H), 1.67 – 1.61 (m, 2H), 1.48 – 1.41 (m, 1H), 1.35 (ddd, J = 12.9, 4.6, 2.6 Hz, 2H), 1.29 (s, 3H), 1.25 (s, 3H), 0.93 (qd, J = 12.9, 3.2 Hz, 1H); ^{13}**C NMR** (126 MHz, $CDCl_3$) δ 72.55, 53.54, 53.07, 46.17, 28.70, 26.34, 25.02, 23.33, 17.54; **HRMS** (ESI+; MeOH): *m/z* calcd. ($C_9H_{16}NaO_2$) 179.1043 $(M+Na)^+$: found: 179.1042.

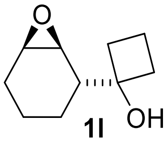

1l

Using dichloromethane as solvent. The product was isolated as a colorless oil, eluent: 20% EA in hexane. Yield: 21%, 106.0 mg. ^{1}**H NMR** (500 MHz, $CDCl_3$) δ 3.20 (dt, J = 4.1, 1.9 Hz, 1H), 3.07 (dd, J = 4.0, 1.3 Hz, 1H), 2.38 (dddd, J = 12.5, 9.6, 4.3, 3.5 Hz, 1H), 2.22 (dddd, J = 13.1, 8.4, 4.4, 3.5 Hz, 1H), 2.14 – 2.08 (m, 2H), 1.99 (dd, J = 12.2, 6.0 Hz, 1H), 1.96 – 1.90 (m, 1H), 1.89 – 1.80 (m, 1H), 1.72 – 1.57 (m, 5H), 1.49 (dtt, J = 12.7, 5.1, 2.6 Hz, 1H), 1.35 (qdd, J = 13.1, 4.6, 2.7 Hz, 1H), 1.12 – 1.02 (m, 1H).^{13}**C NMR** (126 MHz, $CDCl_3$) δ 77.08, 53.57, 52.98, 42.21, 35.69, 34.78, 24.98, 20.82, 17.51, 12.20; **HRMS** (ESI+; MeOH): *m/z* calcd. ($C_{10}H_{16}NaO_2$) 191.1043 $(M+Na)^+$: found: 191.1037.

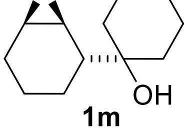

1m

Using methanol as solvent. The product was isolated as a colorless oil, eluent: 10% EA in hexane. Yield: 60%, 353.3 mg. ^{1}**H NMR** (500 MHz, $CDCl_3$) δ 3.24 (dd, J = 4.1, 1.3 Hz, 1H), 3.15 (dt, J = 4.0, 1.9 Hz, 1H), 2.09 (ddt, J = 14.8, 4.2, 1.9 Hz, 1H), 1.77 (dd, J = 12.3, 6.0 Hz, 1H), 1.70 – 1.48 (m, 12H), 1.45 (ddp, J = 12.7, 5.1, 2.5 Hz, 1H), 1.39 – 1.28 (m, 2H), 0.99 (qd, J = 12.8, 3.0 Hz, 1H); ^{13}**C NMR** (126 MHz, $CDCl_3$) δ 73.11, 53.56, 53.09, 44.73, 35.55, 34.22, 25.79, 25.03,

22.03, 22.02, 21.94, 17.62; **HRMS** (ESI+; MeOH): *m/z* calcd. ($C_{12}H_{20}NaO_2$) 219.1356 $(M+Na)^+$: found: 219.1348.

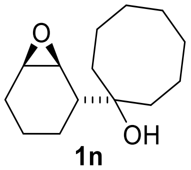

1n

Using dichloromethane as solvent. The product was isolated as a colorless oil, eluent: 15% EA in hexane. Yield: 28%, 188.5 mg. **^{1}H NMR** (500 MHz, $CDCl_3$) δ 3.28 (dd, *J* = 4.1, 1.3 Hz, 1H), 3.18 – 3.14 (m, 1H), 2.14 – 2.07 (m, 1H), 1.99 – 1.92 (m, 1H), 1.85 – 1.75 (m, 3H), 1.71 – 1.59 (m, 9H), 1.55 – 1.28 (m, 7H), 1.05 (dtd, *J* = 13.3, 12.5, 2.9 Hz, 1H); **^{13}C NMR** (126 MHz, $CDCl_3$) δ 76.24, 53.78, 53.15, 44.30, 34.98, 34.44, 28.31, 25.15, 24.98, 22.30, 22.29, 22.21, 17.88; **HRMS** (ESI+; MeOH): *m/z* calcd. ($C_{14}H_{24}NaO_2$) 247.1669 $(M+Na)^+$: found: 247.1670.

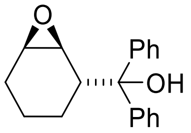

1o

Using methanol as solvent. The product was isolated as a white solid, eluent: 10% EA in hexane. Yield: 30%, 252.3 mg. **^{1}H NMR** (500 MHz, $CDCl_3$) δ 7.64 – 7.59 (m, 2H), 7.42 – 7.39 (m, 2H), 7.38 – 7.33 (m, 2H), 7.29 (dd, *J* = 8.4, 7.1 Hz, 2H), 7.25 – 7.21 (m, 1H), 7.20 – 7.14 (m, 1H), 3.17 – 3.14 (m, 2H), 2.98 (dd, *J* = 12.2, 5.6 Hz, 1H), 2.12 (ddq, *J* = 15.0, 3.7, 1.6 Hz, 1H), 1.67 (dddd, *J* = 15.0, 12.3, 5.9, 1.6 Hz, 1H), 1.46 – 1.31 (m, 2H), 1.26 – 1.19 (m, 1H), 1.08 (qd, *J* = 12.8, 3.5 Hz, 1H); **^{13}C NMR** (126 MHz, $CDCl_3$) δ 145.94, 145.85, 128.76, 128.36, 127.14, 126.70, 126.15, 125.47, 80.39, 54.53, 53.58, 43.49, 24.93, 22.47, 17.72; **HRMS** (ESI+; MeOH): *m/z* calcd. ($C_{19}H_{20}NaO_2$) 303.1356 (M+Na)+: found: 303.1346.

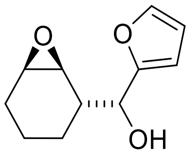

1p

Using dichloromethane as solvent. The product was isolated as colorless oil, eluent: 30% Et_2O in hexane. Yield: 21%, 122.4 mg. **^{1}H NMR** (500 MHz, $CDCl_3$) δ 7.39 (dt, *J* = 1.9, 1.0 Hz, 1H), 6.35 (td, *J* = 2.4, 1.9, 1.2 Hz, 1H), 6.30 (dq, *J* = 3.2, 0.8 Hz, 1H), 4.81 – 4.75 (m, 1H), 3.16 (q, *J* = 2.1 Hz, 1H), 3.08 (dd, *J* = 3.9, 1.1 Hz, 1H), 2.36 (ddd, *J* = 12.4, 7.1, 5.3 Hz, 1H), 2.11 – 2.05 (m, 1H), 1.70 – 1.55 (m, 2H), 1.43 – 1.31 (m, 2H), 1.12 – 1.02 (m, 1H); **^{13}C NMR** (126 MHz, $CDCl_3$) δ

155.17, 142.32, 110.38, 107.27, 70.18, 70.16, 54.05, 54.04, 52.98, 52.97, 40.00, 24.87, 22.68, 17.07; **HRMS** (ESI+; MeOH): *m/z* calcd. ($C_{11}H_{14}NaO_3$) 217.0835 $(M+Na)^+$: found: 217.0830.

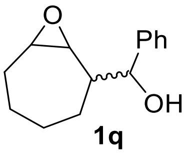

1q

Using dichloromethane as solvent. The product was isolated as colorless oil, eluent: 30% EA in hexane. Yield: 61%, 399.5 mg, *anti*:*syn* = 1:1. The NMR data for both *anti* and *syn* isomers are reported here. **^{1}H NMR** (500 MHz, $CDCl_3$) δ 7.45 – 7.36 (m, 4H), 7.31 (dddd, *J* = 8.0, 4.3, 3.1, 1.7 Hz, 0.97H), 4.91 (dd, *J* = 5.6, 1.9 Hz, 0.52H), 4.79 (dd, *J* = 6.5, 2.9 Hz, 0.48H), 3.19 (dd, *J* = 4.8, 1.1 Hz, 0.52H), 3.10 (t, *J* = 5.2 Hz, 0.52H), 2.99 (tdd, *J* = 5.6, 4.7, 1.6 Hz, 0.48H), 2.89 (dd, *J* = 7.4, 4.8 Hz, 0.48H), 2.30 (dtdd, *J* = 15.0, 5.3, 2.7, 1.2 Hz, 0.55H), 2.25 – 2.16 (m, 1.54H), 2.05 (d, *J* = 3.7 Hz, 0.42H), 2.00 – 1.84 (m, 1.51H), 1.81 – 1.71 (m, 1.16H), 1.70 – 1.65 (m, 0.81H), 1.56 (d, *J* = 14.1 Hz, 0.59H), 1.47 – 1.26 (m, 2.63H), 1.20 (dtd, *J* = 14.0, 12.1, 2.1 Hz, 0.57H), 0.83 (dtt, *J* = 14.3, 12.2, 2.2 Hz, 0.57H); **^{13}C NMR** (126 MHz, $CDCl_3$) δ 142.92, 142.89, 128.57, 128.49, 127.89, 127.74, 126.62, 78.28, 76.34, 58.19, 56.93, 55.21, 55.05, 48.24, 46.87, 30.25, 29.86, 28.97, 28.35, 26.86, 25.06, 24.66, 24.32; **HRMS** (ESI+; MeOH): *m/z* calcd. ($C_{14}H_{18}NaO_2$) 241.1199 $(M+Na)^+$: found: 241.1197.

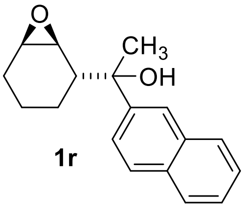

1r

Using dichloromethane as solvent. The product was isolated as a colorless oil, eluent: 30% EA in hexane. Yield: 16%, 128.8 mg. **^{1}H NMR** (400 MHz, $CDCl_3$) δ 7.90 (d, *J* = 1.9 Hz, 1H), 7.88 – 7.80 (m, 3H), 7.54 – 7.42 (m, 3H), 3.42 (dd, *J* = 4.1, 1.3 Hz, 1H), 3.18 (dd, *J* = 3.9, 2.0 Hz, 1H), 2.30 – 2.23 (m, 1H), 2.10 – 2.01 (m, 1H), 1.96 (s, 1H), 1.80 (s, 3H), 1.61 – 1.53 (m, 1H), 1.34 – 1.11 (m, 3H), 0.92 (dq, *J* = 12.8, 3.4 Hz, 1H); **^{13}C NMR** (101 MHz, $CDCl_3$) δ 144.42, 133.21, 132.40, 128.27, 128.17, 127.62, 126.33, 126.00, 123.74, 123.5076.45, 53.68, 53.18, 45.60, 28.56, 24.84, 23.17, 17.67; **HRMS** (ESI+; MeOH): *m/z* calcd. ($C_{18}H_{20}NaO_2$) 291.1356 $(M+Na)^+$: found: 291.1355.

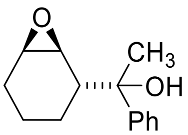

1s

Using methanol as solvent. The product was isolated as a colorless oil, eluent: 10% EA in hexane. Yield: 51%, 334.0 mg. **^{1}H NMR** (400 MHz, $CDCl_3$) δ 7.43 – 7.38 (m, 2H), 7.38 – 7.31 (m, 2H), 7.26 – 7.22 (m, 1H), 3.35 (dd, *J* = 4.1, 1.3 Hz, 1H), 3.16 (dt, *J* = 3.5, 1.8 Hz, 1H), 2.15 (dd, *J* = 12.4, 5.7 Hz, 1H), 2.10 – 2.01 (m, 1H), 1.79 (s, 1H), 1.72 (s, 3H), 1.60 – 1.51 (m, 1H), 1.36 – 1.12 (m, 3H), 0.88 (dq, *J* = 13.0, 3.2 Hz, 1H); **^{13}C NMR** (101 MHz, $CDCl_3$) δ 146.94, 128.36, 126.90, 125.10, 76.26, 53.66, 53.14, 45.86, 28.52, 24.86, 23.07, 17.69; **HRMS** (ESI+; MeOH): *m/z* calcd. ($C_{14}H_{18}NaO_2$) 241.1199 $(M+Na)^+$: found: 241.1187.

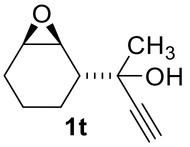

1t

Using dichloromethane as solvent. The product was isolated as a white solid, eluent: 10% EA in hexane. Yield: 70%, 349.1 mg, *anti*:*syn* = 10:1. Only the NMR data for the *anti* isomer is reported here. **^{1}H NMR** (400 MHz, $CDCl_3$) δ 3.37 (dd, J = 4.0, 1.3 Hz, 1H), 3.20 (dt, J = 4.1, 1.9 Hz, 1H), 2.47 (s, 1H), 2.15 – 2.07 (m, 2H), 1.97 (dd, J = 12.1, 6.2 Hz, 1H), 1.73 – 1.63 (m, 2H), 1.55 (s, 3H), 1.41 (dddd, J = 25.7, 12.8, 5.3, 2.7 Hz, 2H), 1.17 – 1.05 (m, 1H); **^{13}C NMR** (101 MHz, $CDCl_3$) δ 86.27, 72.77, 69.97, 53.25, 53.01, 45.77, 28.70, 24.81, 23.62, 17.20; **HRMS** (ESI+; MeOH): *m/z* calcd. ($C_{10}H_{14}NaO_2$) 189.0886 $(M+H)^+$: found: 189.0882.

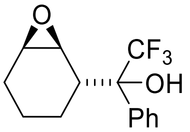

1u

Using dichloromethane as solvent. The product was isolated as a colorless oil, eluent: 13% EA in hexane. Yield: 31%, 235.21 mg, *dr* >20:1 **^{1}H NMR** (400 MHz, $CDCl_3$) δ 7.56 – 7.50 (m, 2H), 7.43 – 7.36 (m, 3H), 3.53 (d, *J* = 4.0 Hz, 1H), 3.13 (dt, *J* = 4.0, 2.0 Hz, 1H), 3.12 – 3.07 (m, 1H), 2.59 (dd, *J* = 12.2, 5.5 Hz, 1H), 2.06 (ddd, *J* = 15.0, 3.9, 2.1 Hz, 1H), 1.63 – 1.53 (m, 1H), 1.42 – 1.20 (m, 3H), 0.97 (qd, *J* = 12.8, 3.4 Hz, 1H); **^{19}F NMR** (376 MHz, $CDCl_3$) δ -73.12; **^{13}C NMR** (101 MHz, $CDCl_3$) δ 136.94, 130.42, 128.64, 128.58, 127.56, 125.92 (q, *J* = 2.0 Hz), 124.70, 121.84, 79.37 (q, *J* = 27.3 Hz), 53.02, 52.25 (q, *J* = 3.5 Hz), 41.56, 24.42, 22.78, 17.41; **HRMS** (APCI+; MeOH): *m/z* calcd. ($C_{14}H_{14}F_3O$) 255.0991 $(M-OH)^+$: found: 255.0986.

6-Membered cyclic carbonates

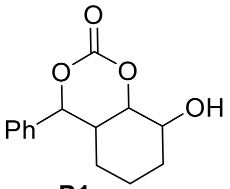

P1

The product was isolated as a white solid, eluent: 30% EA in hexane. Yield: 85%, 21.1 mg. **^{1}H NMR** (500 MHz, $CDCl_3$) δ 7.42 – 7.37 (m, 2H), 7.36 – 7.28 (m, 3H), 5.63 (d, *J* = 3.0 Hz, 1H), 4.71 (t, *J* = 3.1 Hz, 1H), 4.19 (q, *J* = 3.0 Hz, 1H), 2.51 – 2.43 (m, 1H), 1.83 – 1.66 (m, 3H), 1.63 – 1.46 (m, 2H), 1.28 – 1.17 (m, 1H), 1.14 (dt, *J* = 13.8, 4.0 Hz, 1H); **^{13}C NMR** (126 MHz, $CDCl_3$) δ 148.75, 135.96, 128.68, 128.37, 125.22, 82.99, 79.33, 66.64, 33.45, 27.25, 18.22, 17.54; **IR** (neat, C=O, cm^{-1}): *v* = 1731; **HRMS** (ESI+; MeOH): *m/z* calcd. ($C_{14}H_{16}NaO_4$) 217.0941 $(M+Na)^+$: found: 217.0938.

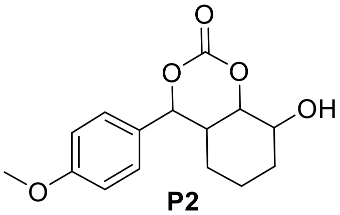

P2

The product was isolated as a white solid, eluent: 70% EA in hexane. Yield: 78%, 21.7 mg. **^{1}H NMR** (500 MHz, $CDCl_3$) δ 7.24 – 7.19 (m, 2H), 6.93 – 6.89 (m, 2H), 5.58 (d, *J* = 3.1 Hz, 1H), 4.68 (t, *J* = 3.0 Hz, 1H), 4.17 (q, *J* = 3.0 Hz, 1H), 3.81 (s, 3H), 2.41 (ddt, *J* = 11.8, 5.8, 2.9 Hz, 1H), 2.08 (s, 1H), 1.80 – 1.66 (m, 2H), 1.62 – 1.48 (m, 2H), 1.26 – 1.15 (m, 2H); **^{13}C NMR** (126 MHz, $CDCl_3$) δ 159.60, 148.90, 127.98, 126.58, 114.05, 82.98, 79.36, 77.41, 77.16, 76.91, 66.63, 55.45, 33.60, 27.27, 18.23, 17.58; **IR** (neat, C=O, cm^{-1}): *v* = 1728; **HRMS** (ESI+; MeOH): *m/z* calcd. ($C_{15}H_{18}NaO_5$) 301.1046 $(M+Na)^+$: found: 301.1051.

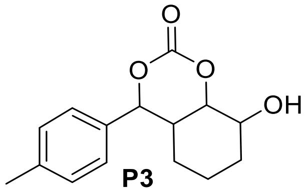

P3

The product was isolated as a white solid, eluent: 50% EA in hexane. Yield: 85%, 22.3 mg. **^{1}H NMR** (500 MHz, $CDCl_3$) δ 7.19 (s, 4H), 5.60 (d, *J* = 3.1 Hz, 1H), 4.69 (t, *J* = 3.1 Hz, 1H), 4.18 (q, *J* = 3.0 Hz, 1H), 2.43 (ddt, *J* = 12.7, 5.5, 2.9 Hz, 1H), 2.35 (s, 3H), 1.91 (s, 1H), 1.82 – 1.69 (m, 2H), 1.62 – 1.47 (m, 2H), 1.27 – 1.12 (m, 2H); **^{13}C NMR** (126 MHz, $CDCl_3$) δ 148.80, 138.16, 132.99, 129.33, 125.19, 83.05, 79.32, 66.70, 33.53, 27.30, 21.26, 18.23, 17.57; **IR** (neat, C=O,

cm^{-1}): v = 1734; **HRMS** (ESI+; MeOH): *m/z* calcd. ($C_{15}H_{18}NaO_4$) 285.1097 $(M+Na)^+$: found: 285.1102.

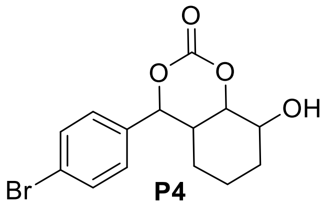

P4

The product was isolated as a white solid, eluent: 50% EA in hexane. Yield: 92%, 30.1 mg. **^{1}H NMR** (500 MHz, $CDCl_3$) δ 7.56 – 7.50 (m, 2H), 7.20 (d, *J* = 8.3 Hz, 2H), 5.59 (d, *J* = 3.1 Hz, 1H), 4.69 (t, *J* = 3.1 Hz, 1H), 4.19 (q, *J* = 3.1 Hz, 1H), 2.48 – 2.40 (m, 1H), 1.81 – 1.67 (m, 3H), 1.62 – 1.50 (m, 2H), 1.23 – 1.07 (m, 2H); **^{13}C NMR** (126 MHz, $CDCl_3$) δ 148.28, 135.06, 131.92, 126.95, 122.40, 82.33, 79.17, 66.63, 33.30, 27.25, 18.17, 17.48; **IR** (neat, C=O, cm^{-1}): v = 1733; **HRMS** (ESI+; MeOH): *m/z* calcd. ($C_{14}H_{15}BrNaO_4$) 349.0046 $(M+Na)^+$: found: 349.0046.

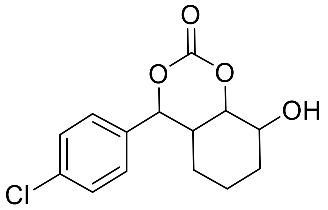

P5

The product was isolated as a white solid, eluent: 50% EA in hexane. Yield: 96%, 27.1 mg. **^{1}H NMR** (500 MHz, $CDCl_3$) δ 7.40 – 7.33 (m, 2H), 7.27 – 7.24 (m, 2H), 5.61 (d, *J* = 3.1 Hz, 1H), 4.69 (t, *J* = 3.2 Hz, 1H), 4.19 (s, 1H), 2.44 (ddt, *J* = 12.9, 5.6, 2.9 Hz, 1H), 1.82 – 1.67 (m, 3H), 1.63 – 1.49 (m, 3H), 1.24 – 1.16 (m, 1H), 1.12 (dd, *J* = 13.7, 4.0 Hz, 1H); **^{13}C NMR** (126 MHz, $CDCl_3$) δ 148.28, 134.52, 134.32, 128.97, 126.65, 82.32, 79.17, 66.65, 33.36, 27.27, 18.18, 17.49; **IR** (neat, C=O, cm^{-1}): v = 1733; **HRMS** (ESI+; MeOH): *m/z* calcd. ($C_{14}H_{15}ClNaO_4$) 305.0551 $(M+Na)^+$: found: 305.0553.

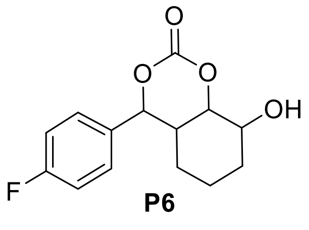

P6

The product was isolated as a white solid, eluent: 50% EA in hexane. Yield: 89%, 23.7 mg. **^{1}H NMR** (500 MHz, $CDCl_3$) δ 7.31 – 7.26 (m, 2H), 7.11 – 7.05 (m, 2H), 5.61 (d, *J* = 3.1 Hz, 1H), 4.70 (t, *J* = 3.1 Hz, 1H), 4.19 (s, 1H), 2.44 (ddt, *J* = 12.9, 4.8, 2.9 Hz, 1H), 1.95 (s, 1H), 1.81 – 1.68 (m, 2H), 1.55 (ddt, *J* = 27.7, 10.6, 4.1 Hz, 2H), 1.21 (qd, *J* = 13.0, 4.2 Hz, 1H), 1.13 (dd, *J* =

13.6, 4.1 Hz, 1H); **^{13}C NMR** (126 MHz, $CDCl_3$) δ 162.62 (d, *J* = 247.3 Hz), 148.49, 131.76 (d, *J* = 3.2 Hz), 127.05 (d, *J* = 8.2 Hz), 115.73 (d, *J* = 21.7 Hz), 82.48, 79.25, 66.61, 33.46, 27.25, 18.17, 17.50; **^{19}F NMR** (471 MHz, $CDCl_3$) δ -113.54; **IR** (neat, C=O, cm^{-1}): *ν* = 1735; **HRMS** (ESI+; MeOH): *m/z* calcd. ($C_{14}H_{15}FNaO_4$) 289.0847 $(M+Na)^+$: found: 289.0852.

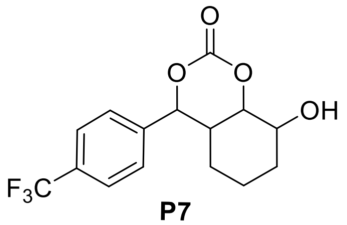

P7

The product was isolated as a white solid, eluent: 50% EA in hexane. Yield: 87%, 27.5 mg. **^{1}H NMR** (500 MHz, $CDCl_3$) δ 7.71 – 7.62 (m, 2H), 7.49 – 7.43 (m, 2H), 5.69 (d, *J* = 3.1 Hz, 1H), 4.73 (t, *J* = 3.0 Hz, 1H), 4.21 (d, *J* = 3.0 Hz, 1H), 2.51 (ddt, *J* = 13.1, 4.6, 2.9 Hz, 1H), 1.83 (s, 1H), 1.82 – 1.68 (m, 2H), 1.64 – 1.51 (m, 2H), 1.22 (qd, *J* = 13.2, 4.4 Hz, 1H), 1.07 (dd, *J* = 13.8, 4.0 Hz, 1H); **^{13}C NMR** (126 MHz, $CDCl_3$) δ 148.12, 140.00, 139.99, 131.15, 130.89, 130.63, 130.37, 127.24, 125.83, 125.80, 125.77, 125.74, 125.65, 125.08, 122.91, 120.75, 82.20, 79.17, 66.61, 33.28, 27.22, 18.20, 17.44; **^{19}F NMR** (471 MHz, $CDCl_3$) δ -62.67; **IR** (neat, C=O, cm^{-1}): *ν* = 1737; **HRMS** (ESI+; MeOH): *m/z* calcd. ($C_{15}H_{15}F_3NaO_4$) 339.0815 $(M+Na)^+$: found: 339.0812.

P8

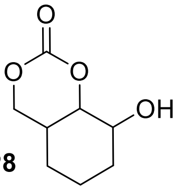

The product was isolated as a white solid, eluent: 70% EA in hexane. Yield: 91%, 15.7 mg. **^{1}H NMR** (500 MHz, $CDCl_3$) δ 4.48 – 4.39 (m, 2H), 4.24 (dd, *J* = 11.0, 3.9 Hz, 1H), 4.07 (td, *J* = 4.8, 3.1 Hz, 1H), 2.39 – 2.33 (m, 1H), 2.19 (s, 1H), 1.87 – 1.79 (m, 1H), 1.78 – 1.53 (m, 6H); **^{13}C NMR** (126 MHz, $CDCl_3$) δ 148.37, 79.85, 72.13, 67.26, 28.37, 28.00, 23.13, 18.19; **IR** (neat, C=O, cm^{-1}): *ν* = 1716 cm^{-1}; **HRMS** (ESI+; MeOH): *m/z* calcd. ($C_8H_{12}NaO_4$) 195.0628 $(M+Na)^+$: found: 195.0621.

P9

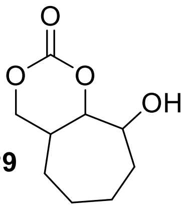

The product was isolated as a white solid, eluent: 50% EA in hexane. Yield: 57%, 10.6 mg. **^{1}H NMR** (500 MHz, $CDCl_3$) δ 4.57 (ddd, *J* = 7.2, 6.0, 0.9 Hz, 1H), 4.41 (dd, *J* = 11.0, 3.6 Hz, 1H),

4.13 (dd, J = 11.0, 3.6 Hz, 1H), 3.88 (ddd, J = 10.5, 7.4, 1.4 Hz, 1H), 2.74 (s, 1H), 2.28 – 2.21 (m, 1H), 1.98 – 1.85 (m, 3H), 1.72 – 1.60 (m, 3H), 1.52 – 1.43 (m, 1H), 1.40 – 1.31 (m, 1H); **^{13}C NMR** (126 MHz, $CDCl_3$) δ 149.53, 88.40, 75.63, 72.57, 35.09, 32.05, 29.14, 26.87, 26.50; **IR** (neat, C=O, cm^{-1}): v = 1730; **HRMS** (ESI+; MeOH): *m/z* calcd. ($C_9H_{14}NaO_4$) 209.0748 $(M+Na)^+$: found: 209.0748.

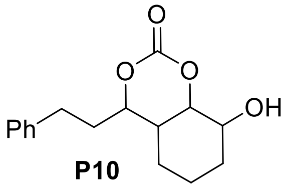

P10

The product was isolated as a white solid, eluent: 50% EA in hexane. Yield: 73%, 20.2 mg. **^{1}H NMR** (500 MHz, $CDCl_3$) δ 7.30 (dd, J = 8.2, 6.9 Hz, 2H), 7.24 – 7.17 (m, 3H), 4.43 – 4.38 (m, 2H), 4.11 – 4.07 (m, 1H), 2.86 (ddd, J = 14.3, 9.3, 5.4 Hz, 1H), 2.71 (dt, J = 13.9, 8.1 Hz, 1H), 2.16 (ddt, J = 10.1, 5.3, 3.2 Hz, 1H), 2.08 (dddd, J = 13.9, 9.7, 8.5, 5.4 Hz, 1H), 1.86 – 1.79 (m, 1H), 1.77 – 1.57 (m, 6H), 1.35 – 1.29 (m, 1H); **^{13}C NMR** (126 MHz, $CDCl_3$) δ 149.02, 140.52, 128.76, 128.59, 126.47, 81.72, 79.35, 66.43, 33.28, 31.17, 30.91, 27.16, 18.16, 17.48; **IR** (neat, C=O, cm^{-1}): v = 1719; **HRMS** (ESI+; MeOH): *m/z* calcd. ($C_{16}H_{20}NaO_4$) 299.1254 $(M+Na)^+$: found: 299.1249.

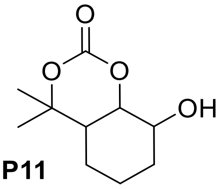

P11

The product was isolated as a white solid, eluent: 50% EA in hexane. Yield: 81%, 16.2 mg. **^{1}H NMR** (500 MHz, $CDCl_3$) δ 4.67 (t, J = 3.2 Hz, 1H), 4.17 – 4.10 (m, 1H), 2.21 (s, 1H), 1.98 (ddd, J = 13.0, 4.7, 2.8 Hz, 1H), 1.79 – 1.71 (m, 2H), 1.70 – 1.57 (m, 3H), 1.51 (s, 3H), 1.38 (s, 3H), 1.32 – 1.23 (m, 1H); **^{13}C NMR** (126 MHz, $CDCl_3$) δ 149.35, 84.47, 75.67, 66.44, 35.36, 27.94, 26.87, 25.87, 20.75, 17.81; **IR** (neat, C=O, cm^{-1}): v = 1714; **HRMS** (ESI+; MeOH): *m/z* calcd. ($C_{10}H_{16}NaO_4$) 223.0941 $(M+Na)^+$: found: 223.0938.

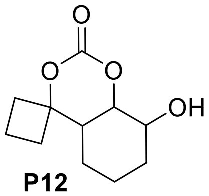

P12

The product was isolated as a white solid, eluent: 50% EA in hexane. Yield: 75%, 15.9 mg. **^{1}H NMR** (500 MHz, $CDCl_3$) δ 4.49 (t, J = 3.1 Hz, 1H), 4.12 (q, J = 2.8 Hz, 1H), 2.43 (ddt, J = 10.1,

8.2, 5.7 Hz, 1H), 2.30 (td, *J* = 7.9, 7.3, 1.9 Hz, 2H), 2.19 (dddd, *J* = 15.6, 8.9, 6.6, 3.5 Hz, 2H), 2.00 – 1.90 (m, 1H), 1.77 – 1.57 (m, 6H), 1.22 – 1.10 (m, 1H); **^{13}C NMR** (126 MHz, $CDCl_3$) δ 148.84, 85.91, 75.01, 66.17, 34.98, 33.36, 31.06, 26.97, 18.91, 17.65, 12.25; **IR** (neat, C=O, cm^{-1}): *v* = 1717; **HRMS** (ESI+; MeOH): *m/z* calcd. ($C_{11}H_{16}NaO_4$) 235.0941 $(M+Na)^+$: found: 235.0933.

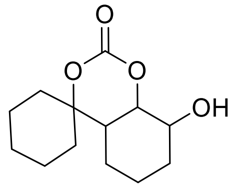

P13

The product was isolated as a white solid, eluent: 30% EA in hexane. Yield: 86%, 20.7 mg. **^{1}H NMR** (500 MHz, $CDCl_3$) δ 4.64 (t, *J* = 3.3 Hz, 1H), 4.12 (d, *J* = 3.5 Hz, 1H), 2.10 (ddd, *J* = 13.1, 4.5, 2.8 Hz, 2H), 1.96 (dt, *J* = 12.3, 4.0 Hz, 1H), 1.81 – 1.65 (m, 8H), 1.60 – 1.50 (m, 4H), 1.48 – 1.35 (m, 2H), 1.31 – 1.21 (m, 1H). **^{13}C NMR** (126 MHz, $CDCl_3$) δ 149.46, 85.56, 75.20, 66.48, 35.80, 33.82, 27.01, 25.10, 22.10, 21.59, 19.85, 17.89; **IR** (neat, C=O, cm^{-1}): *v* = 1719; **HRMS** (ESI+; MeOH): *m/z* calcd. ($C_{13}H_{20}NaO_4$) 263.1254 (M+Na)+: found: 263.1247.

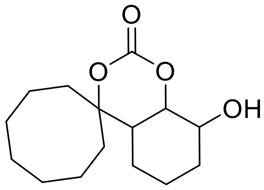

P14

The product was isolated as a white solid, eluent: 50% EA in hexane. Yield: 44%, 12.6 mg. **^{1}H NMR** (500 MHz, $CDCl_3$) δ 4.65 (t, *J* = 3.3 Hz, 1H), 4.13 (q, *J* = 2.8 Hz, 1H), 2.23 (ddd, *J* = 15.2, 10.2, 2.6 Hz, 1H), 2.14 – 2.00 (m, 2H), 1.78 – 1.71 (m, 6H), 1.69 – 1.51 (m, 9H), 1.44 – 1.29 (m, 4H); **^{13}C NMR** (126 MHz, $CDCl_3$) δ 149.27, 89.34, 75.76, 66.66, 33.95, 32.75, 31.90, 28.11, 27.94, 27.10, 25.41, 21.85, 21.62, 20.56, 17.93; **IR** (neat, C=O, cm^{-1}): *v* = 1718; **HRMS** (ESI+; MeOH): *m/z* calcd. ($C_{15}H_{24}NaO_4$) 291.1567 $(M+Na)^+$: found: 291.1562.

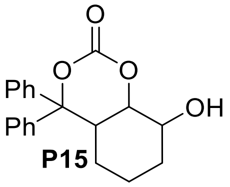

P15

The product was isolated as a white solid, eluent: 25% EA in hexane. Yield: 39%, 12.7 mg. **^{1}H NMR** (400 MHz, DMSO) δ 7.62 – 7.55 (m, 4H), 7.43 (t, *J* = 7.8 Hz, 2H), 7.39 – 7.28 (m, 3H), 7.27 – 7.22 (m, 1H), 5.25 (d, *J* = 3.8 Hz, 1H), 4.22 (t, *J* = 3.2 Hz, 1H), 3.78 (t, *J* = 3.4 Hz, 1H), 3.50 (dt, *J* = 12.5, 3.8 Hz, 1H), 1.71 (q, *J* = 13.3, 11.1 Hz, 1H), 1.60 (d, *J* = 13.4 Hz, 1H), 1.50 –

1.39 (m, 2H), 1.33 (d, *J* = 13.7 Hz, 1H), 1.08 – 0.95 (m, 1H); ^{13}**C NMR** (101 MHz, DMSO) δ 147.67, 143.58, 141.44, 129.73, 129.05, 128.31, 127.76, 124.89, 124.85, 89.35, 77.47, 65.28, 34.03, 27.00, 21.08, 17.95; **IR** (neat, C=O, cm^{-1}): *v* = 1710; **HRMS** (ESI+; MeOH): *m/z* calcd. ($C_{20}H_{20}NaO_4$) 347.1254 (M+Na)$^+$: found: 347.1261.

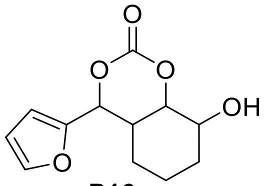

P16

The product was isolated as a white solid, eluent: 50% EA in hexane. Yield: 25%, 6.0 mg, isomeric ratio is 56:44. ^{1}**H NMR** (500 MHz, $CDCl_3$) δ 7.43 (ddd, *J* = 15.6, 1.8, 0.8 Hz, 0.92H), 6.43 (ddt, *J* = 8.1, 3.3, 0.8 Hz, 0.94H), 6.39 (td, *J* = 3.2, 1.8 Hz, 0.94H), 5.58 (d, *J* = 3.2 Hz, 0.55H), 5.35 (d, *J* = 6.0 Hz, 0.43H), 4.63 (t, *J* = 3.2 Hz, 0.56H), 4.50 (dd, *J* = 6.2, 4.2 Hz, 0.44H), 4.17 (t, *J* = 3.2 Hz, 0.56H), 4.08 (dt, *J* = 6.4, 3.2 Hz, 0.44H), 2.78 – 2.70 (m, 0.45H), 2.58 (ddt, *J* = 12.5, 5.5, 3.0 Hz, 0.58H), 2.21 (d, *J* = 3.2 Hz, 0.42H), 1.96 (ddd, *J* = 12.2, 7.8, 3.9 Hz, 0.47H), 1.84 – 1.67 (m, 3.03H), 1.67 – 1.61 (m, 0.7H), 1.59 – 1.51 (m, 1.53H), 1.45 – 1.31 (m, 1.27H); ^{13}**C NMR** (126 MHz, $CDCl_3$) δ 149.42, 148.76, 147.94, 147.83, 143.74, 142.88, 110.77, 110.61, 110.08, 108.63, 79.42, 79.01, 78.20, 76.64, 67.99, 66.59, 32.61, 31.83, 29.25, 27.10, 24.44, 18.91, 18.47, 17.48; **IR** (neat, C=O, cm^{-1}): *v* = 1731; **HRMS** (ESI+; MeOH): *m/z* calcd. ($C_{12}H_{14}NaO_5$) 261.0733 (M+Na)$^+$: found: 261.0731.

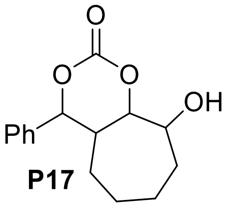

P17

The product was isolated as a white solid, eluent: 50% EA in hexane. Yield: 54%, 14.2 mg. ^{1}**H NMR** (500 MHz, $CDCl_3$) δ 7.44 – 7.39 (m, 2H), 7.36 – 7.32 (m, 3H), 5.60 (d, *J* = 2.5 Hz, 1H), 4.84 (ddd, *J* = 6.5, 5.0, 1.2 Hz, 1H), 3.95 (dd, *J* = 10.3, 6.7 Hz, 1H), 2.87 (s, 1H), 2.40 – 2.33 (m, 1H), 1.87 (d, *J* = 12.0 Hz, 2H), 1.82 – 1.67 (m, 2H), 1.44 (dtd, *J* = 27.6, 14.6, 13.7, 4.9 Hz, 2H), 1.32 – 1.24 (m, 1H), 1.11 – 1.01 (m, 1H); ^{13}**C NMR** (126 MHz, $CDCl_3$) δ 149.27, 135.85, 128.86, 128.37, 125.33, 89.87, 81.15, 76.53, 40.52, 31.83, 29.50, 26.58, 19.75; **IR** (neat, C=O, cm^{-1}): *v* = 1742; **HRMS** (ESI+; MeOH): *m/z* calcd. ($C_{15}H_{18}NaO_4$) 285.1097 (M+Na)$^+$: found: 285.1095.

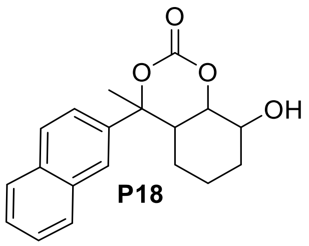

The product was isolated as a light-yellow solid, eluent: 50% EA in hexane. Yield: 65%, 20.3 mg. ^{1}**H NMR** (500 MHz, $CDCl_3$) δ 8.06 – 7.75 (m, 4H), 7.58 – 7.32 (m, 3H), 4.94 (t, *J* = 3.3 Hz, 1H), 4.25 (s, 1H), 2.51 (ddd, *J* = 12.3, 5.4, 2.9 Hz, 1H), 1.96 (s, 1H), 1.89 (s, 3H), 1.79 – 1.68 (m, 2H), 1.57 (ddt, *J* = 17.2, 8.7, 4.5 Hz, 1H), 1.40 (dt, *J* = 13.6, 3.5 Hz, 1H), 1.10 – 0.96 (m, 2H); ^{13}**C NMR** (101 MHz, $CDCl_3$) δ 148.76, 140.65, 133.21, 132.58, 128.68, 128.48, 127.65, 126.77, 126.51, 123.27, 121.92, 86.91, 75.89, 66.74, 36.19, 28.45, 27.08, 21.47, 17.76; **IR** (neat, C=O, cm^{-1}): *v* = 1723; **HRMS** (ESI+; MeOH): *m/z* calcd. ($C_{19}H_{20}NaO_4$) 335.1254 $(M+Na)^+$: found: 335.1252.

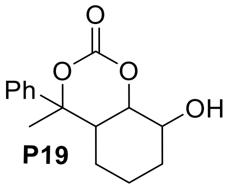

The product was isolated as a white solid, eluent: 50% EA in hexane. Yield: 81%, 21.3 mg. ^{1}**H NMR** (500 MHz, $CDCl_3$) δ 7.47 – 7.35 (m, 3H), 7.34 – 7.27 (m, 2H), 4.88 (t, *J* = 3.2 Hz, 1H), 4.22 (q, *J* = 3.1 Hz, 1H), 2.38 (ddd, *J* = 11.5, 6.7, 2.8 Hz, 1H), 1.81 (s, 3H), 1.76 – 1.64 (m, 2H), 1.61 – 1.50 (m, 1H), 1.43 (dq, *J* = 13.6, 3.6 Hz, 1H), 1.02 – 0.96 (m, 2H); ^{13}**C NMR** (126 MHz, $CDCl_3$) δ 148.74, 141.90, 128.73, 127.62, 124.12, 86.78, 75.91, 66.71, 36.39, 28.48, 27.07, 21.34, 17.78; **IR** (neat, C=O, cm^{-1}): *v* = 1721; **HRMS** (ESI+; MeOH): *m/z* calcd. ($C_{15}H_{18}NaO_4$) 185.1097 $(M+Na)^+$: found: 185.1100.

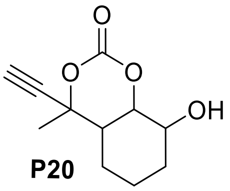

The product was isolated as a white solid, eluent: 50% EA in hexane. Yield: 62%, 13.0 mg. ^{1}**H NMR** (500 MHz, $CDCl_3$) δ 4.61 (t, *J* = 3.8 Hz, 1H), 4.21 (t, *J* = 3.5 Hz, 1H), 2.74 (s, 1H), 2.20 (ddd, *J* = 12.3, 4.7, 3.1 Hz, 1H), 2.13 – 2.06 (m, 1H), 1.95 (d, *J* = 3.7 Hz, 1H), 1.81 (s, 3H), 1.79 – 1.65 (m, 4H), 1.52 – 1.42 (m, 1H); ^{13}**C NMR** (126 MHz, $CDCl_3$) δ 147.66, 80.96, 80.11, 77.01, 75.61, 66.60, 36.21, 28.27, 27.33, 22.31, 17.95; **IR** (neat, C=O, cm^{-1}): *v* = 1726; **HRMS** (ESI+; MeOH): *m/z* calcd. ($C_{11}H_{14}NaO_4$) 233.0784 $(M+Na)^+$: found: 233.0783.

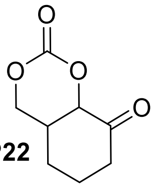

P22

The product was isolated as a white solid, eluent: 50% EA in hexane. Yield: 75%, 25.5 mg, exo/endo 65:35 **^{1}H NMR** (400 MHz, $CDCl_3$) δ 4.95 (dt, *J* = 5.3, 1.2 Hz, 1H), 4.76 (dd, *J* = 11.9, 1.2 Hz, 1.85H), 4.44 (dd, *J* = 10.6, 5.1 Hz, 2H), 4.36 (ddd, *J* = 11.3, 5.0, 1.5 Hz, 1.13H), 4.30 – 4.15 (m, 3H), 2.87 (dp, *J* = 9.8, 5.1 Hz, 1H), 2.67 – 2.39 (m, 6.11H), 2.37 – 2.15 (m, 3.99H), 2.09 – 1.75 (m, 8.18H), 1.58 (tdd, *J* = 13.3, 12.0, 3.9 Hz, 2.32H); **^{13}C NMR** (101 MHz, $CDCl_3$) δ 203.47, 199.92, 147.24, 146.72, 82.10, 81.90, 71.71, 69.08, 39.55, 38.94, 38.31, 34.70, 25.49, 24.83, 23.67, 23.46; **IR** (neat, C=O, cm^{-1}): *ν* = 1741; **HRMS** (ESI+; MeOH): *m/z* calcd. ($C_8H_{10}O_4$) 193.0471 $(M+H)^+$: found: 193.0466.

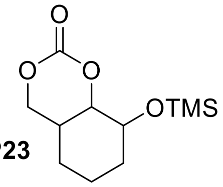

P23

The product was isolated as a white solid, eluent: 10% EA in hexane. Yield: 91%, 44.5 mg. **^{1}H NMR** (500 MHz, $CDCl_3$) δ 4.45 (dd, *J* = 11.0, 3.7 Hz, 1H), 4.32 (t, *J* = 3.5 Hz, 1H), 4.18 (dd, *J* = 11.0, 1.8 Hz, 1H), 4.00 (q, *J* = 3.1 Hz, 1H), 2.30 – 2.23 (m, 1H), 1.78 – 1.64 (m, 2H), 1.58 – 1.50 (m, 4H), 0.12 (s, 9H); **^{13}C NMR** (126 MHz, $CDCl_3$) δ 148.35, 79.21, 77.42, 77.16, 76.91, 73.04, 67.12, 27.81, 27.45, 22.80, 17.92, 0.08; **IR** (neat, C=O, cm^{-1}): *ν* = 1757; **HRMS** (ESI+; MeOH): *m/z* calcd. ($C_{11}H_{20}NaO_4Si$) 267.1023 $(M+Na)^+$: found: 267.1029.

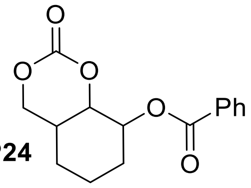

P24

The product was isolated as a white solid, eluent: 50% EA in hexane. Yield: 74%, 41.0 mg. **^{1}H NMR** (400 MHz, $CDCl_3$) δ 8.05 – 7.99 (m, 2H), 7.62 – 7.55 (m, 1H), 7.49 – 7.42 (m, 2H), 5.35 (q, *J* = 3.5 Hz, 1H), 4.69 (t, *J* = 3.6 Hz, 1H), 4.49 (dd, *J* = 11.1, 3.7 Hz, 1H), 4.25 (dd, *J* = 11.1, 2.3 Hz, 1H), 2.30 (tdd, *J* = 7.0, 5.4, 3.3 Hz, 1H), 1.91 (dt, *J* = 7.3, 3.6 Hz, 2H), 1.82 – 1.65 (m, 4H); **^{13}C NMR** (101 MHz, $CDCl_3$) δ 165.19, 147.68, 133.59, 129.75, 128.65, 76.05, 72.27, 69.05, 28.82, 24.71, 22.53, 18.86; **IR** (neat, C=O, cm^{-1}): *ν* = 1749, 1717; **HRMS** (ESI+; MeOH): *m/z* calcd. ($C_{15}H_{16}NaO_5$) 299.0890 $(M+Na)^+$: found: 299.0892.

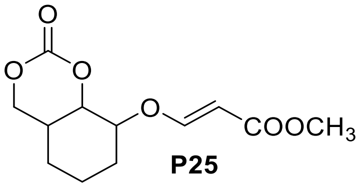

P25

The product was isolated as a colorless oil, eluent: 50% EA in hexane. Yield: 76%, 38.9 mg. **^{1}H NMR** (500 MHz, $CDCl_3$) δ 7.48 (dd, *J* = 12.5, 0.9 Hz, 1H), 5.33 (dd, *J* = 12.5, 1.0 Hz, 1H), 4.60 (dt, *J* = 3.8, 2.0 Hz, 1H), 4.53 – 4.45 (m, 1H), 4.28 (d, *J* = 3.2 Hz, 1H), 4.22 (dd, *J* = 11.2, 1.7 Hz, 1H), 3.70 (s, 3H), 2.28 – 2.16 (m, 1H), 1.94 – 1.86 (m, 1H), 1.82 – 1.72 (m, 1H), 1.70 – 1.59 (m, 4H); **^{13}C NMR** (126 MHz, $CDCl_3$) δ 167.82, 160.27, 147.46, 99.09, 76.45, 75.83, 72.53, 51.42, 28.18, 24.25, 22.26, 18.02; **IR** (neat, C=O, cm^{-1}): *v* = 1748, 1703, 1640, 1625; **HRMS** (ESI+; MeOH): *m/z* calcd. ($C_{12}H_{16}NaO_6$) 279.0839 $(M+Na)^+$: found: 279.0841.

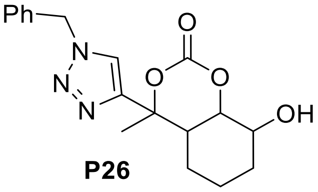

P26

The product was isolated as a white solid, eluent: 70% EA in hexane. Yield: 85%, 58.4 mg. **^{1}H NMR** (400 MHz, $CDCl_3$) δ 7.50 (s, 1H), 7.40 (dd, *J* = 5.1, 1.9 Hz, 3H), 7.31 – 7.27 (m, 2H), 5.53 (d, *J* = 14.7 Hz, 1H), 5.45 (d, *J* = 14.7 Hz, 1H), 4.20 (t, *J* = 3.2 Hz, 1H), 4.02 (s, 1H), 2.91 (ddd, *J* = 13.1, 4.8, 2.7 Hz, 1H), 1.93 (dd, *J* = 13.4, 4.0 Hz, 2H), 1.82 – 1.65 (m, 4H), 1.63 (s, 3H), 1.40 (qd, *J* = 12.8, 4.1 Hz, 1H); **^{13}C NMR** (101 MHz, $CDCl_3$) δ 151.82, 148.40, 133.97, 129.48, 129.27, 128.48, 121.20, 84.00, 76.58, 66.34, 54.67, 34.35, 26.69, 26.30, 19.93, 17.73; **IR** (neat, C=O, cm^{-1}): *v* = 1734; **HRMS** (ESI+; MeOH): *m/z* calcd. ($C_{18}H_{21}N_3NaO_4$) 366.1424 $(M+Na)^+$: found: 366.1424.

Intramolecular isomerization product

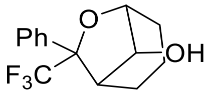

P21

The product was isolated as a colorless oil, eluent: 30% EA in hexane. Yield: 56%, 15.3 mg. **^{1}H NMR** (400 MHz, $CDCl_3$) δ 7.48 – 7.34 (m, 5H), 4.89 (dd, *J* = 8.2, 5.0 Hz, 1H), 4.06 (dt, *J* = 10.0, 5.2 Hz, 1H), 3.56 (q, *J* = 7.8 Hz, 1H), 1.98 (s, 1H), 1.84 – 1.72 (m, 1H), 1.54 – 1.35 (m, 3H), 1.31 – 1.18 (m, 2H); **^{13}C NMR** (101 MHz, $CDCl_3$) δ 134.41, 129.70, 128.70, 128.28, 126.87, 126.31, 124.04, 121.21, 86.78 (q, *J* = 30.2 Hz), 82.93, 71.35, 37.34, 26.70, 20.81, 17.75; **^{19}F NMR** (376 MHz, $CDCl_3$) δ -81.50; **HRMS** (APCI+; MeOH): *m/z* calcd. ($C_{14}H_{15}F_3NaO_2$) 295.0916 $(M+Na)^+$: found: 295.0925.

5-Membered cyclic carbonate product

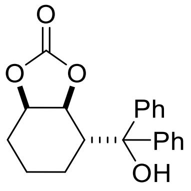

P15[b]

The product was isolated as white solid, eluent: 20% EA in hexane. Yield: 18%, 5.8 mg. **^{1}H NMR** (500 MHz, $CDCl_3$) δ 7.42 (ddd, *J* = 8.2, 2.7, 1.2 Hz, 4H), 7.34 (ddd, *J* = 13.0, 8.6, 7.0 Hz, 4H), 7.27 – 7.21 (m, 3H), 4.82 (t, *J* = 6.6 Hz, 1H), 4.74 (dt, *J* = 6.5, 4.2 Hz, 1H), 2.97 (ddd, *J* = 10.4, 6.6, 5.0 Hz, 1H), 2.54 (s, 1H), 2.13 – 2.03 (m, 1H), 1.84 – 1.77 (m, 1H), 1.75 – 1.60 (m, 3H), 1.11 (dtd, *J* = 14.3, 10.6, 3.9 Hz, 1H); **^{13}C NMR** (126 MHz, $CDCl_3$) δ 154.88, 145.03, 144.92, 128.65, 128.54, 127.46, 127.41, 126.12, 125.96, 80.17, 76.84, 76.71, 46.26, 27.16, 23.99, 18.55; **IR (neat, C=O, cm^{-1}):** *v* = 1783; **HRMS** (ESI+; MeOH): *m/z* calcd. ($C_{20}H_{20}NaO_4$) 347.1254 $(M+Na)^+$: found: 347.1256.

Products derived from the from *syn* isomer of substrate 1a

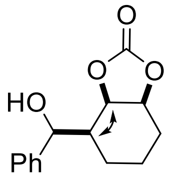

P1[a] *syn*

The product was isolated as a colorless oil, eluent: 15% EA in hexane. Yield: 29%, 36.0 mg. **^{1}H NMR** (400 MHz, $CDCl_3$) δ 7.44 – 7.29 (m, 5H), 4.71 (d, *J* = 8.8 Hz, 1H), 4.57 (dt, *J* = 8.0, 6.3 Hz, 1H), 4.17 (ddd, *J* = 6.6, 2.9, 1.0 Hz, 1H), 2.23 – 2.12 (m, 1H), 2.08 – 1.99 (m, 1H), 1.95 – 1.79 (m, 2H), 1.65 – 1.45 (m, 3H), 1.41 – 1.30 (m, 1H); **^{13}C NMR** (101 MHz, $CDCl_3$) δ 155.08, 142.00, 128.89, 128.49, 126.74, 76.80, 76.03, 75.62, 44.81, 26.94, 20.62, 18.70; **IR** (neat, C=O, cm^{-1}): *ν* = 1787; **HRMS** (ESI+; MeOH): *m/z* calcd. ($C_{14}H_{16}NaO_4$) 271.0941 $(M+Na)^+$: found: 271.0940.

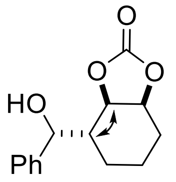

P1[b] *anti*

The product was isolated as a white solid, eluent: 20% EA in hexane. Yield: 37%, 45.9 mg. **^{1}H NMR** (500 MHz, $CDCl_3$) δ 7.39 – 7.26 (m, 5H), 5.08 (d, *J* = 2.5 Hz, 1H), 4.89 (dd, *J* = 9.3, 6.2 Hz, 1H), 4.82 (ddd, *J* = 6.4, 4.0, 2.8 Hz, 1H), 2.28 – 2.20 (m, 1H), 2.14 (s, 1H), 1.81 – 1.74 (m, 1H), 1.71 – 1.54 (m, 3H), 1.44 (dqd, *J* = 13.4, 3.7, 1.9 Hz, 1H), 1.37 – 1.28 (m, 1H), 1.20 (dtd, *J* = 13.7, 12.5, 3.2 Hz, 1H); **^{13}C NMR** (126 MHz, $CDCl_3$) δ 155.54, 142.19, 128.52, 127.67, 125.77, 77.23, 77.14, 72.09, 47.21, 26.55, 19.39, 18.97; **IR** (neat, C=O, cm^{-1}): *ν* = 1786; **HRMS** (ESI+; MeOH): *m/z* calcd. ($C_{14}H_{16}NaO_4$) 271.0941 $(M+Na)^+$: found: 271.0937.

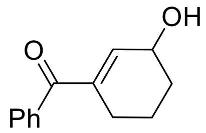

ByP1

The product was isolated as white solid, eluent: 15% EA in hexane. Yield: 7%, 7.1 mg. **^{1}H NMR** (500 MHz, $CDCl_3$) δ 7.72 – 7.64 (m, 2H), 7.56 – 7.49 (m, 1H), 7.46 – 7.39 (m, 2H), 6.43 (qd, *J* = 1.9, 0.9 Hz, 1H), 4.45 (s, 1H), 2.49 – 2.35 (m, 2H), 2.02 (dddd, *J* = 12.5, 8.2, 5.1, 2.2 Hz, 1H), 1.91 (dddd, *J* = 13.3, 10.7, 5.4, 3.1 Hz, 1H), 1.77 – 1.62 (m, 3H); **^{13}C NMR** (126 MHz, $CDCl_3$) δ 198.23, 142.45, 140.20, 137.88, 132.07, 129.47, 128.35, 66.48, 31.61, 24.40, 19.27; **IR** (neat,

C=O, cm^{-1}): v = 1637; **HRMS** (ESI+; MeOH): *m/z* calcd. ($C_{13}H_{14}NaO_2$) 225.0886 $(M+Na)^+$: found: 225.0882.

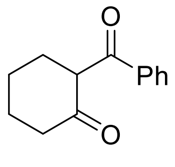

ByP2

The product was isolated as yellow oil, eluent: 20% EA in hexane. Yield: 7%, 7.2 mg **^{1}H NMR** (500 MHz, $CDCl_3$) δ 7.96 – 7.92 (m, 2H), 7.61 – 7.57 (m, 1H), 7.49 (ddt, *J* = 7.9, 6.7, 1.2 Hz, 2H), 3.82 (tt, *J* = 10.6, 4.2 Hz, 1H), 2.72 (ddd, *J* = 14.5, 10.8, 1.1 Hz, 1H), 2.53 – 2.39 (m, 3H), 2.12 (dddd, *J* = 11.2, 6.9, 5.7, 3.1 Hz, 2H), 1.90 – 1.80 (m, 2H);**^{13}C NMR** (126 MHz, $CDCl_3$) δ 210.42, 200.56, 135.49, 133.65, 129.02, 128.54, 45.33, 43.31, 41.15, 28.55, 24.98; **IR** (neat, C=O, cm^{-1}): v = 1709, 1676; **HRMS** (ESI+; MeOH): *m/z* calcd. ($C_{13}H_{14}NaO_2$) 225.0886 $(M+Na)^+$: found: 225.0886.

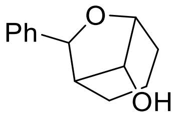

ByP3

The product was isolated as white solid, eluent: 30% EA in hexane. Yield: 46%, 47.0 mg. **^{1}H NMR** (500 MHz, $CDCl_3$) δ 7.40 (dq, *J* = 7.4, 1.2 Hz, 2H), 7.35 (dd, *J* = 8.6, 6.9 Hz, 2H), 7.26 – 7.22 (m, 1H), 5.28 (d, *J* = 4.1 Hz, 1H), 4.55 (q, *J* = 4.9 Hz, 1H), 4.31 (t, *J* = 5.3 Hz, 1H), 2.55 (p, *J* = 3.8 Hz, 1H), 1.95 (d, *J* = 4.6 Hz, 1H), 1.86 – 1.73 (m, 2H), 1.70 – 1.64 (m, 1H), 1.50 – 1.38 (m, 1H), 1.25 – 1.14 (m, 2H); **^{13}C NMR** (126 MHz, $CDCl_3$) δ 140.43, 128.19, 126.48, 125.34, 80.01, 76.52, 72.26, 42.30, 24.84, 18.95, 16.58; **HRMS** (APCI+; MeOH): *m/z* calcd. ($C_{13}H_{15}O$) 187.1121 $(M-OH)^+$: found: 187.1117.

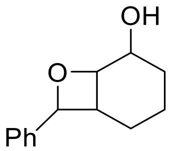

ByP4

The product was isolated as white solid, eluent: 15% EA in hexane. Yield: 16%, 16.3 mg. **^{1}H NMR** (500 MHz, $CDCl_3$) δ 7.43 (dq, *J* = 7.5, 1.2 Hz, 2H), 7.37 – 7.34 (m, 2H), 7.23 (dddd, *J* = 7.7, 6.7, 2.3, 1.1 Hz, 1H), 5.49 (d, *J* = 4.4 Hz, 1H), 4.36 (d, *J* = 5.2 Hz, 1H), 4.07 (d, *J* = 3.5 Hz, 1H), 2.61 (s, 1H), 1.97 – 1.88 (m, 2H), 1.52 – 1.46 (m, 2H), 1.40 – 1.33 (m, 2H), 1.14 – 1.06 (m, 1H); **^{13}C NMR** (126 MHz, $CDCl_3$) δ 140.20, 128.17, 126.37, 125.59, 82.06, 80.83, 80.68, 45.96,

31.11, 25.82, 16.46; **HRMS** (ESI+; MeOH): *m/z* calcd. ($C_{13}H_{16}NaO_2$) 227.1043 $(M+Na)^+$: found: 227.1040.

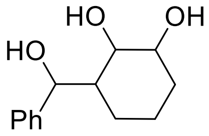

ByP5

The product was isolated as white solid, eluent: 50% EA in hexane. Yield: 72%, 80 mg. **^{1}H NMR** (500 MHz, $CDCl_3$) δ 7.36 – 7.31 (m, 4H), 7.28 – 7.26 (m, 1H), 5.02 (d, *J* = 3.2 Hz, 1H), 3.98 (t, *J* = 3.0 Hz, 1H), 3.88 (q, *J* = 3.3 Hz, 1H), 2.02 (dtd, *J* = 12.5, 3.6, 2.5 Hz, 1H), 1.93 – 1.81 (m, 1H), 1.64 (qd, *J* = 12.8, 4.0 Hz, 1H), 1.56 – 1.39 (m, 3H), 1.30 – 1.25 (m, 1H); **^{13}C NMR** (126 MHz, $CDCl_3$) δ 143.13, 128.35, 127.39, 125.92, 78.08, 74.77, 70.32, 42.43, 27.68, 19.44, 18.07; **HRMS** (ESI+; MeOH): *m/z* calcd. ($C_{13}H_{18}NaO_3$) 245.1148 $(M+Na)^+$: found: 245.1150.

S35. References

[1] C. W. Bond, A. J. Cresswell, S. G. Davies, A. M. Fletcher, W. Kurosawa, J. A. Lee, P. M. Roberts, A. J. Russell, A. D. Smith, J. E. Thomson, *J. Org. Chem.* **2009**, *74*, 6735–6748.

[2] X.-Y. Liu, B.-Q. Cheng, Y.-C. Guo, X.-Q. Chu, W. Rao, T.-P. Loh, Z.-L. Shen, *Org. Chem. Front.* **2019**, *6*, 1581–1586.

[3] Z. Peng, T. D. Blümke, P. Mayer, P. Knochel, *Angew. Chem. Int. Ed.* **2010**, *49*, 8516−8519.

[4] T. Itoh, K. Jitsukawa, K. Kaneda, S. Teranishi, *J. Am. Chem. Soc.* **1979**, *101*, 159–169.

[5] C. J. Whiteoak, N. Kielland, V. Laserna, F. Castro-Gómez, E. Martin, E. C. Escudero-Adán, C. Bo, A. W. Kleij, *Chem. Eur. J.* **2014**, *20*, 2264-2275.

S36. IR, ^{1}H NMR, ^{13}C NMR and ^{19}F NMR spectra

^{1}H/^{13}C NMR spectra for the cyclic epoxy alcohol products

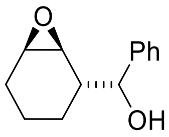

1a (*anti*)

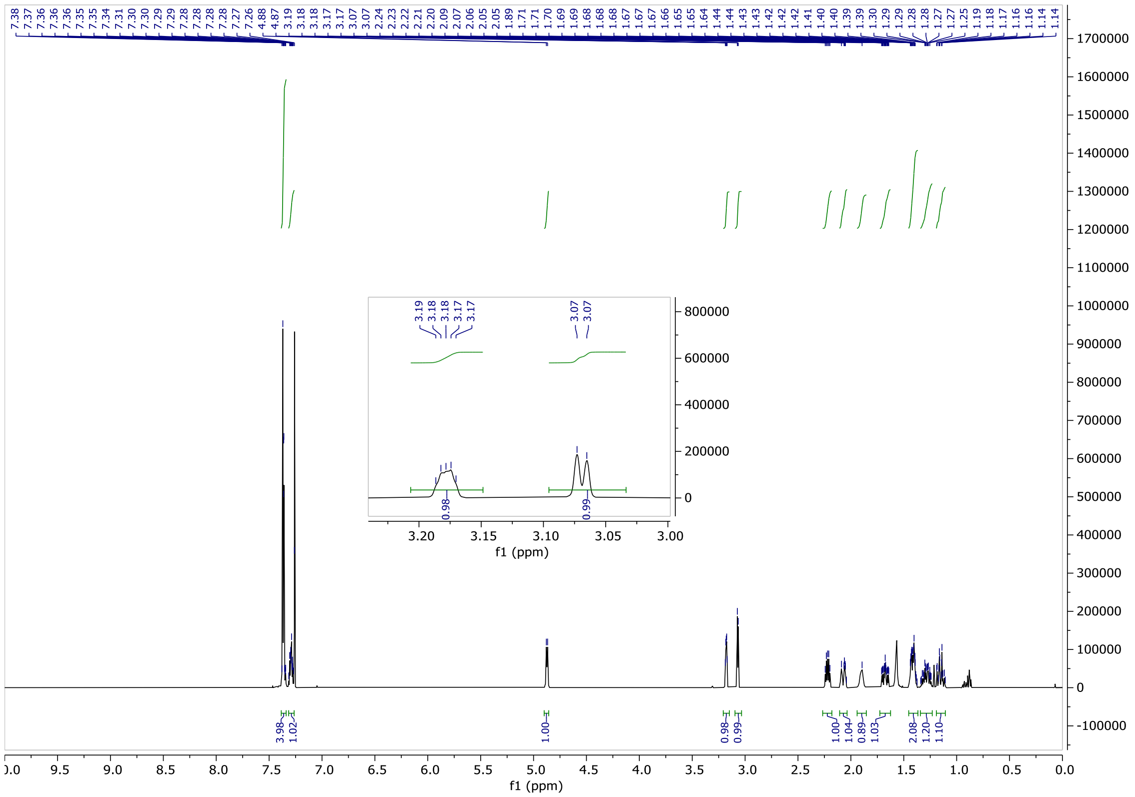

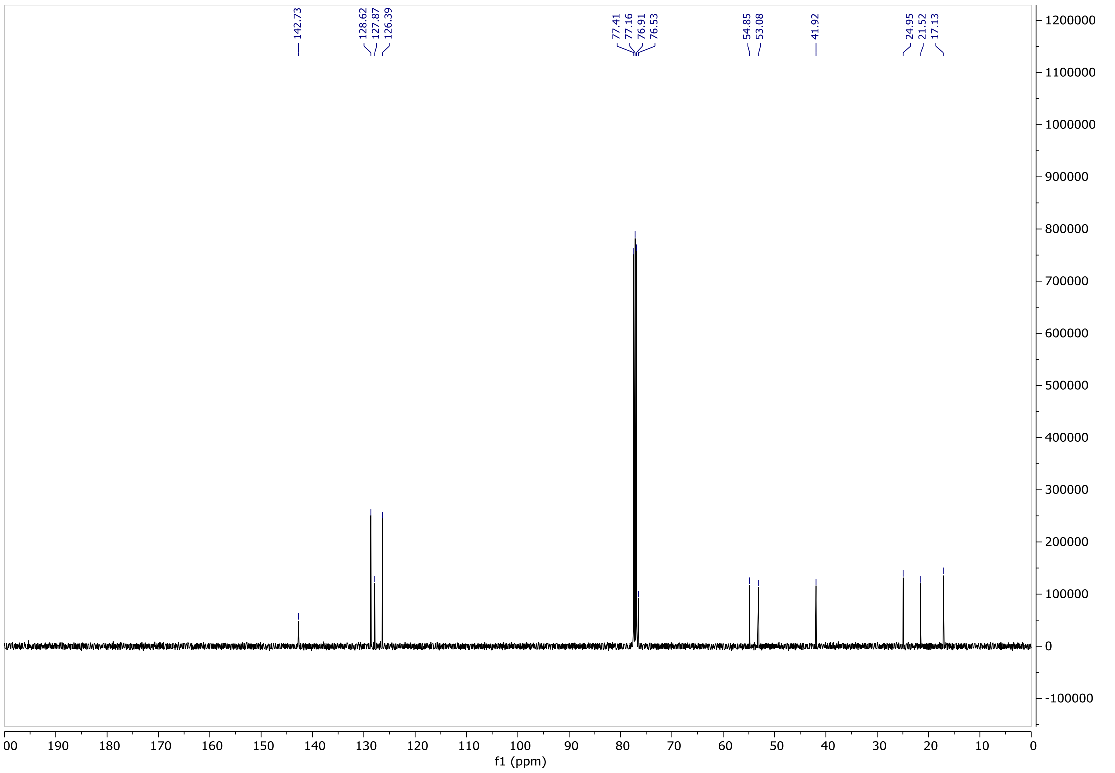
142.73
128.62
127.87
126.39
77.41
77.16
76.91
76.53
54.85
53.08
41.92
24.95
21.52
17.13
1200000
1100000
1000000
900000
800000
700000
600000
500000
400000
300000
200000
100000
0
-100000
00 190 180 170 160 150 140 130 120 110 100 90 80 70 60 50 40 30 20 10 0
f1 (ppm)

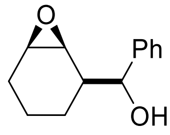
O
Ph
OH

1a (*syn*)

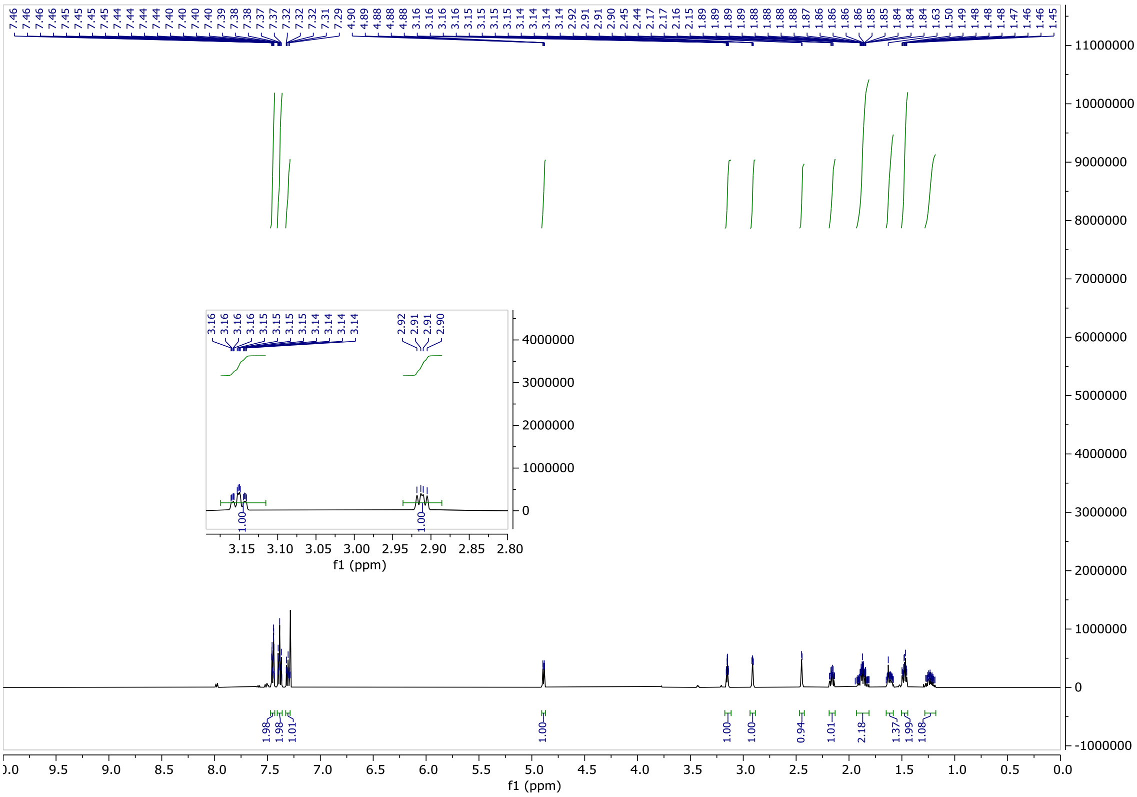
7.46 7.46 7.46 7.45 7.45 7.45 7.44 7.44 7.44 7.40 7.40 7.40 7.39 7.38 7.38 7.37 7.37 7.32 7.32 7.32 7.31 7.29 4.90 4.89 4.88 4.88 3.16 3.16 3.16 3.15 3.15 3.15 3.15 3.14 3.14 3.14 2.92 2.91 2.91 2.90 2.45 2.44 2.17 2.17 2.16 2.15 1.89 1.89 1.89 1.88 1.88 1.88 1.88 1.87 1.86 1.86 1.86 1.86 1.85 1.85 1.84 1.84 1.84 1.63 1.50 1.49 1.48 1.48 1.48 1.47 1.46 1.46 1.45
3.16 3.16 3.16 3.15 3.15 3.15 3.15 3.14 3.14 3.14
2.92 2.91 2.91 2.90
1.00
1.00
f1 (ppm)
1.98 1.98 1.01 1.00 1.00 1.00 0.94 1.01 2.18 1.37 1.99 1.08
f1 (ppm)

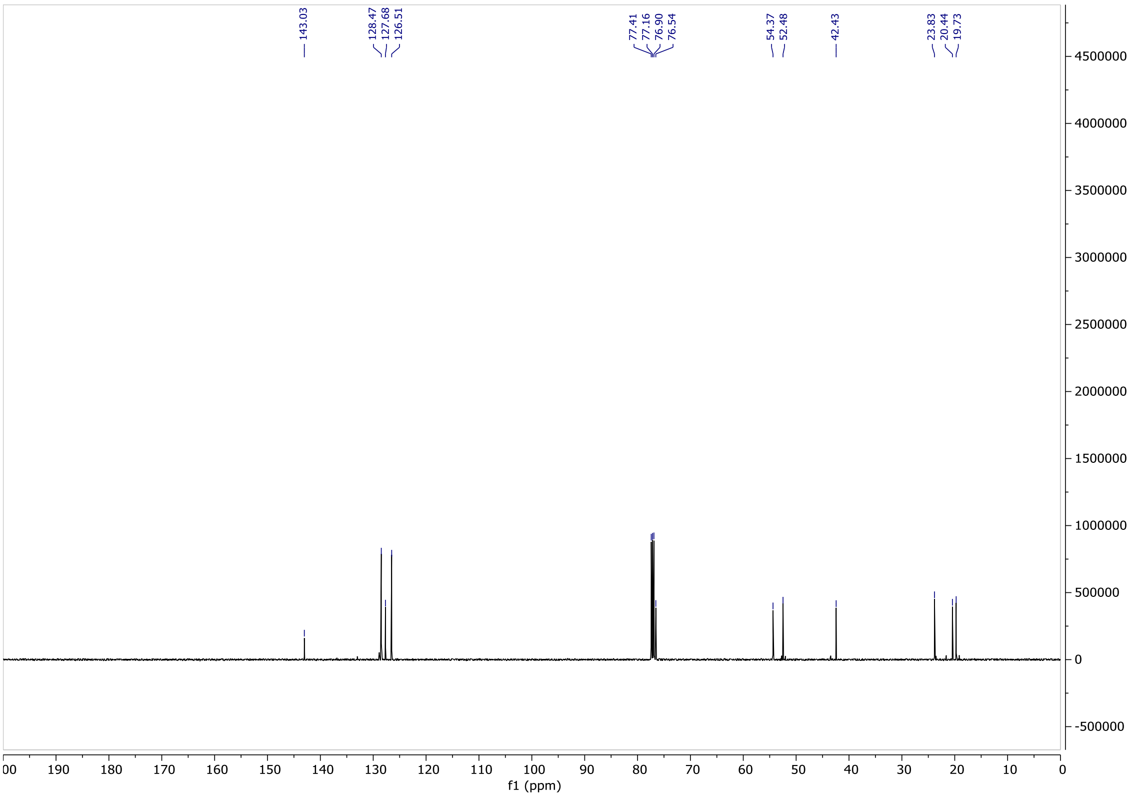
143.03
128.47 127.68 126.51
77.41 77.16 76.90 76.54
54.37 52.48
42.43
23.83 20.44 19.73
f1 (ppm)

1b

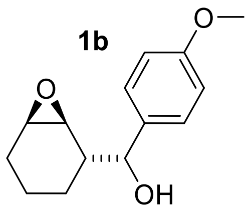

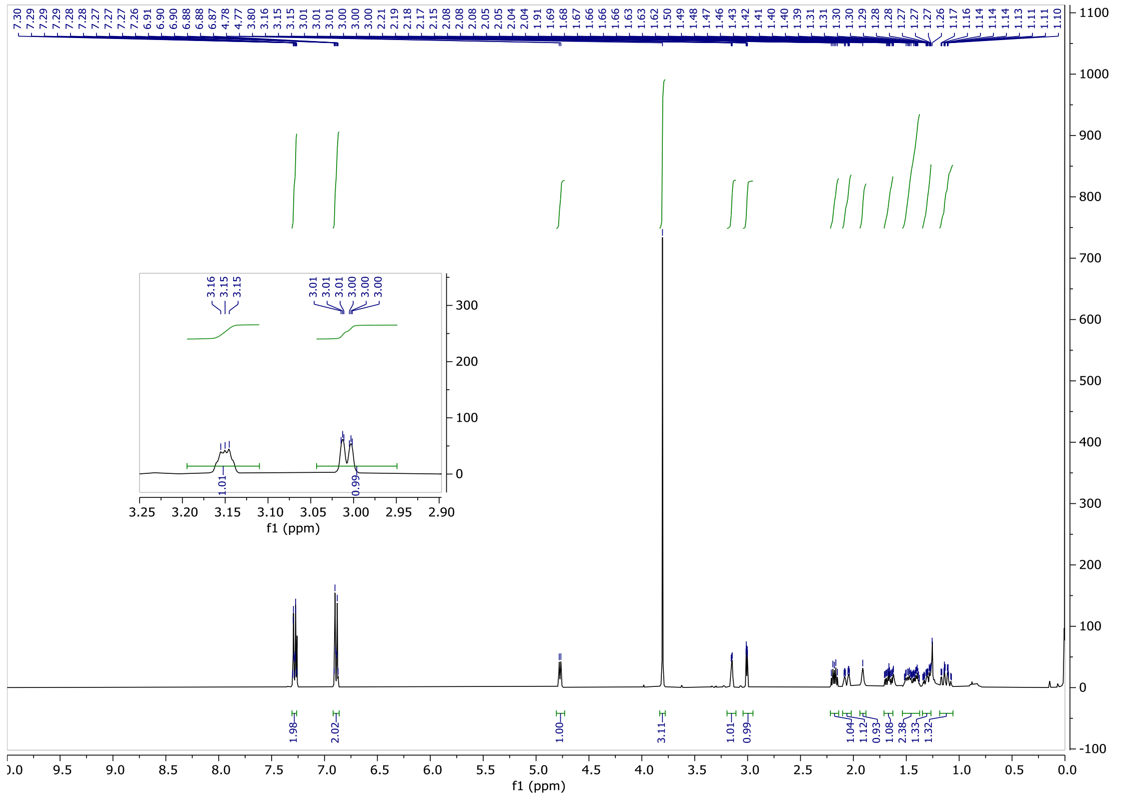

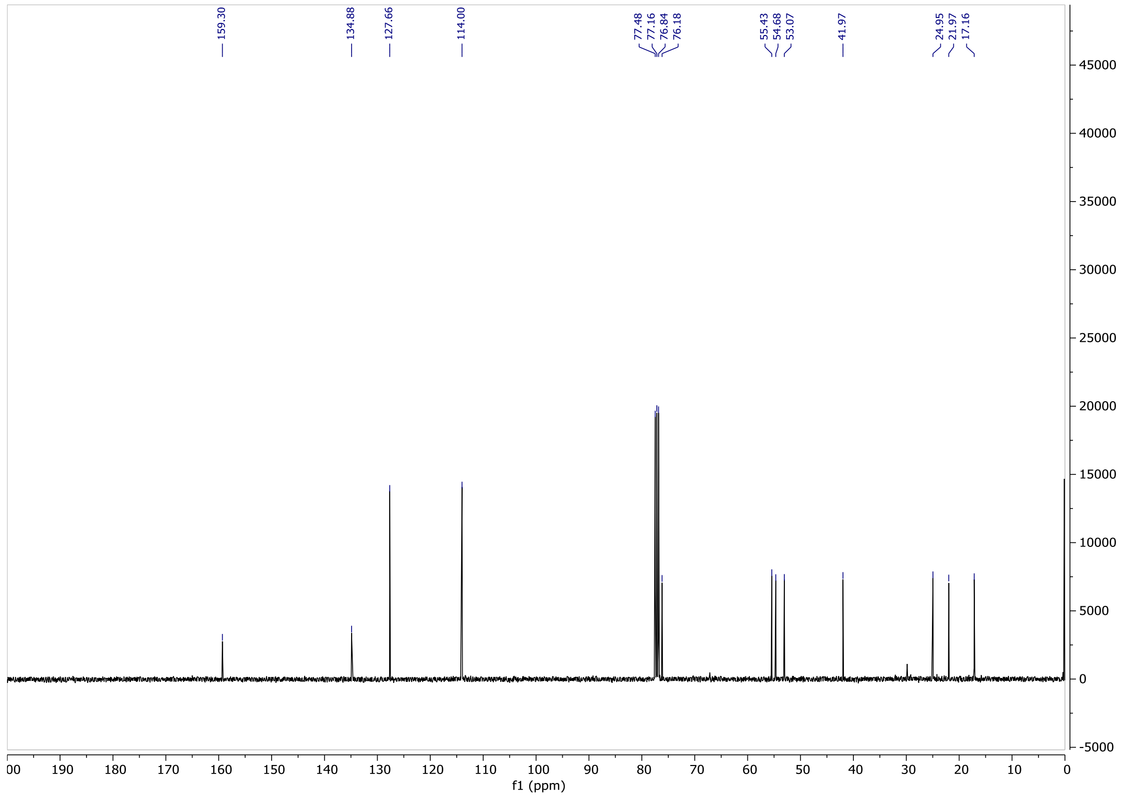

1c

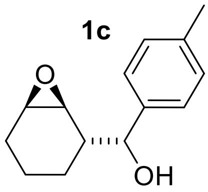
O
OH

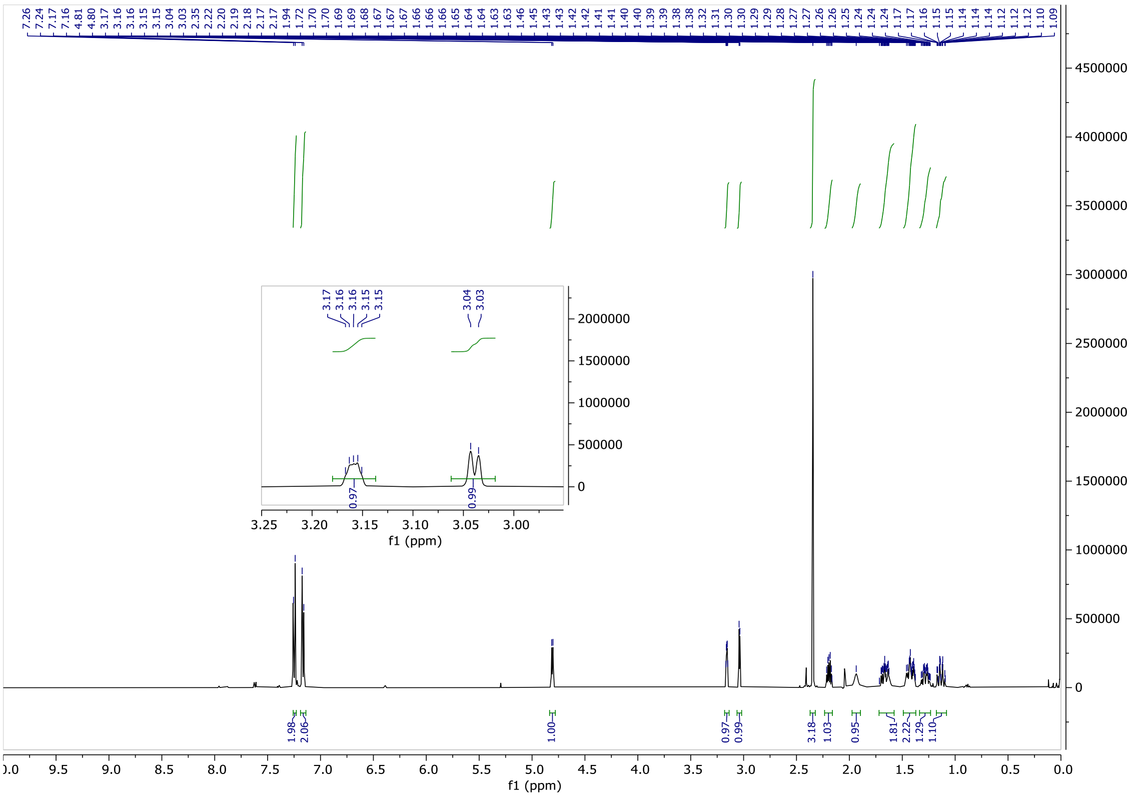
f1 (ppm)

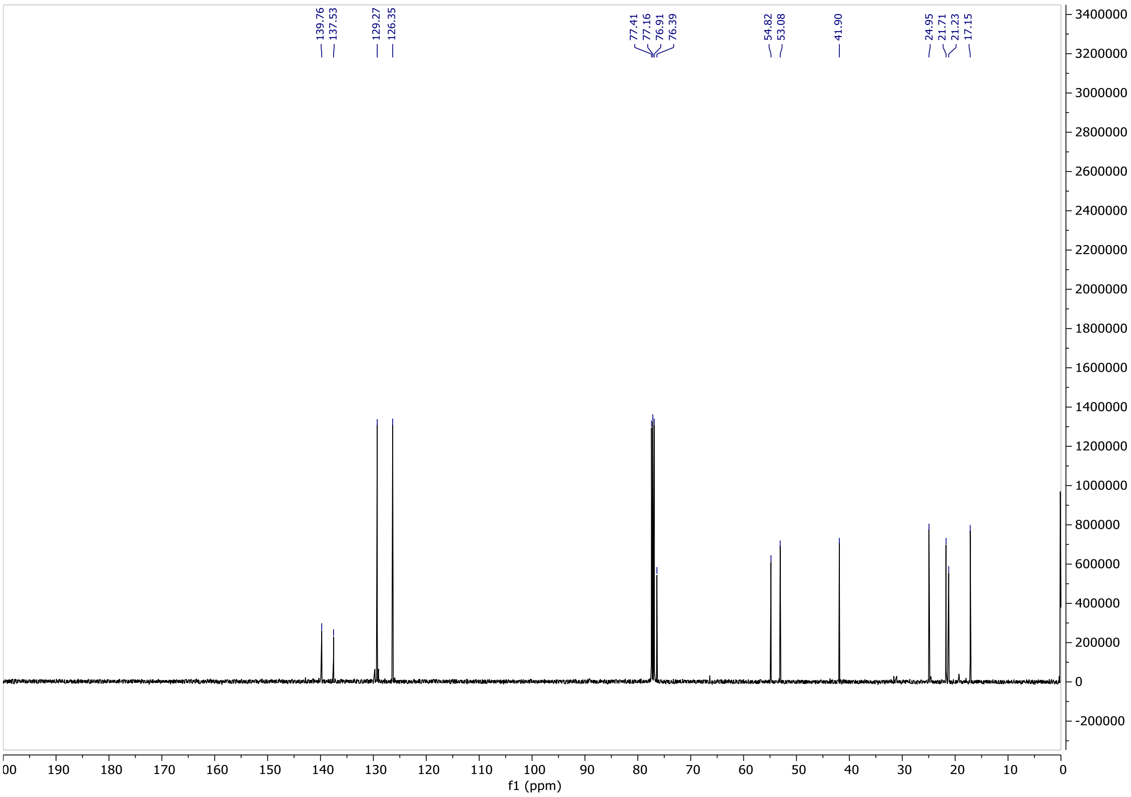
139.76
137.53
129.27
126.35
77.41
77.16
76.91
76.39
54.82
53.08
41.90
24.95
21.71
21.23
17.15
f1 (ppm)

1d

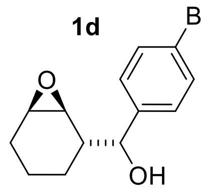

Br
O
OH

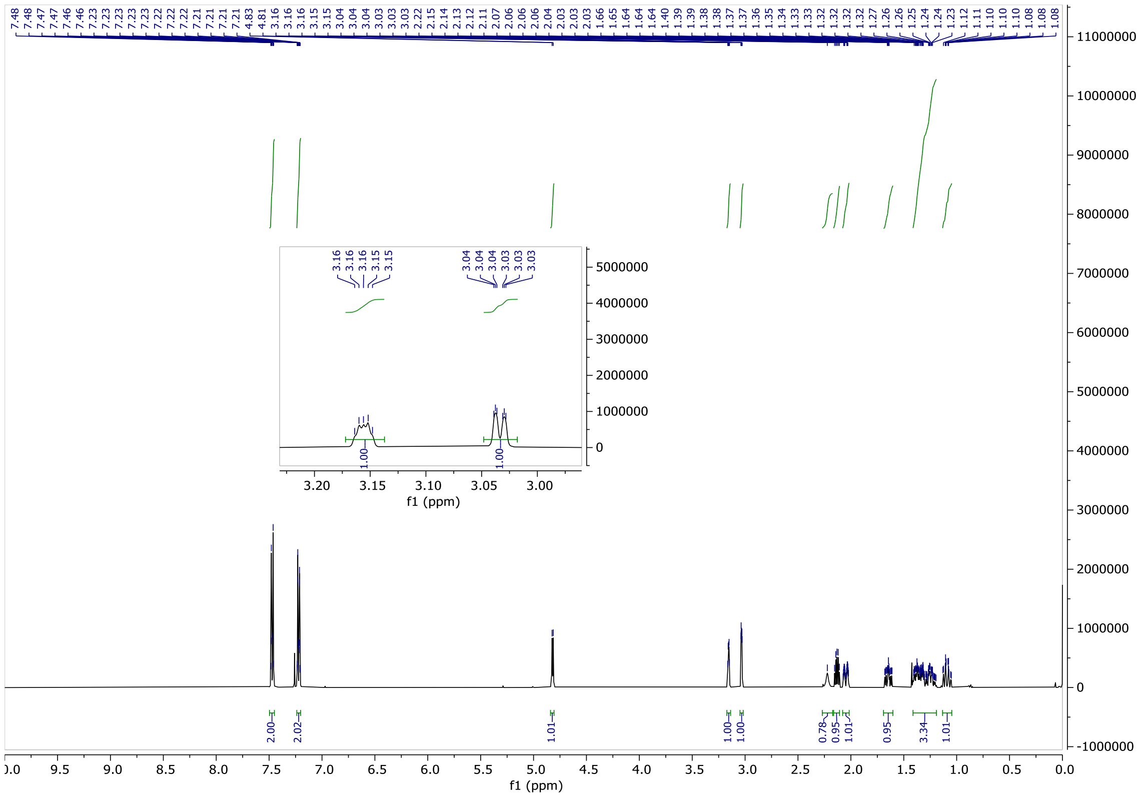

f1 (ppm)

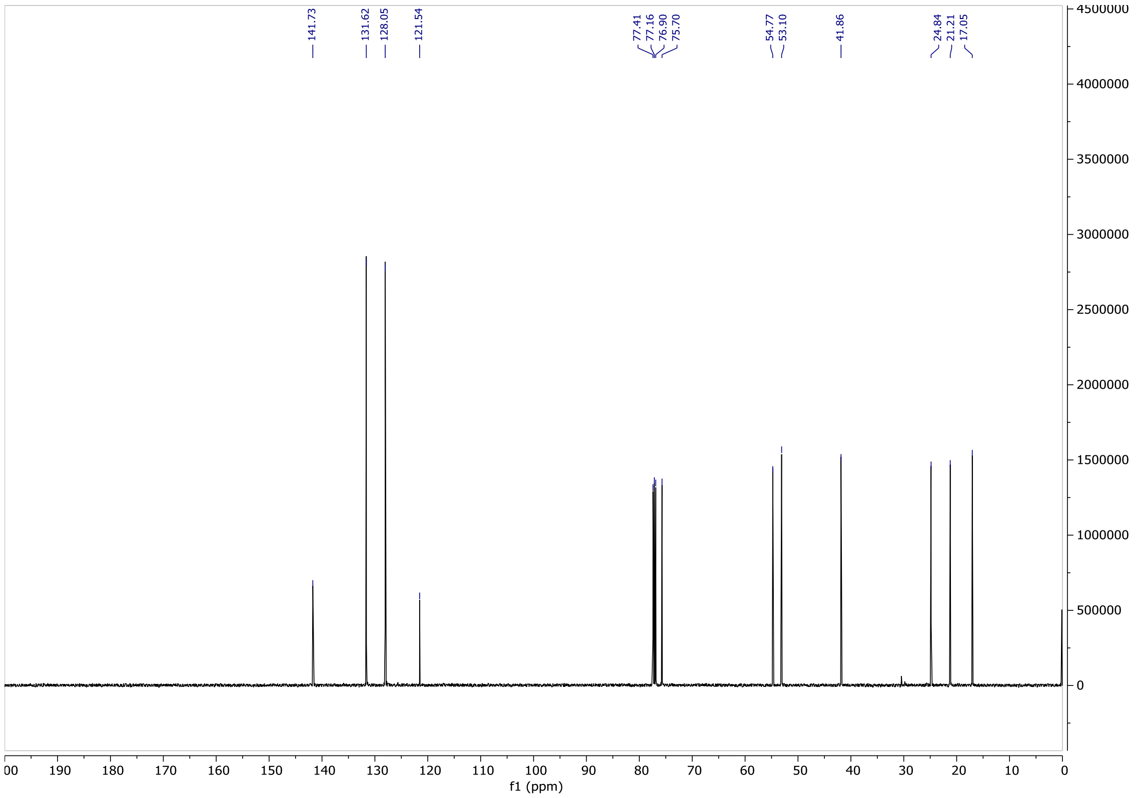

141.73
131.62
128.05
121.54
77.41
77.16
76.90
75.70
54.77
53.10
41.86
24.84
21.21
17.05
f1 (ppm)

1e

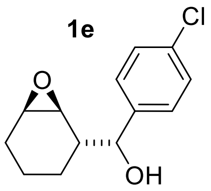

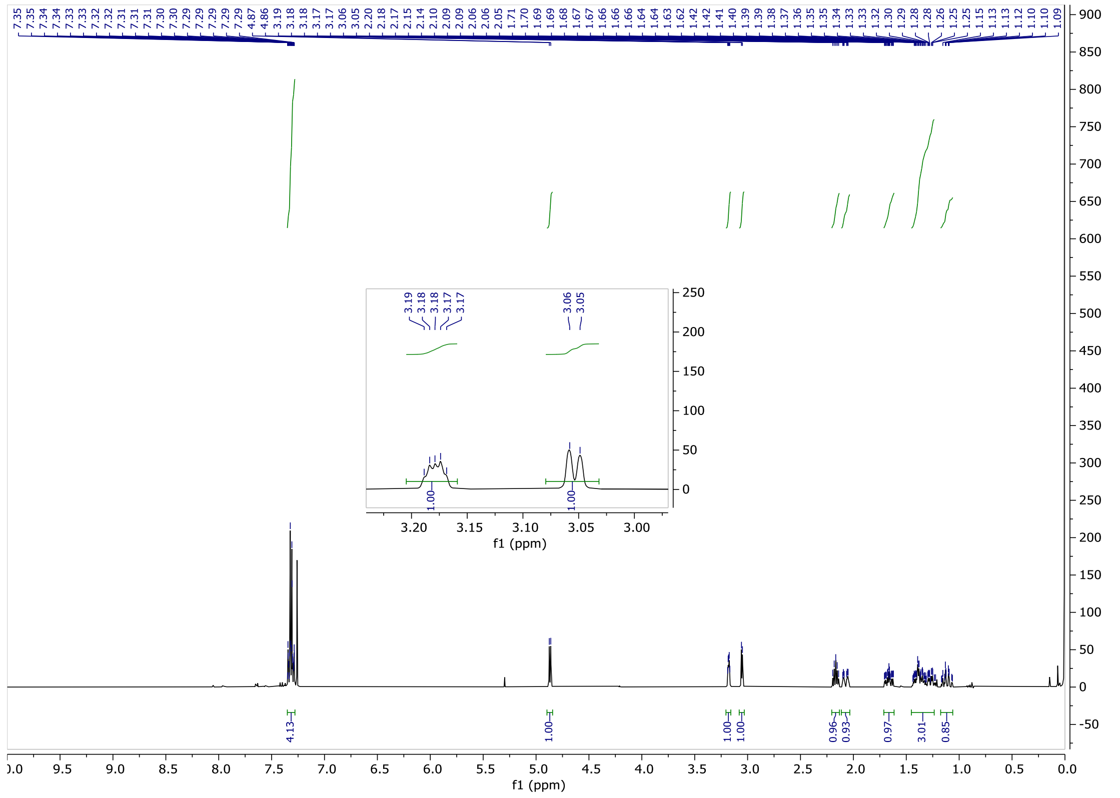

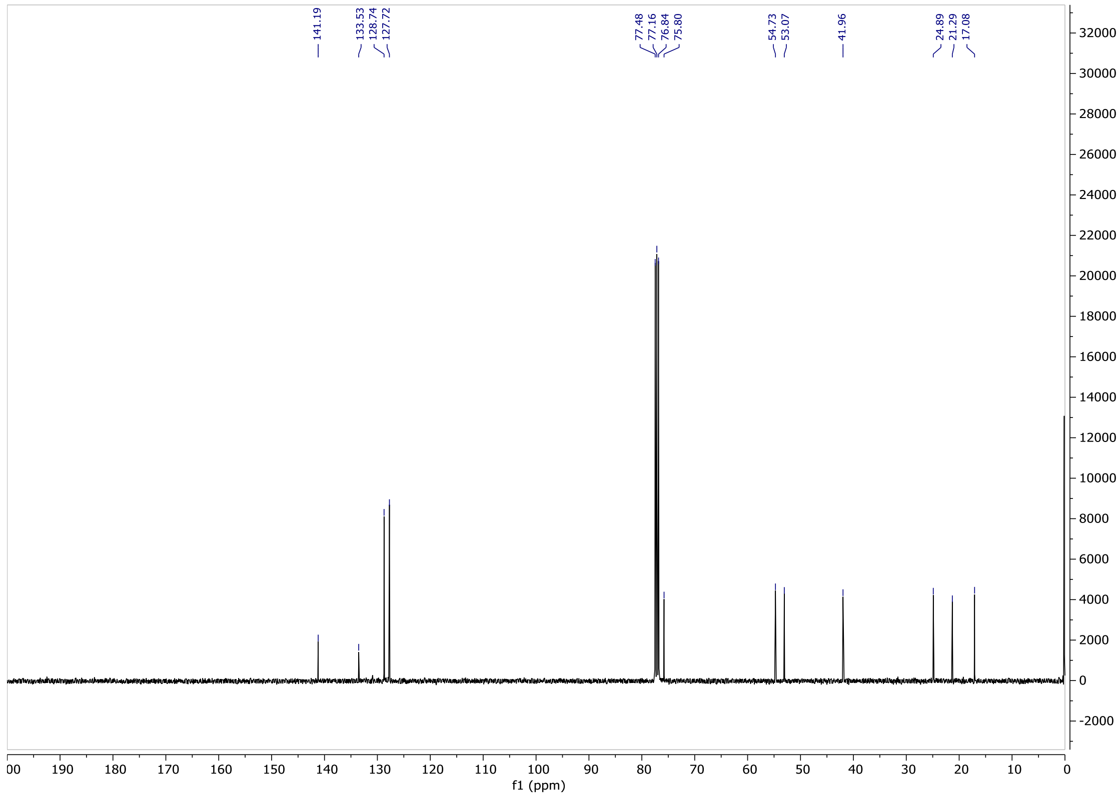

1f

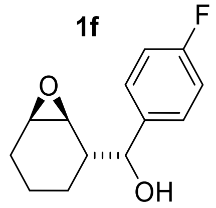

O
F
OH

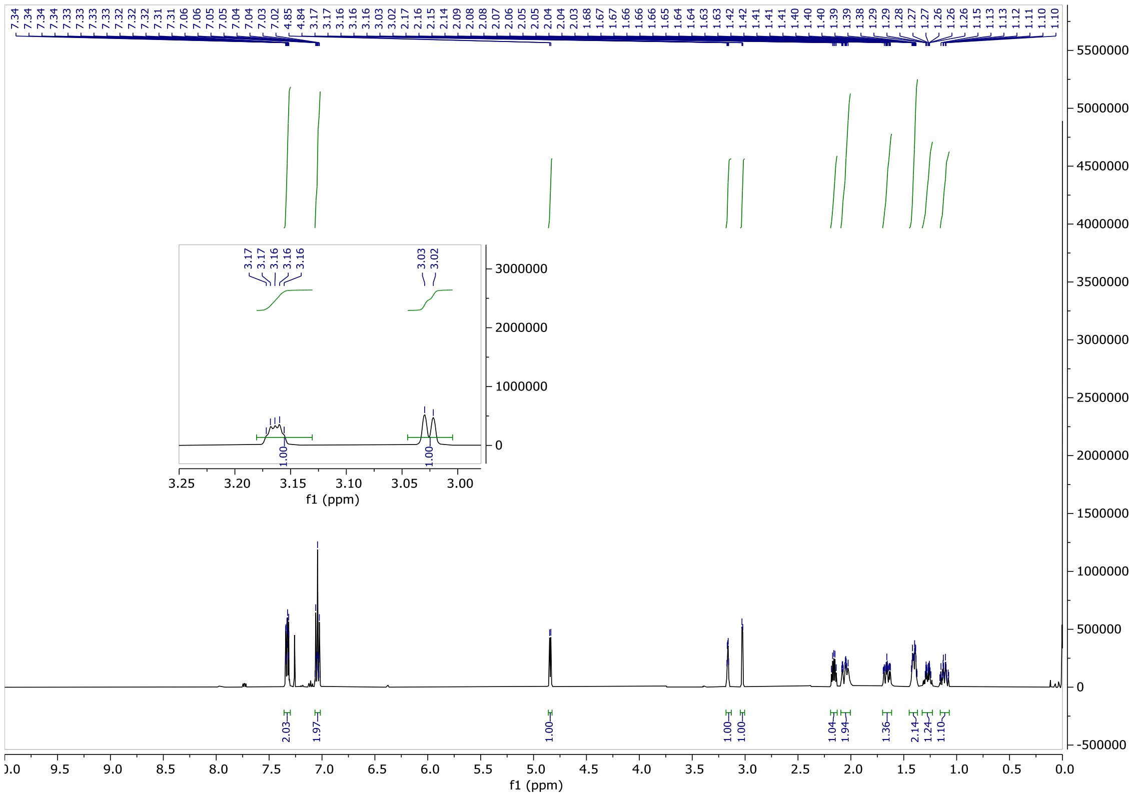

7.34 7.34 7.34 7.34 7.33 7.33 7.33 7.32 7.32 7.32 7.31 7.31 7.06 7.06 7.05 7.05 7.05 7.04 7.04 7.03 7.02 4.85 4.84 3.17 3.17 3.16 3.16 3.03 3.02 2.17 2.16 2.15 2.14 2.09 2.08 2.08 2.07 2.06 2.05 2.05 2.04 2.04 2.03 1.68 1.67 1.67 1.66 1.66 1.65 1.64 1.64 1.63 1.63 1.42 1.42 1.41 1.41 1.40 1.40 1.40 1.39 1.39 1.38 1.29 1.28 1.27 1.27 1.26 1.26 1.26 1.15 1.13 1.13 1.12 1.11 1.10
3.17 3.17 3.16 3.16 3.16
3.03 3.02
1.00 1.00
f1 (ppm)
2.03 1.97 1.00 1.00 1.00 1.04 1.94 1.36 2.14 1.24 1.10
f1 (ppm)

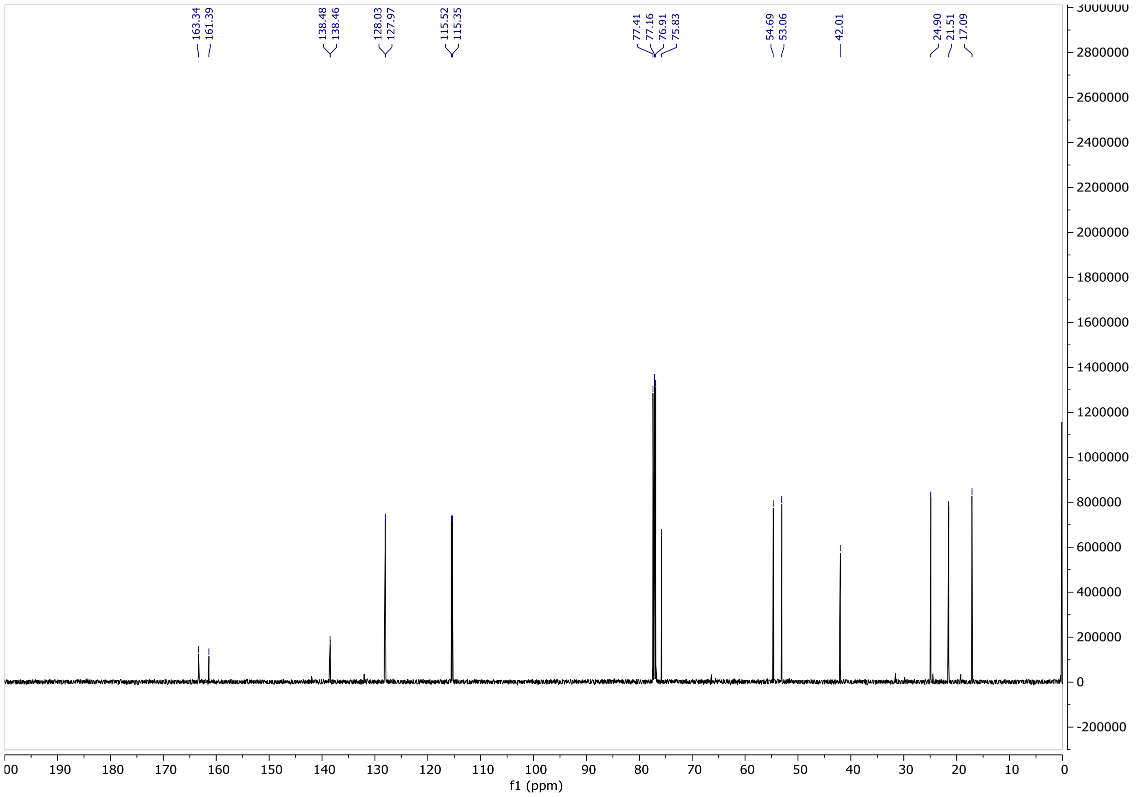

163.34 161.39 138.48 138.46 128.03 127.97 115.52 115.35 77.41 77.16 76.91 75.83 54.69 53.06 42.01 24.90 21.51 17.09
f1 (ppm)

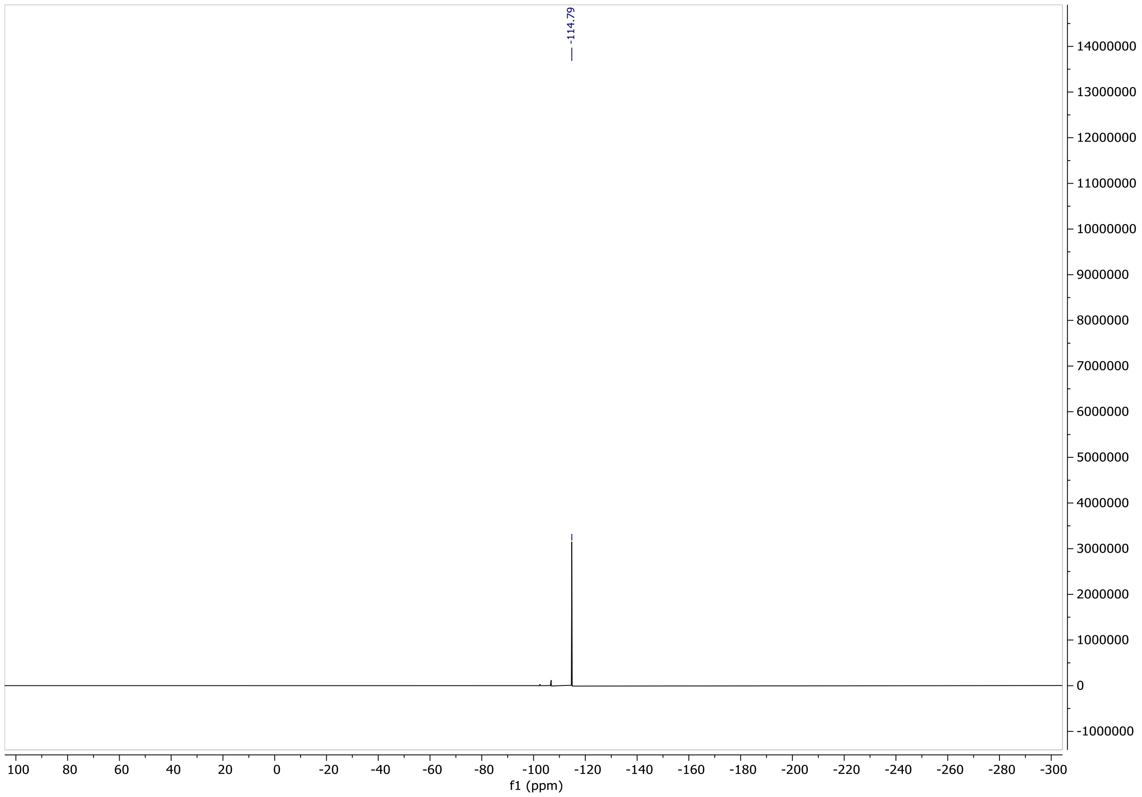

-114.79
14000000
13000000
12000000
11000000
10000000
9000000
8000000
7000000
6000000
5000000
4000000
3000000
2000000
1000000
0
-1000000
100 80 60 40 20 0 -20 -40 -60 -80 -100 -120 -140 -160 -180 -200 -220 -240 -260 -280 -300
f1 (ppm)

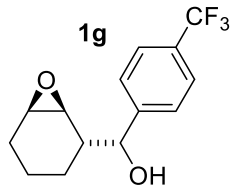

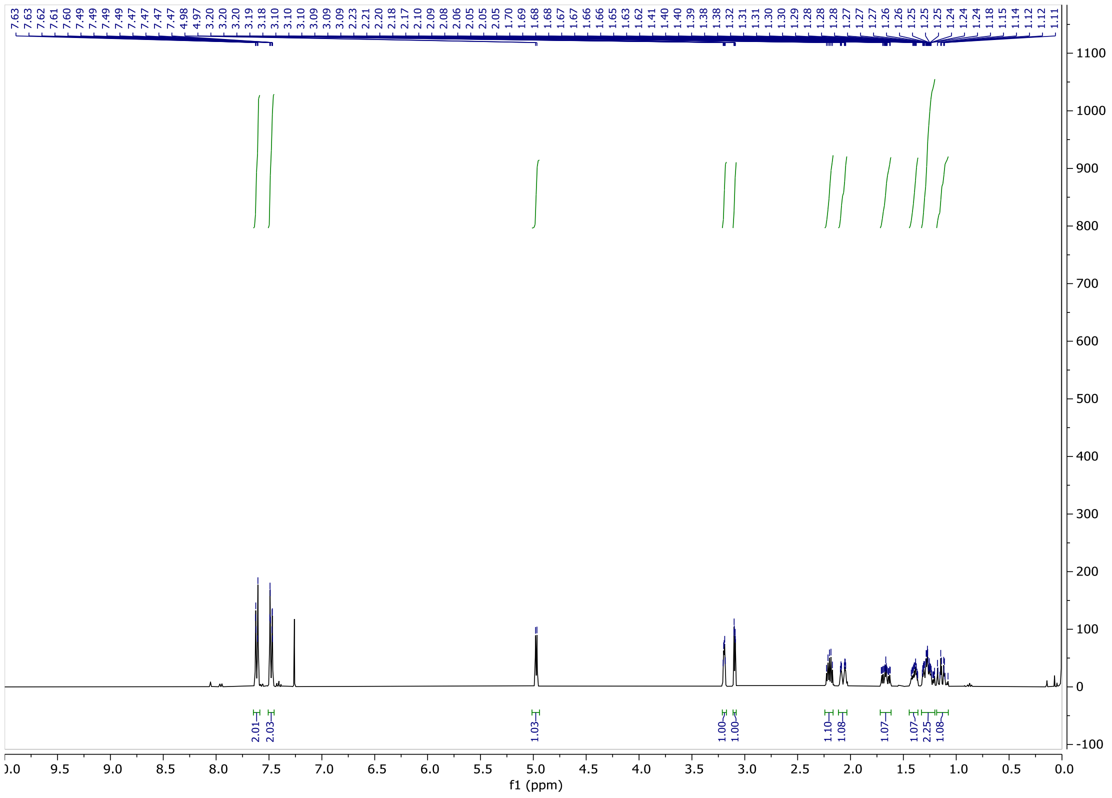

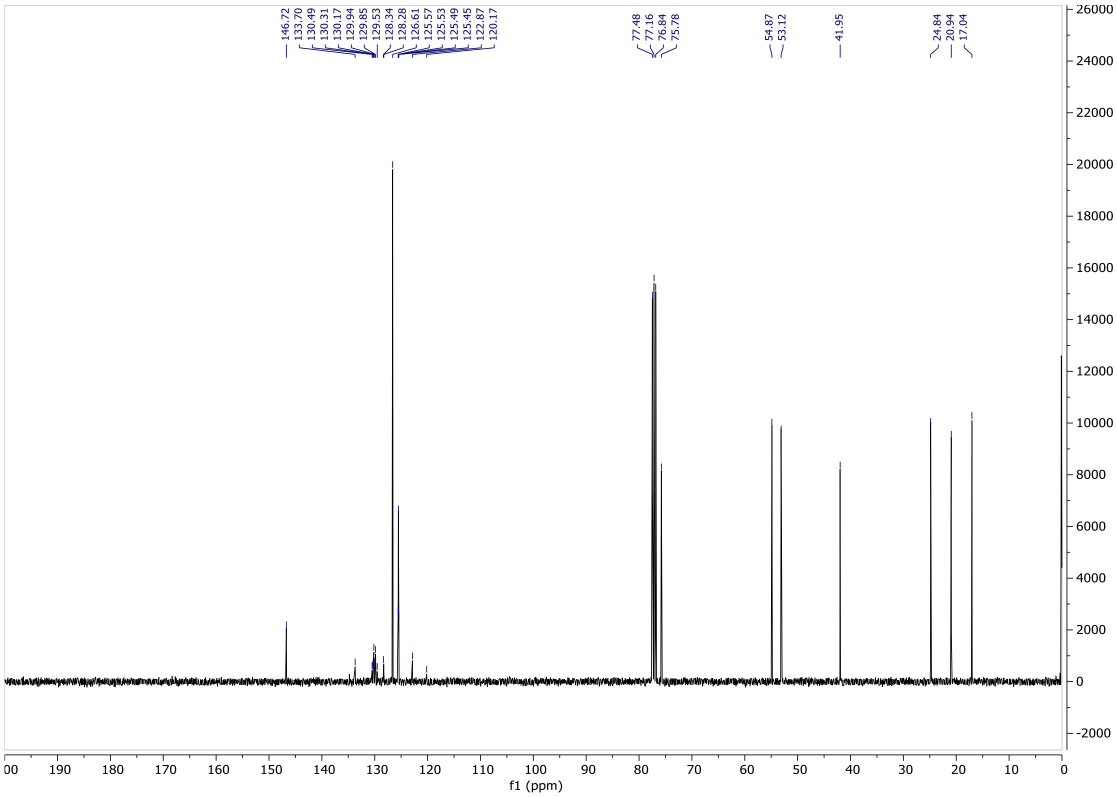
146.72
133.70
130.49
130.31
130.17
129.94
129.85
129.53
128.34
128.28
126.61
125.57
125.53
125.49
125.45
122.87
120.17
77.48
77.16
76.84
75.78
54.87
53.12
41.95
24.84
20.94
17.04
f1 (ppm)

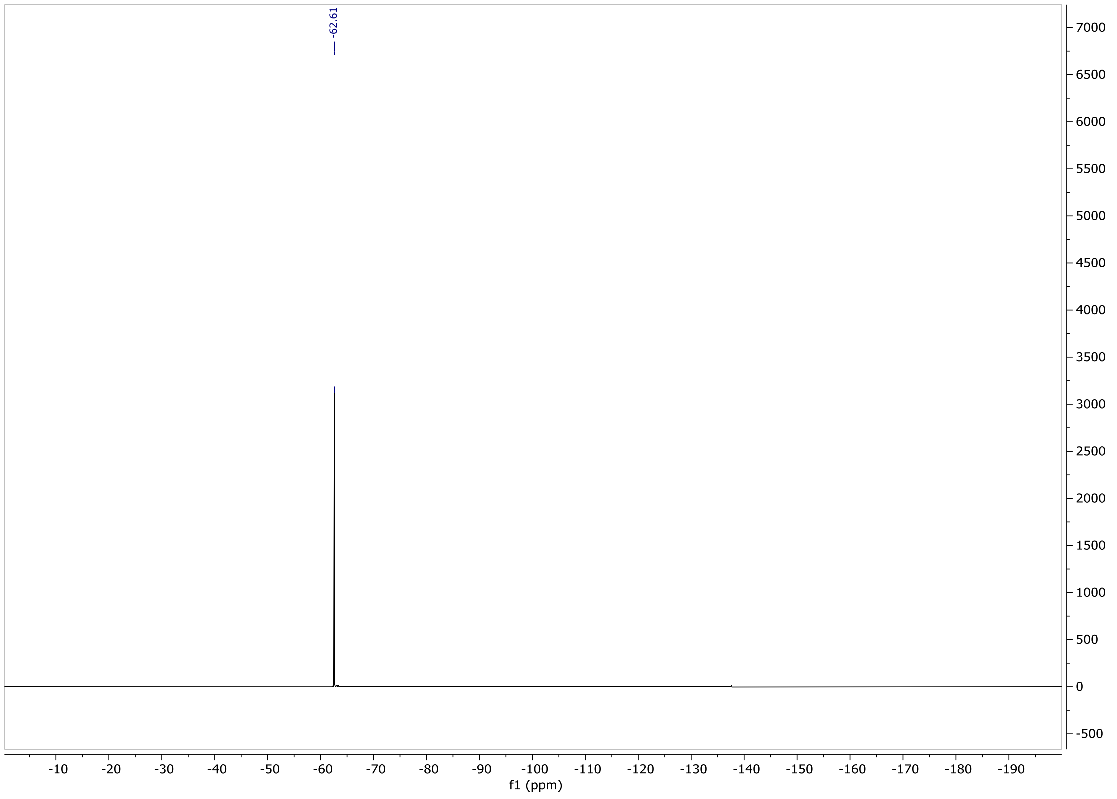
-62.61
f1 (ppm)

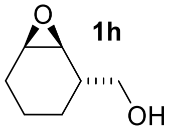
OH

1h

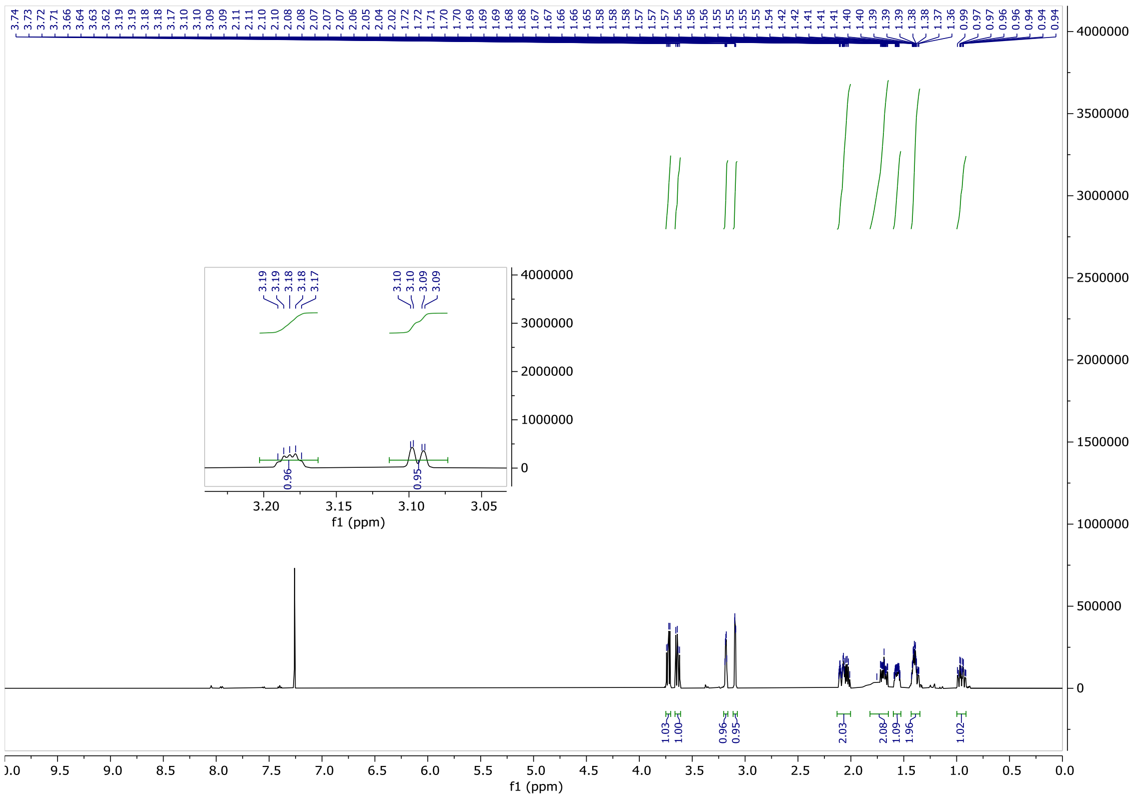
3.74 3.73 3.72 3.71 3.66 3.64 3.63 3.62 3.19 3.19 3.18 3.18 3.17 3.10 3.10 3.09 3.09 2.11 2.11 2.10 2.10 2.08 2.08 2.07 2.07 2.07 2.06 2.05 2.04 2.02 1.72 1.72 1.71 1.70 1.70 1.69 1.69 1.69 1.68 1.67 1.67 1.66 1.65 1.58 1.58 1.58 1.57 1.57 1.57 1.56 1.56 1.56 1.55 1.55 1.55 1.55 1.54 1.42 1.42 1.41 1.41 1.41 1.40 1.40 1.39 1.39 1.39 1.38 1.38 1.37 1.36 0.99 0.97 0.97 0.96 0.96 0.94 0.94 0.94
1.03 1.00 0.96 0.95 2.03 2.08 1.09 1.96 1.02
f1 (ppm)
3.19 3.19 3.18 3.18 3.17 3.10 3.10 3.09 3.09
0.96 0.95
f1 (ppm)

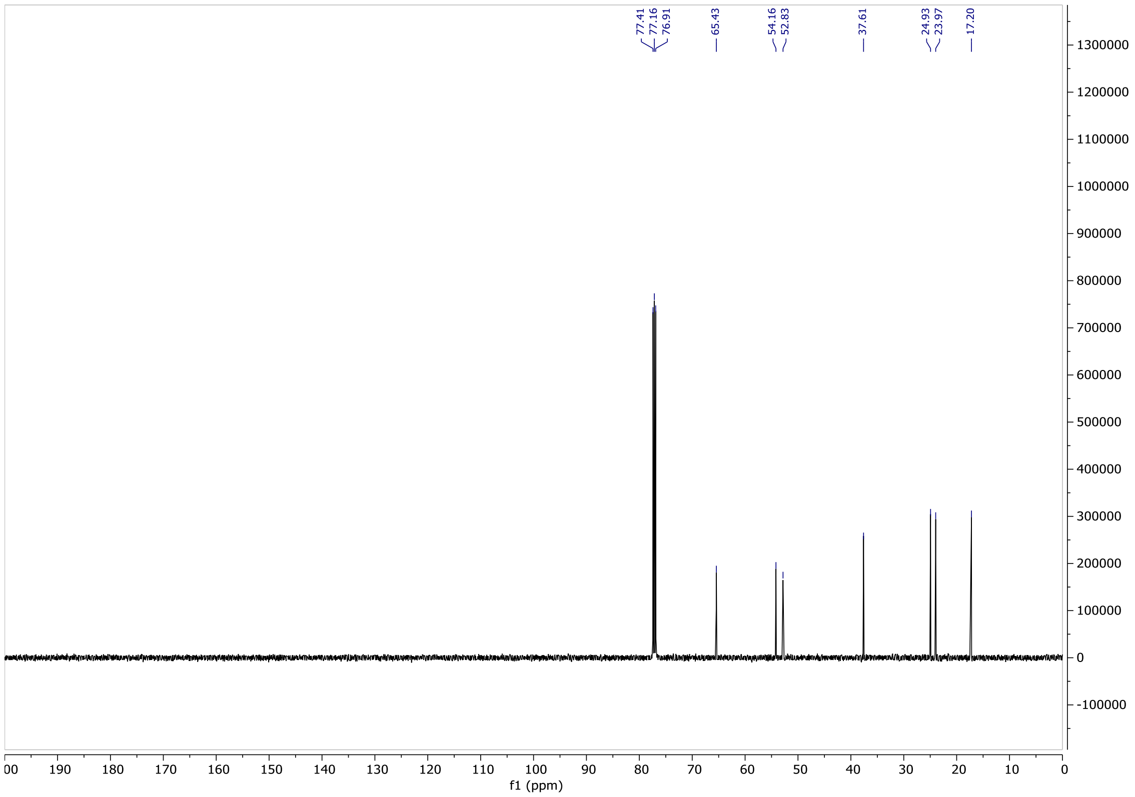
77.41 77.16 76.91 65.43 54.16 52.83 37.61 24.93 23.97 17.20
f1 (ppm)

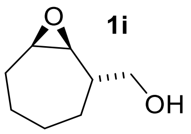

1i

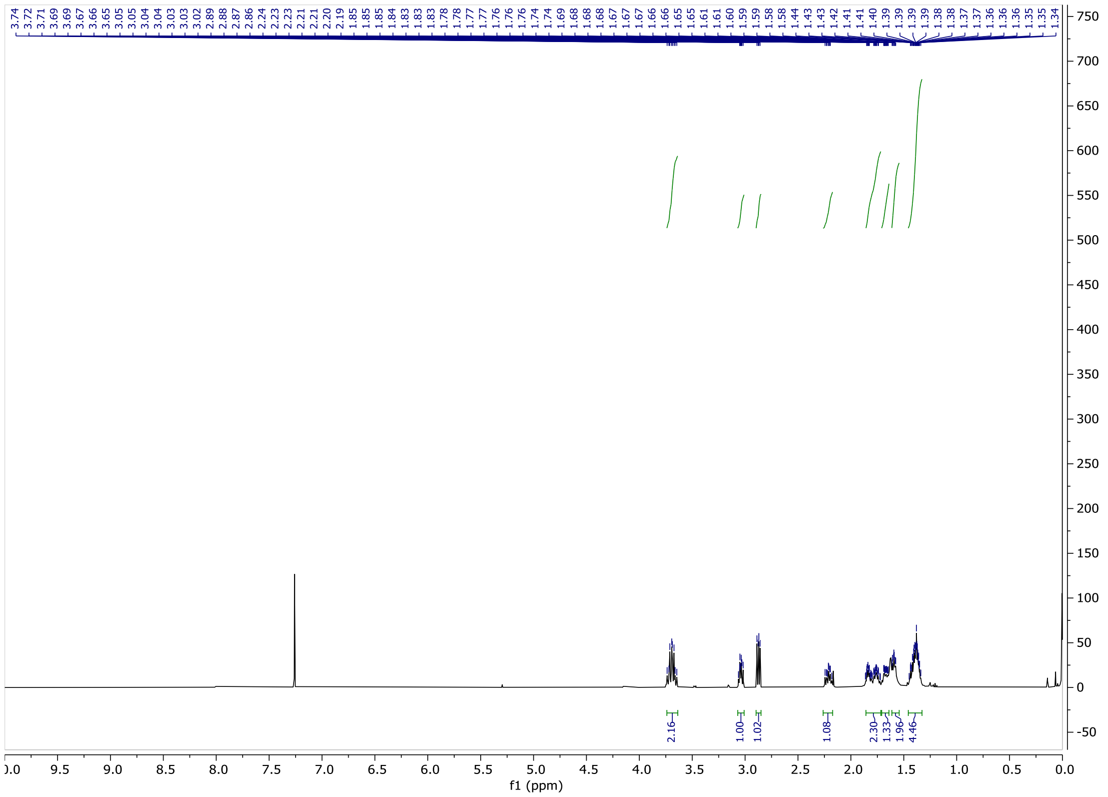
3.74
3.72
3.71
3.69
3.69
3.67
3.66
3.65
3.05
3.05
3.04
3.04
3.03
3.03
3.02
2.89
2.88
2.87
2.86
2.24
2.23
2.23
2.21
2.21
2.20
2.19
1.85
1.85
1.85
1.84
1.83
1.83
1.83
1.78
1.78
1.77
1.77
1.76
1.76
1.74
1.74
1.69
1.68
1.68
1.68
1.67
1.67
1.67
1.66
1.66
1.65
1.65
1.61
1.61
1.60
1.59
1.59
1.58
1.58
1.44
1.43
1.43
1.42
1.41
1.41
1.40
1.39
1.39
1.39
1.39
1.38
1.38
1.37
1.37
1.36
1.36
1.35
1.35
1.34
2.16
1.00
1.02
1.08
2.30
1.33
1.96
4.46
f1 (ppm)

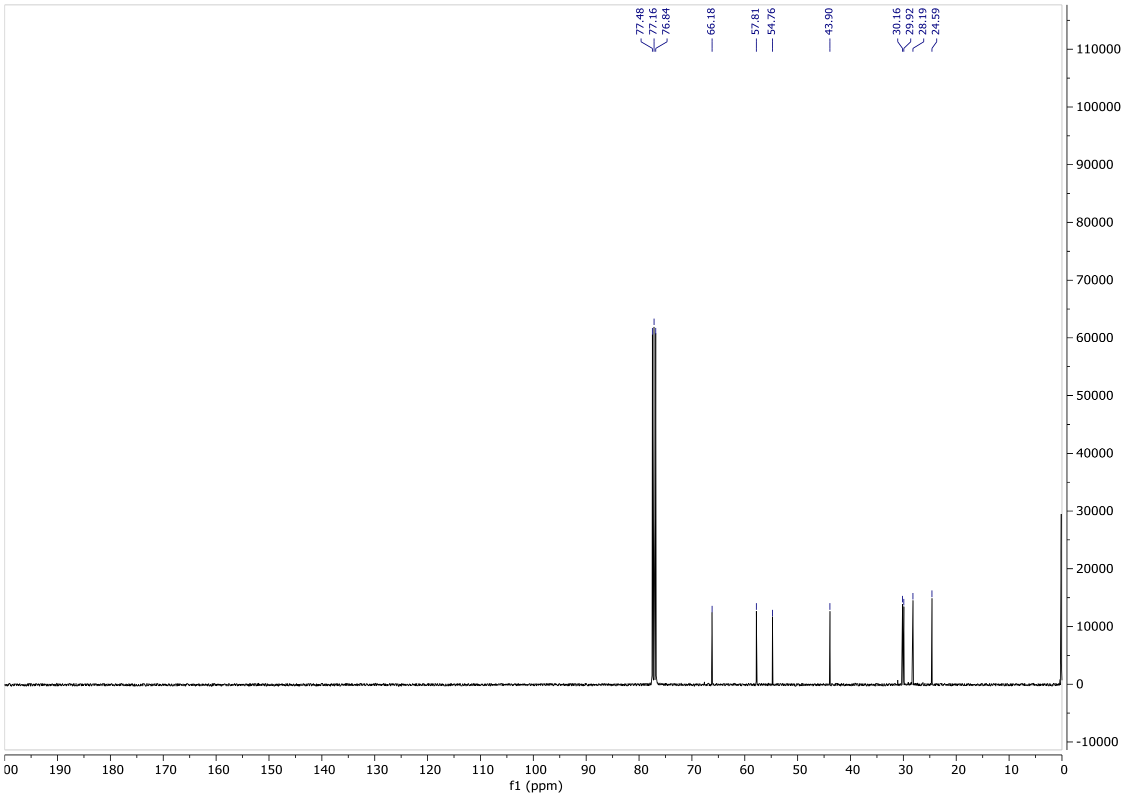
77.48
77.16
76.84
66.18
57.81
54.76
43.90
30.16
29.92
28.19
24.59
f1 (ppm)

1j

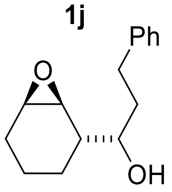

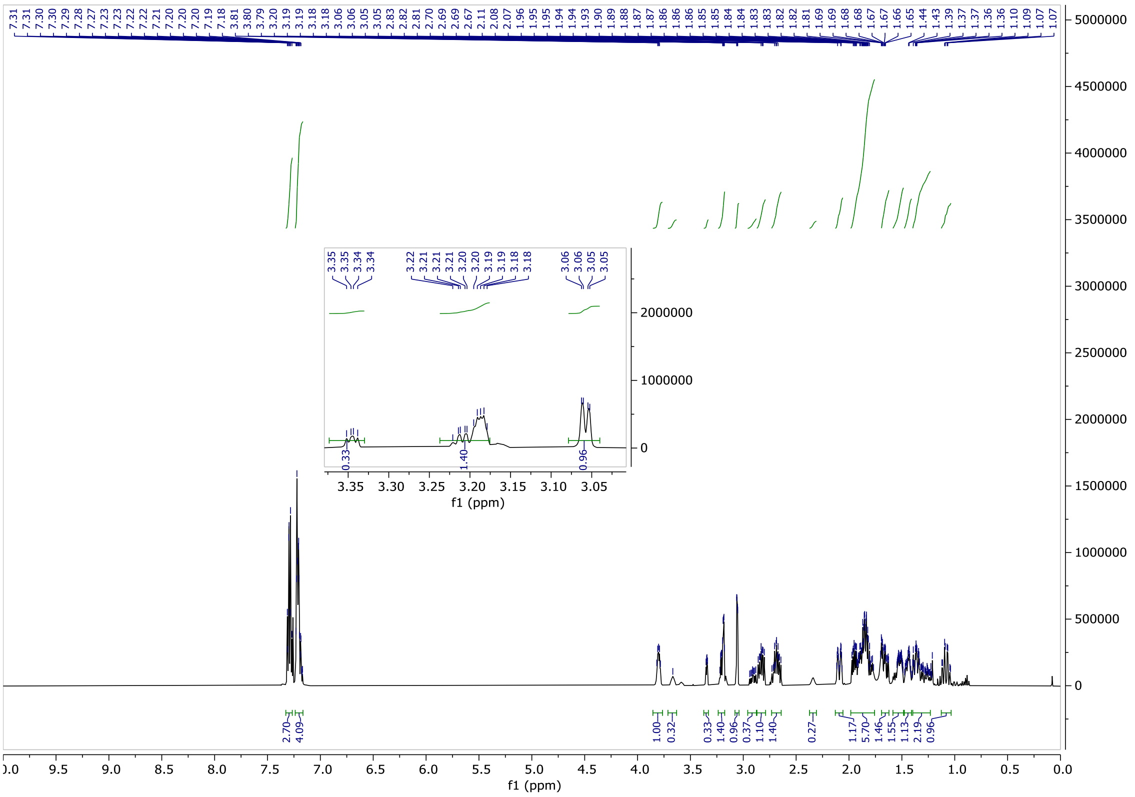

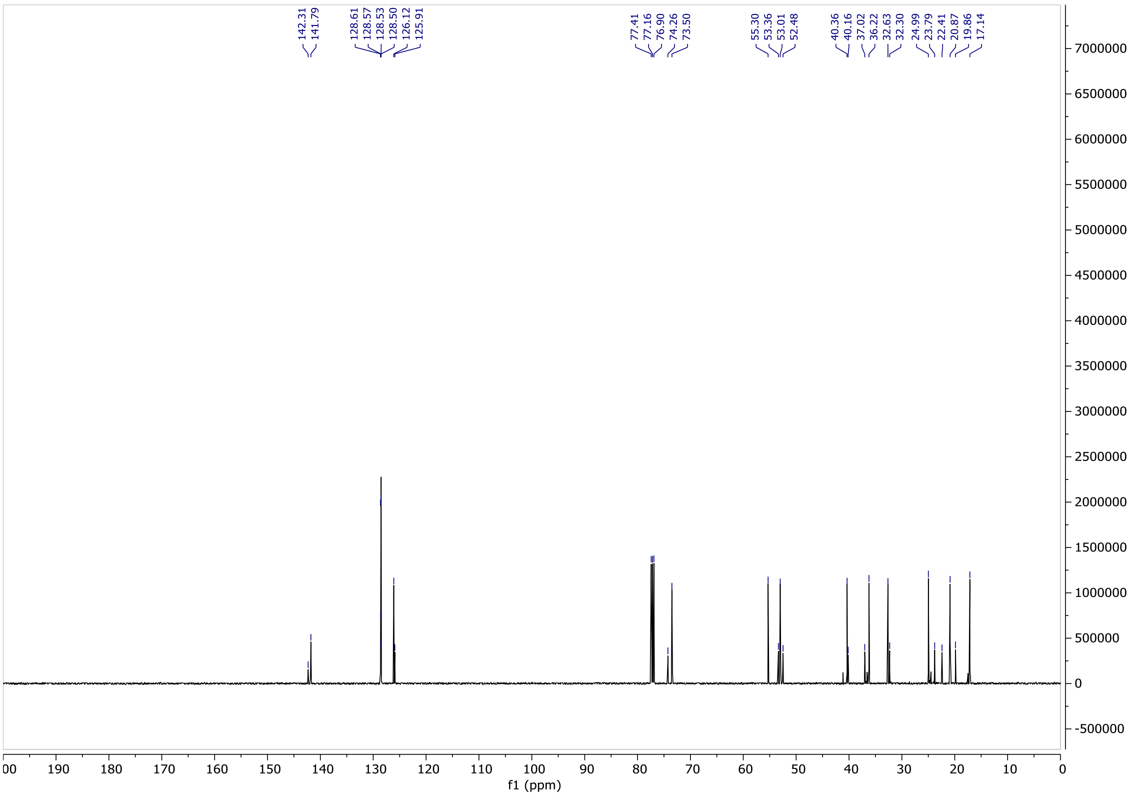

1k

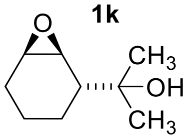

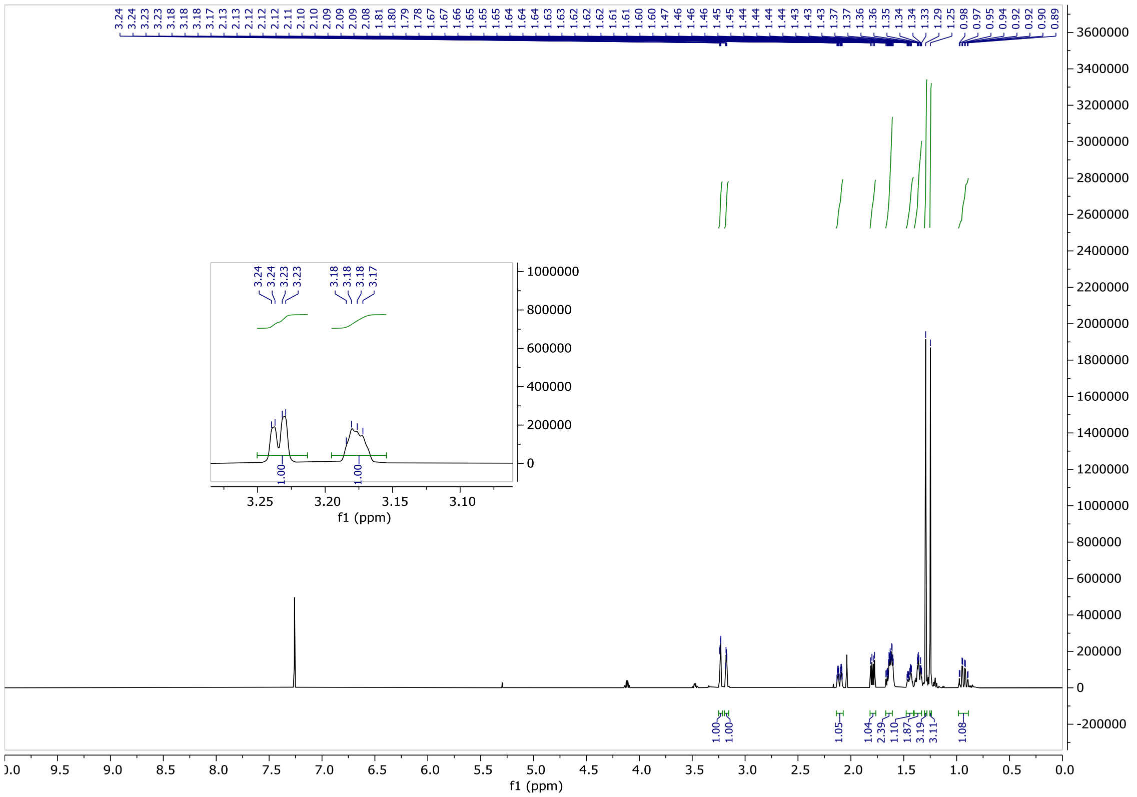

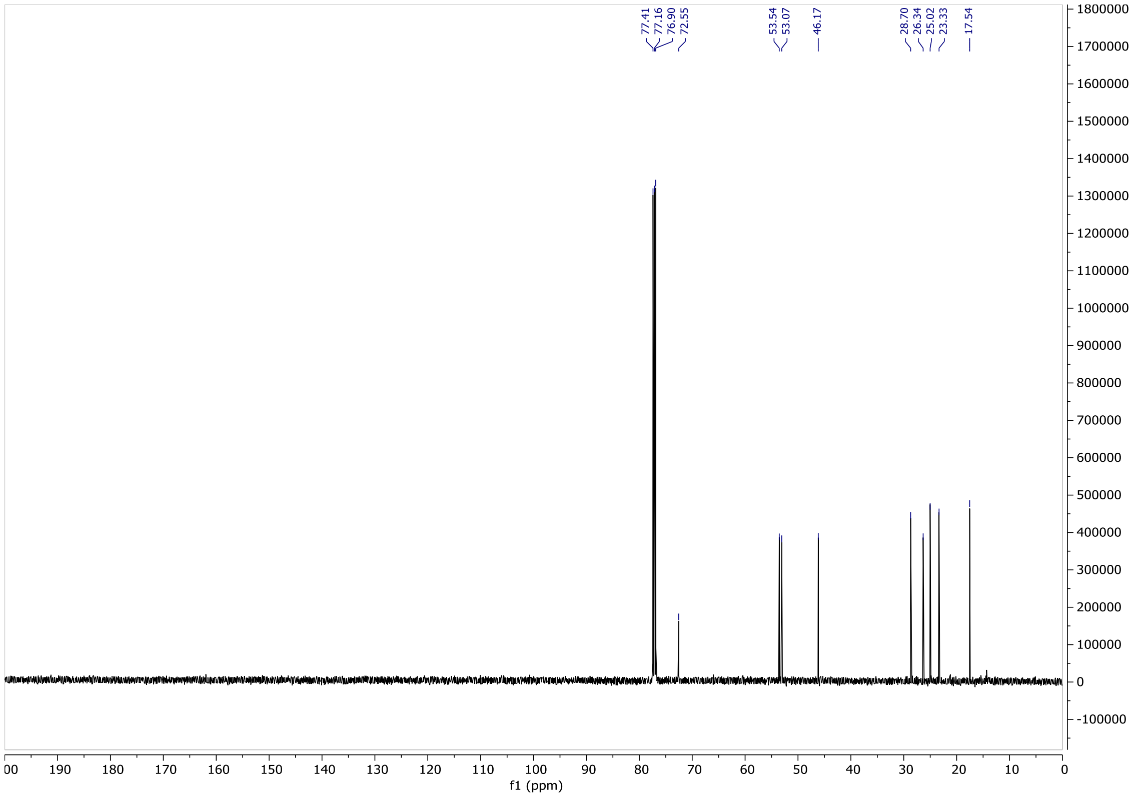

1l

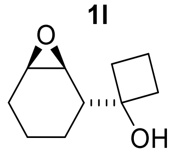

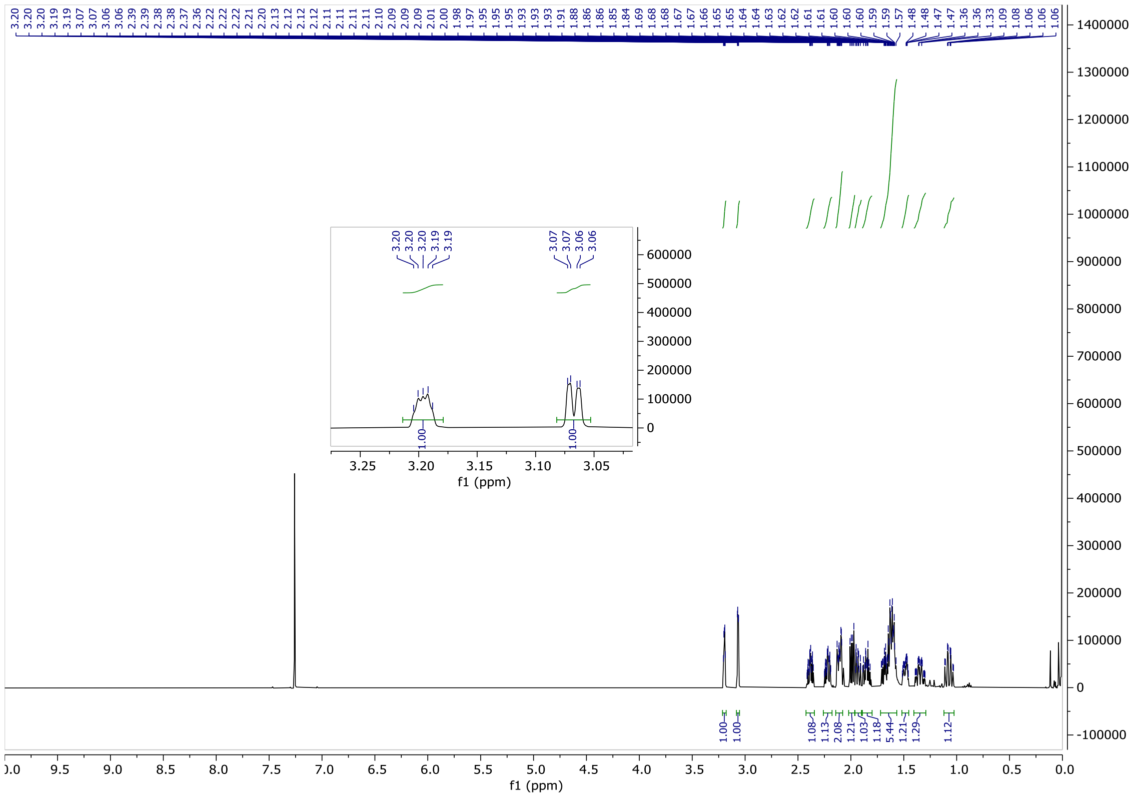
1.00
1.00
1.08
1.13
2.08
1.21
1.03
1.18
5.44
1.21
1.29
1.12
f1 (ppm)

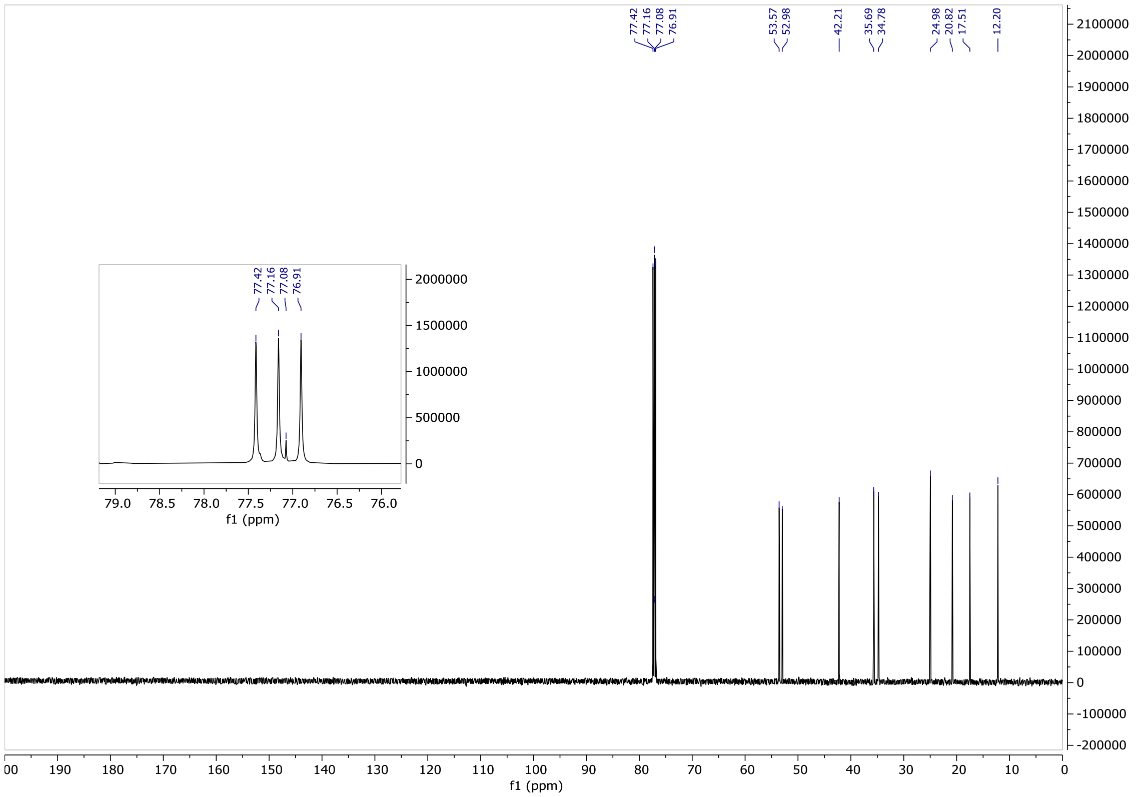
77.42
77.16
77.08
76.91
53.57
52.98
42.21
35.69
34.78
24.98
20.82
17.51
12.20
f1 (ppm)

1m

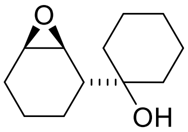

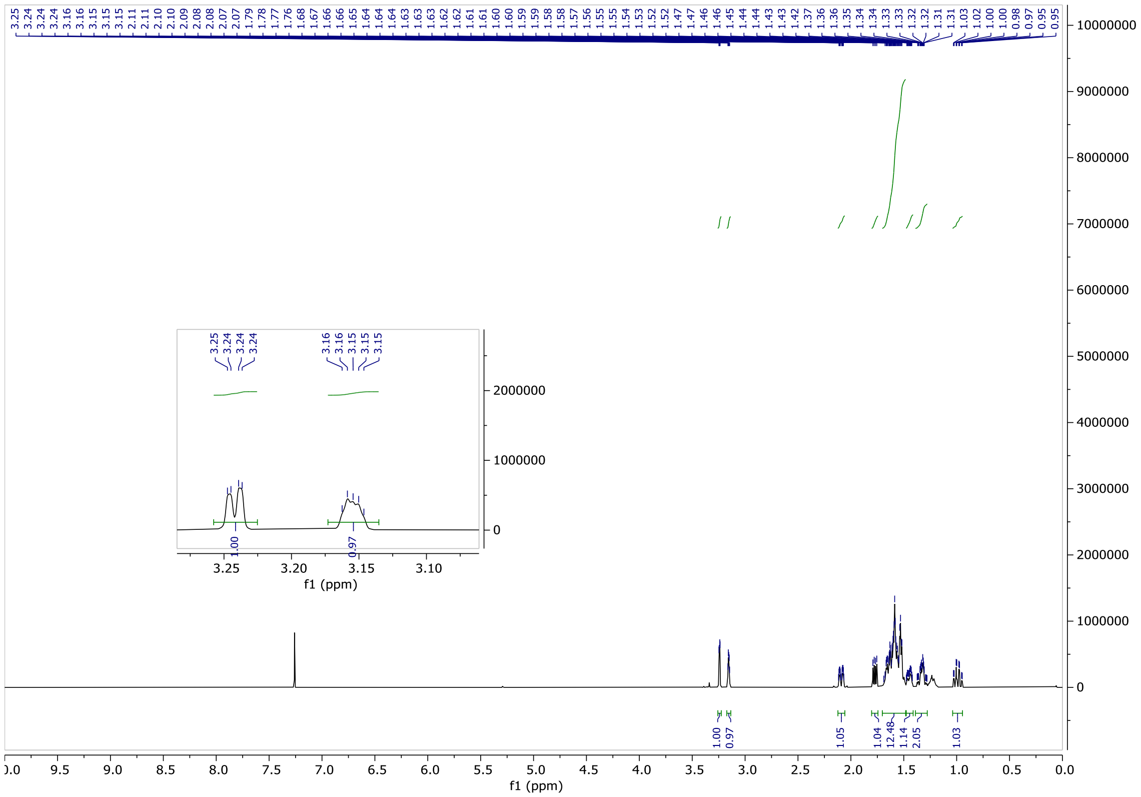

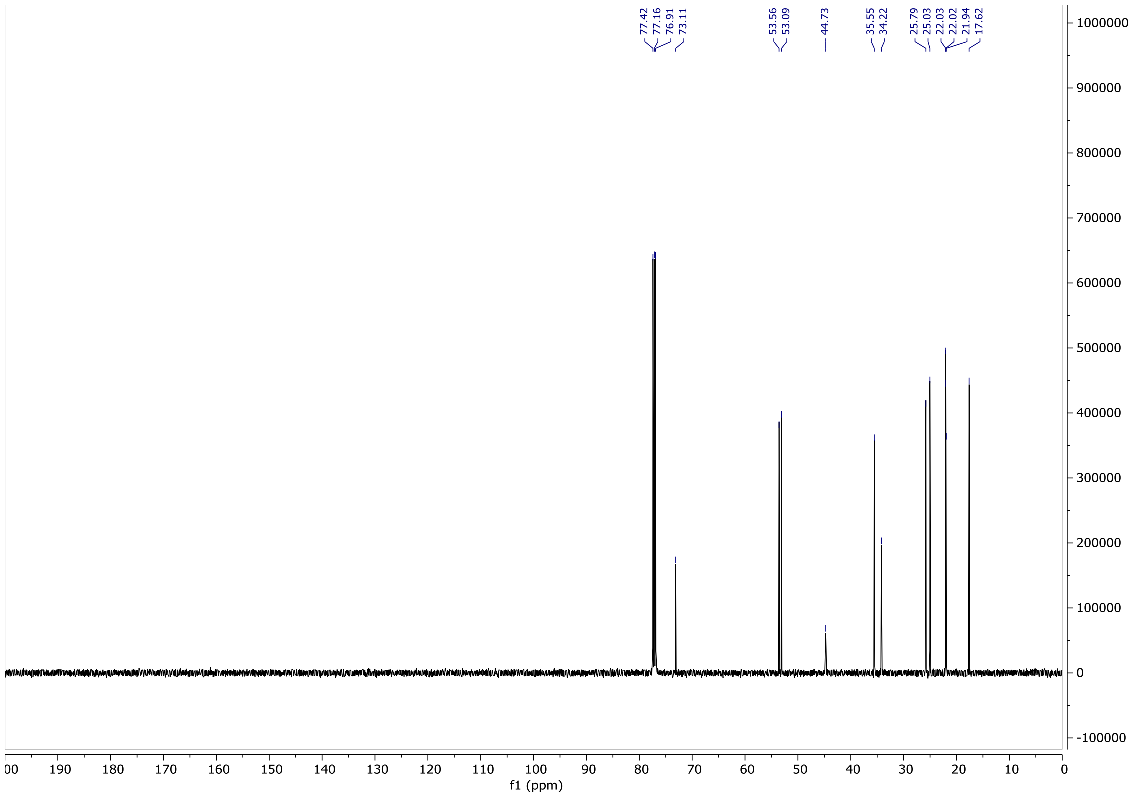

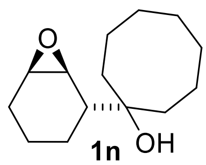
O
OH

1n

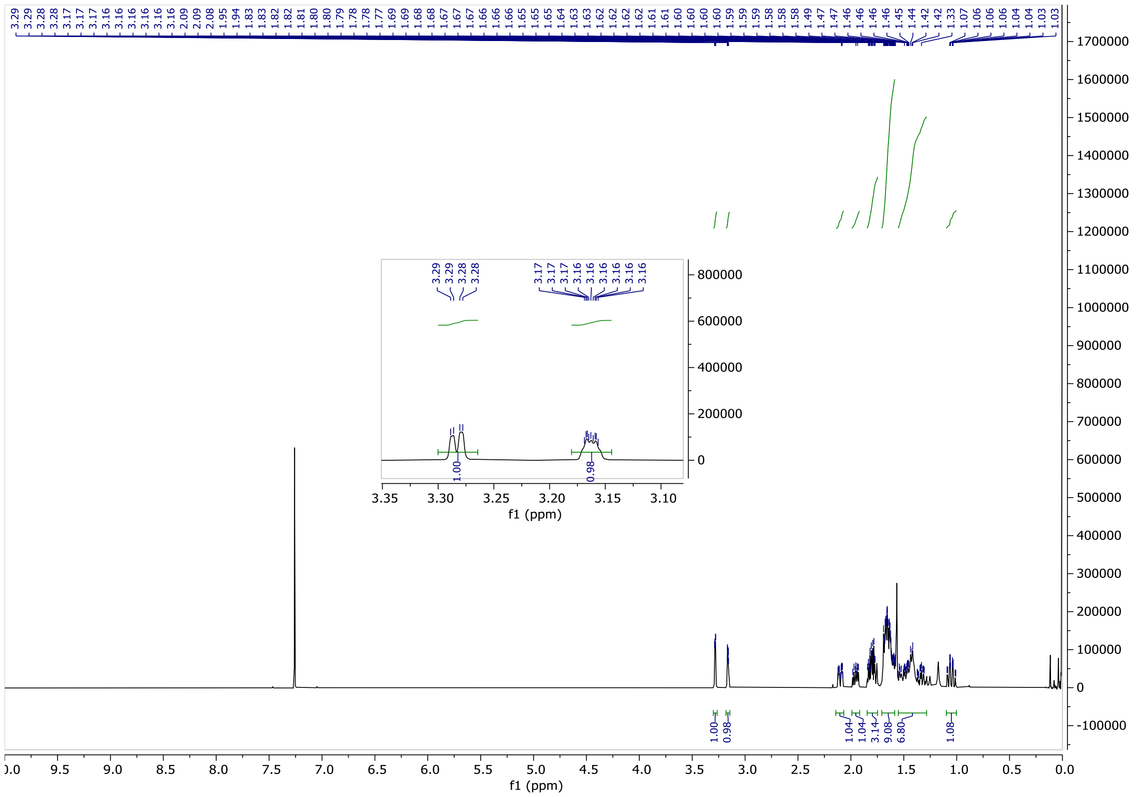
3.29 3.29 3.28 3.28 3.17 3.17 3.16 3.16 3.16 3.16 2.09 2.08 1.95 1.94 1.83 1.83 1.82 1.81 1.80 1.79 1.78 1.77 1.69 1.69 1.68 1.67 1.67 1.66 1.66 1.65 1.65 1.64 1.63 1.63 1.62 1.62 1.62 1.61 1.61 1.60 1.60 1.60 1.59 1.59 1.59 1.58 1.58 1.58 1.49 1.47 1.47 1.46 1.46 1.46 1.45 1.44 1.42 1.42 1.33 1.07 1.06 1.06 1.04 1.04 1.03 1.03
1.00
0.98
1.04
1.04
3.14
9.08
6.80
1.08
f1 (ppm)

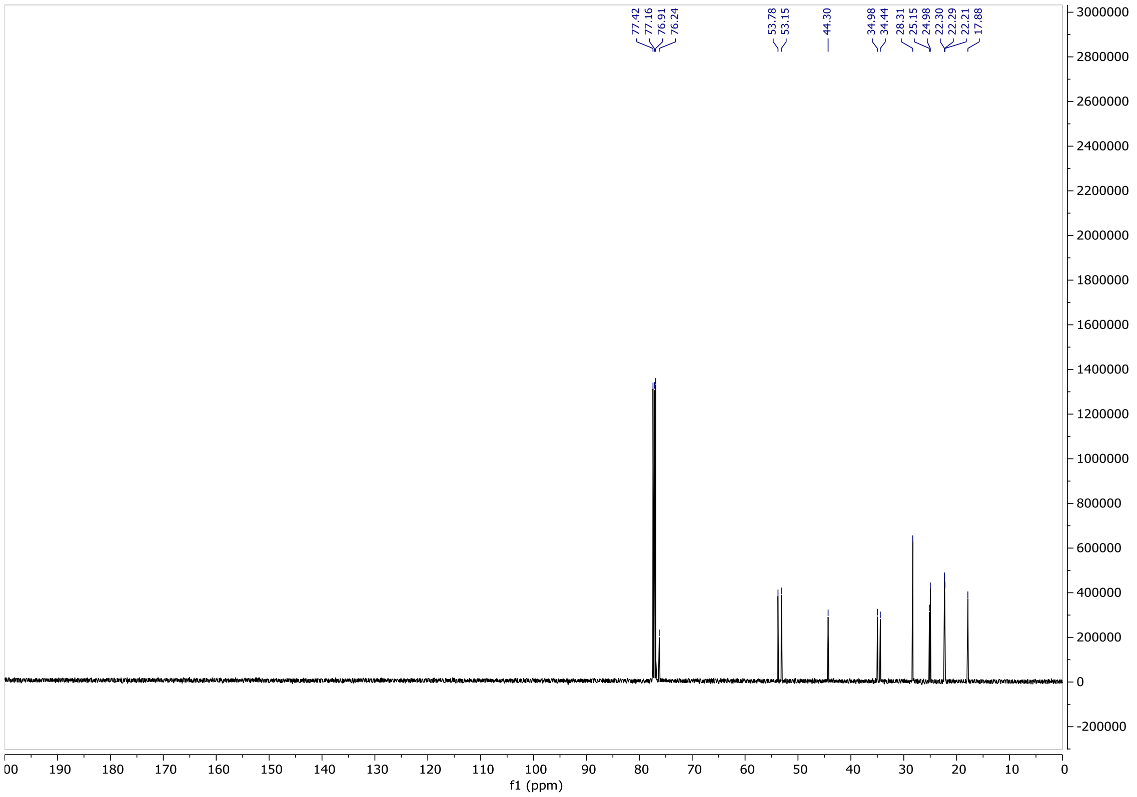
77.42 77.16 76.91 76.24
53.78 53.15
44.30
34.98 34.44 28.31 25.15 24.98 22.30 22.29 22.21 17.88
f1 (ppm)

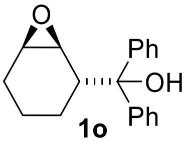
O
Ph
OH
Ph

1o

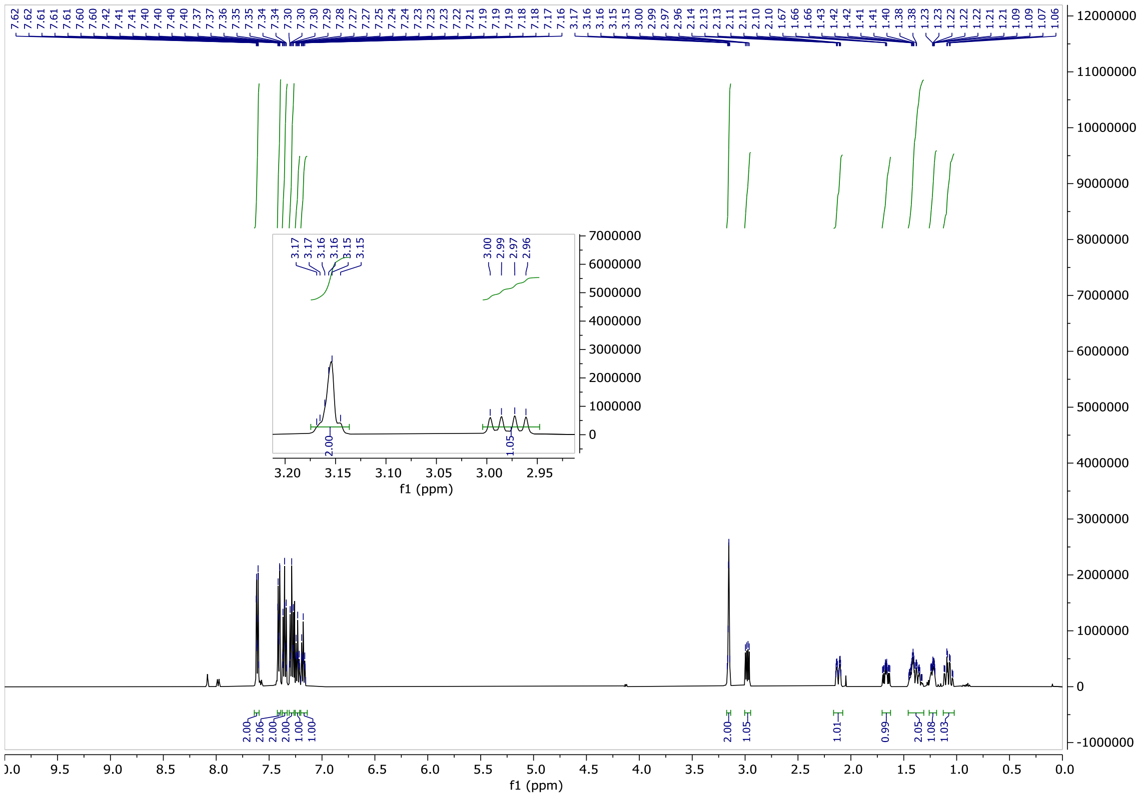
7.62
7.62
7.61
7.61
7.61
7.60
7.60
7.42
7.41
7.41
7.40
7.40
7.40
7.40
7.37
7.37
7.36
7.35
7.35
7.34
7.34
7.30
7.30
7.30
7.29
7.28
7.27
7.27
7.25
7.24
7.24
7.23
7.23
7.22
7.21
7.19
7.19
7.19
7.18
7.18
7.17
7.16
3.17
3.16
3.16
3.15
3.15
3.00
2.99
2.97
2.96
2.14
2.13
2.13
2.11
2.11
2.10
2.10
1.67
1.66
1.66
1.43
1.42
1.42
1.41
1.41
1.40
1.38
1.38
1.23
1.23
1.22
1.22
1.21
1.21
1.09
1.09
1.07
1.06
3.17
3.17
3.16
3.16
3.15
3.15
3.00
2.99
2.97
2.96
2.00
1.05
3.20
3.15
3.10
3.05
3.00
2.95
f1 (ppm)
2.00
2.06
2.00
2.00
1.00
1.00
2.00
1.05
1.01
0.99
2.05
1.08
1.03
f1 (ppm)

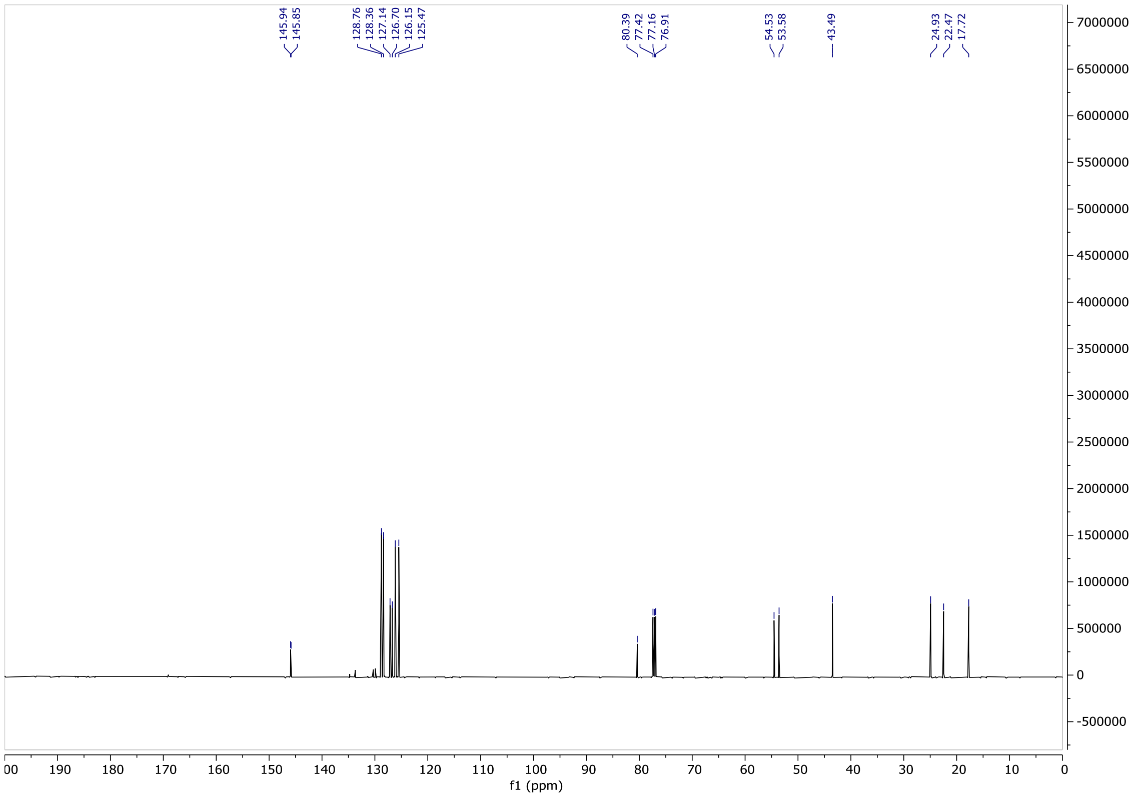
145.94
145.85
128.76
128.36
127.14
126.70
126.15
125.47
80.39
77.42
77.16
76.91
54.53
53.58
43.49
24.93
22.47
17.72
f1 (ppm)

1p

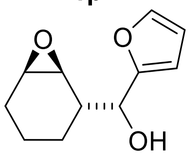

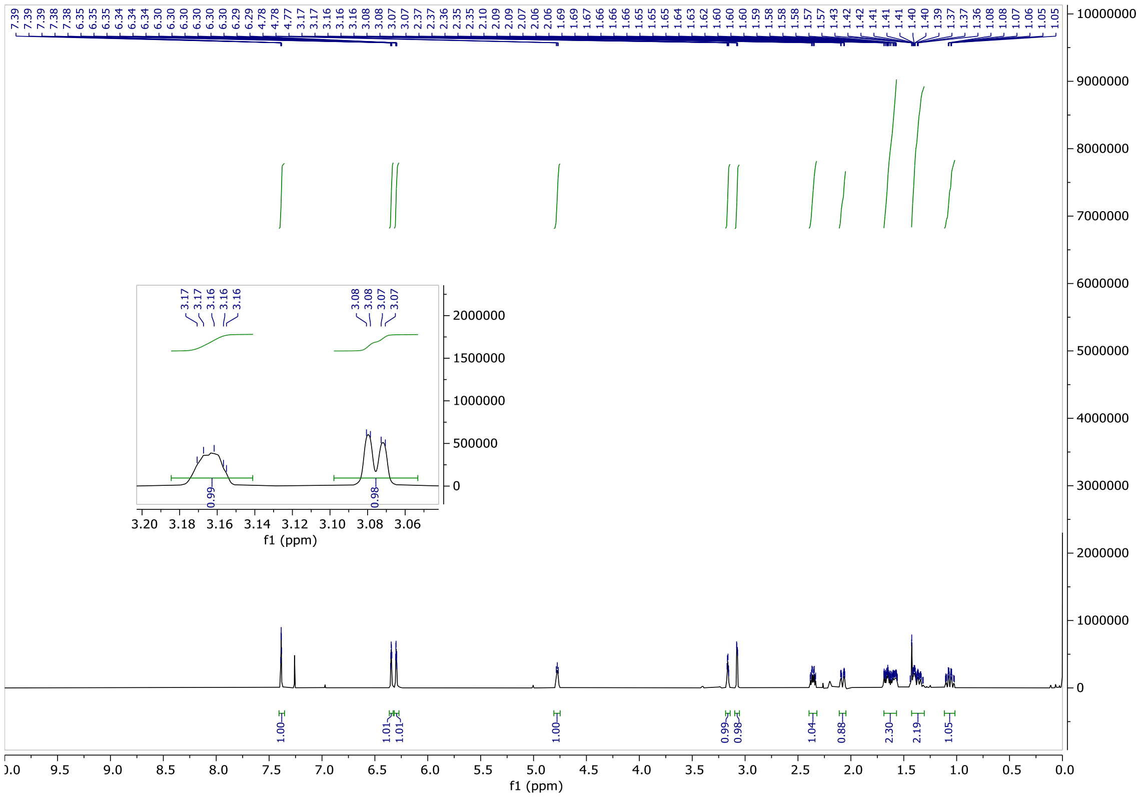

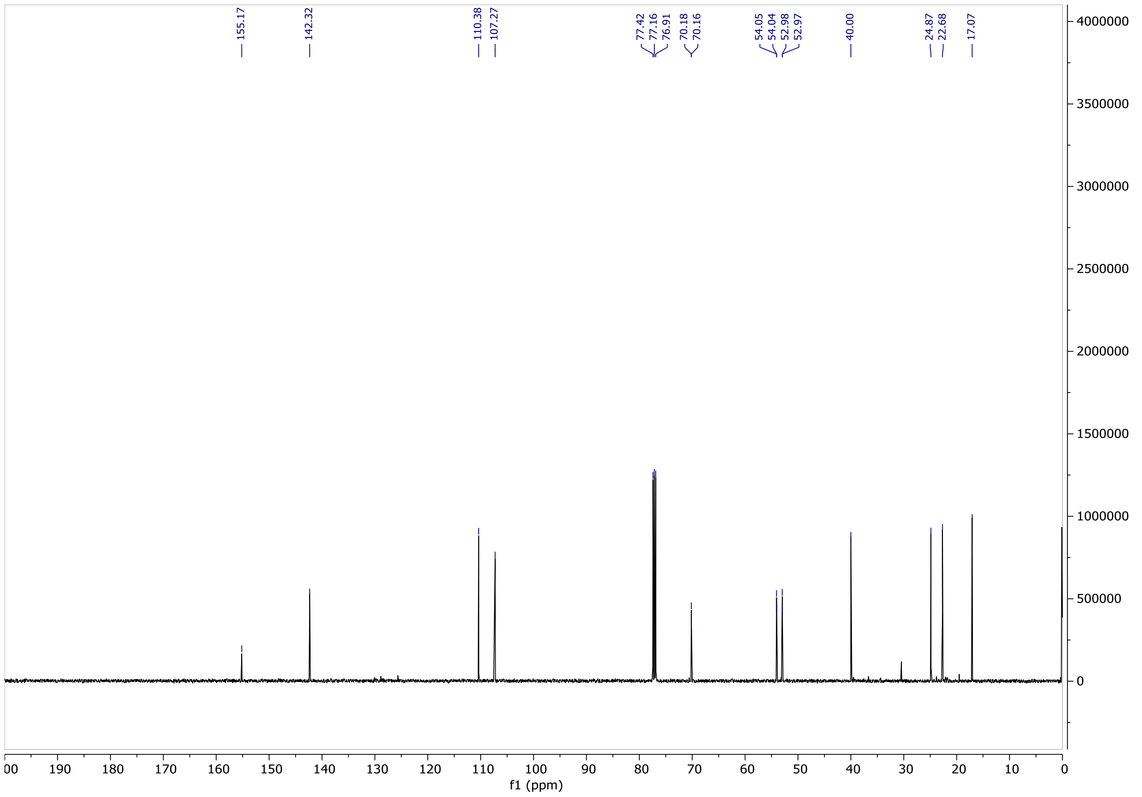

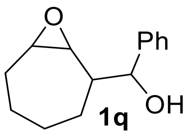

1q

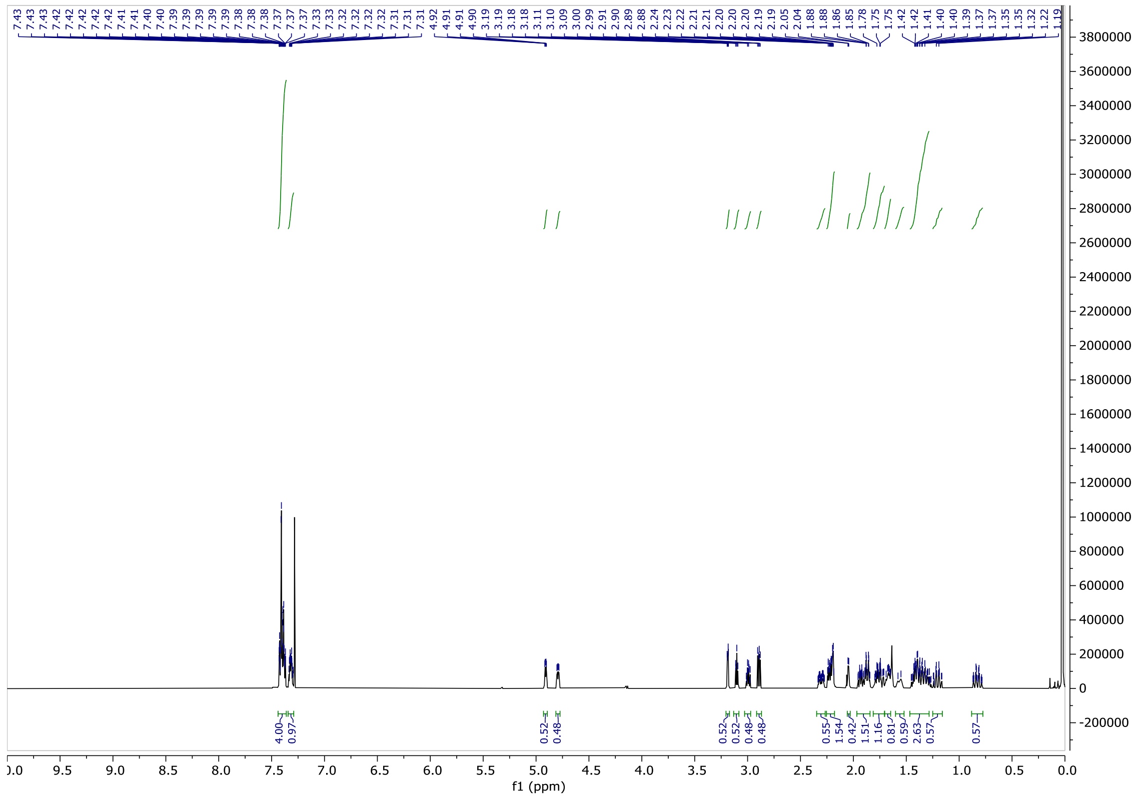

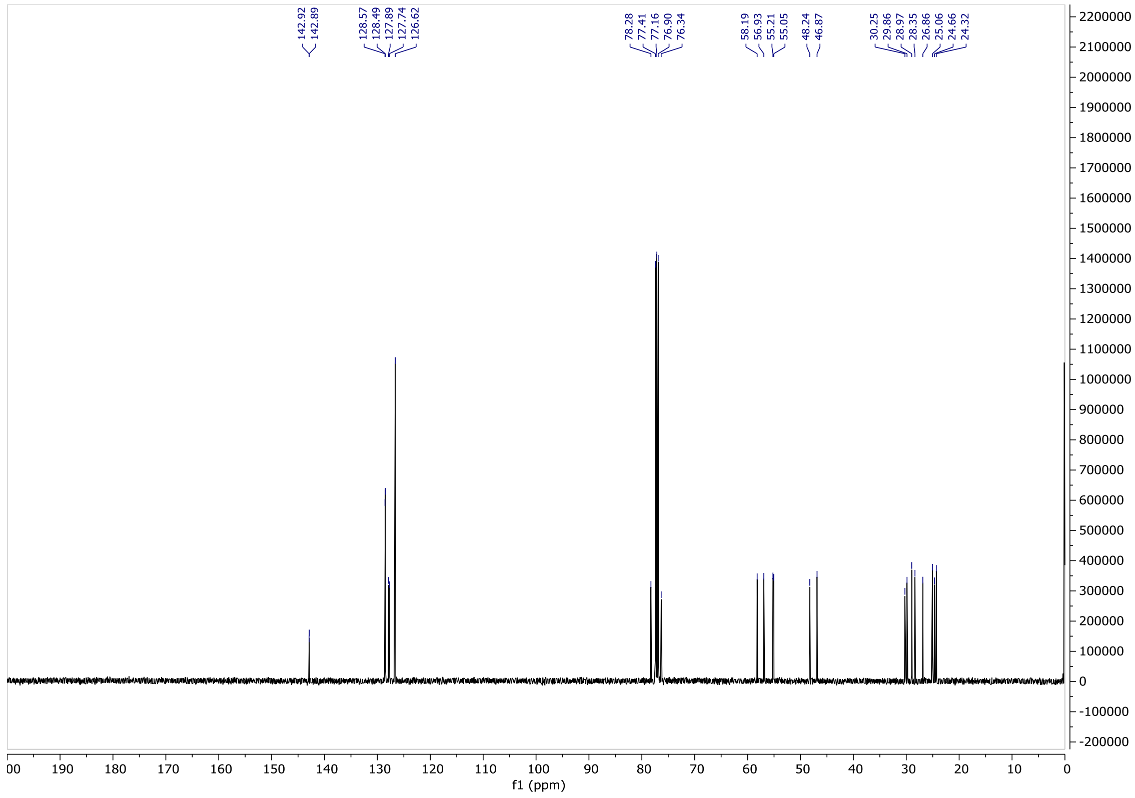

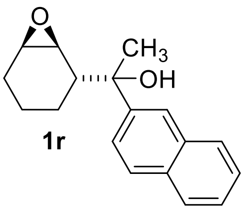
CH3
OH
1r

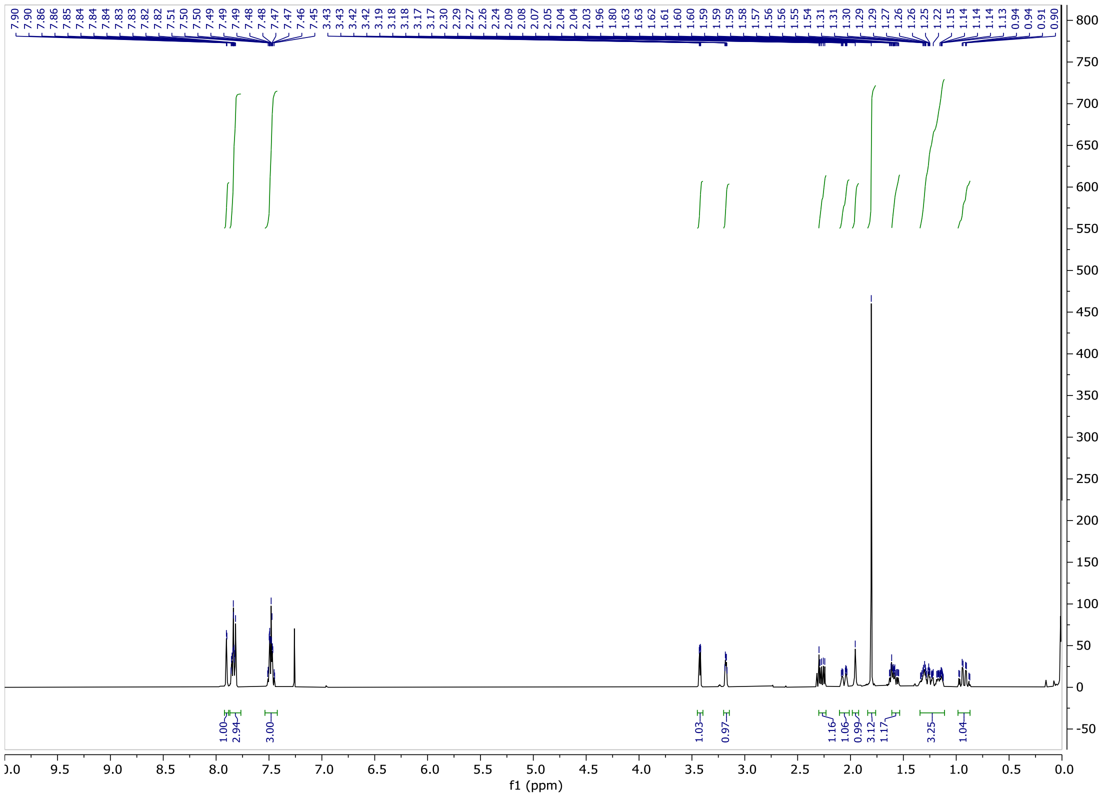
7.90 7.90 7.86 7.85 7.84 7.84 7.84 7.83 7.83 7.82 7.82 7.51 7.50 7.50 7.49 7.49 7.49 7.48 7.48 7.47 7.47 7.46 7.45 3.43 3.43 3.42 3.42 3.19 3.18 3.18 3.17 3.17 2.30 2.29 2.27 2.26 2.24 2.09 2.08 2.07 2.05 2.04 2.04 2.03 1.96 1.80 1.63 1.63 1.62 1.61 1.60 1.59 1.59 1.59 1.58 1.57 1.56 1.56 1.55 1.54 1.31 1.31 1.30 1.29 1.29 1.27 1.26 1.25 1.22 1.15 1.14 1.14 1.14 1.13 0.94 0.94 0.91 0.90
1.00 2.94 3.00 1.03 0.97 1.16 1.06 0.99 3.12 1.17 3.25 1.04
f1 (ppm)

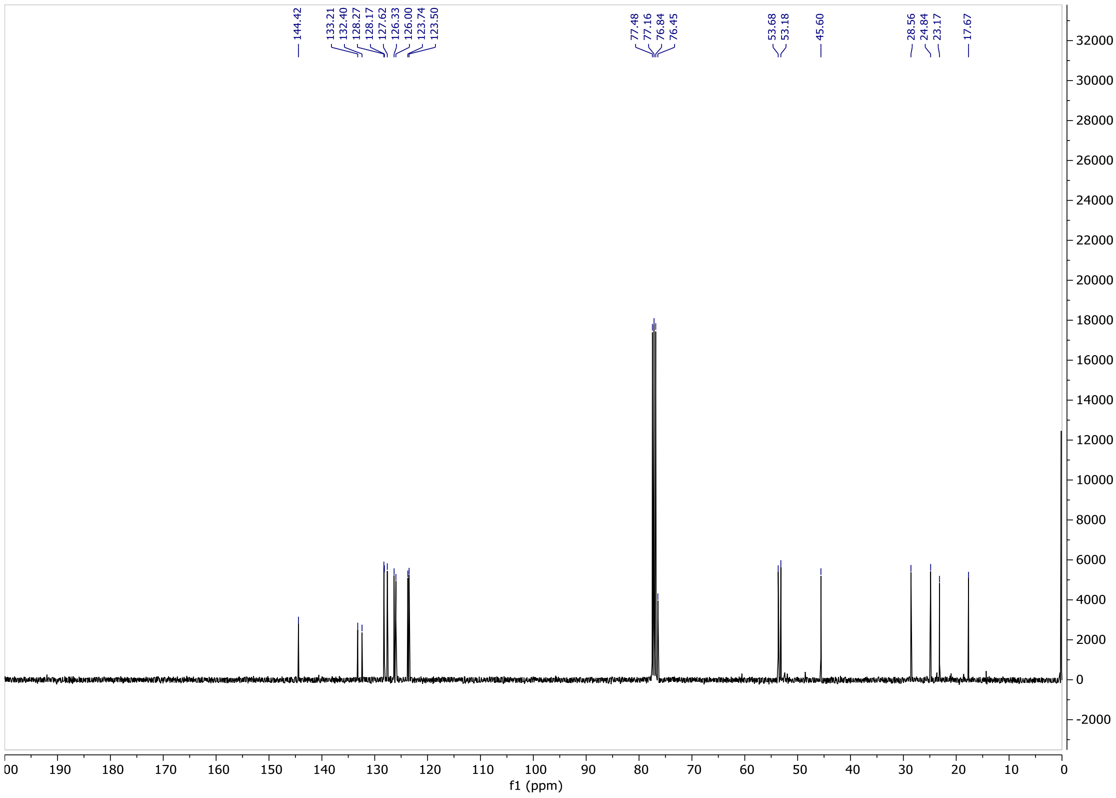
144.42 133.21 132.40 128.27 128.17 127.62 126.33 126.00 123.74 123.50 77.48 77.16 76.84 76.45 53.68 53.18 45.60 28.56 24.84 23.17 17.67
f1 (ppm)

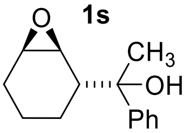

1s

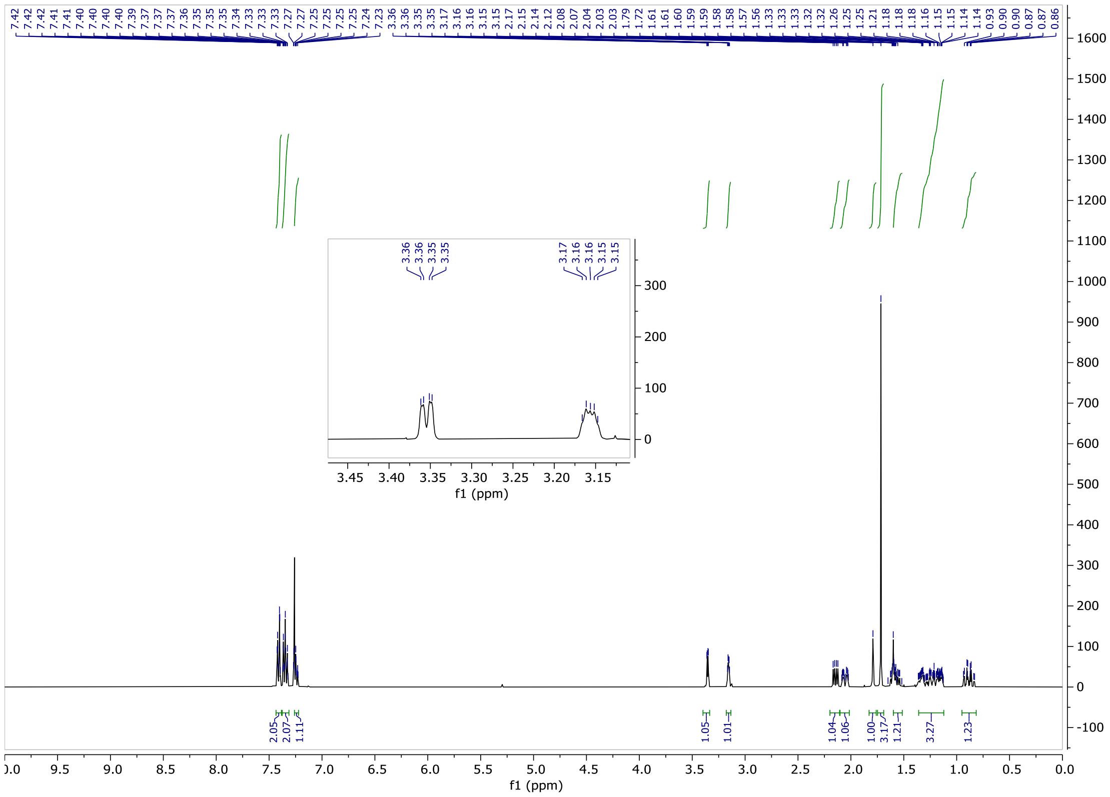

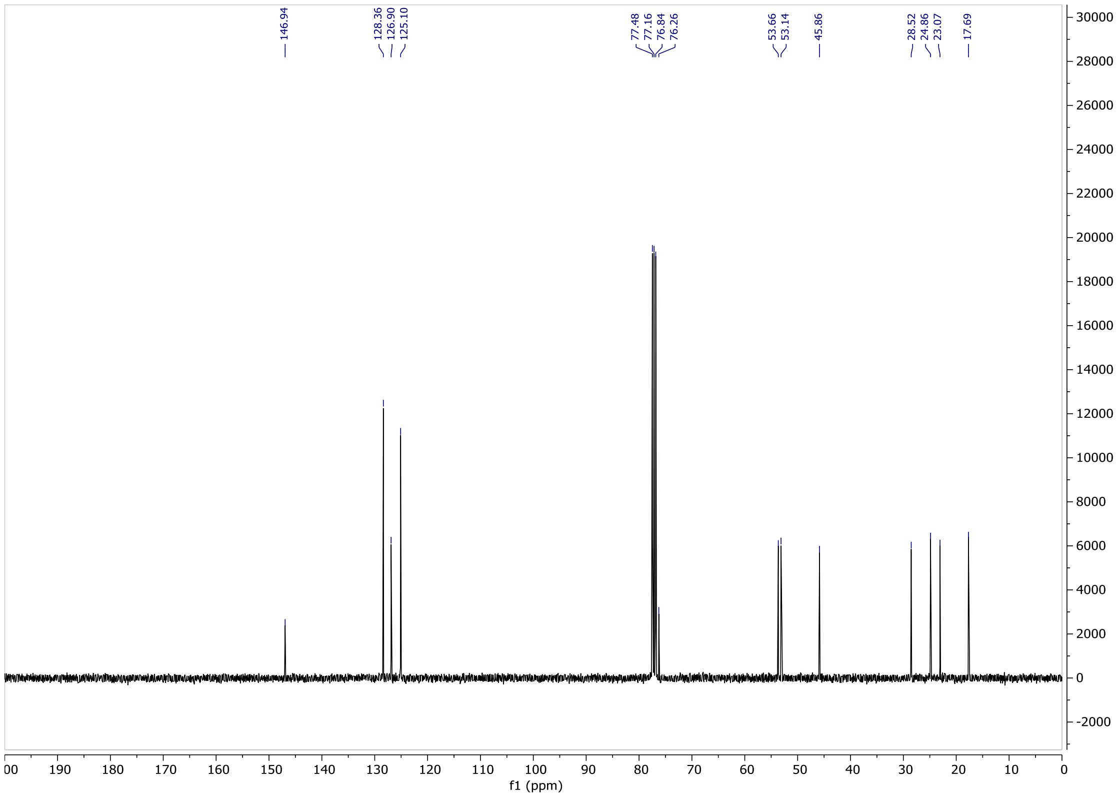

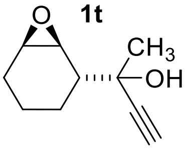

1t

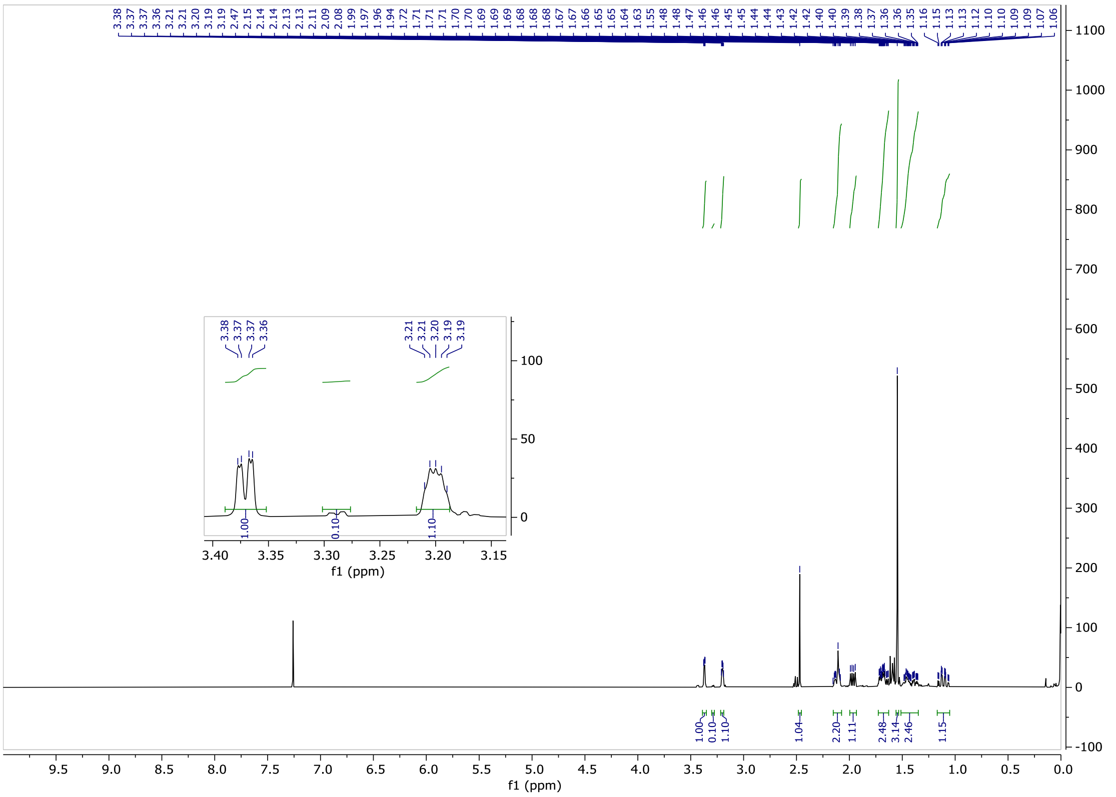

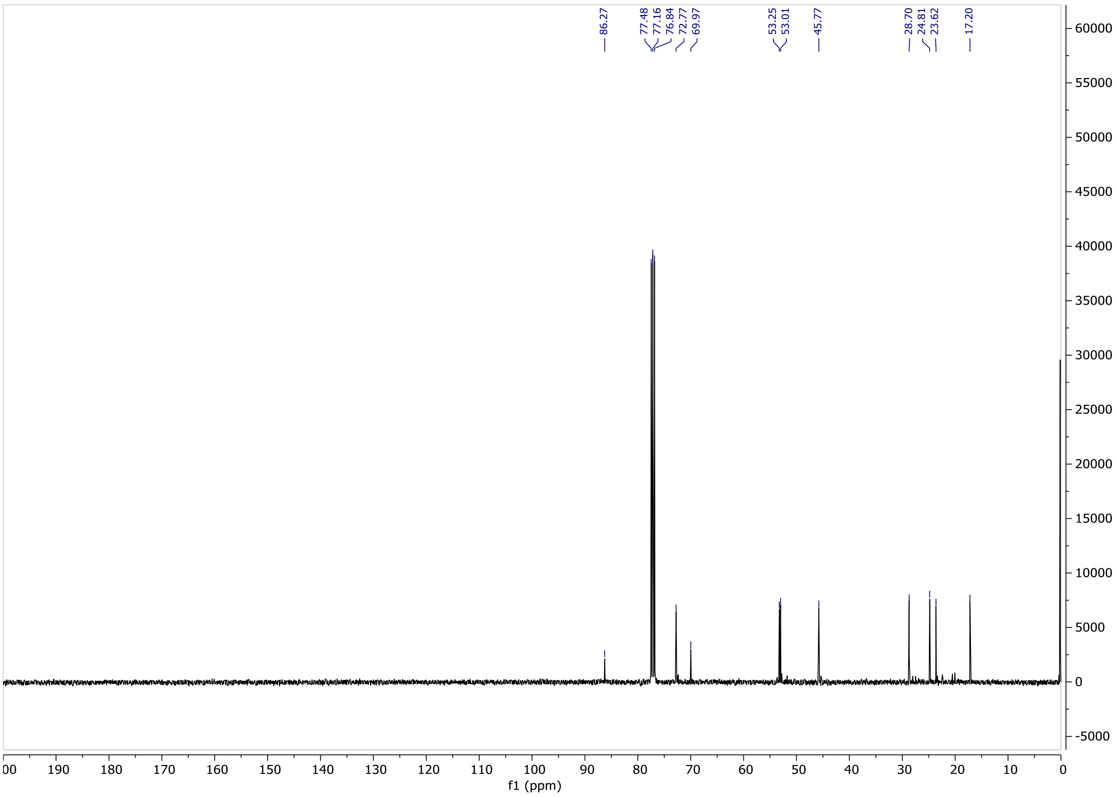

1u

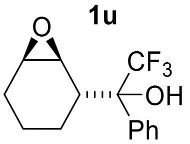
O
CF3
OH
Ph

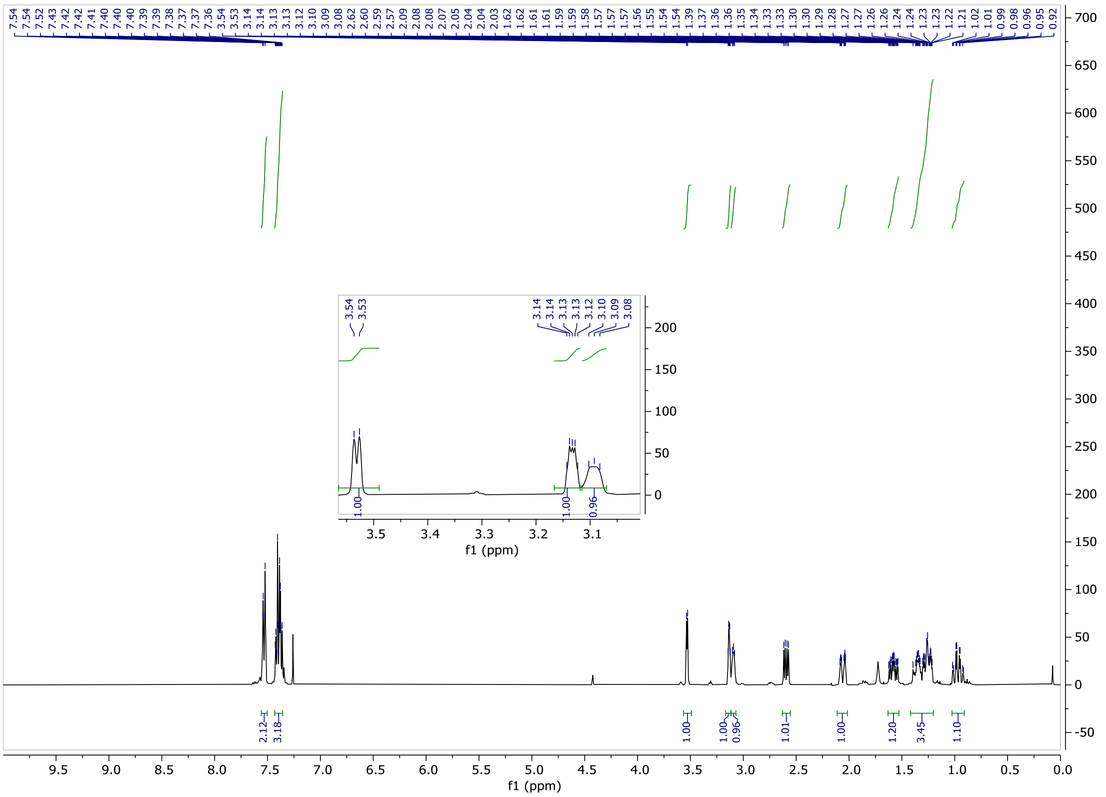
7.54 7.54 7.52 7.43 7.42 7.42 7.41 7.40 7.40 7.39 7.39 7.38 7.37 7.37 7.36 3.54 3.53 3.14 3.14 3.13 3.13 3.12 3.10 3.09 3.08 2.62 2.60 2.59 2.57 2.09 2.08 2.08 2.07 2.05 2.04 2.04 2.03 1.62 1.62 1.61 1.61 1.59 1.59 1.58 1.57 1.57 1.57 1.56 1.55 1.54 1.54 1.39 1.37 1.36 1.35 1.34 1.33 1.33 1.30 1.30 1.29 1.28 1.27 1.27 1.26 1.26 1.24 1.23 1.23 1.22 1.21 1.02 1.01 0.99 0.98 0.96 0.95 0.92
2.12 3.18 1.00 1.00 0.96 1.01 1.00 1.20 3.45 1.10
f1 (ppm)

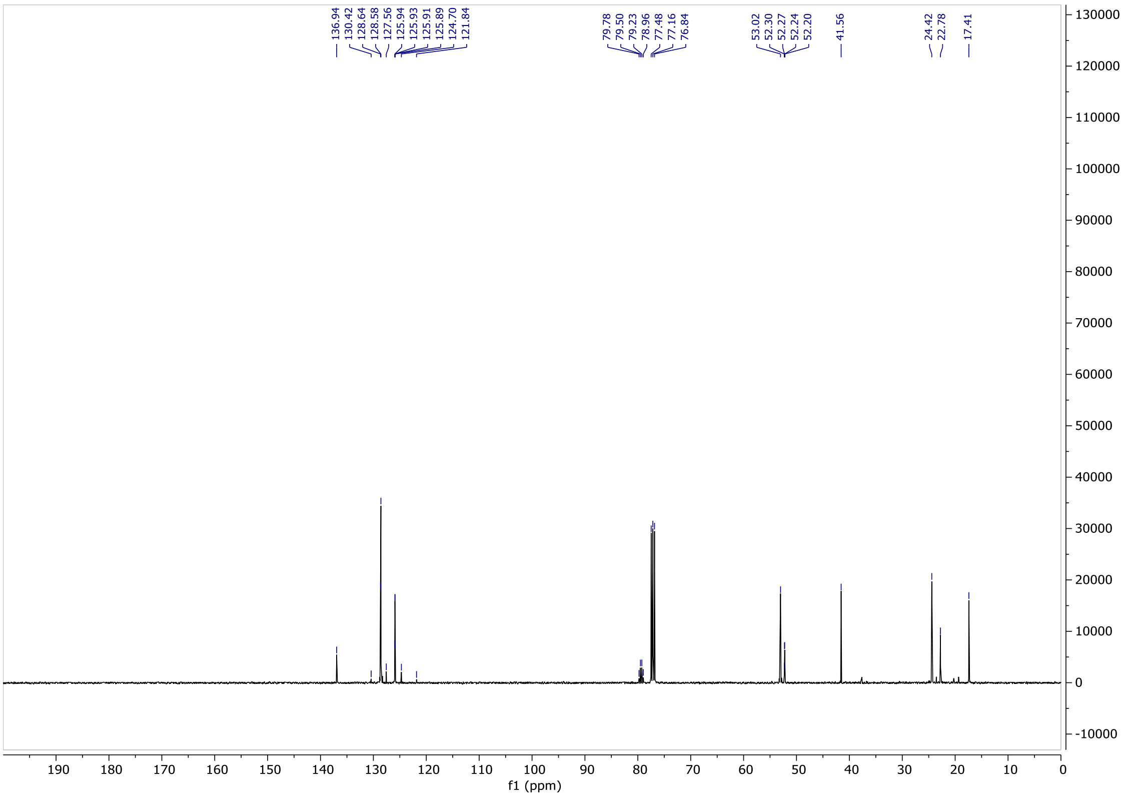
136.94 130.42 128.64 128.58 127.56 125.94 125.93 125.91 125.89 124.70 121.84
79.78 79.50 79.23 78.96 77.48 77.16 76.84
53.02 52.30 52.27 52.24 52.20 41.56
24.42 22.78 17.41
f1 (ppm)

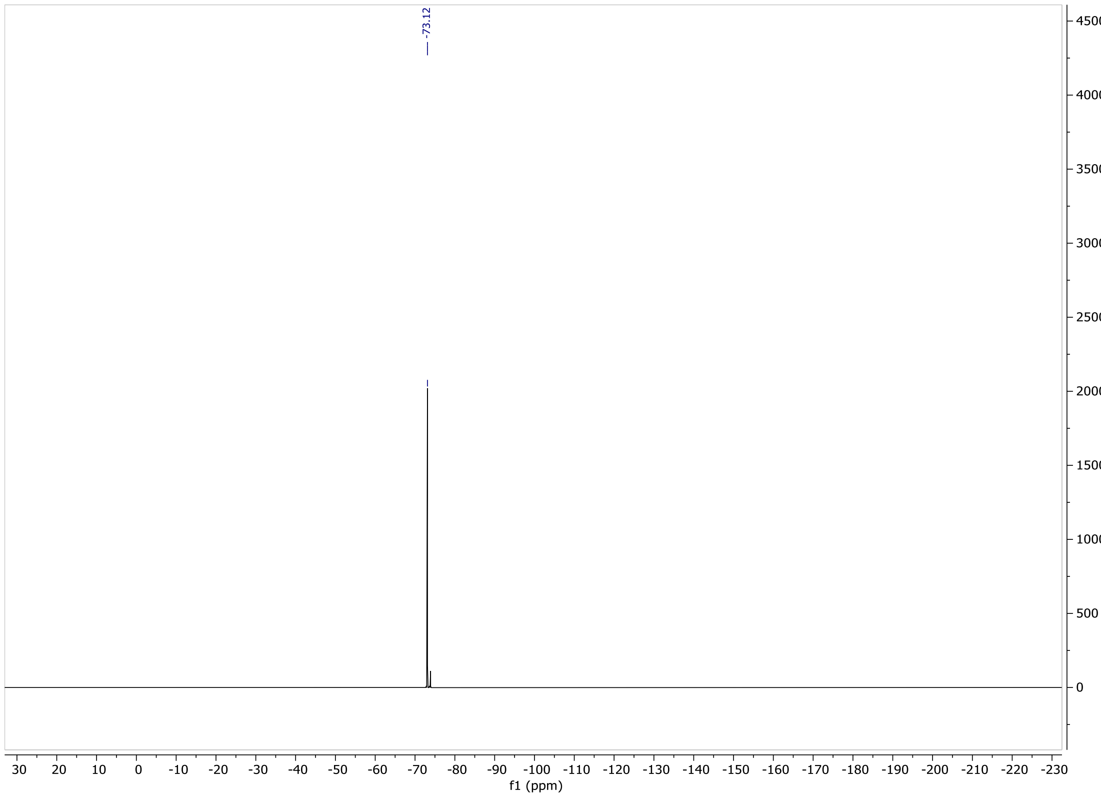

-73.12
4500
4000
3500
3000
2500
2000
1500
1000
500
0
30 20 10 0 -10 -20 -30 -40 -50 -60 -70 -80 -90 -100 -110 -120 -130 -140 -150 -160 -170 -180 -190 -200 -210 -220 -230
f1 (ppm)

$^{1}H/^{13}C$ NMR and IR spectra for the 6-membered carbonate products

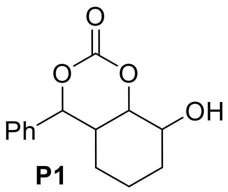

P1

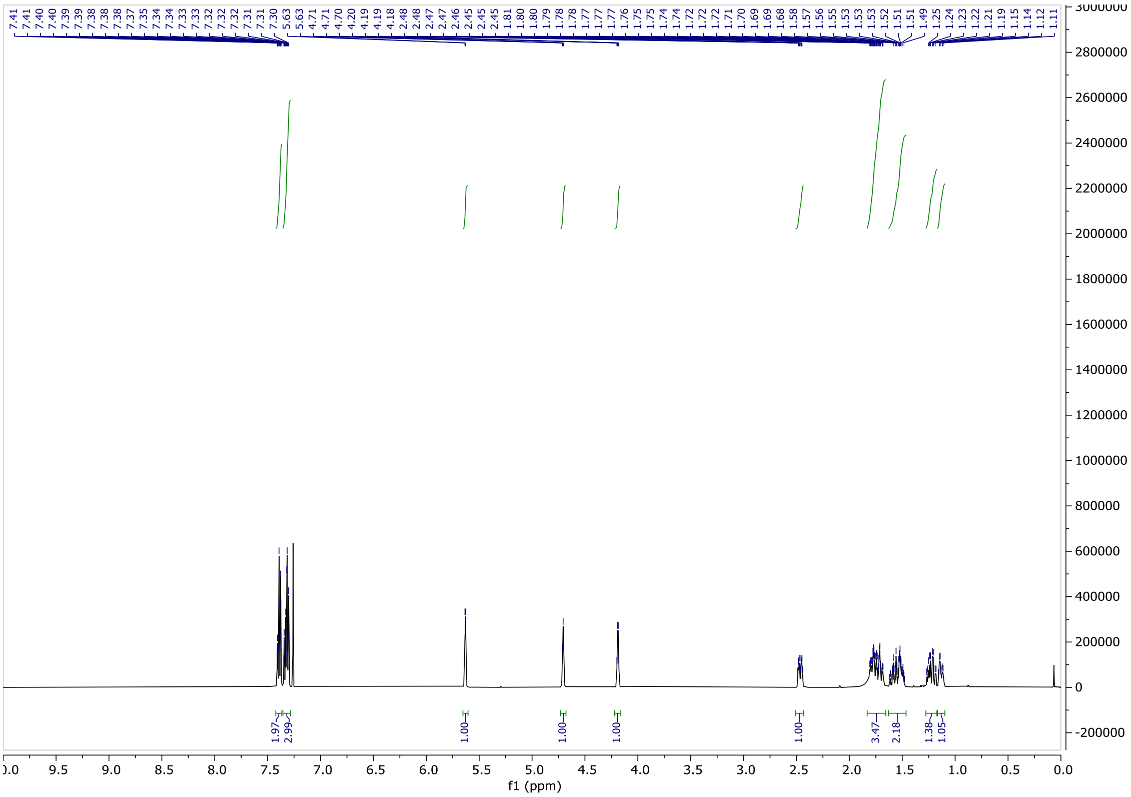

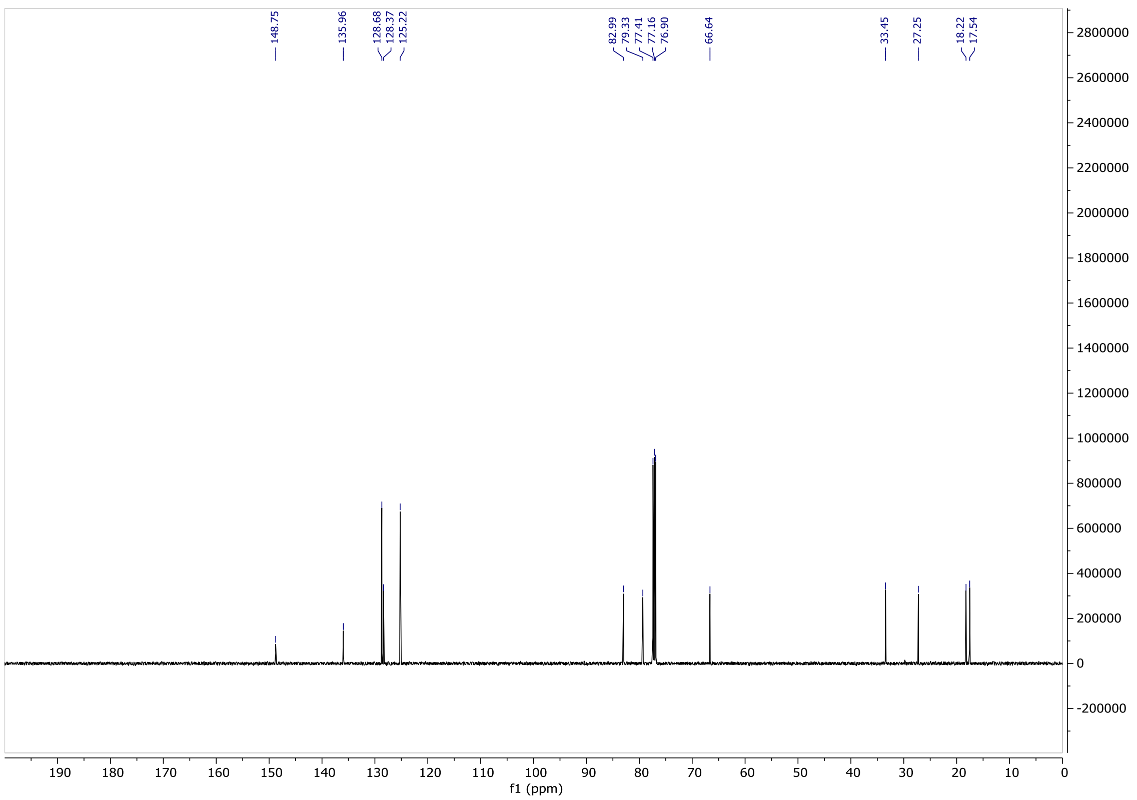
148.75
135.96
128.68
128.37
125.22
82.99
79.33
77.41
77.16
76.90
66.64
33.45
27.25
18.22
17.54
f1 (ppm)

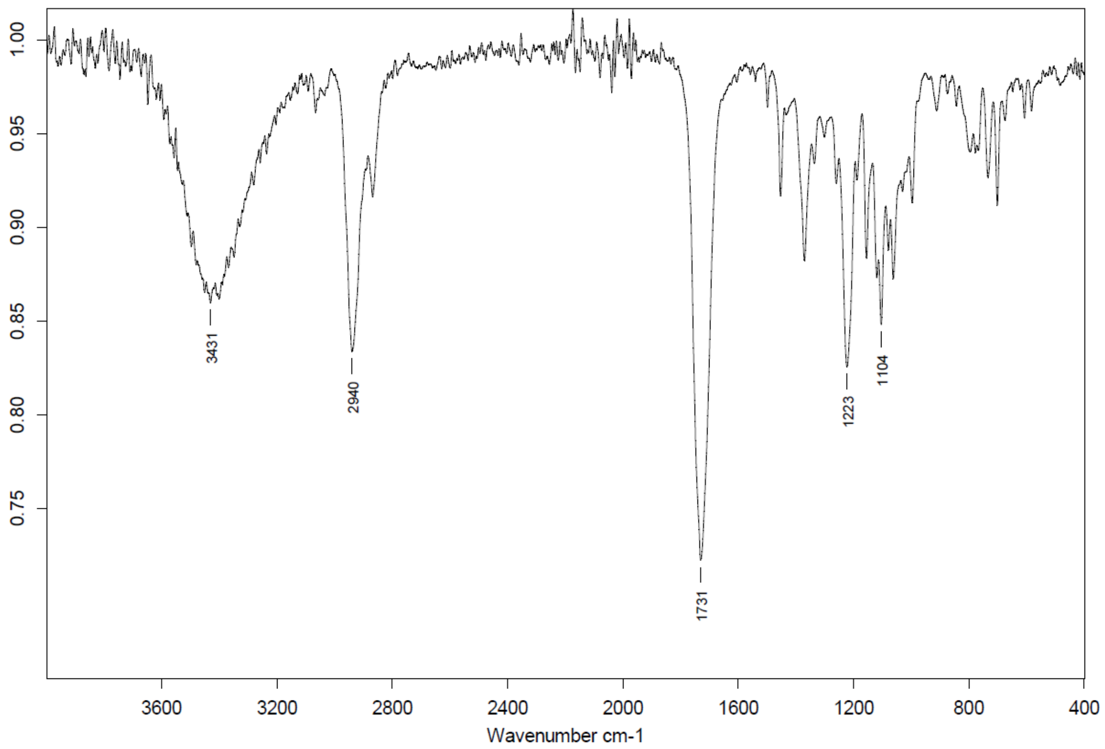
3431
2940
1731
1223
1104
Wavenumber cm-1

P2

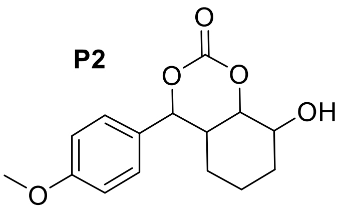
O
O
O
OH
O

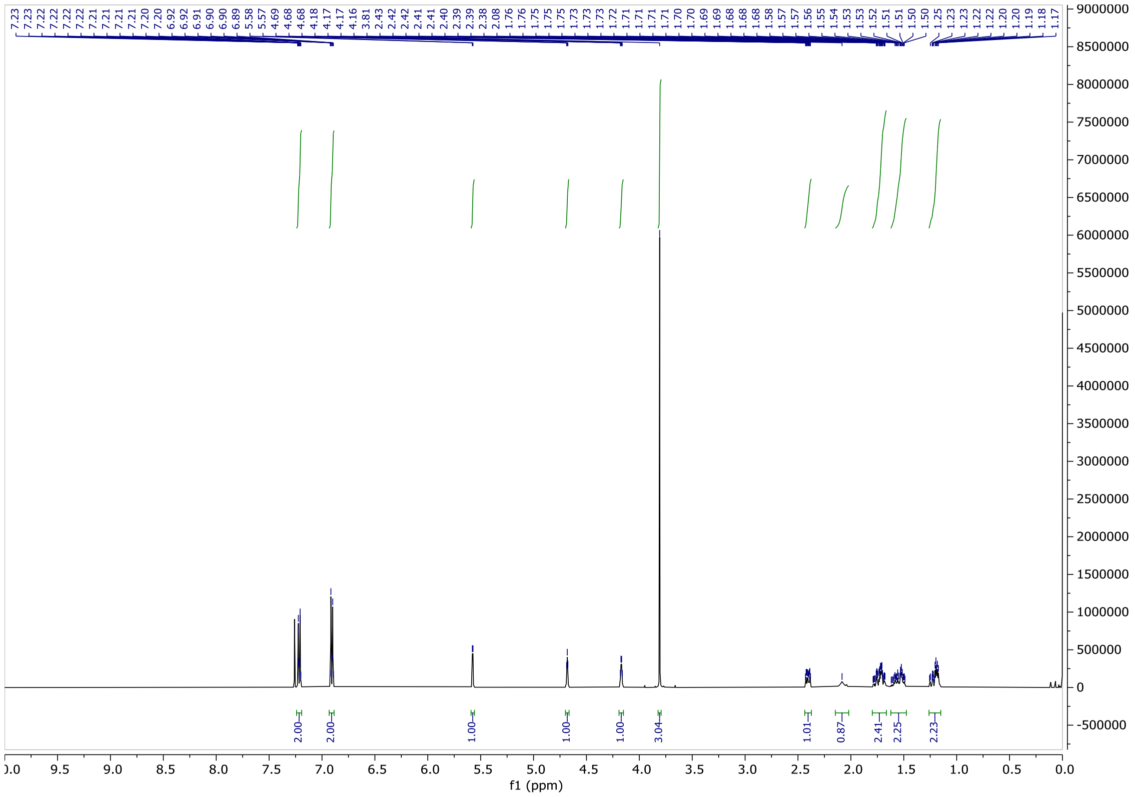
2.00
2.00
1.00
1.00
1.00
3.04
1.01
0.87
2.41
2.25
2.23
f1 (ppm)

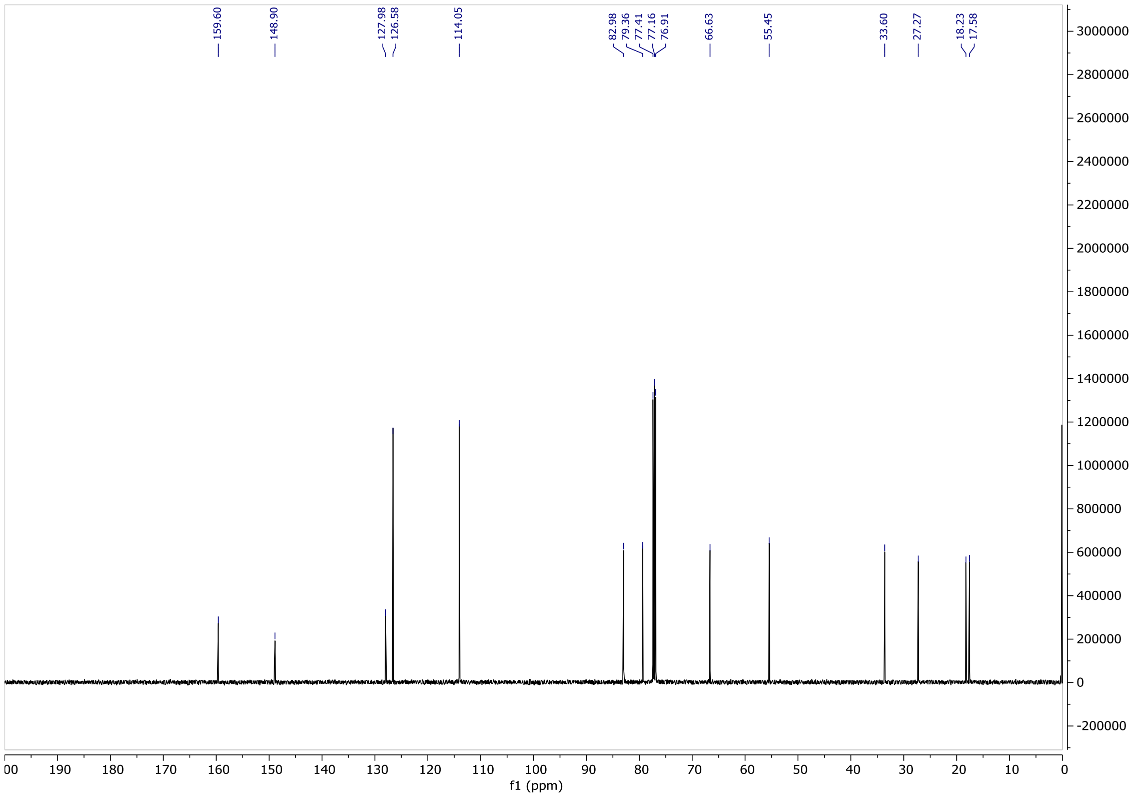
159.60
148.90
127.98
126.58
114.05
82.98
79.36
77.41
77.16
76.91
66.63
55.45
33.60
27.27
18.23
17.58
f1 (ppm)

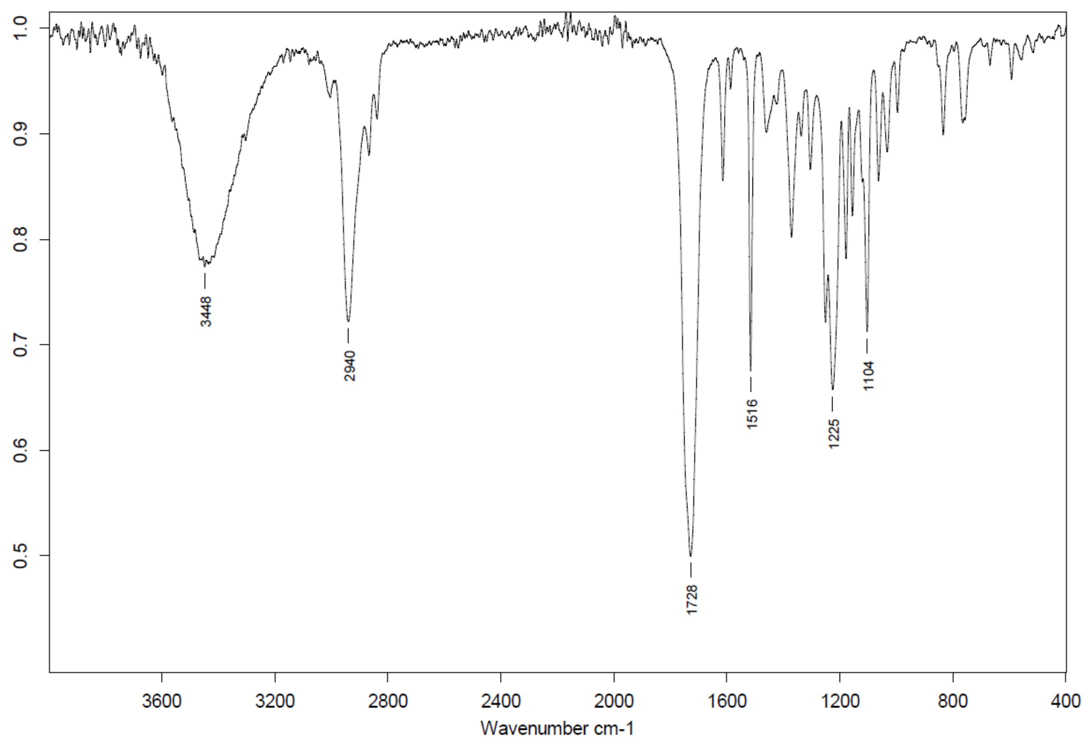

1.0
0.9
0.8
0.7
0.6
0.5
3448
2940
1728
1516
1225
1104
3600
3200
2800
2400
2000
1600
1200
800
400
Wavenumber cm-1

P3

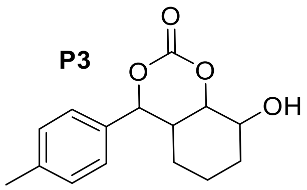
O
O
O
OH

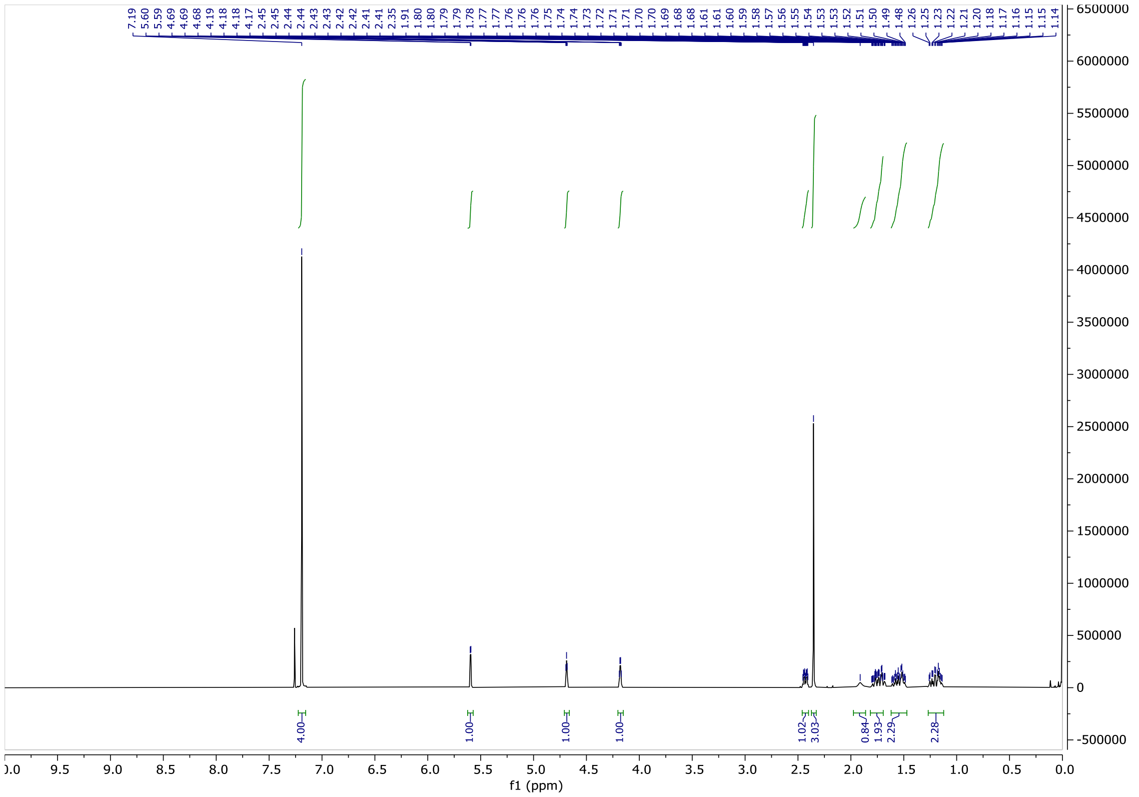
7.19
5.60
5.59
4.69
4.69
4.68
4.19
4.18
4.18
4.17
2.45
2.45
2.44
2.44
2.43
2.43
2.42
2.42
2.41
2.41
2.35
1.91
1.80
1.79
1.79
1.78
1.77
1.77
1.76
1.76
1.75
1.74
1.74
1.73
1.72
1.71
1.71
1.70
1.70
1.69
1.68
1.68
1.61
1.61
1.60
1.59
1.58
1.57
1.56
1.55
1.54
1.53
1.53
1.52
1.51
1.50
1.49
1.48
1.26
1.25
1.23
1.22
1.21
1.20
1.18
1.17
1.16
1.15
1.15
1.14
4.00
1.00
1.00
1.00
1.02
3.03
0.84
1.93
2.29
2.28
f1 (ppm)

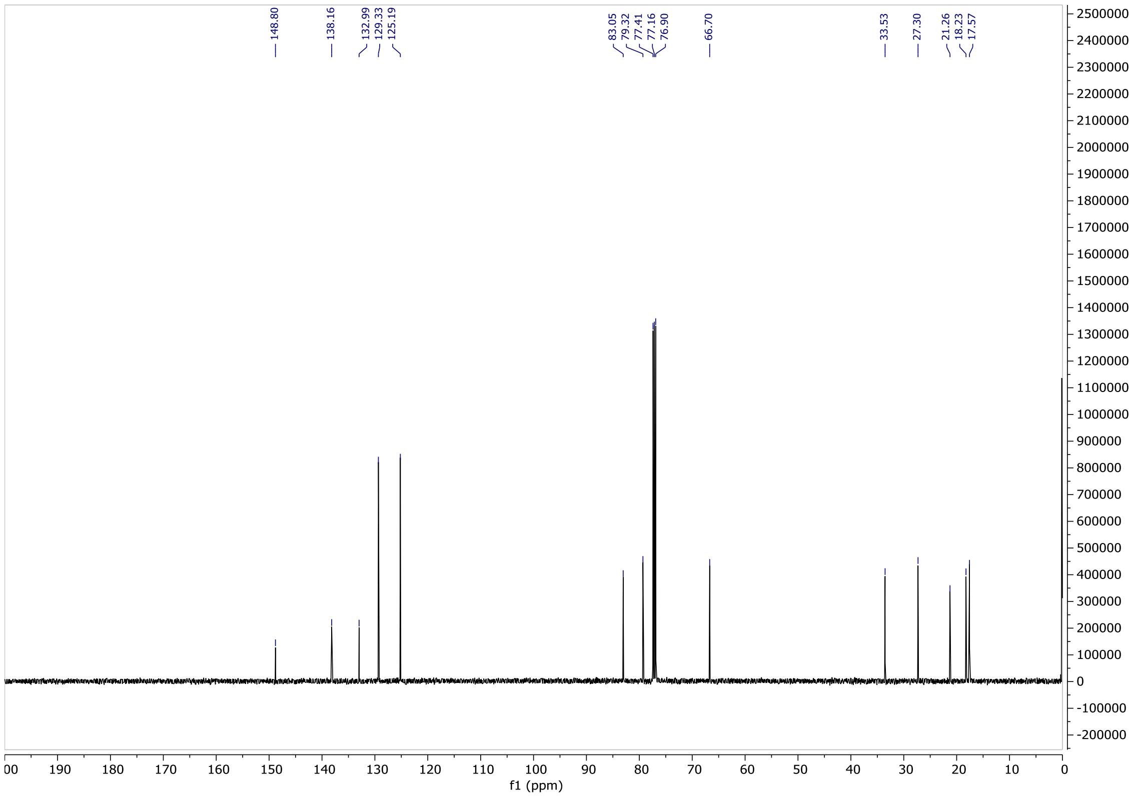
148.80
138.16
132.99
129.33
125.19
83.05
79.32
77.41
77.16
76.90
66.70
33.53
27.30
21.26
18.23
17.57
f1 (ppm)

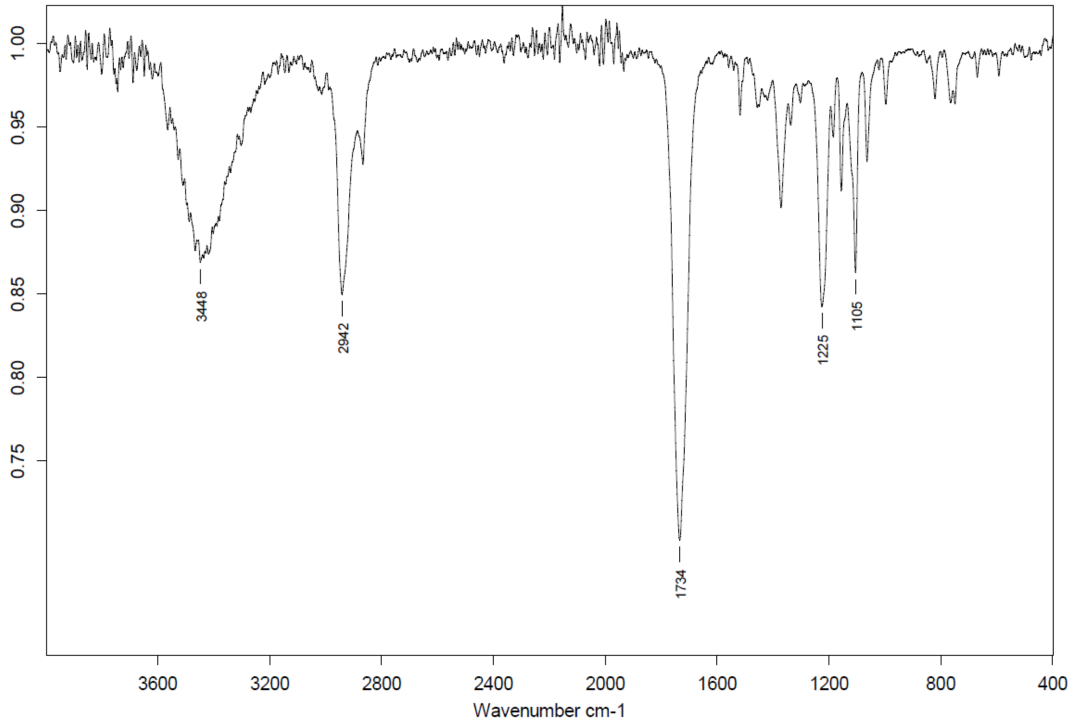

1.00
0.95
0.90
0.85
0.80
0.75
3448
2942
1734
1225
1105
3600
3200
2800
2400
2000
1600
1200
800
400
Wavenumber cm-1

P4

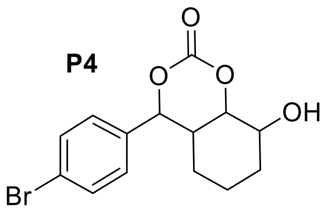
O
O
O
OH
Br

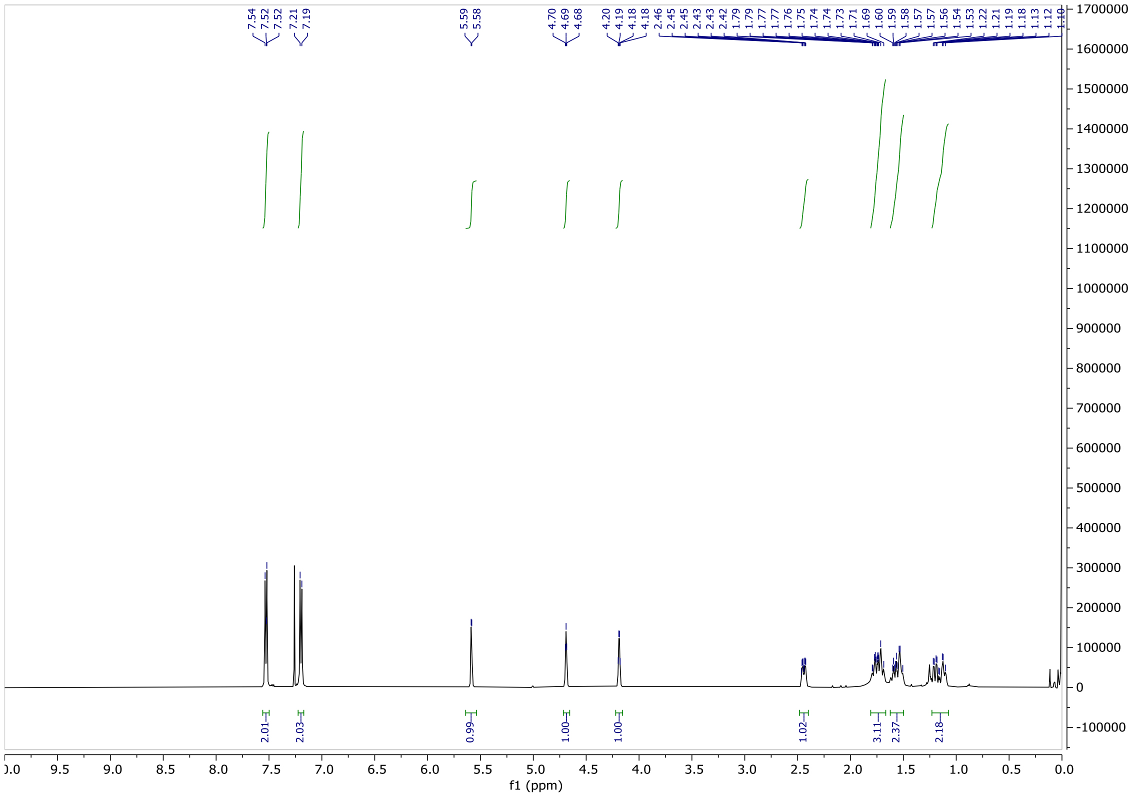
7.54
7.52
7.52
7.21
7.19
5.59
5.58
4.70
4.69
4.68
4.20
4.19
4.18
4.18
2.46
2.45
2.45
2.43
2.42
1.79
1.79
1.77
1.76
1.75
1.74
1.73
1.71
1.69
1.60
1.59
1.58
1.57
1.57
1.56
1.54
1.53
1.22
1.21
1.19
1.18
1.13
1.12
1.10
2.01
2.03
0.99
1.00
1.00
1.02
3.11
2.37
2.18
f1 (ppm)

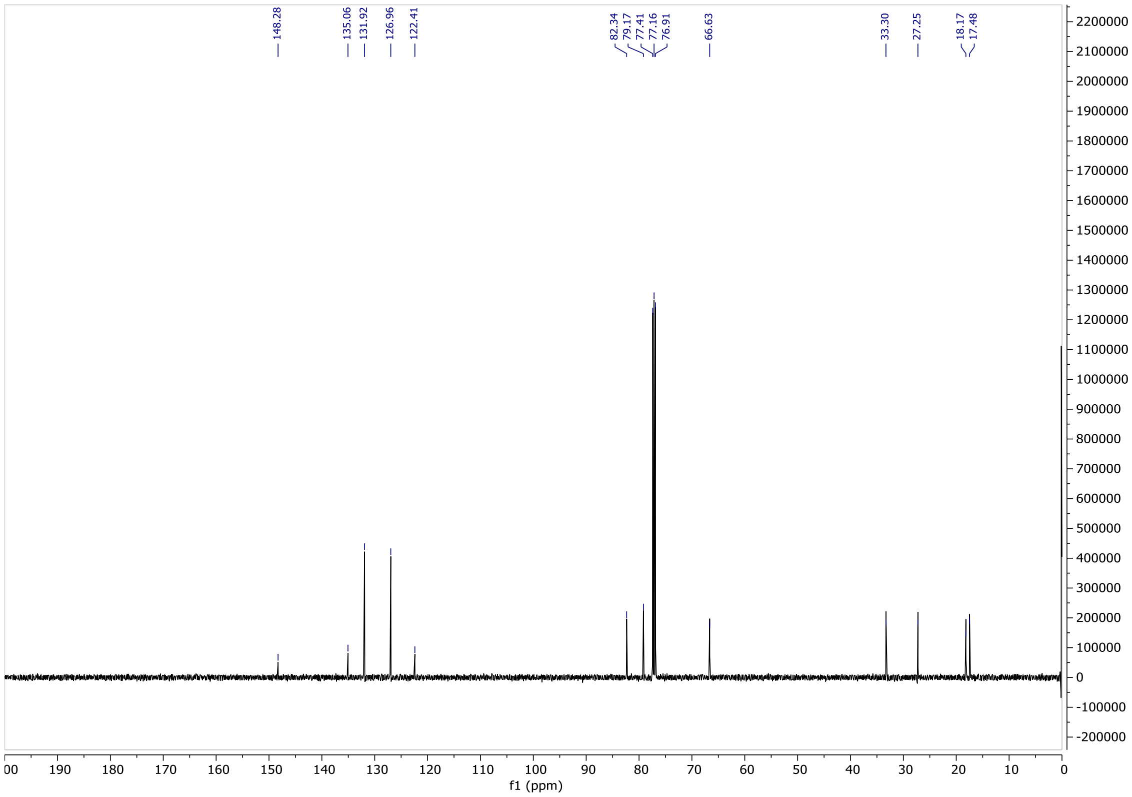
148.28
135.06
131.92
126.96
122.41
82.34
79.17
77.41
77.16
76.91
66.63
33.30
27.25
18.17
17.48
f1 (ppm)

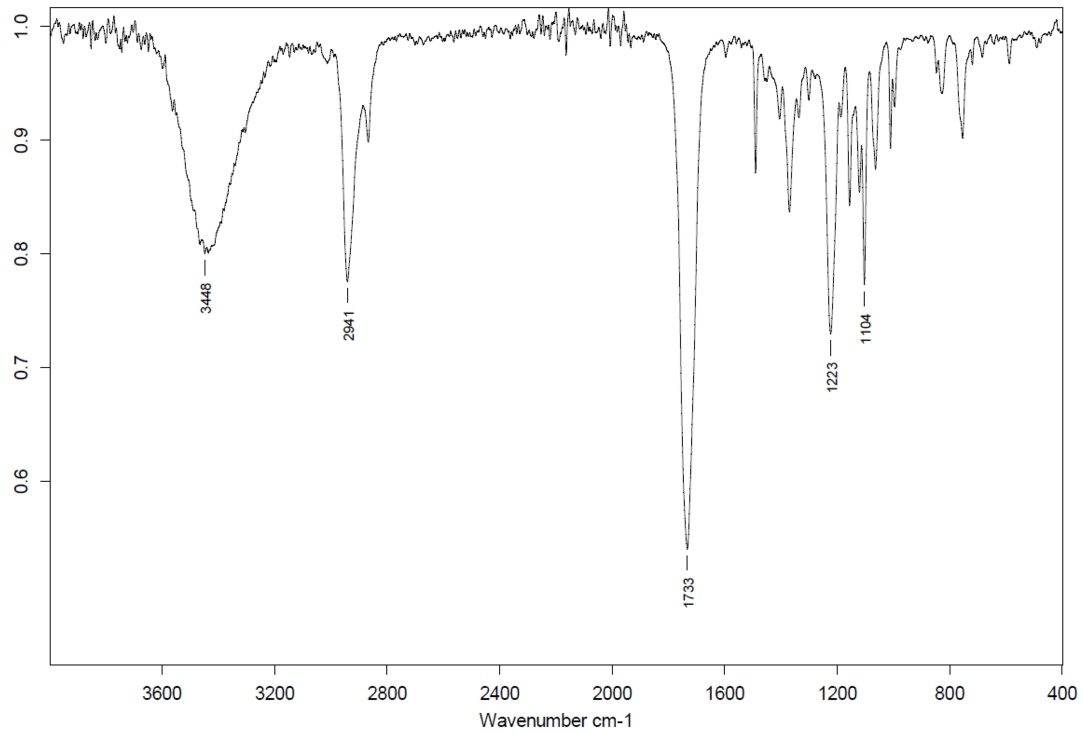
1.0
0.9
0.8
0.7
0.6
3448
2941
1733
1223
1104
3600
3200
2800
2400
2000
1600
1200
800
400
Wavenumber cm-1

P5

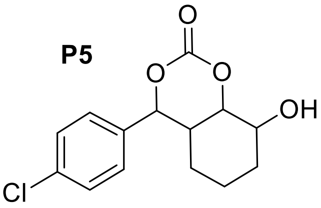
O
O
O
OH
Cl

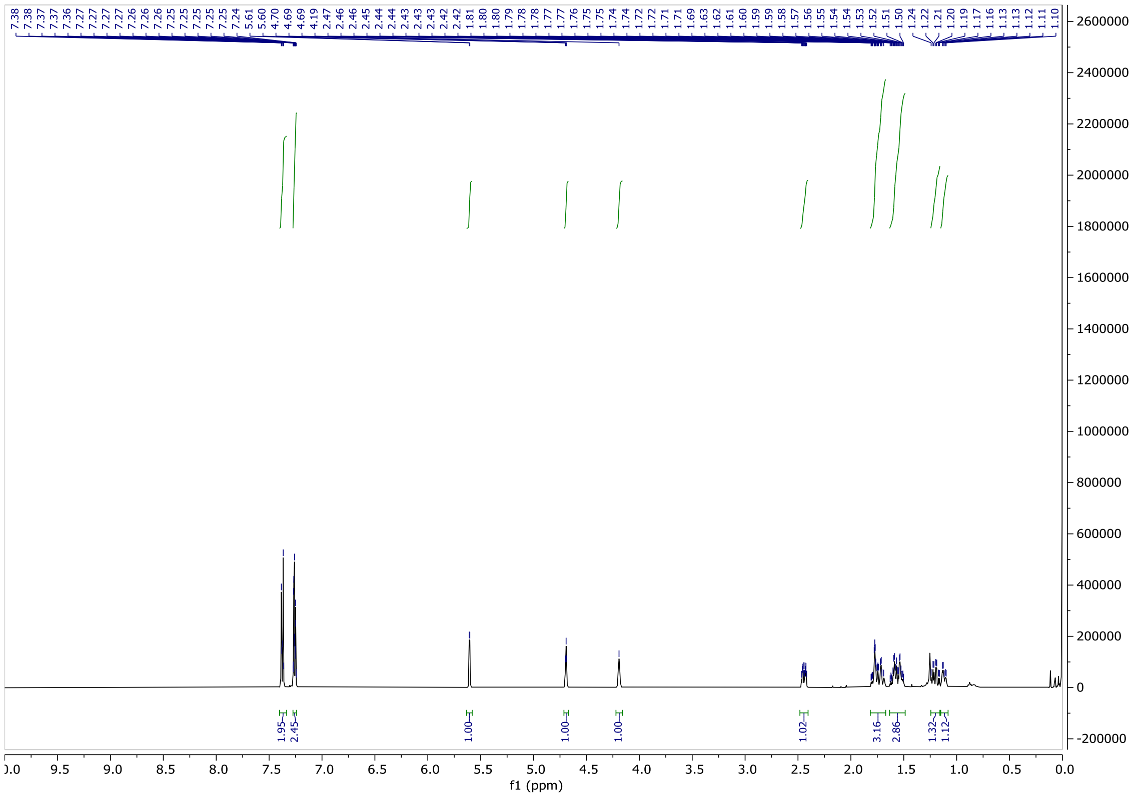
7.38 7.38 7.37 7.36 7.27 7.27 7.27 7.26 7.26 7.25 7.25 7.25 7.25 7.24 5.61 5.60 4.70 4.69 4.69 4.19 2.47 2.46 2.46 2.45 2.44 2.44 2.43 2.43 2.43 2.42 2.42 1.81 1.80 1.80 1.79 1.78 1.78 1.77 1.77 1.76 1.75 1.75 1.74 1.74 1.72 1.72 1.71 1.71 1.69 1.63 1.62 1.61 1.60 1.59 1.59 1.58 1.57 1.56 1.55 1.54 1.54 1.53 1.53 1.52 1.51 1.50 1.24 1.22 1.21 1.20 1.19 1.17 1.16 1.13 1.13 1.12 1.11 1.10
1.95
2.45
1.00
1.00
1.00
1.02
3.16
2.86
1.32
1.12
f1 (ppm)

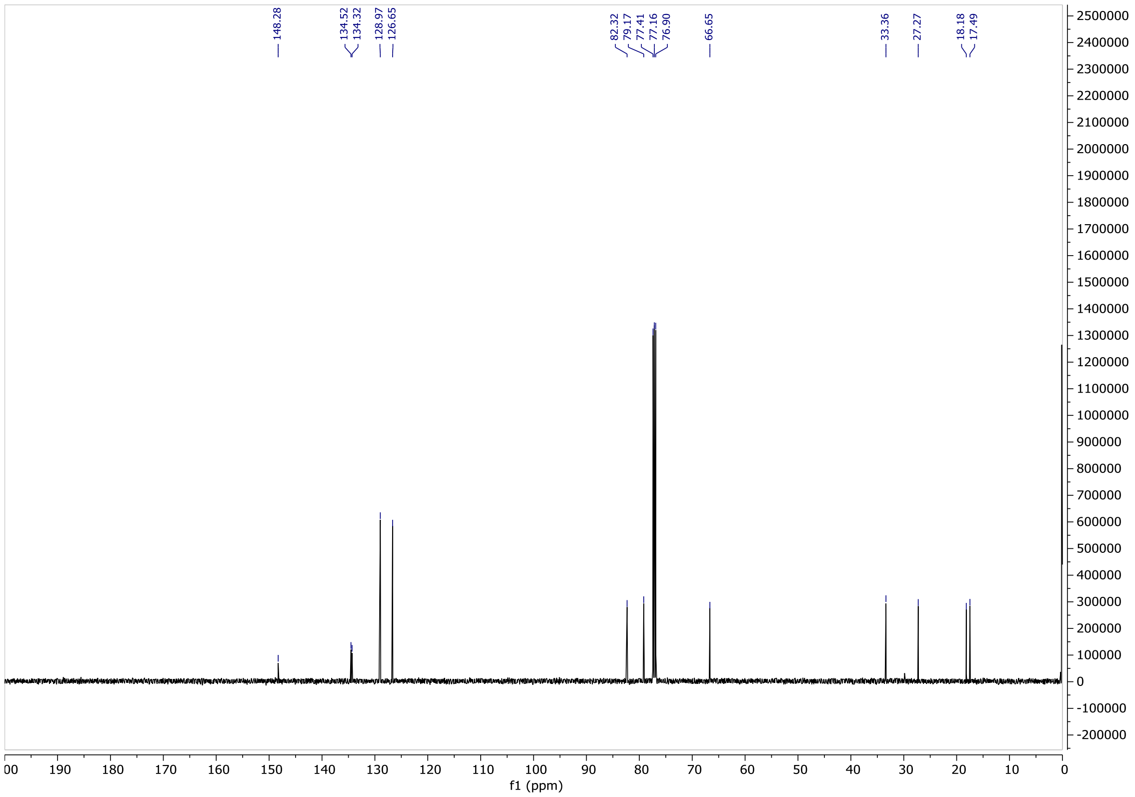
148.28
134.52
134.32
128.97
126.65
82.32
79.17
77.41
77.16
76.90
66.65
33.36
27.27
18.18
17.49
f1 (ppm)

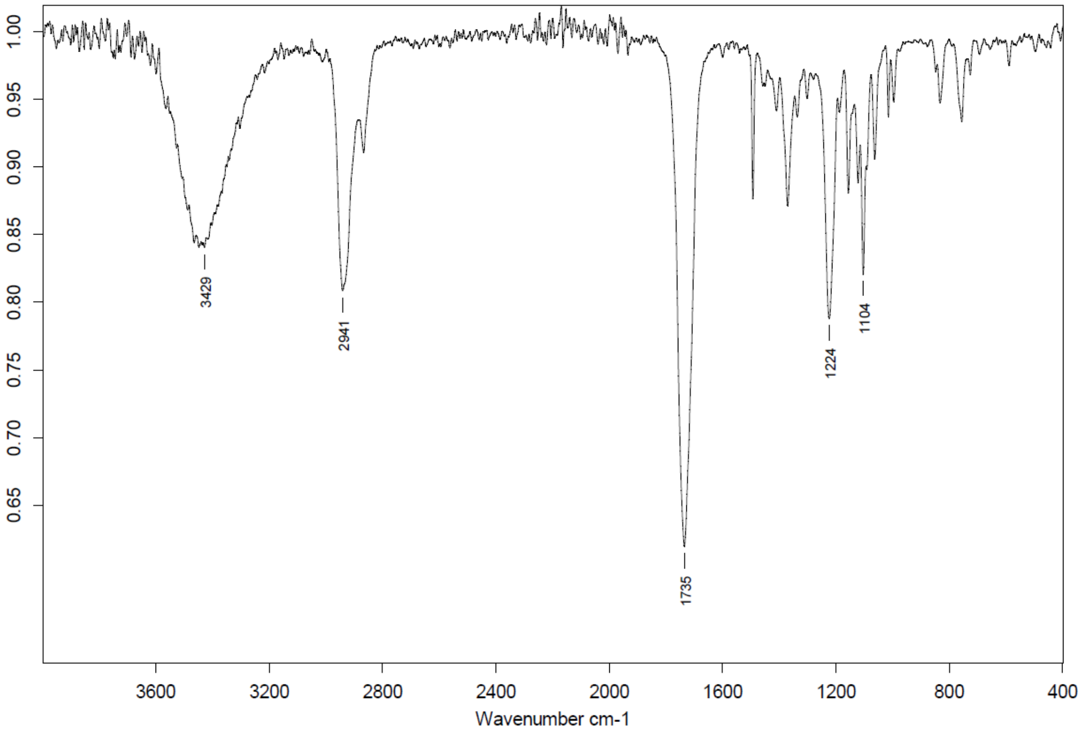

1.00
0.95
0.90
0.85
0.80
0.75
0.70
0.65
3429
2941
1735
1224
1104
3600
3200
2800
2400
2000
1600
1200
800
400
Wavenumber cm-1

P6

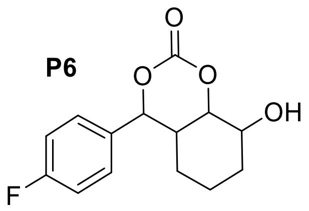
O
O
O
OH
F

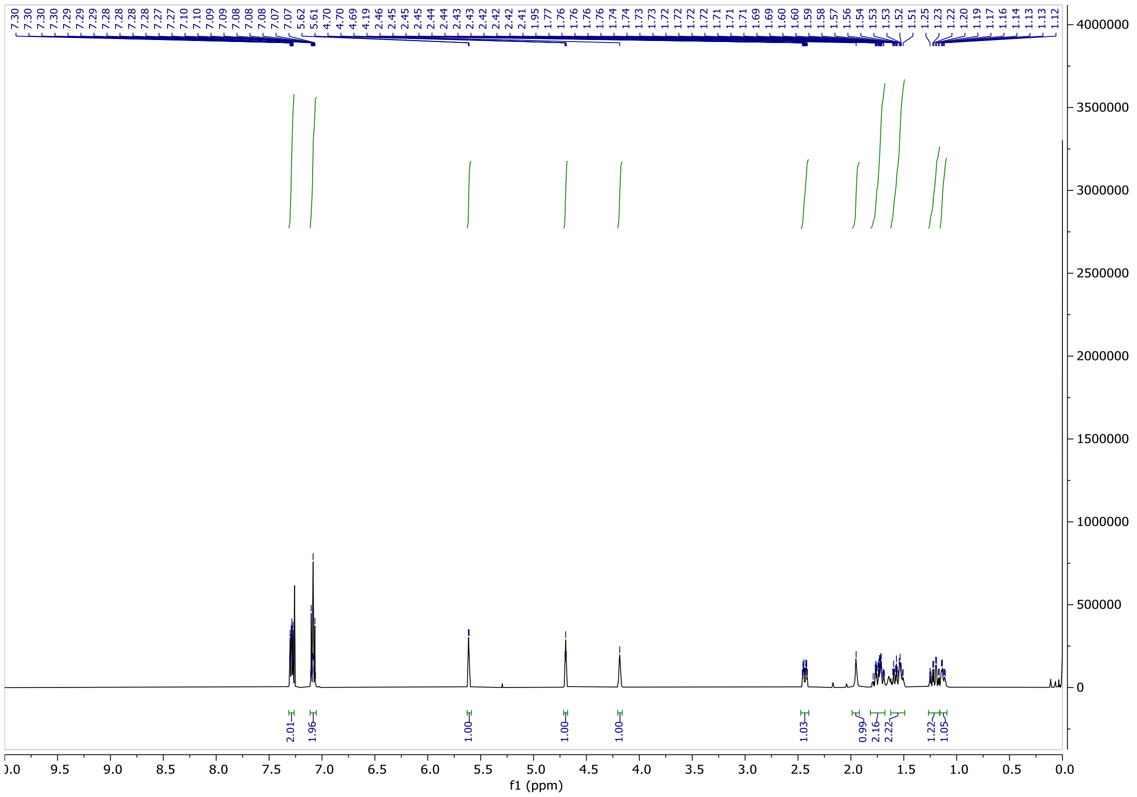
7.30 7.30 7.30 7.29 7.29 7.28 7.28 7.28 7.27 7.27 7.10 7.10 7.09 7.09 7.08 7.08 7.08 7.07 7.07 5.62 5.61 4.70 4.70 4.69 4.19 2.46 2.45 2.45 2.45 2.44 2.44 2.43 2.43 2.42 2.42 2.42 2.41 1.95 1.77 1.76 1.76 1.76 1.74 1.74 1.73 1.73 1.72 1.72 1.72 1.71 1.71 1.71 1.69 1.69 1.60 1.59 1.58 1.57 1.56 1.54 1.53 1.53 1.52 1.51 1.25 1.23 1.22 1.20 1.19 1.17 1.16 1.14 1.13 1.12
2.01
1.96
1.00
1.00
1.00
1.03
0.99
2.16
2.22
1.22
1.05
4000000
3500000
3000000
2500000
2000000
1500000
1000000
500000
0
9.5 9.0 8.5 8.0 7.5 7.0 6.5 6.0 5.5 5.0 4.5 4.0 3.5 3.0 2.5 2.0 1.5 1.0 0.5 0.0
f1 (ppm)

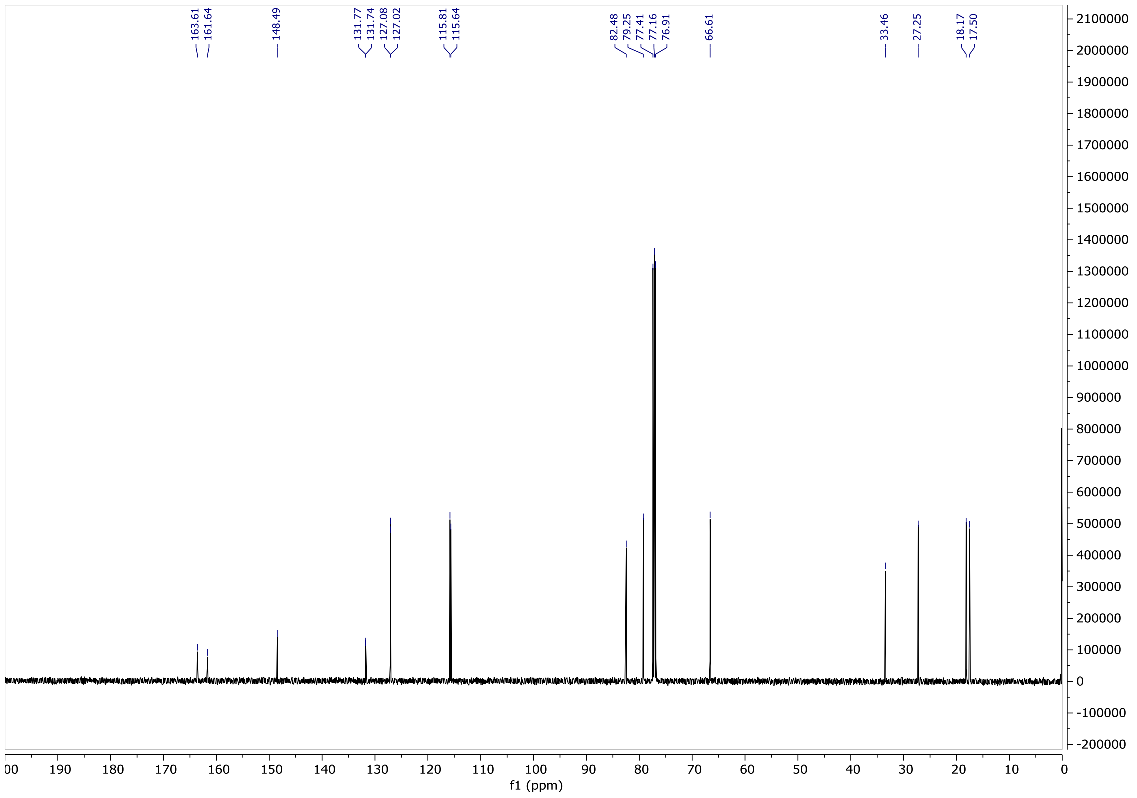
163.61 161.64
148.49
131.77 131.74 127.08 127.02
115.81 115.64
82.48 79.25 77.41 77.16 76.91
66.61
33.46
27.25
18.17 17.50
2100000
2000000
1900000
1800000
1700000
1600000
1500000
1400000
1300000
1200000
1100000
1000000
900000
800000
700000
600000
500000
400000
300000
200000
100000
0
-100000
-200000
190 180 170 160 150 140 130 120 110 100 90 80 70 60 50 40 30 20 10 0
f1 (ppm)

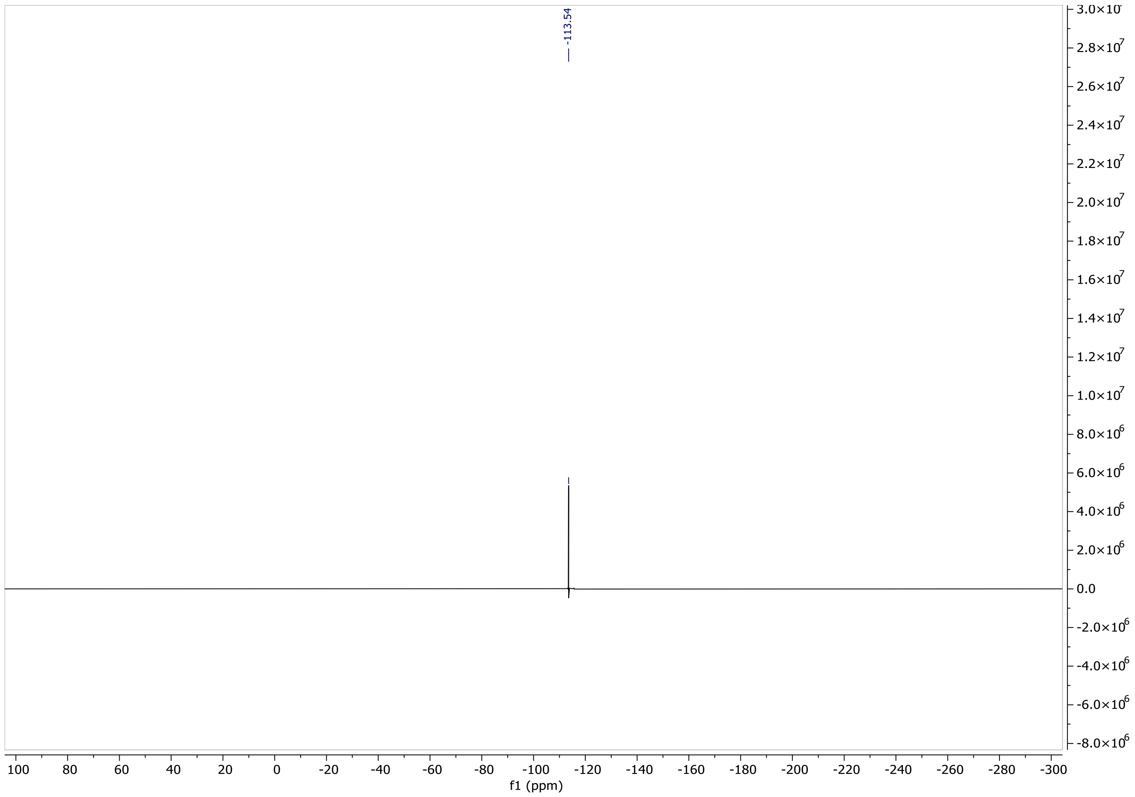
-113.54
3.0×10^7
2.8×10^7
2.6×10^7
2.4×10^7
2.2×10^7
2.0×10^7
1.8×10^7
1.6×10^7
1.4×10^7
1.2×10^7
1.0×10^7
8.0×10^6
6.0×10^6
4.0×10^6
2.0×10^6
0.0
-2.0×10^6
-4.0×10^6
-6.0×10^6
-8.0×10^6
100 80 60 40 20 0 -20 -40 -60 -80 -100 -120 -140 -160 -180 -200 -220 -240 -260 -280 -300
f1 (ppm)

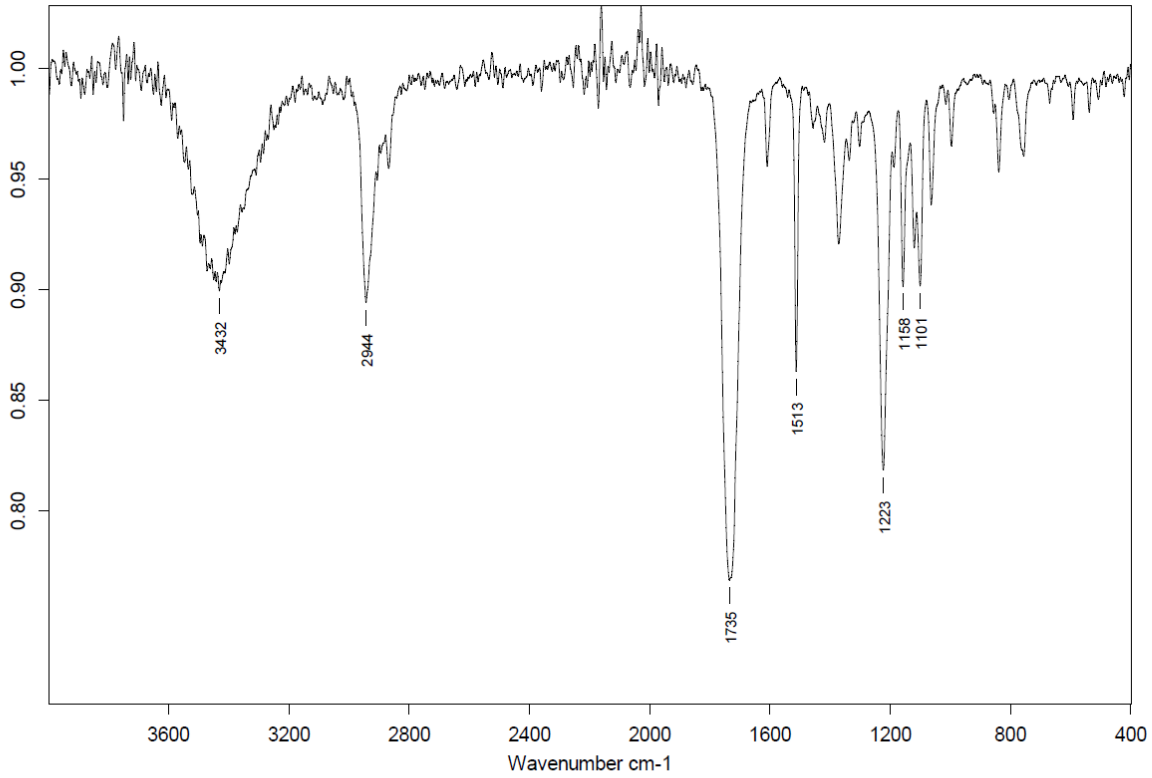
1.00
0.95
0.90
0.85
0.80
3432
2944
1735
1513
1223
1158
1101
3600 3200 2800 2400 2000 1600 1200 800 400
Wavenumber cm-1

P7

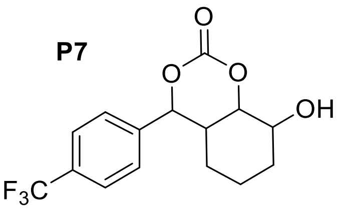

O
O
O
OH
F3C

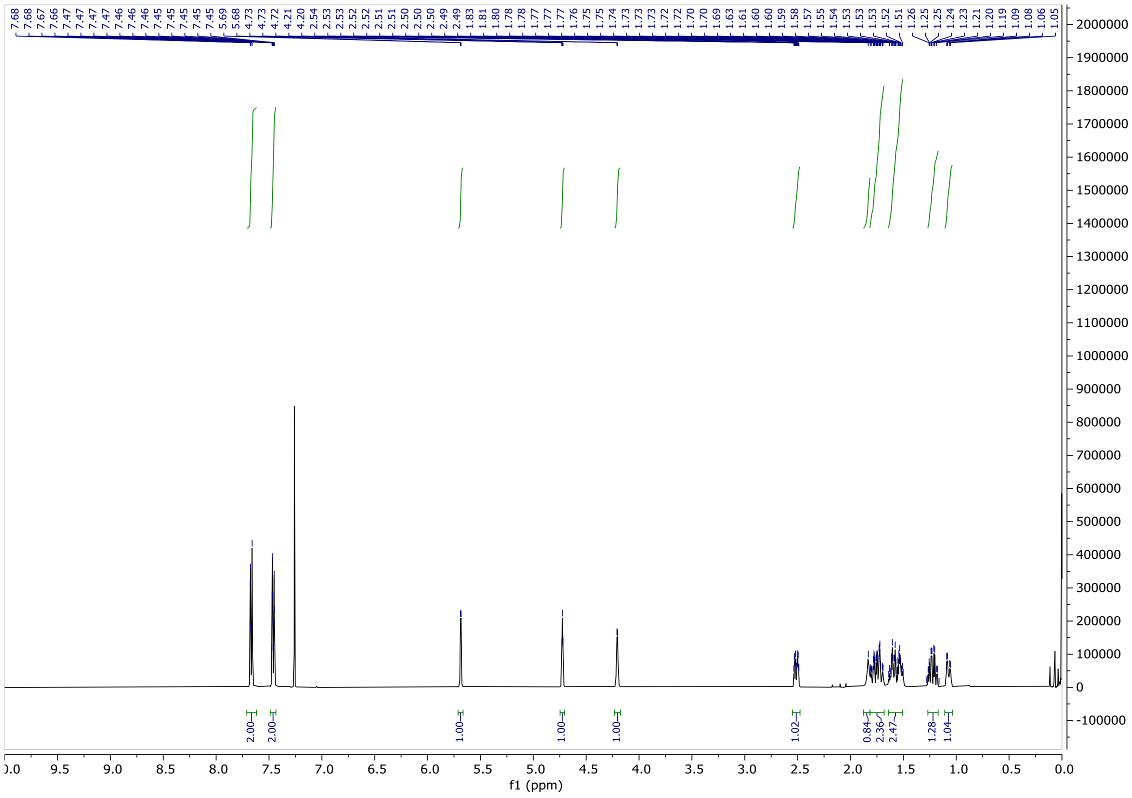

7.68 7.68 7.67 7.66 7.47 7.47 7.47 7.46 7.46 7.45 7.45 7.45 7.45 7.45 5.69 5.68 4.73 4.73 4.72 4.21 4.20 2.54 2.53 2.53 2.52 2.52 2.51 2.51 2.50 2.50 2.50 2.49 2.49 1.83 1.81 1.80 1.78 1.78 1.77 1.77 1.76 1.75 1.75 1.74 1.73 1.73 1.73 1.72 1.70 1.70 1.69 1.63 1.61 1.60 1.60 1.59 1.58 1.57 1.55 1.54 1.53 1.53 1.53 1.52 1.51 1.26 1.25 1.25 1.24 1.23 1.21 1.20 1.19 1.09 1.08 1.06 1.05
2.00 2.00 1.00 1.00 1.00 1.02 0.84 2.36 2.47 1.28 1.04
f1 (ppm)

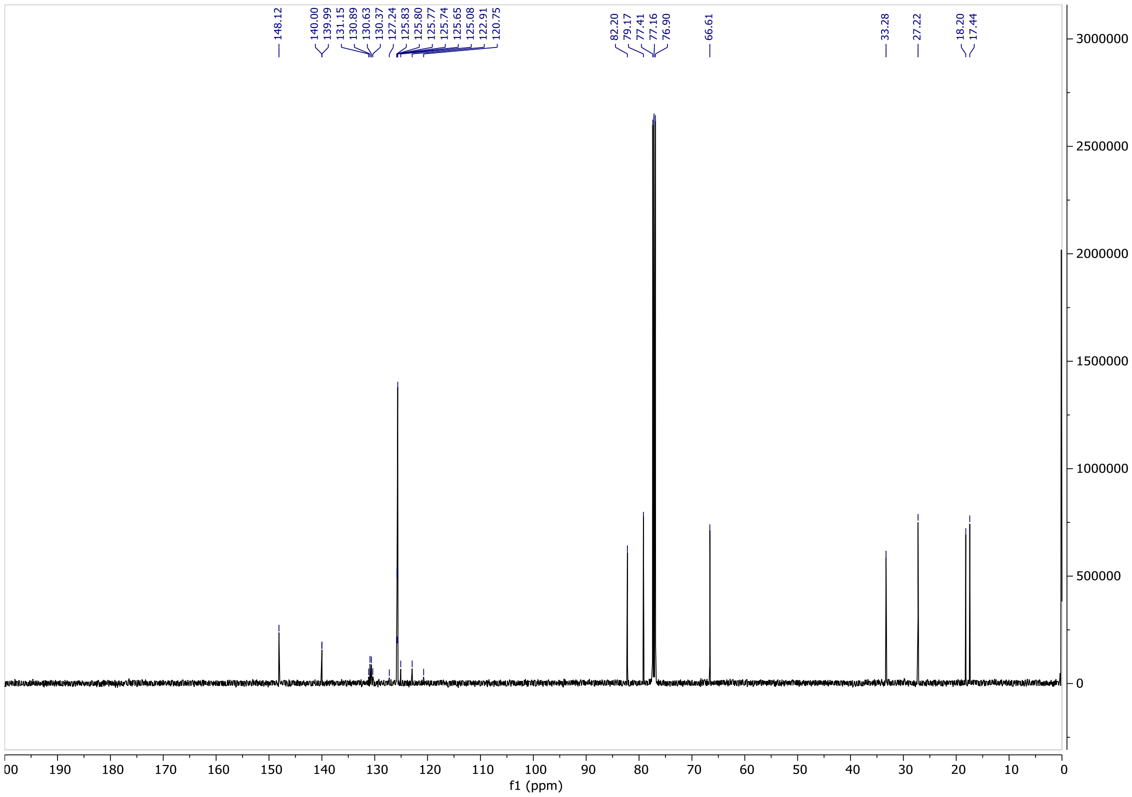

148.12 140.00 139.99 131.15 130.89 130.63 130.37 127.24 125.83 125.80 125.77 125.74 125.65 125.08 122.91 120.75
82.20 79.17 77.41 77.16 76.90 66.61
33.28 27.22 18.20 17.44
f1 (ppm)

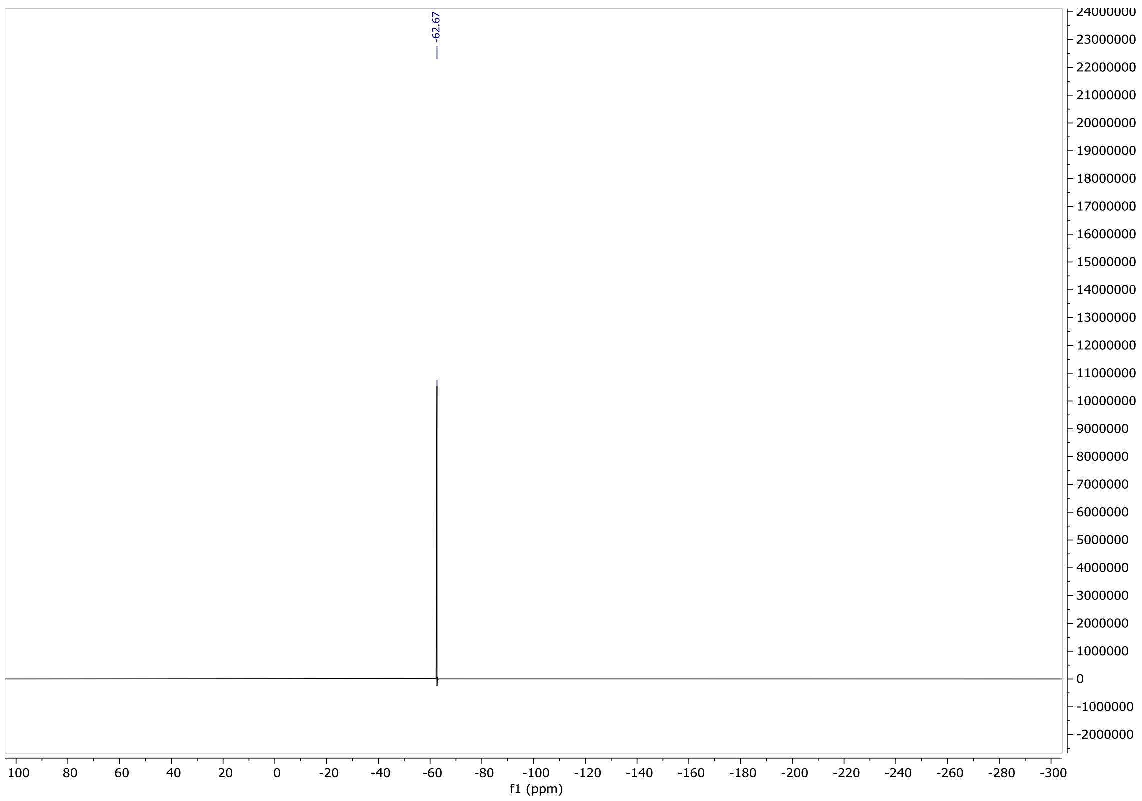
-62.67
f1 (ppm)

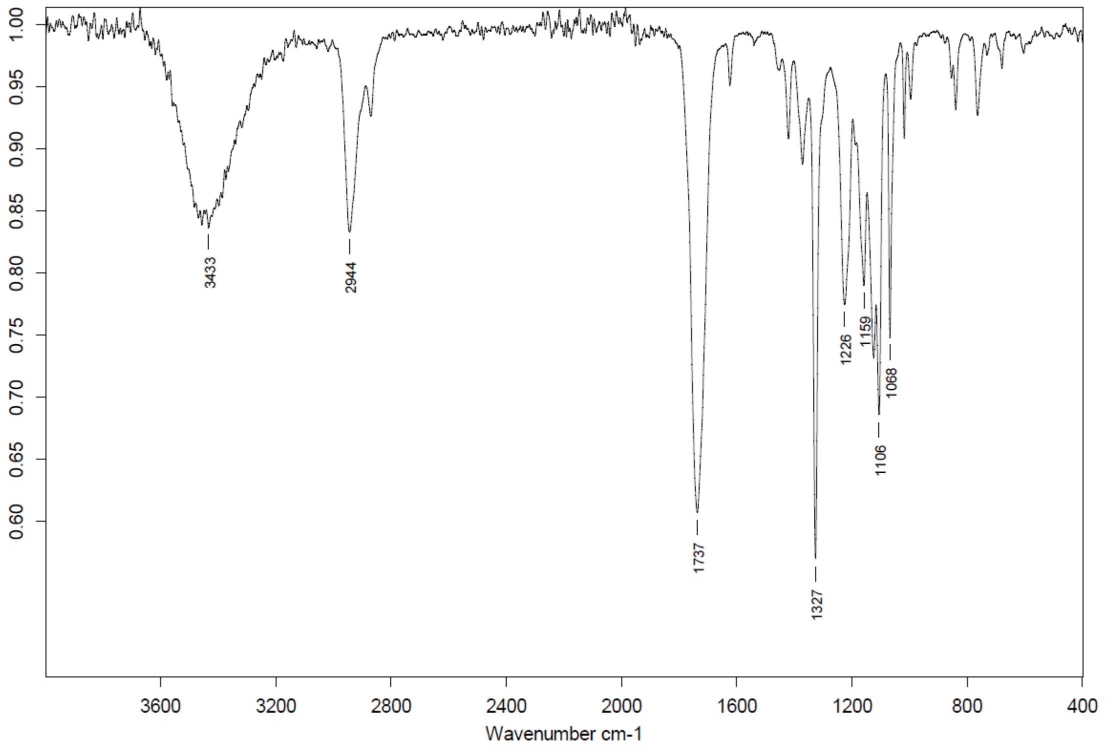
3433
2944
1737
1327
1226
1159
1106
1068
Wavenumber cm-1

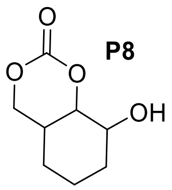

P8

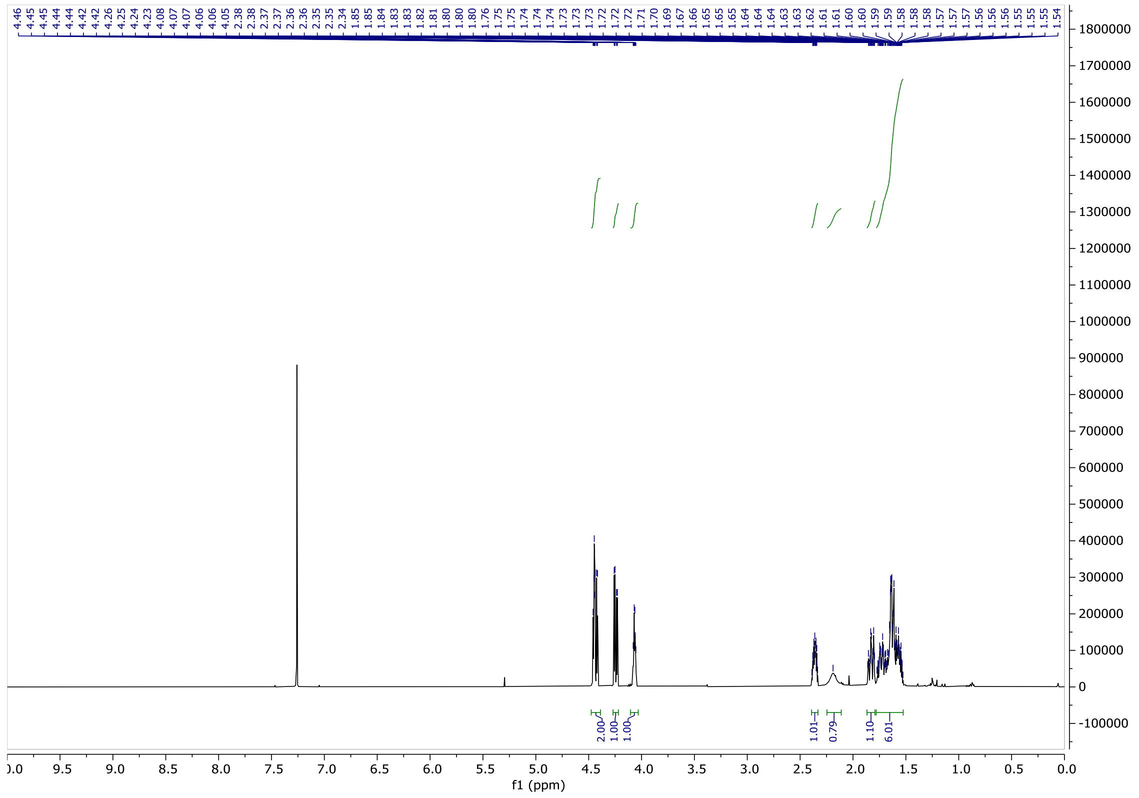

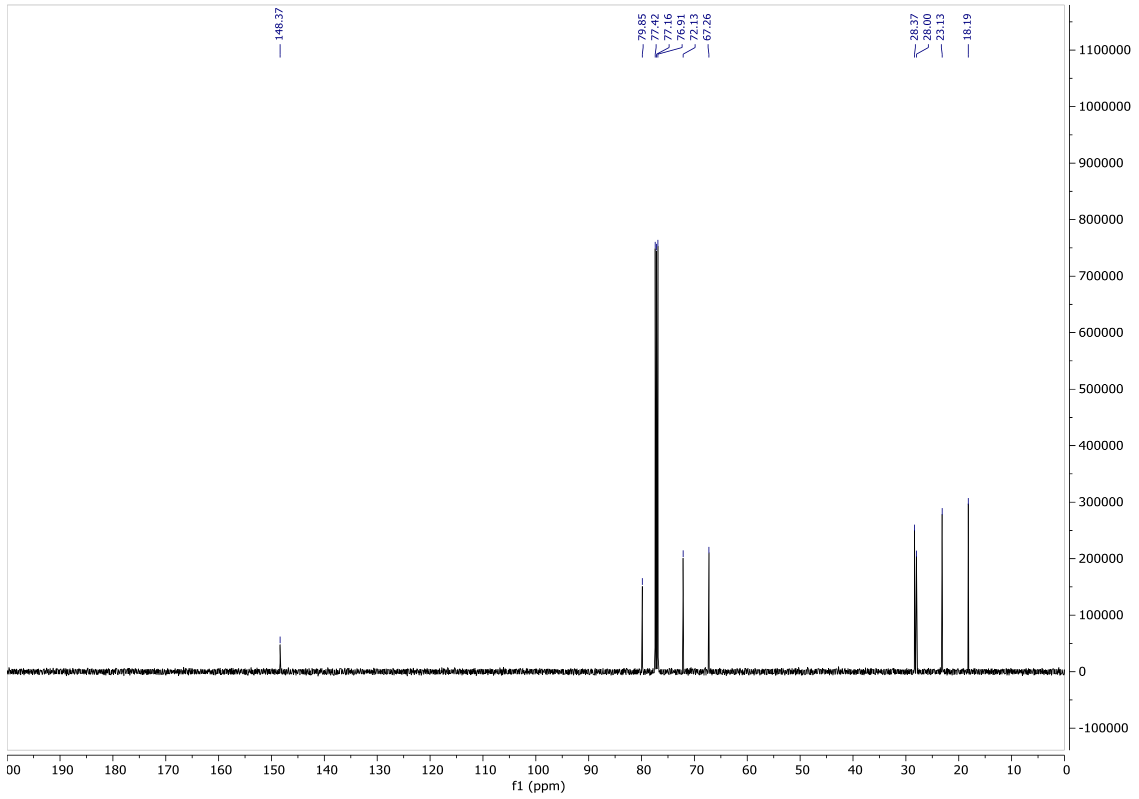

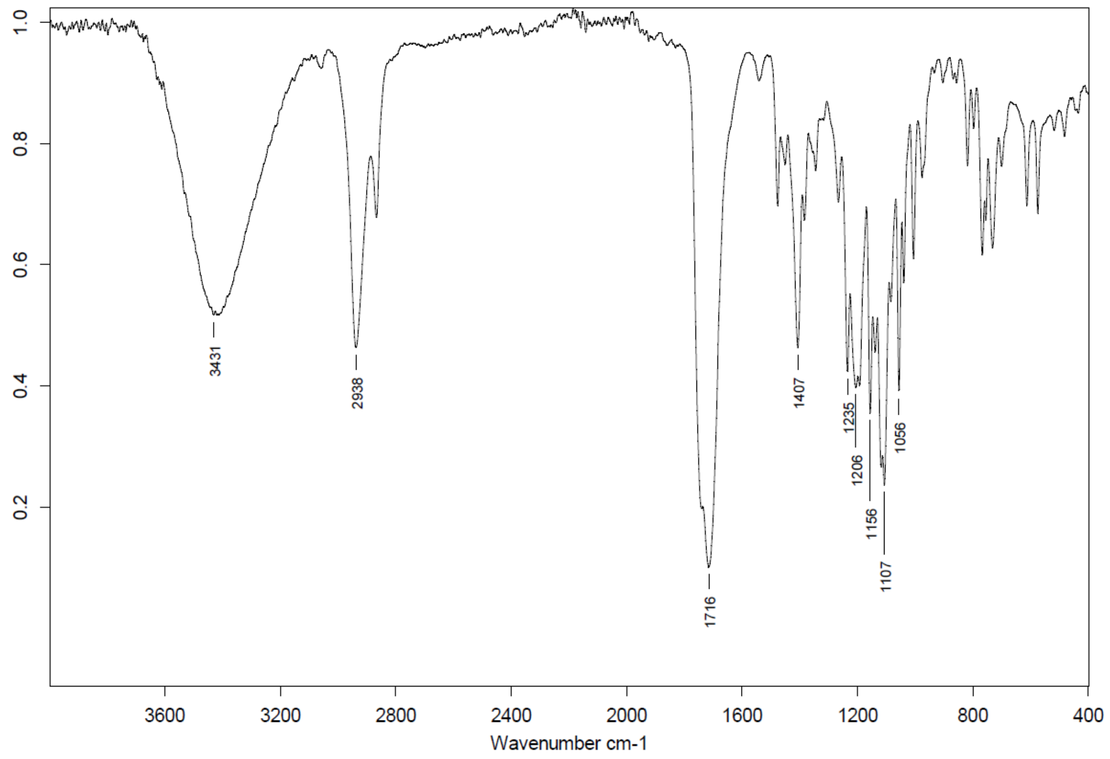
1.0
0.8
0.6
0.4
0.2
3431
2938
1716
1407
1235
1206
1156
1107
1056
3600
3200
2800
2400
2000
1600
1200
800
400
Wavenumber cm-1

P9

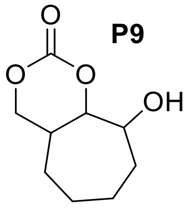
O
O
O
OH

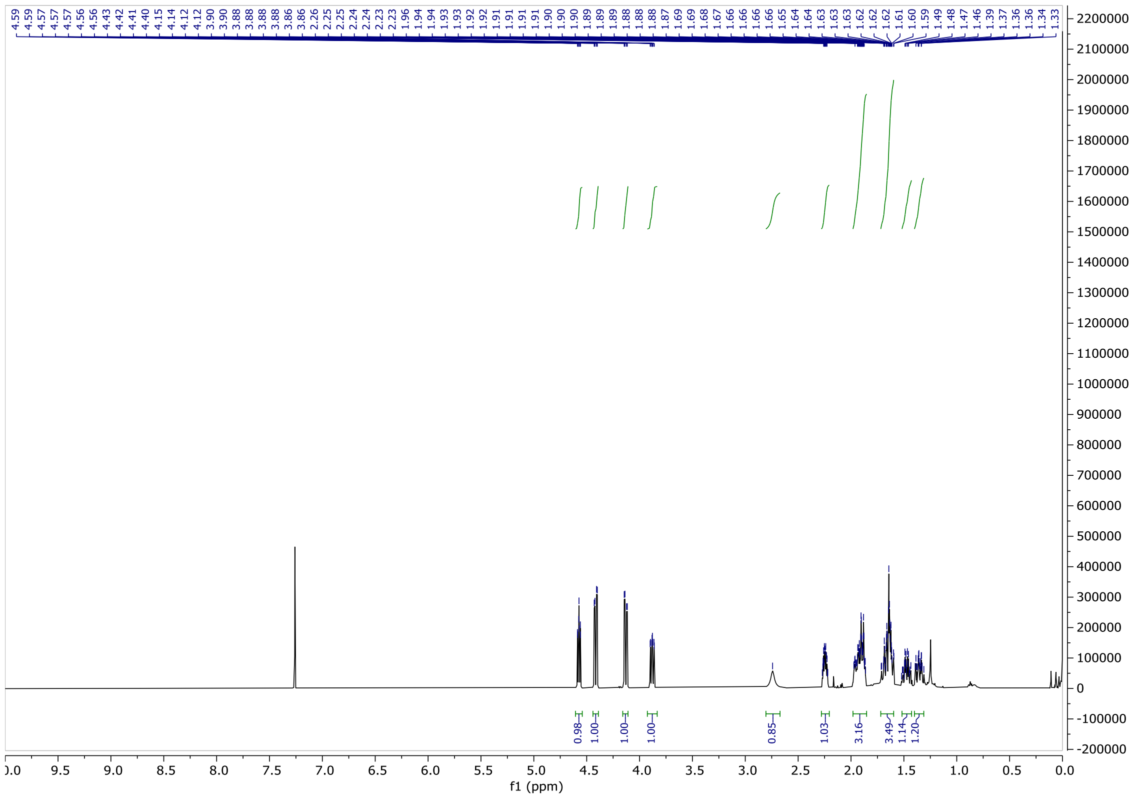
4.59 4.59 4.57 4.57 4.57 4.56 4.56 4.43 4.42 4.41 4.40 4.15 4.14 4.12 4.12 3.90 3.90 3.88 3.88 3.88 3.88 3.86 3.86 2.26 2.25 2.25 2.24 2.24 2.23 2.23 1.96 1.94 1.94 1.93 1.93 1.92 1.92 1.91 1.91 1.91 1.91 1.90 1.90 1.90 1.89 1.89 1.89 1.88 1.88 1.88 1.87 1.69 1.69 1.68 1.67 1.66 1.66 1.66 1.66 1.65 1.64 1.64 1.63 1.63 1.63 1.62 1.62 1.62 1.61 1.60 1.59 1.49 1.48 1.47 1.46 1.39 1.37 1.36 1.36 1.34 1.33
0.98 1.00 1.00 1.00 0.85 1.03 3.16 3.49 1.14 1.20
f1 (ppm)

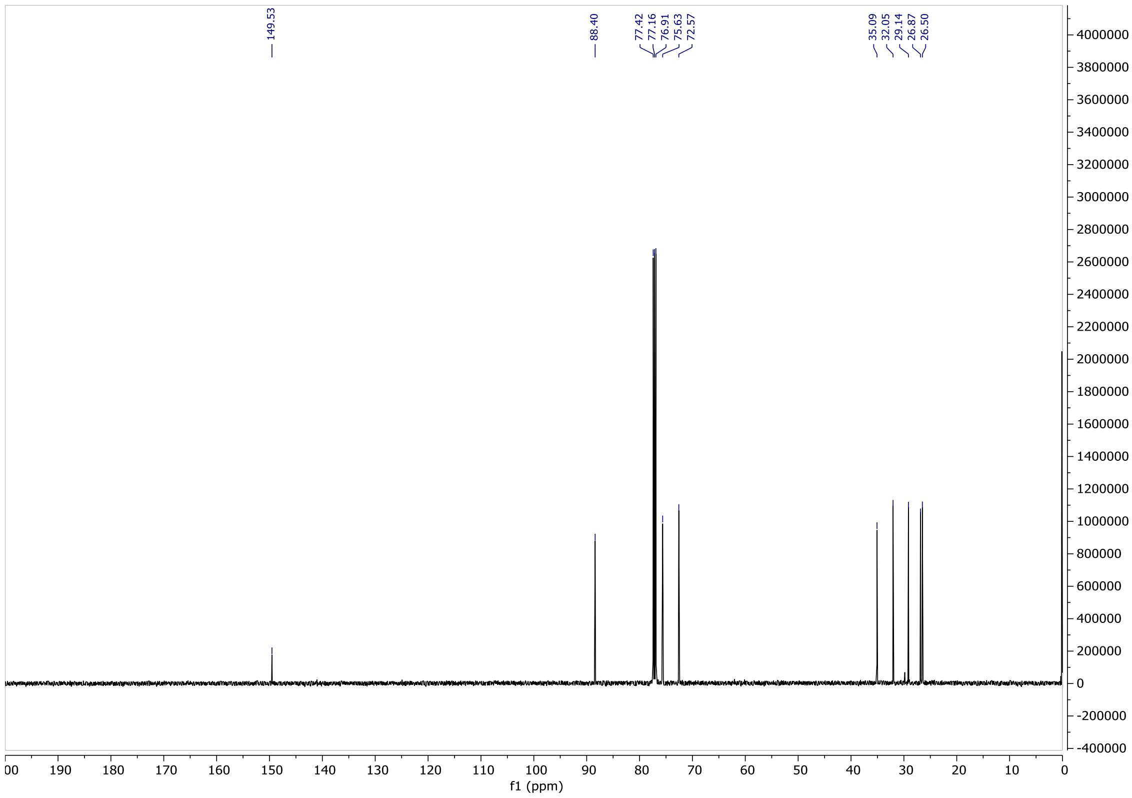
149.53
88.40
77.42 77.16 76.91 75.63 72.57
35.09 32.05 29.14 26.87 26.50
f1 (ppm)

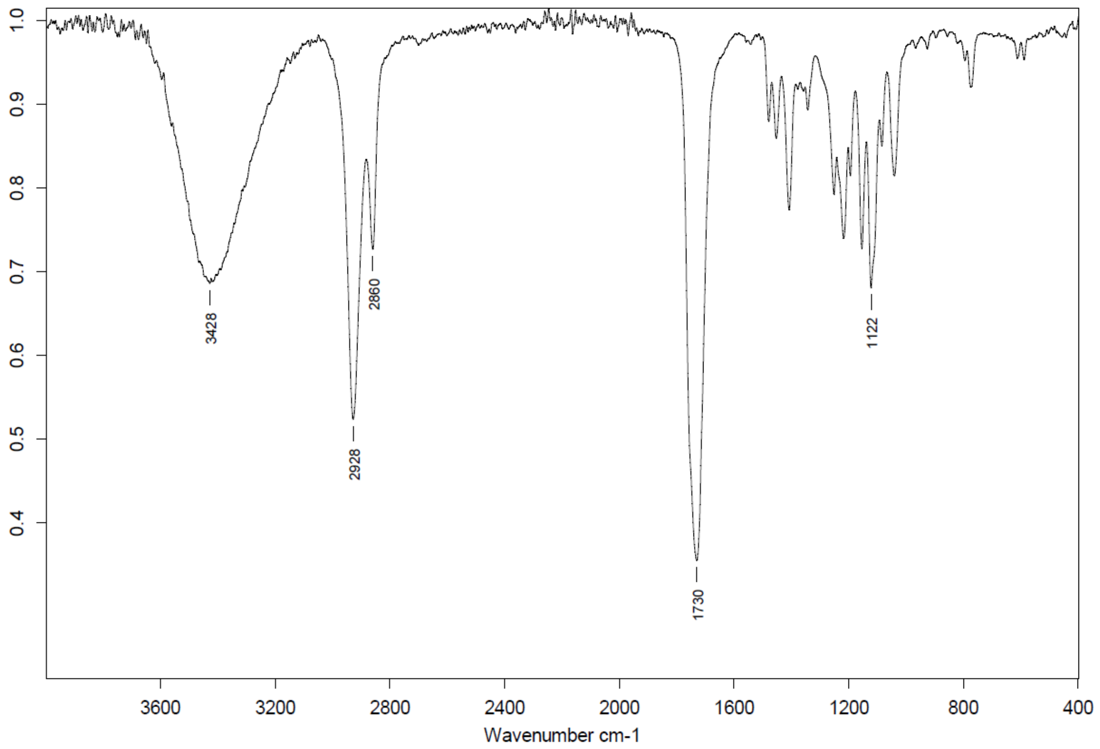

1.0
0.9
0.8
0.7
0.6
0.5
0.4
3428
2928
2860
1730
1122
3600
3200
2800
2400
2000
1600
1200
800
400
Wavenumber cm-1

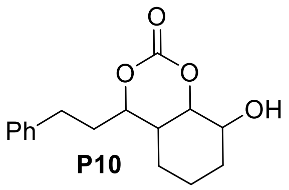
O
O
O
OH
Ph

P10

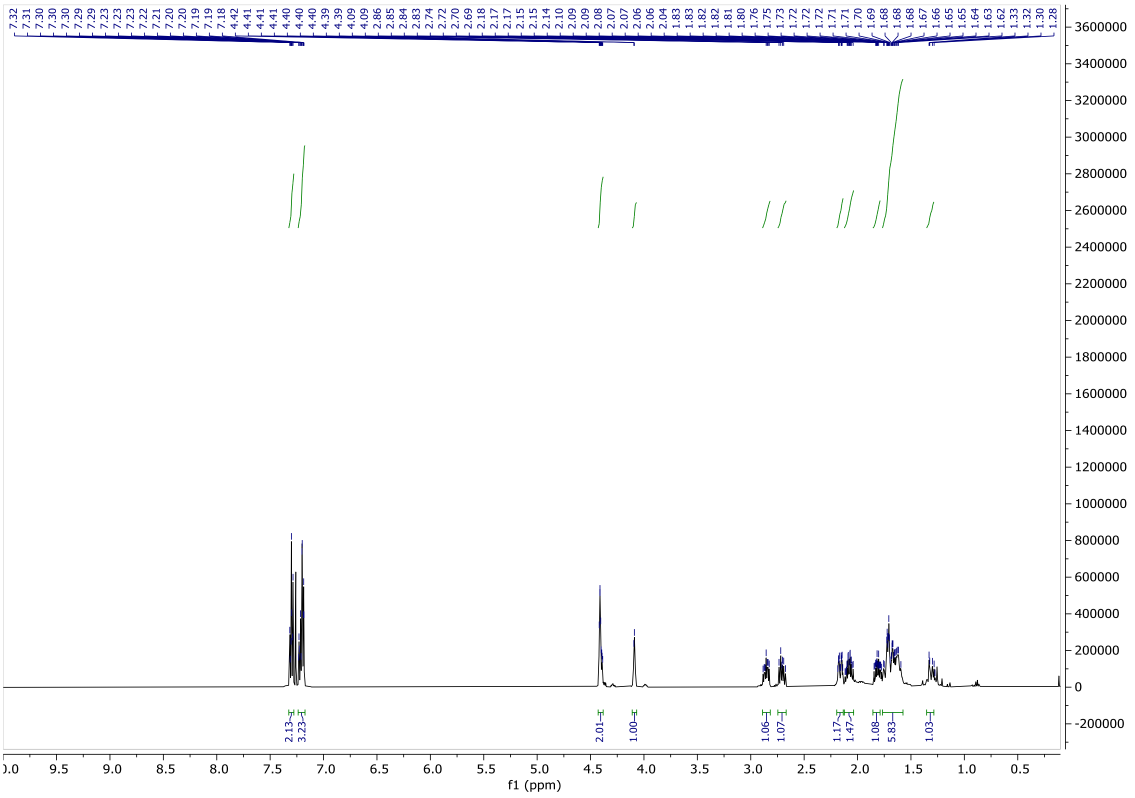
7.32 7.31 7.30 7.30 7.29 7.29 7.23 7.23 7.22 7.21 7.20 7.19 7.19 7.18 4.42 4.41 4.41 4.41 4.40 4.40 4.40 4.39 4.39 4.09 4.09 2.86 2.85 2.84 2.83 2.74 2.72 2.70 2.69 2.18 2.17 2.17 2.15 2.15 2.14 2.10 2.09 2.09 2.08 2.07 2.06 2.06 2.04 1.83 1.83 1.82 1.82 1.81 1.80 1.76 1.75 1.73 1.72 1.72 1.72 1.71 1.71 1.70 1.69 1.68 1.68 1.68 1.67 1.66 1.65 1.65 1.64 1.63 1.62 1.33 1.32 1.30 1.28
2.13
3.23
2.01
1.00
1.06
1.07
1.17
1.47
1.08
5.83
1.03
f1 (ppm)

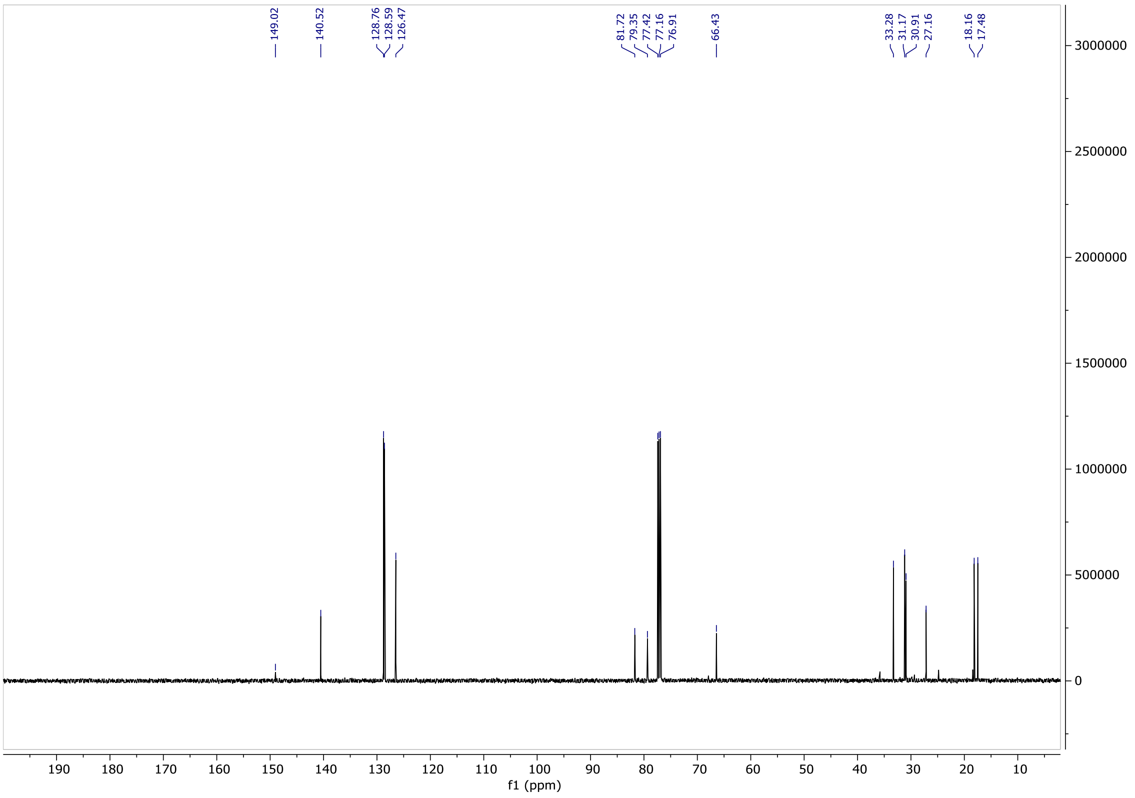
149.02
140.52
128.76
128.59
126.47
81.72
79.35
77.42
77.16
76.91
66.43
33.28
31.17
30.91
27.16
18.16
17.48
f1 (ppm)

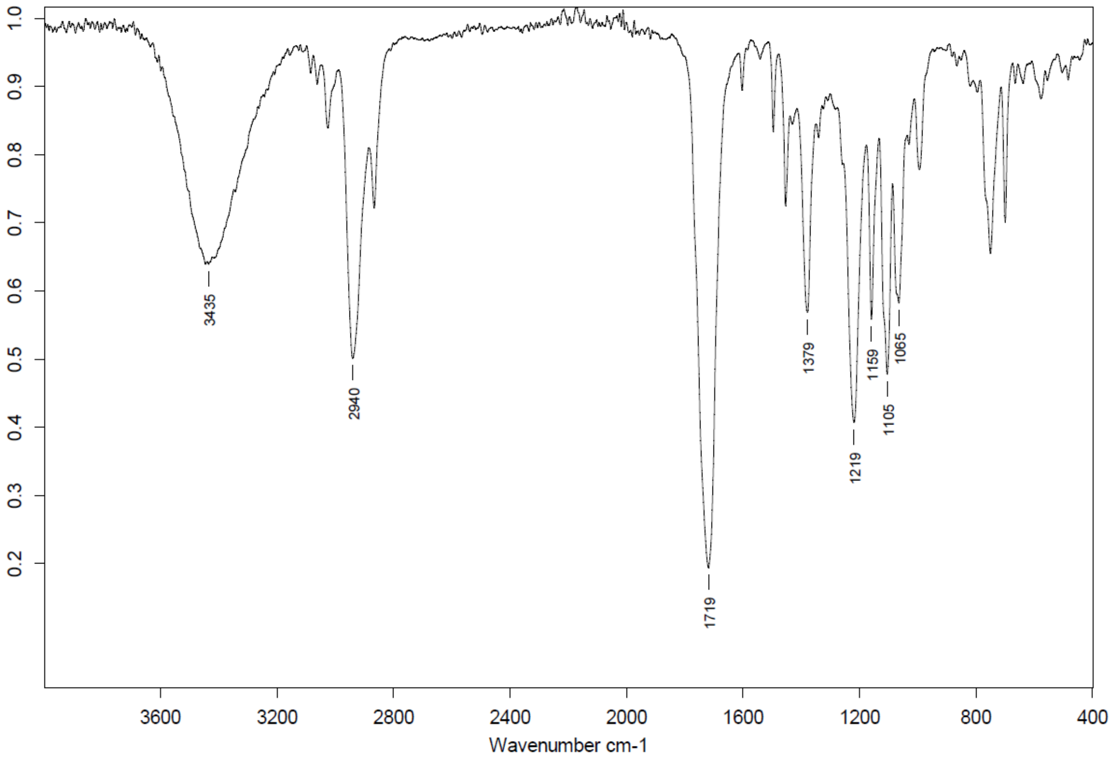

0.2
0.3
0.4
0.5
0.6
0.7
0.8
0.9
1.0
3435
2940
1719
1379
1219
1159
1105
1065
3600
3200
2800
2400
2000
1600
1200
800
400
Wavenumber cm-1

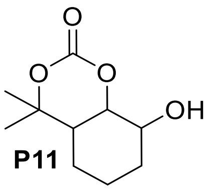
OH

P11

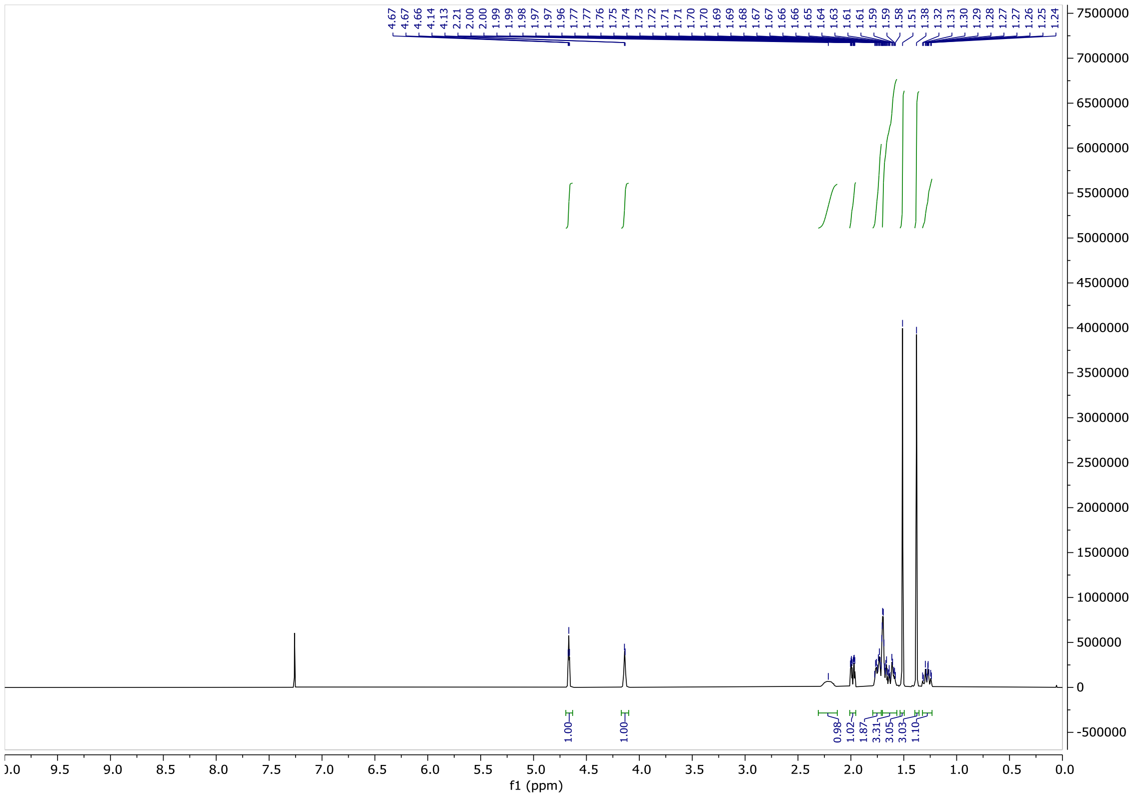
4.67
4.67
4.66
4.14
4.13
2.21
2.00
1.99
1.99
1.98
1.97
1.97
1.96
1.77
1.77
1.76
1.75
1.74
1.73
1.72
1.71
1.71
1.70
1.69
1.69
1.68
1.67
1.67
1.66
1.66
1.65
1.64
1.63
1.61
1.61
1.61
1.59
1.59
1.58
1.51
1.38
1.32
1.31
1.30
1.29
1.28
1.27
1.27
1.26
1.25
1.24
1.00
1.00
0.98
1.02
1.87
3.31
3.05
3.03
1.10
f1 (ppm)

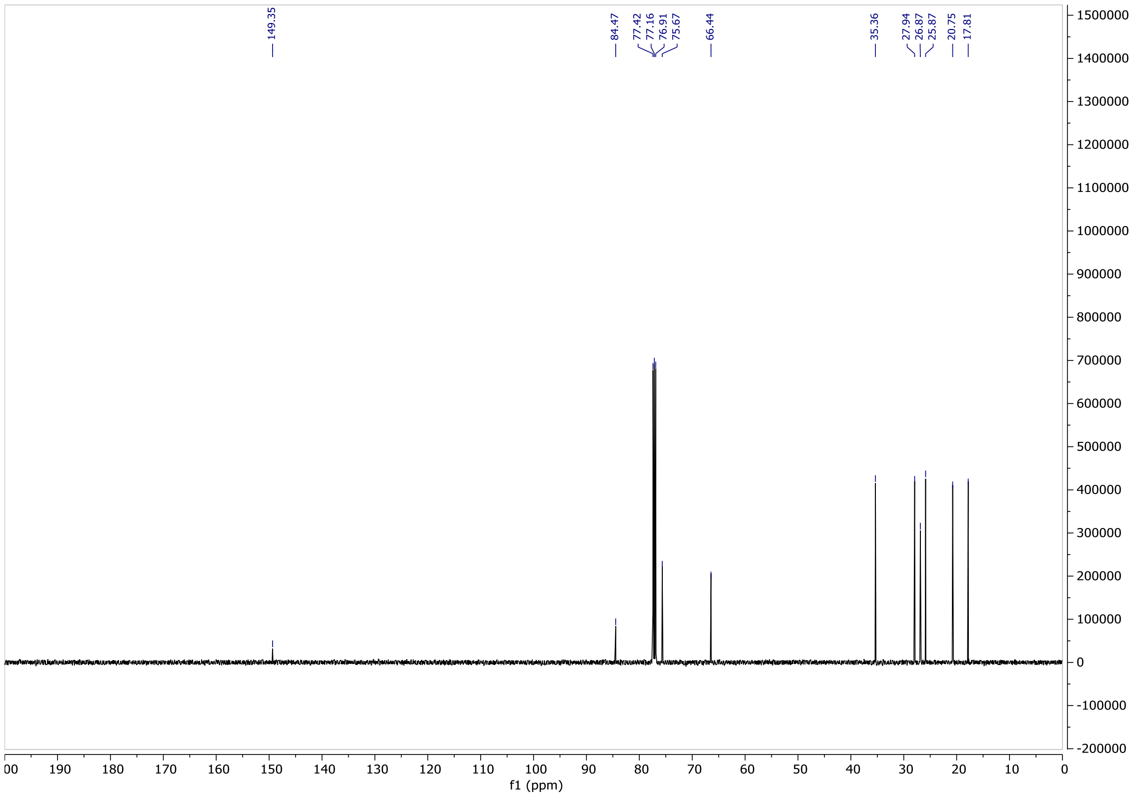
149.35
84.47
77.42
77.16
76.91
75.67
66.44
35.36
27.94
26.87
25.87
20.75
17.81
f1 (ppm)

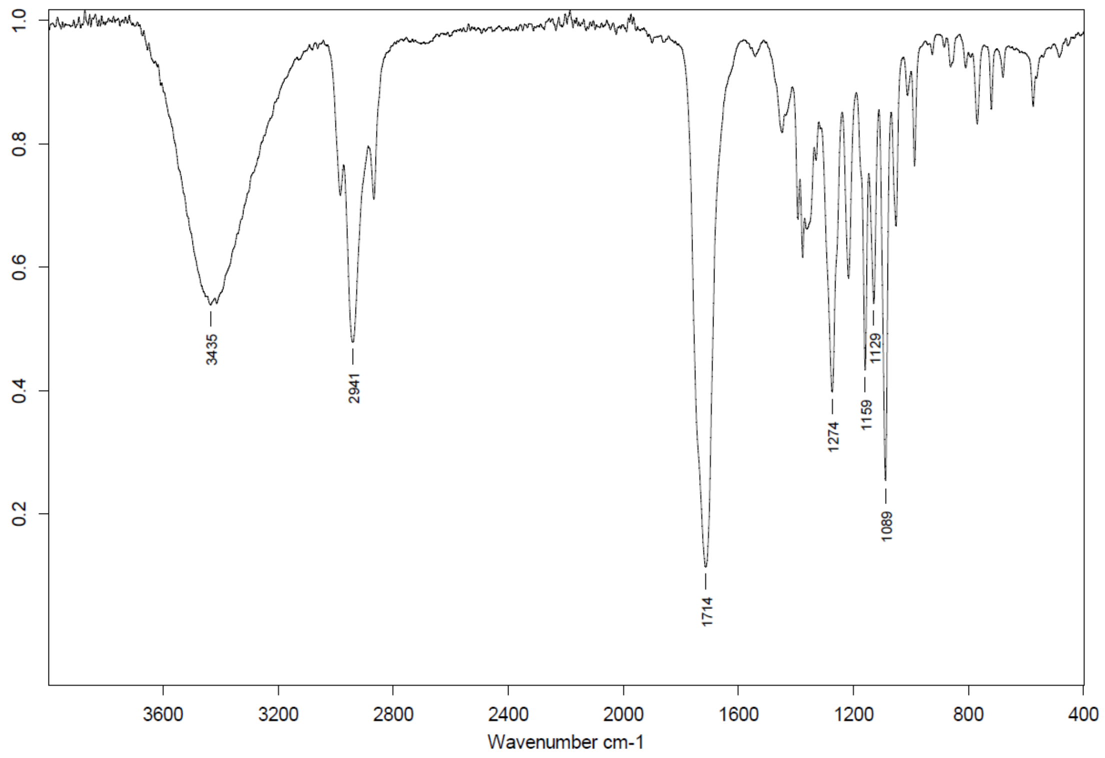
1.0
0.8
0.6
0.4
0.2
3435
2941
1714
1274
1159
1129
1089
3600
3200
2800
2400
2000
1600
1200
800
400
Wavenumber cm-1

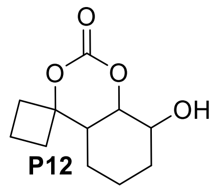
O
O
O
OH

P12

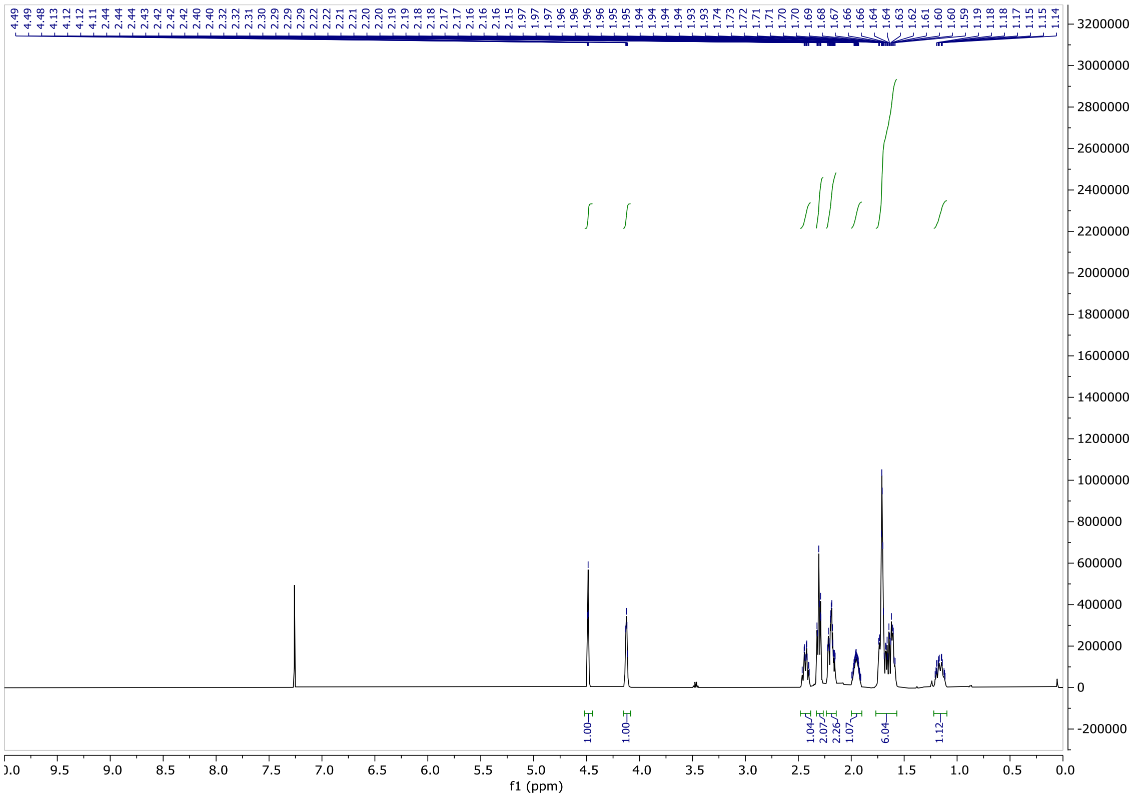
4.49 4.49 4.48 4.13 4.12 4.12 4.11 2.44 2.44 2.43 2.42 2.42 2.42 2.40 2.40 2.32 2.32 2.31 2.30 2.29 2.29 2.29 2.22 2.22 2.21 2.21 2.20 2.20 2.19 2.19 2.18 2.18 2.17 2.17 2.16 2.16 2.16 2.15 1.97 1.97 1.97 1.96 1.96 1.96 1.95 1.95 1.94 1.94 1.94 1.94 1.93 1.93 1.74 1.73 1.72 1.71 1.71 1.70 1.70 1.69 1.68 1.67 1.66 1.66 1.64 1.64 1.63 1.62 1.61 1.60 1.60 1.59 1.19 1.18 1.18 1.17 1.15 1.15 1.14
1.00
1.00
1.04
2.07
2.26
1.07
6.04
1.12
f1 (ppm)

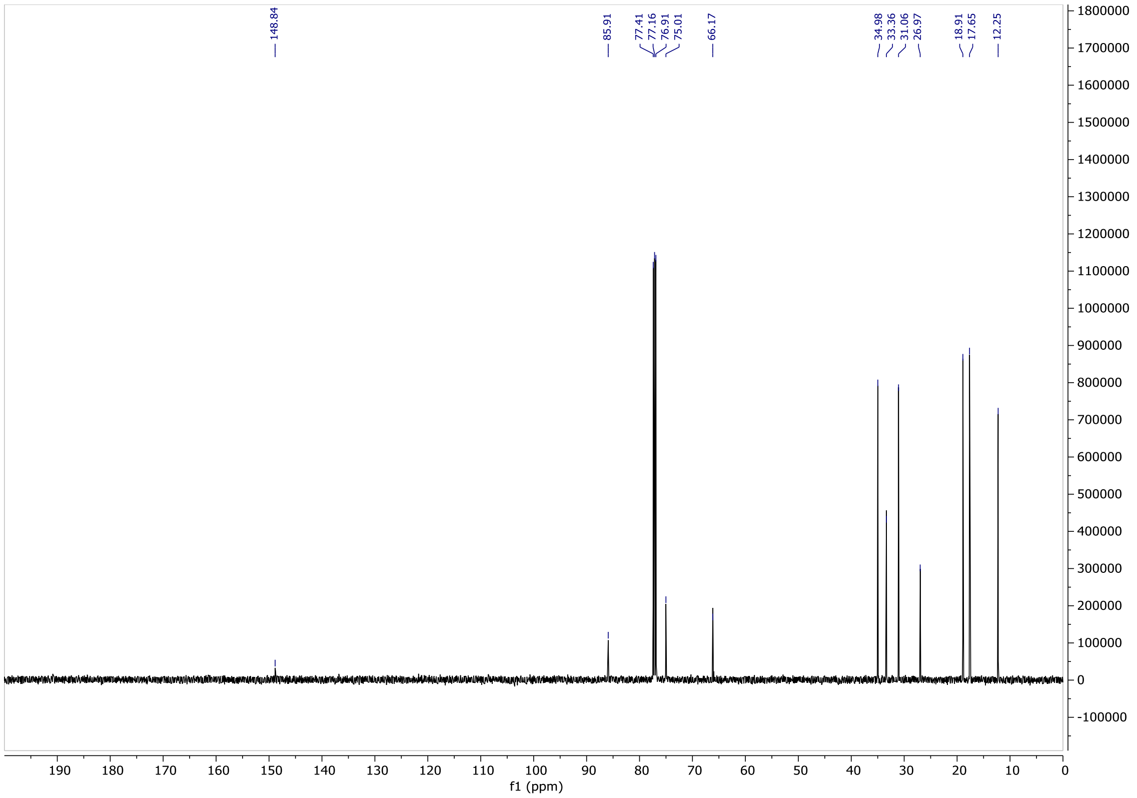
148.84
85.91
77.41
77.16
76.91
75.01
66.17
34.98
33.36
31.06
26.97
18.91
17.65
12.25
f1 (ppm)

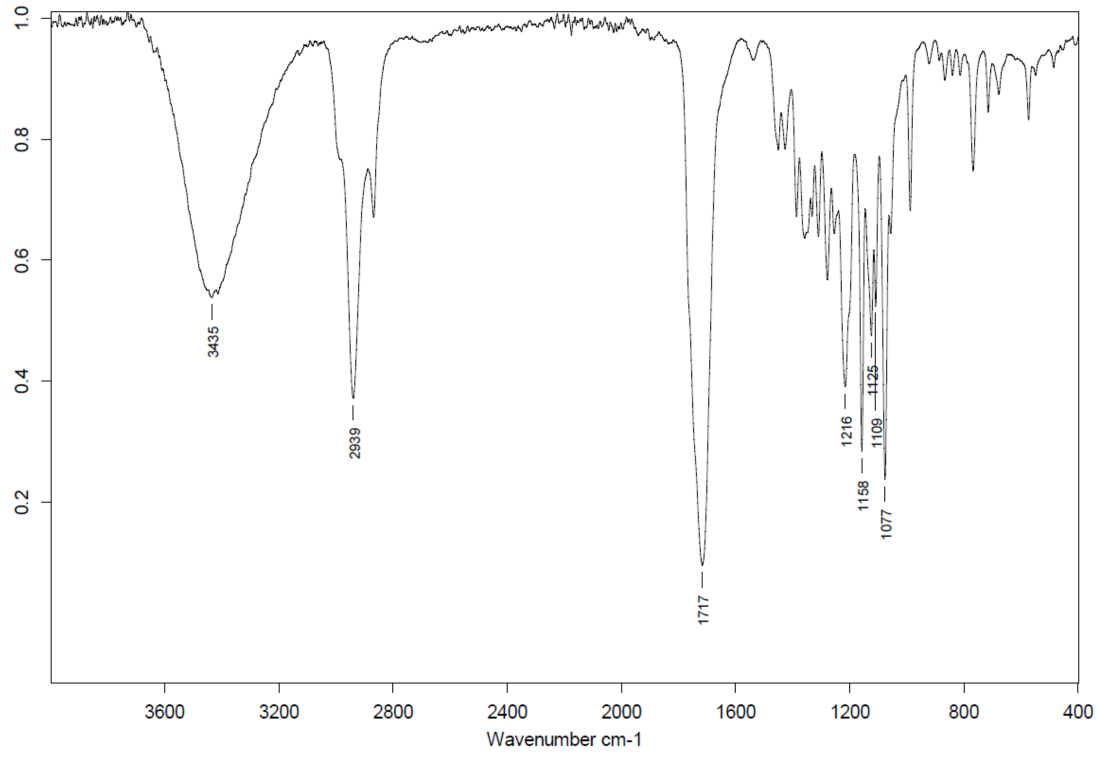

1.0
0.8
0.6
0.4
0.2
3435
2939
1717
1216
1158
1125
1109
1077
3600
3200
2800
2400
2000
1600
1200
800
400
Wavenumber cm-1

P13

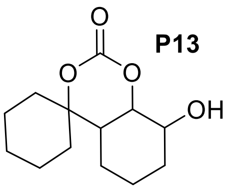

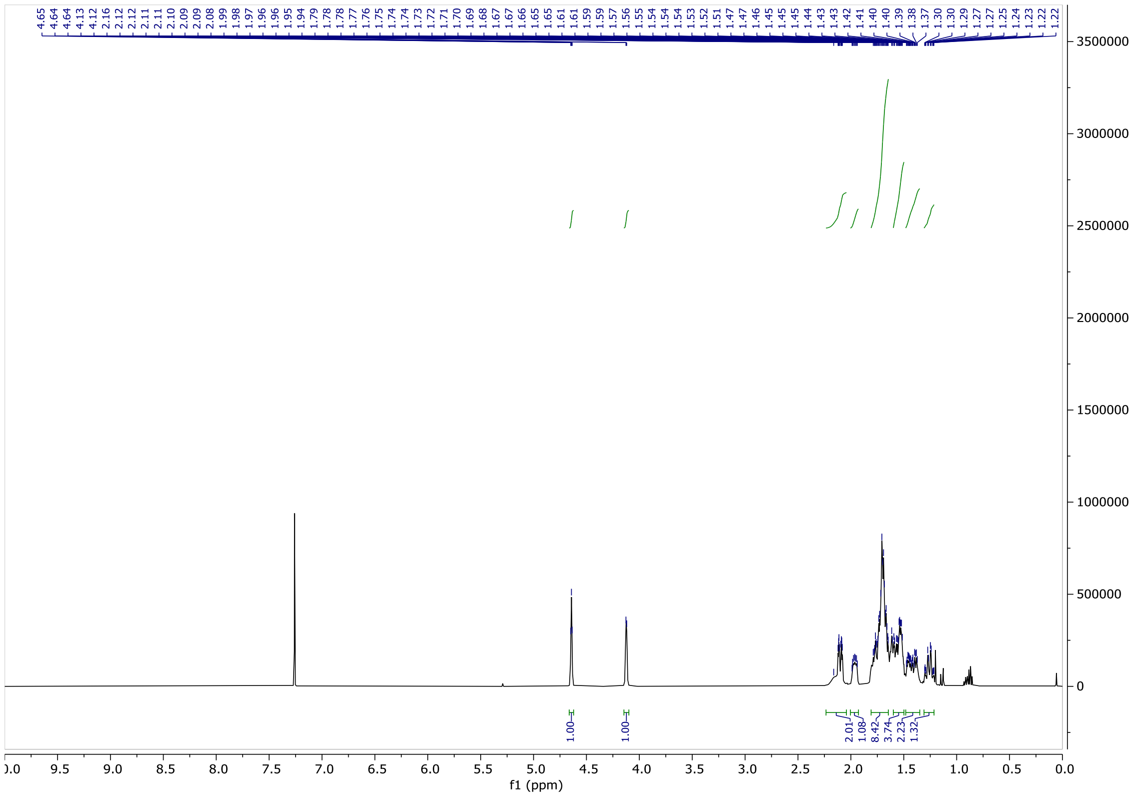
4.65 4.64 4.64 4.13 4.12 2.16 2.12 2.11 2.10 2.09 2.08 1.99 1.98 1.97 1.96 1.95 1.94 1.79 1.78 1.77 1.76 1.75 1.74 1.73 1.72 1.71 1.70 1.69 1.68 1.67 1.67 1.66 1.65 1.65 1.61 1.61 1.59 1.59 1.57 1.56 1.55 1.54 1.54 1.53 1.52 1.51 1.47 1.46 1.45 1.45 1.45 1.44 1.43 1.43 1.42 1.41 1.40 1.40 1.39 1.38 1.37 1.30 1.30 1.29 1.27 1.27 1.25 1.24 1.23 1.22 1.22
1.00
1.00
2.01
1.08
8.42
3.74
2.23
1.32
f1 (ppm)

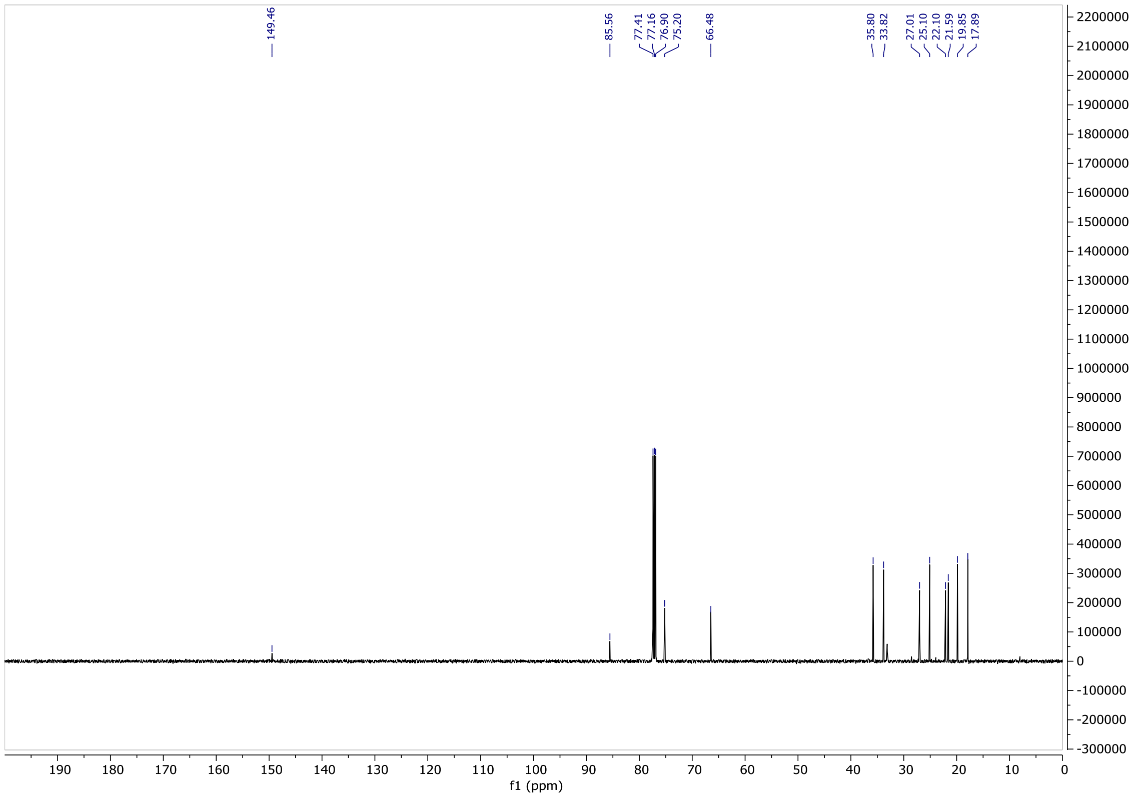
149.46
85.56
77.41
77.16
76.90
75.20
66.48
35.80
33.82
27.01
25.10
22.10
21.59
19.85
17.89
f1 (ppm)

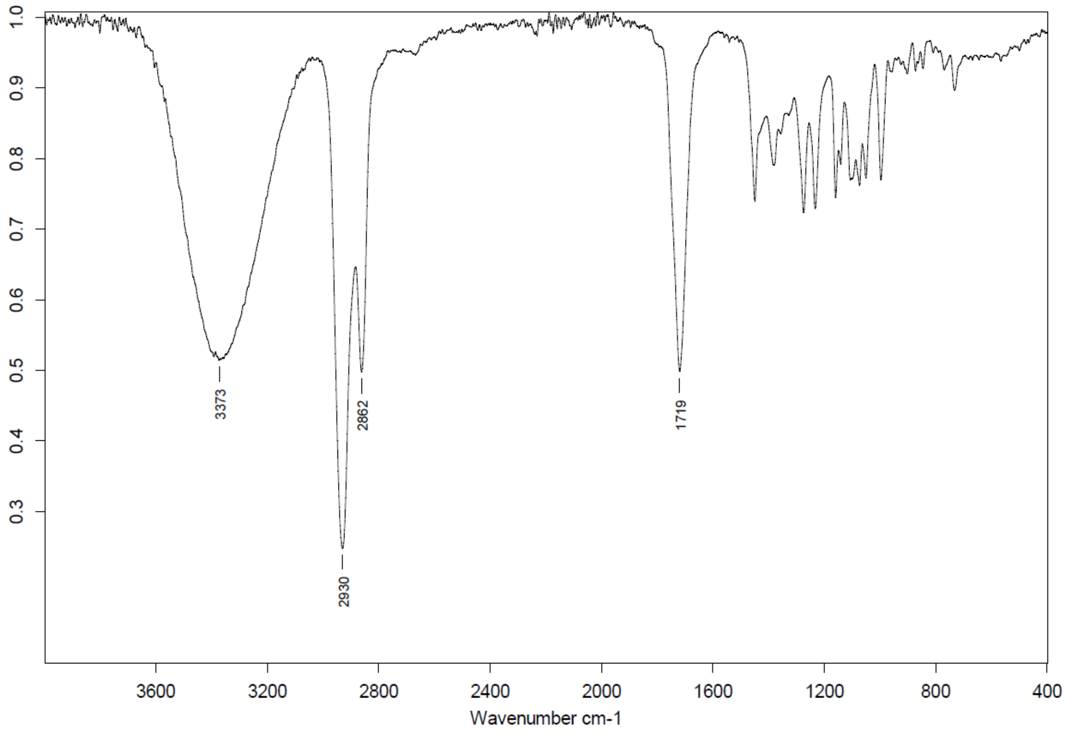
1.0
0.9
0.8
0.7
0.6
0.5
0.4
0.3
3373
2930
2862
1719
3600
3200
2800
2400
2000
1600
1200
800
400
Wavenumber cm-1

P14

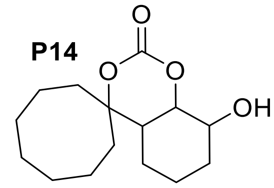
O
O
O
OH

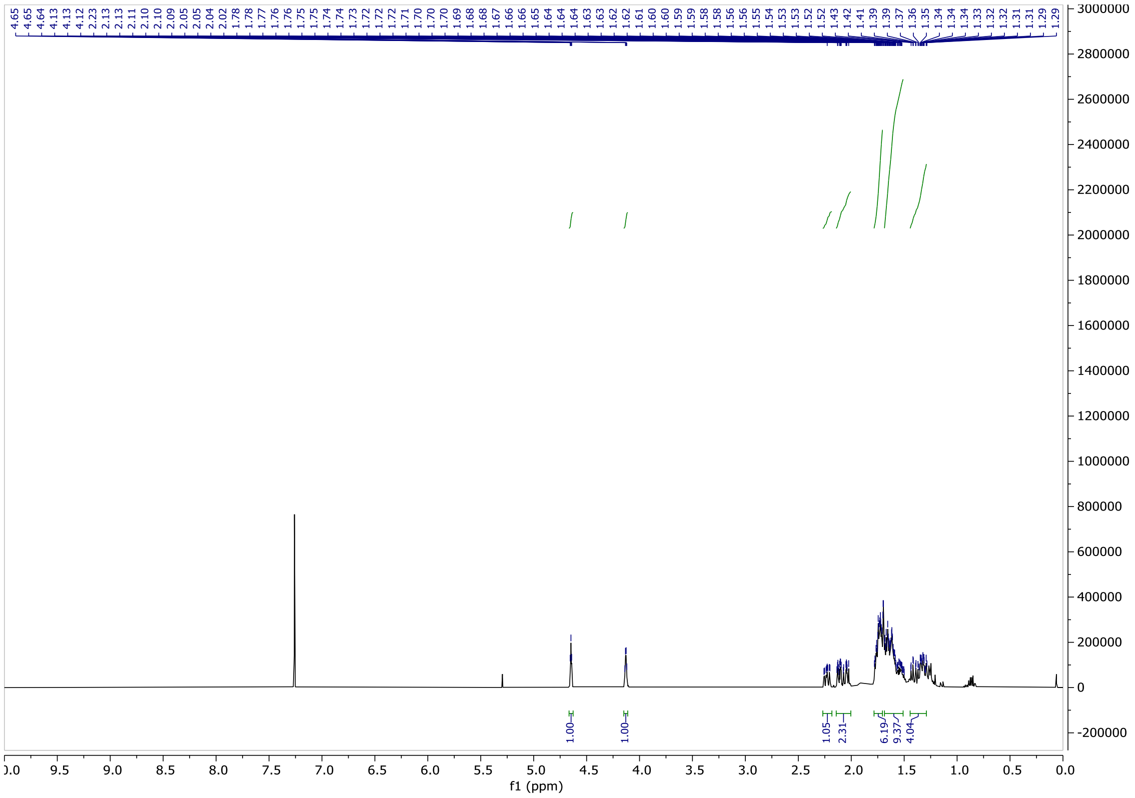
4.65 4.65 4.64 4.13 4.13 4.12 2.23 2.13 2.13 2.11 2.10 2.10 2.09 2.05 2.05 2.04 2.02 1.78 1.78 1.77 1.76 1.76 1.75 1.75 1.74 1.74 1.73 1.73 1.72 1.72 1.72 1.71 1.70 1.70 1.70 1.69 1.68 1.68 1.67 1.66 1.66 1.65 1.64 1.64 1.64 1.63 1.63 1.62 1.62 1.61 1.60 1.60 1.59 1.59 1.58 1.58 1.56 1.56 1.55 1.54 1.53 1.53 1.52 1.52 1.52 1.43 1.42 1.41 1.39 1.39 1.37 1.36 1.35 1.34 1.34 1.34 1.33 1.32 1.31 1.31 1.29
1.00
1.00
1.05
2.31
6.19
9.37
4.04
f1 (ppm)

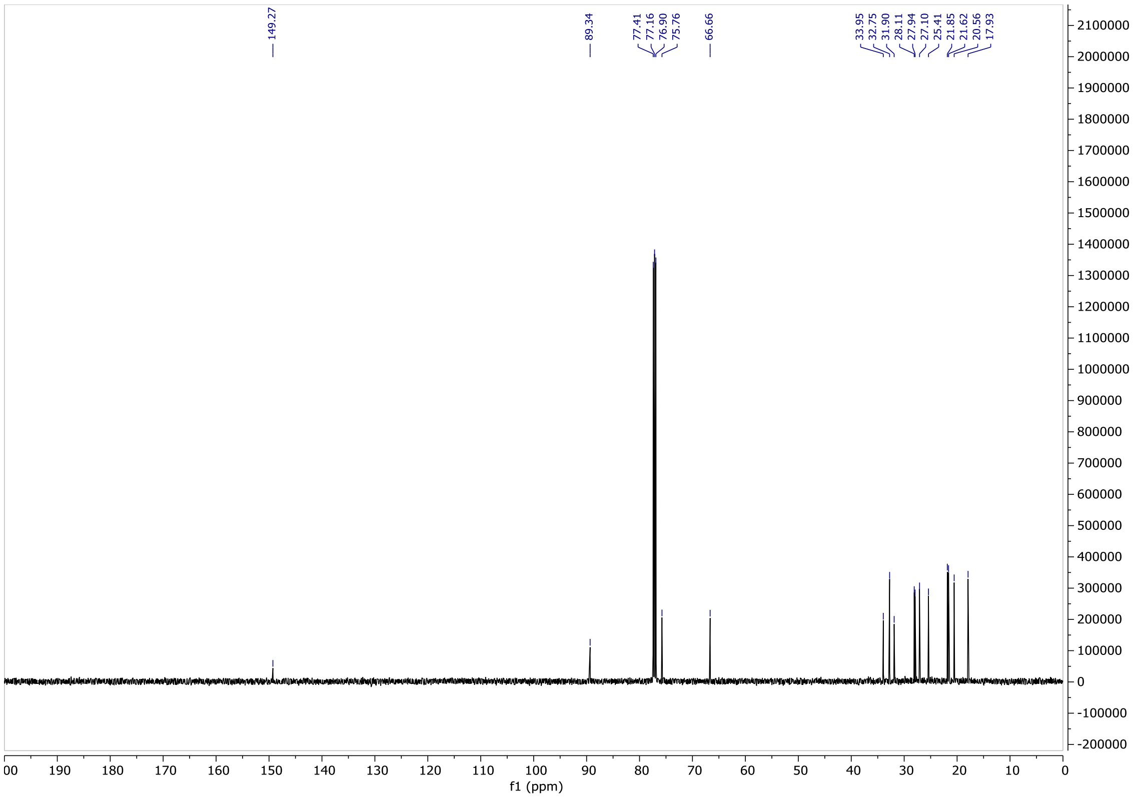
149.27
89.34
77.41 77.16 76.90 75.76
66.66
33.95 32.75 31.90 28.11 27.94 27.10 25.41 21.85 21.62 20.56 17.93
f1 (ppm)

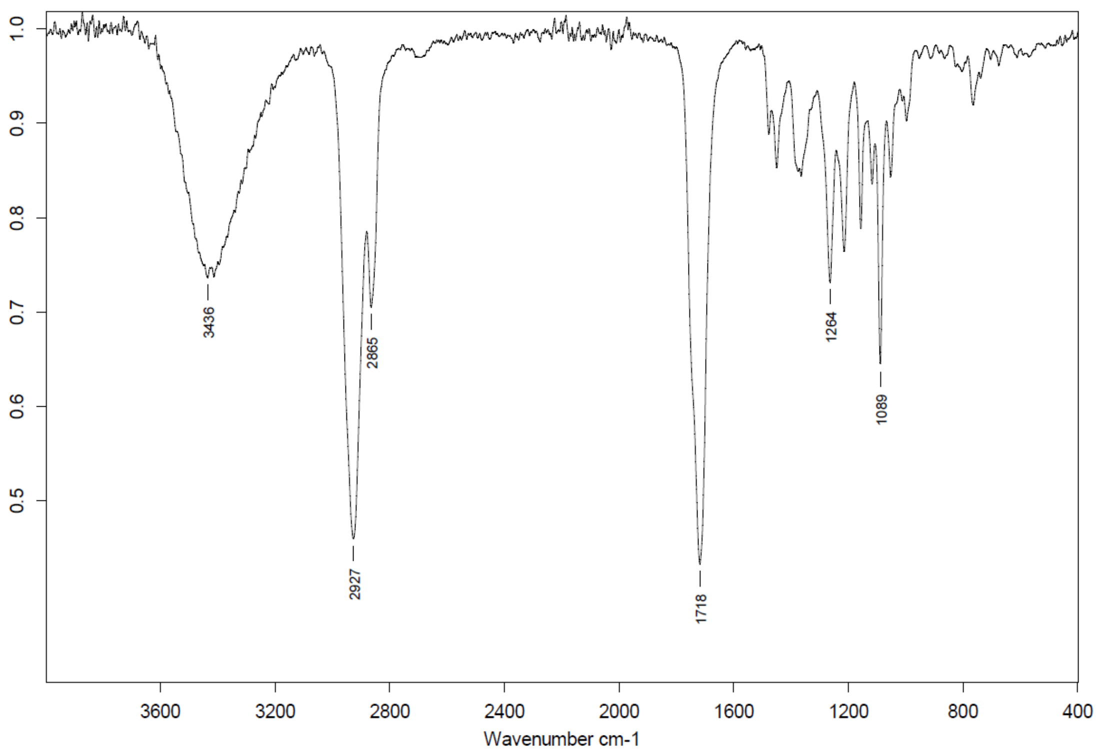

1.0
0.9
0.8
0.7
0.6
0.5
3436
2927
2865
1718
1264
1089
3600
3200
2800
2400
2000
1600
1200
800
400
Wavenumber cm-1

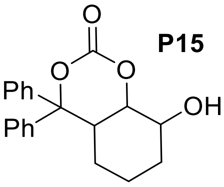
O
O
O
Ph
Ph
OH

P15

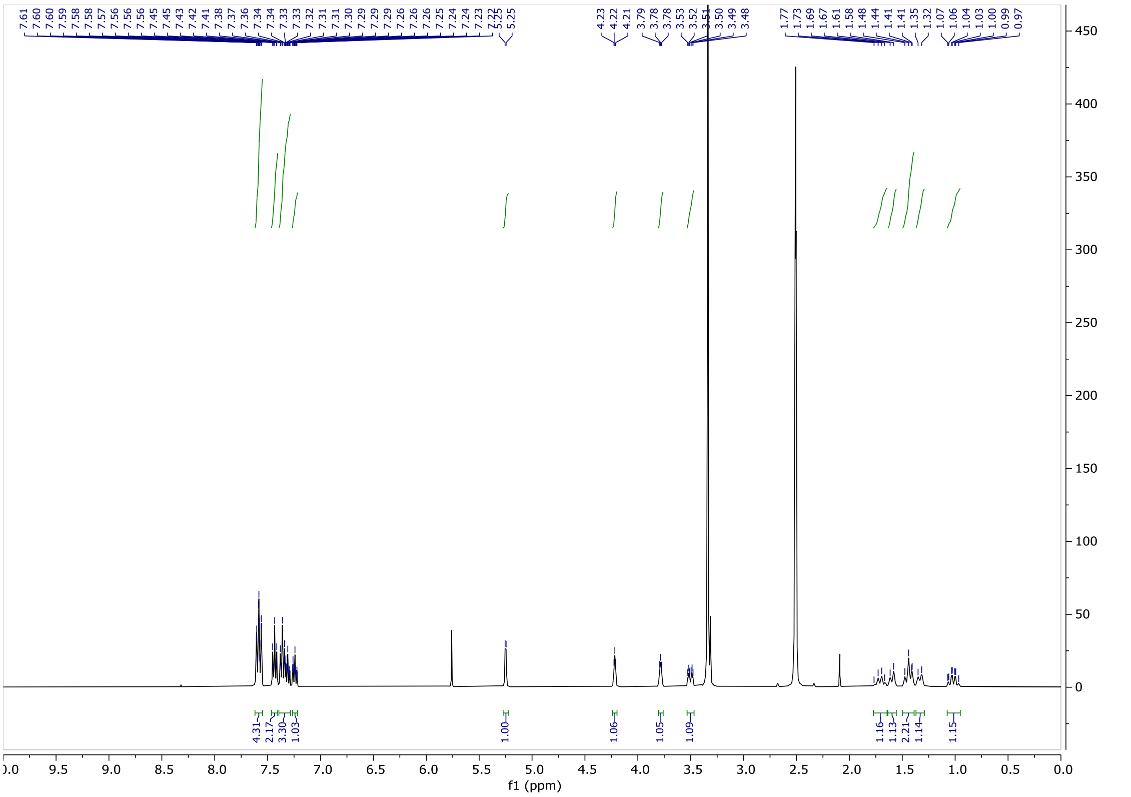
7.61 7.60 7.60 7.59 7.58 7.58 7.57 7.56 7.56 7.56 7.45 7.45 7.43 7.42 7.41 7.38 7.37 7.36 7.34 7.34 7.33 7.33 7.32 7.31 7.30 7.29 7.29 7.29 7.26 7.26 7.25 7.24 7.24 7.23 5.25
4.23 4.22 4.21 3.79 3.78 3.78 3.53 3.52 3.51 3.50 3.49 3.48
1.77 1.73 1.69 1.67 1.61 1.58 1.48 1.44 1.41 1.41 1.35 1.32 1.07 1.06 1.04 1.03 1.00 0.99 0.97
4.31 2.17 3.30 1.03 1.00 1.06 1.05 1.09 1.16 1.13 2.21 1.14 1.15
f1 (ppm)

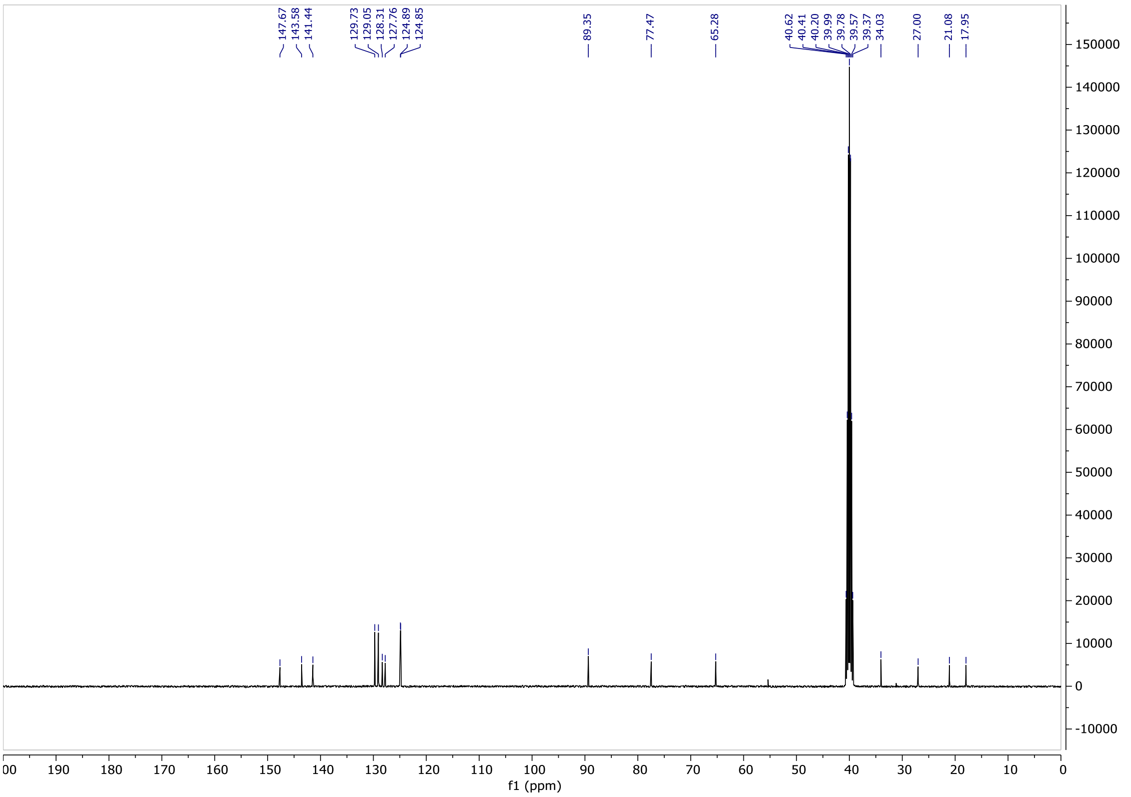
147.67 143.58 141.44 129.73 129.05 128.31 127.76 124.89 124.85 89.35 77.47 65.28 40.62 40.41 40.20 39.99 39.78 39.57 39.37 34.03 27.00 21.08 17.95
f1 (ppm)

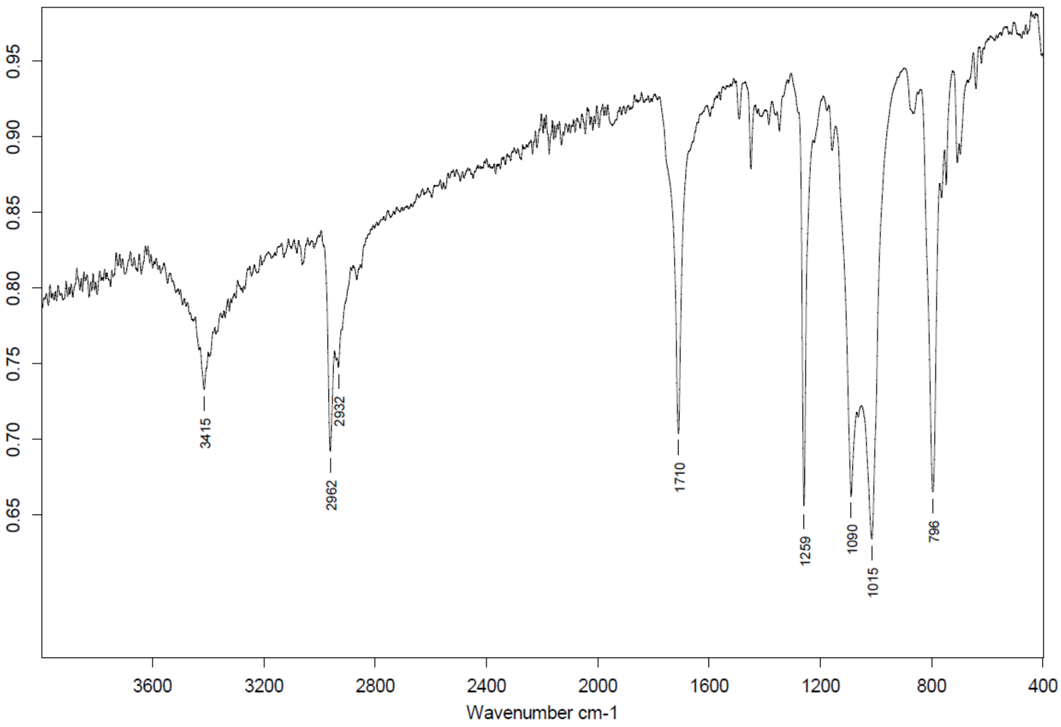
0.95
0.90
0.85
0.80
0.75
0.70
0.65
3415
2962
2932
1710
1259
1090
1015
796
3600
3200
2800
2400
2000
1600
1200
800
400
Wavenumber cm-1

P16

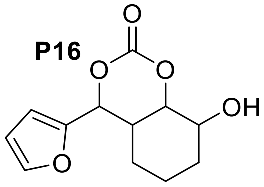

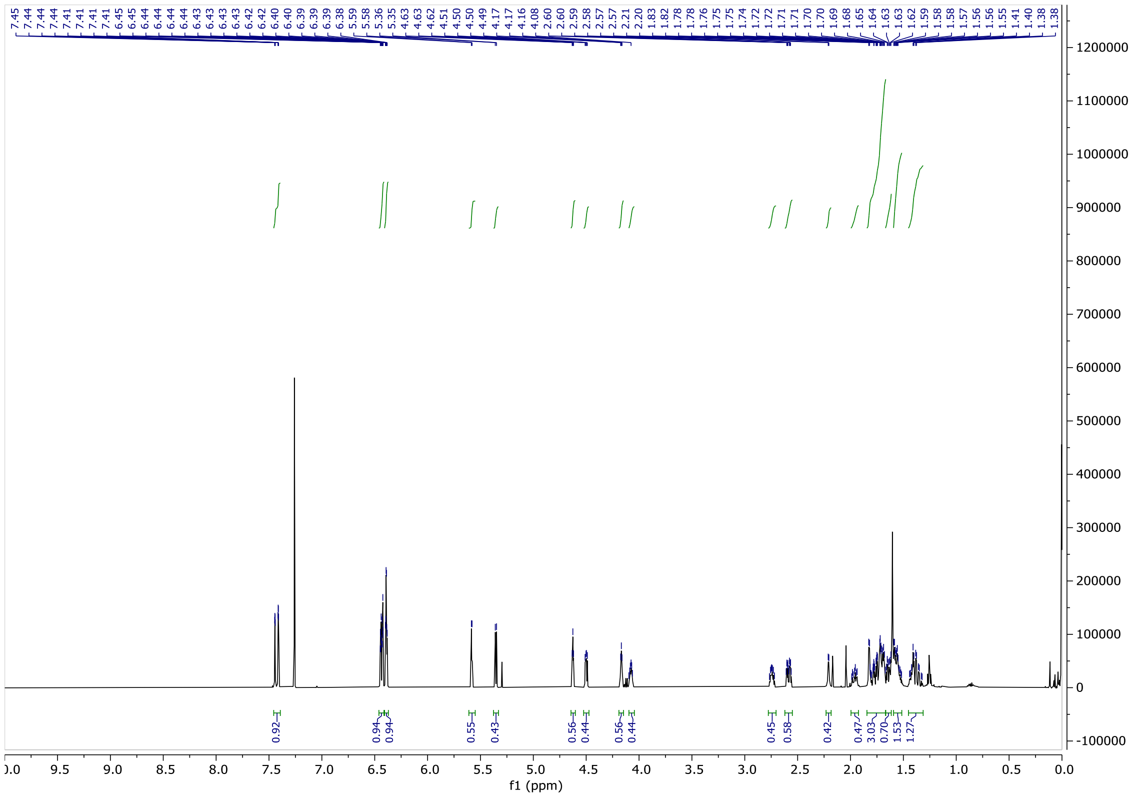

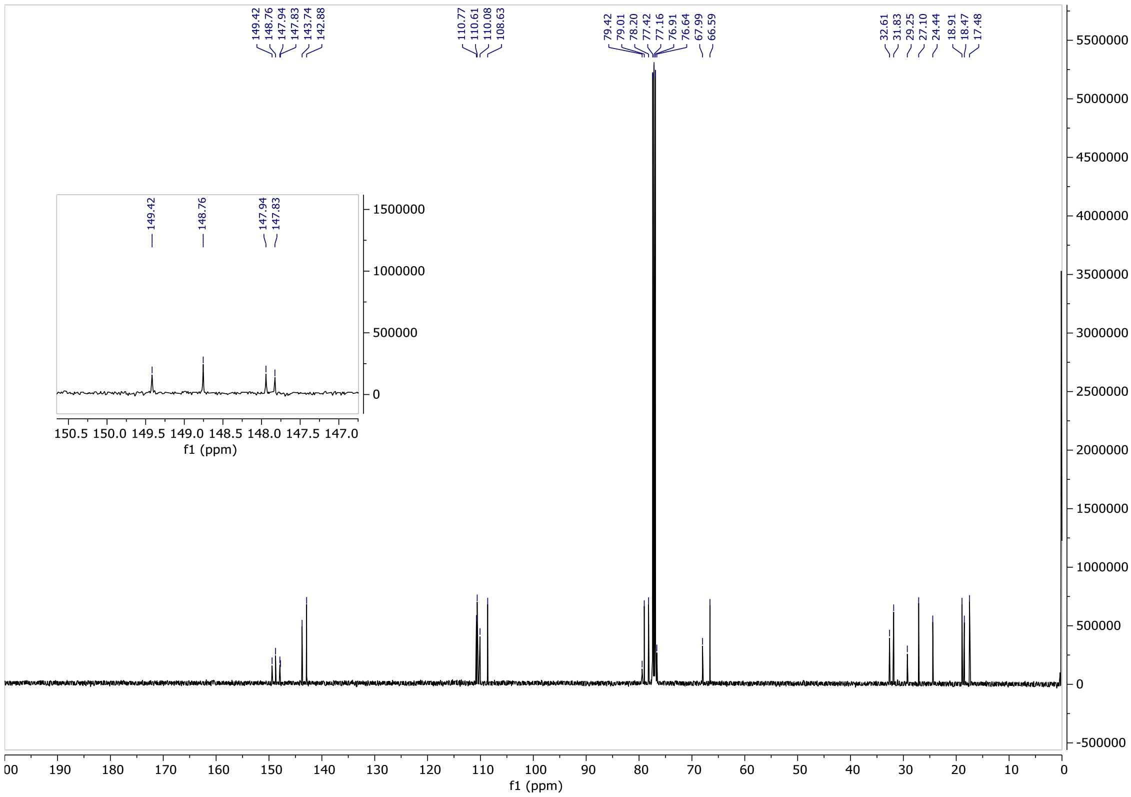

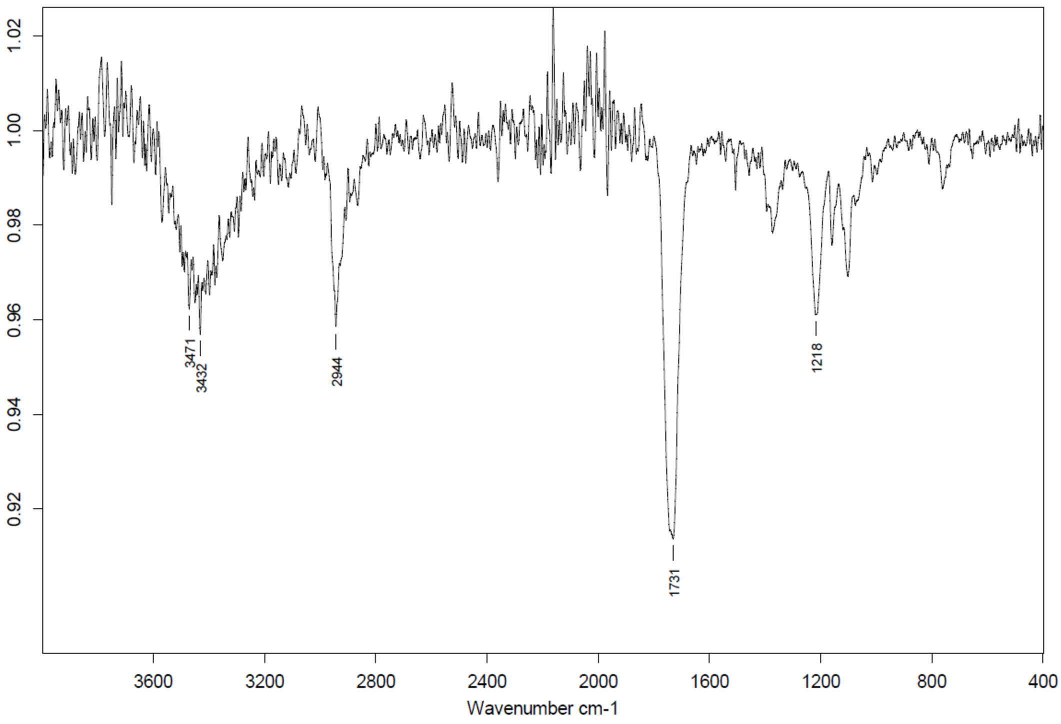
1.02
1.00
0.98
0.96
0.94
0.92
3471
3432
2944
1731
1218
3600
3200
2800
2400
2000
1600
1200
800
400
Wavenumber cm-1

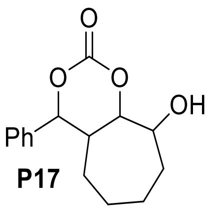
O
O
O
OH
Ph

P17

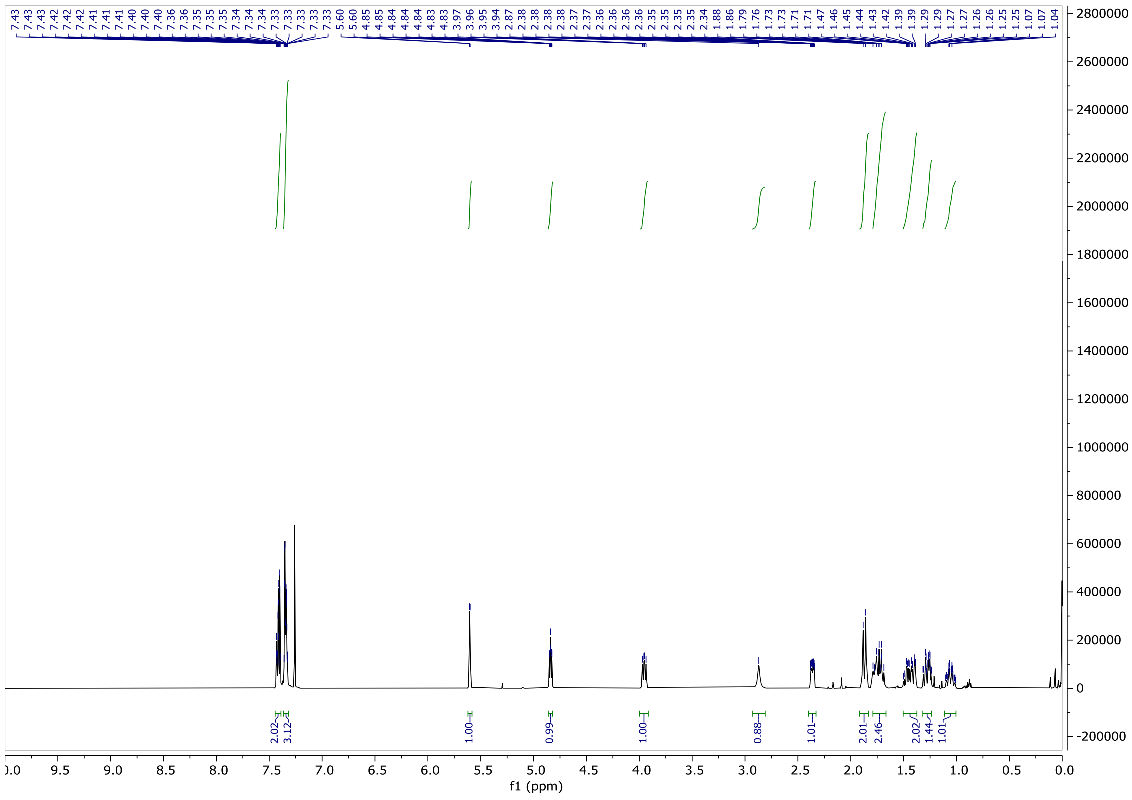
7.43 7.43 7.42 7.42 7.42 7.41 7.41 7.40 7.40 7.36 7.36 7.35 7.35 7.35 7.34 7.34 7.33 7.33 7.33 7.33 7.33 5.60 5.60 4.85 4.84 4.84 4.84 4.83 4.83 3.97 3.96 3.95 3.94 2.87 2.38 2.38 2.38 2.37 2.37 2.36 2.36 2.36 2.35 2.35 2.35 2.34 1.88 1.86 1.79 1.76 1.73 1.73 1.71 1.71 1.47 1.46 1.45 1.44 1.43 1.42 1.39 1.29 1.29 1.27 1.27 1.26 1.26 1.25 1.25 1.07 1.04
2.02 3.12 1.00 0.99 1.00 0.88 1.01 2.01 2.46 2.02 1.44 1.01
f1 (ppm)

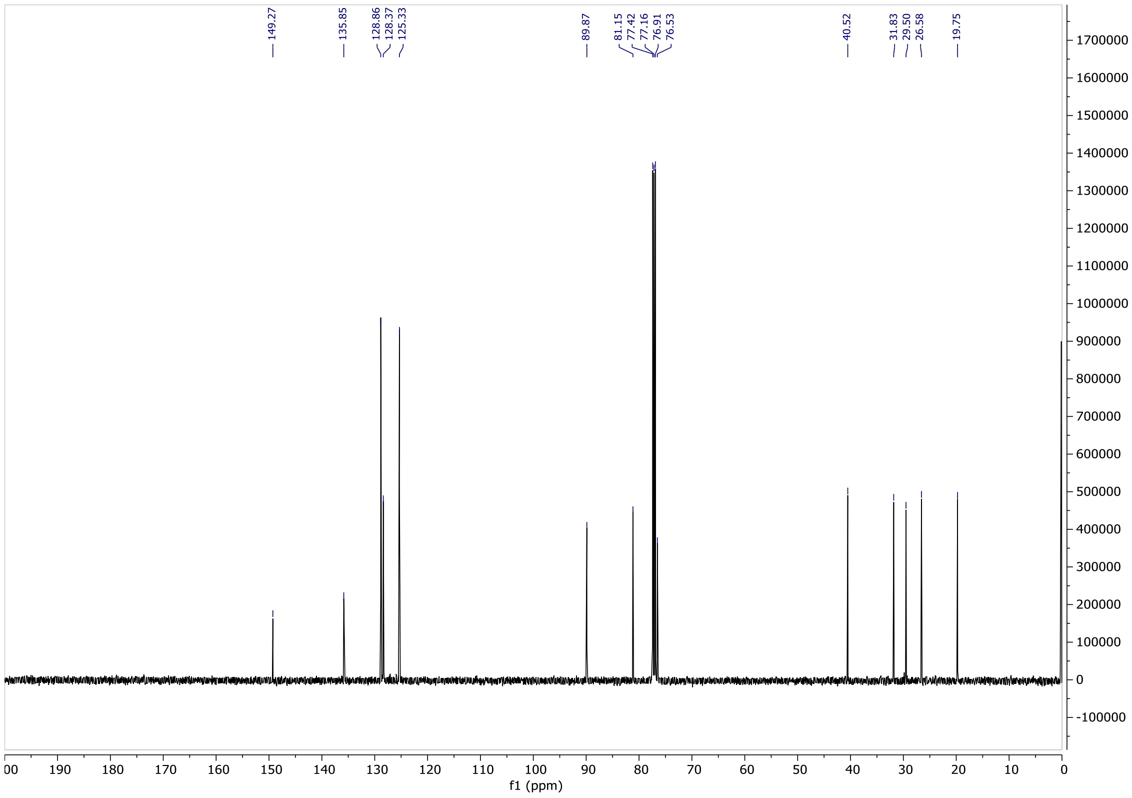
149.27
135.85
128.86 128.37 125.33
89.87
81.15 77.42 77.16 76.91 76.53
40.52
31.83 29.50 26.58
19.75
f1 (ppm)

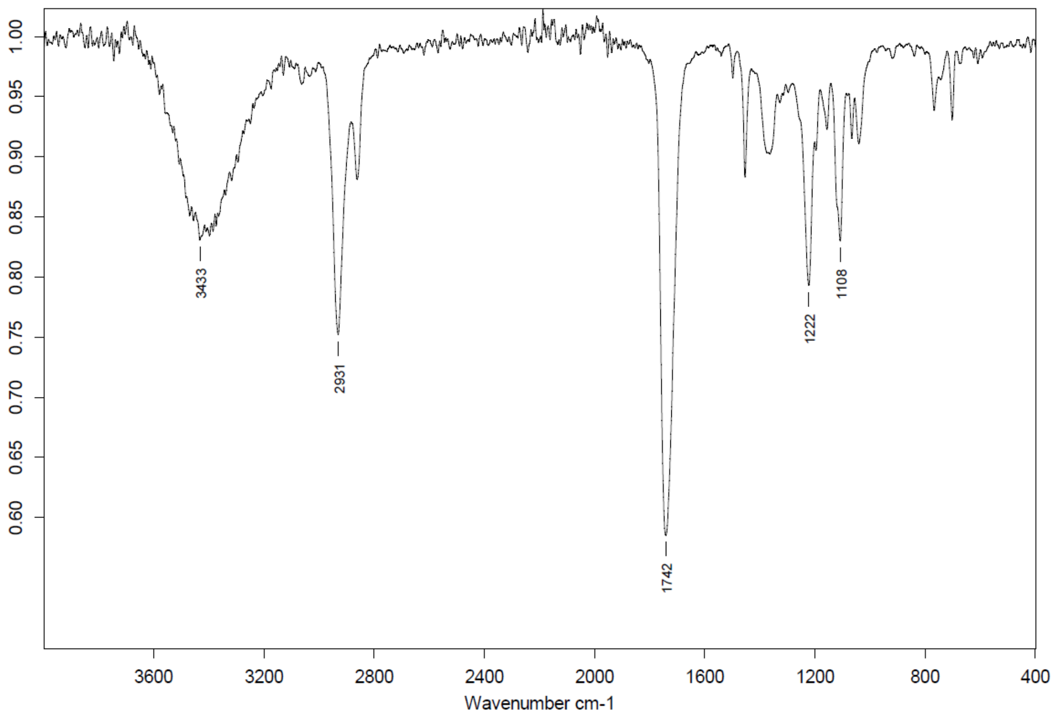
1.00
0.95
0.90
0.85
0.80
0.75
0.70
0.65
0.60
3433
2931
1742
1222
1108
3600
3200
2800
2400
2000
1600
1200
800
400
Wavenumber cm-1

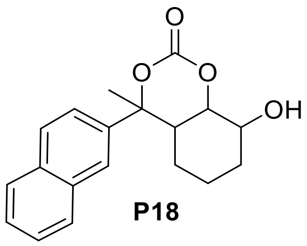

P18

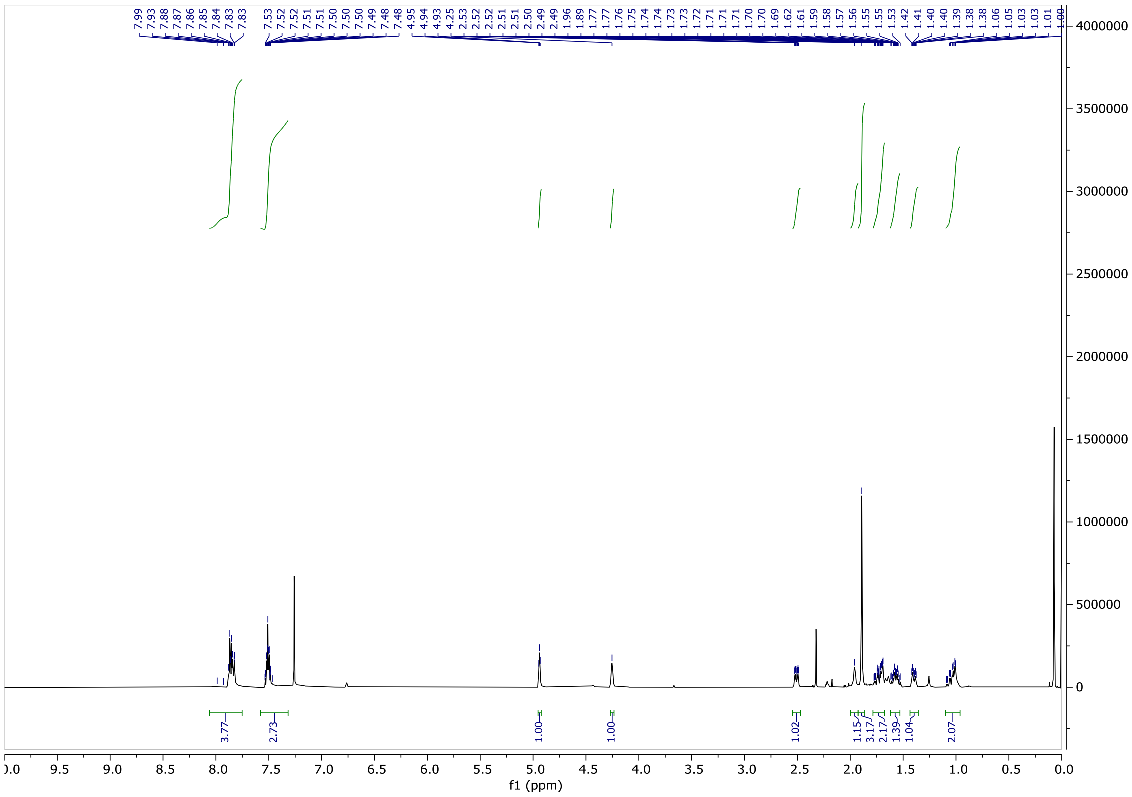

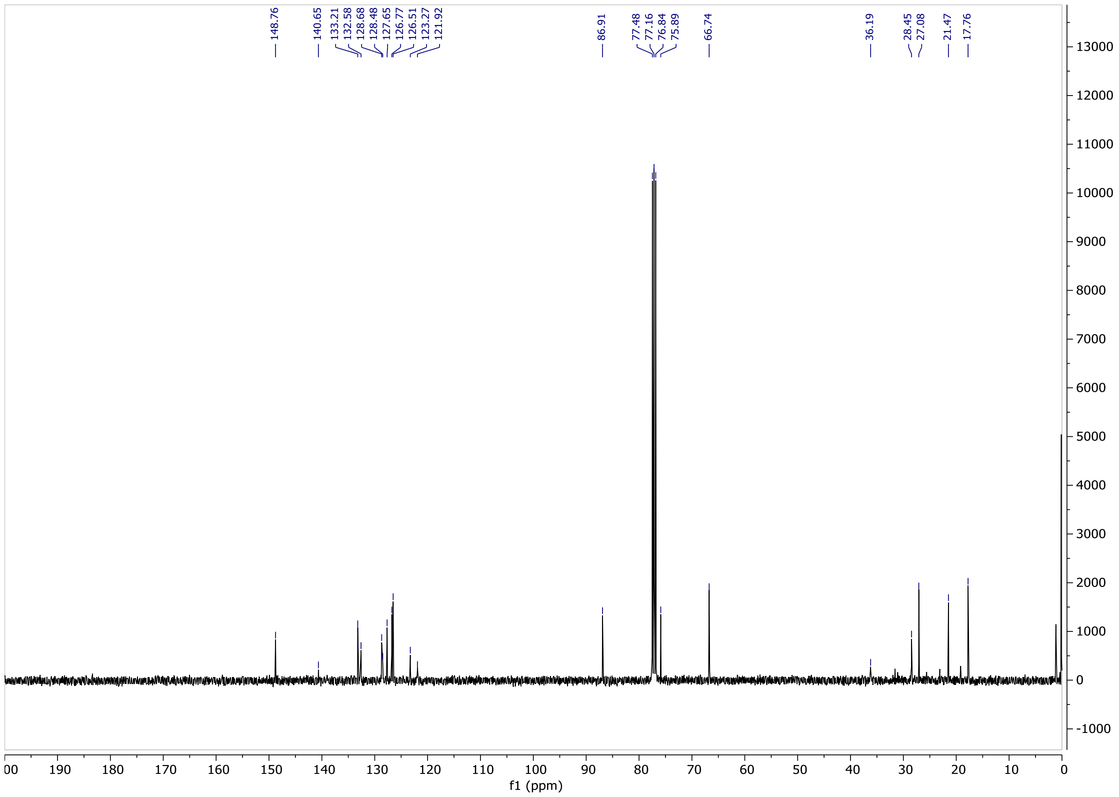
148.76
140.65
133.21
132.58
128.68
128.48
127.65
126.77
126.51
123.27
121.92
86.91
77.48
77.16
76.84
75.89
66.74
36.19
28.45
27.08
21.47
17.76
f1 (ppm)

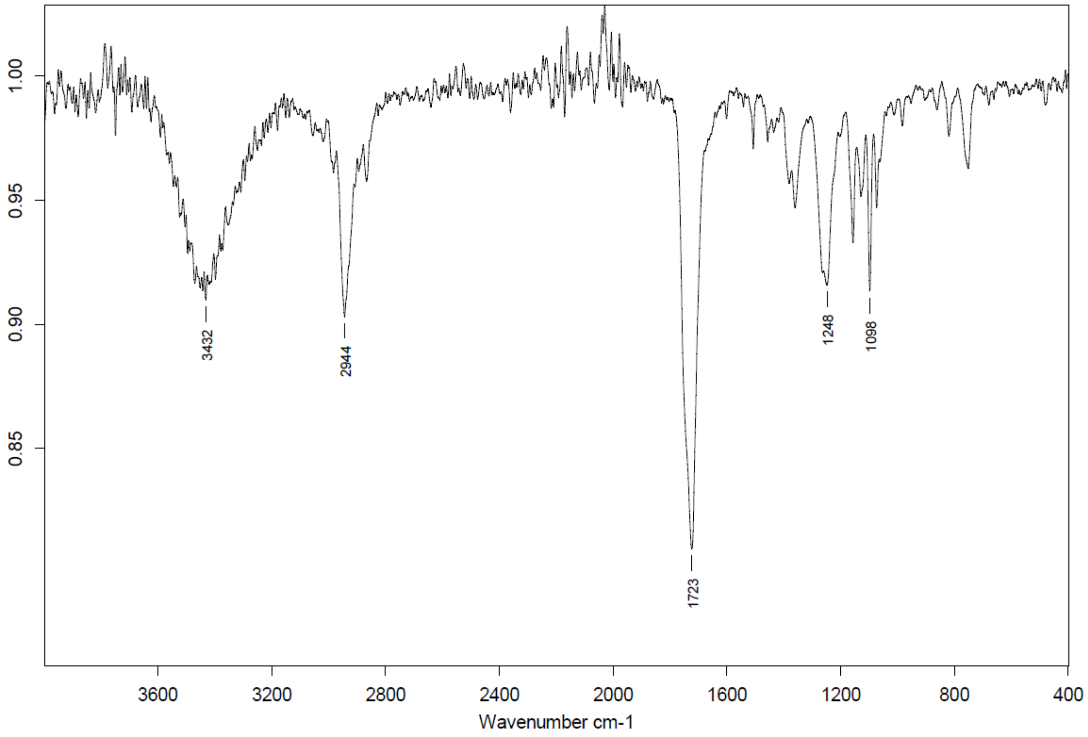
3432
2944
1723
1248
1098
Wavenumber cm-1

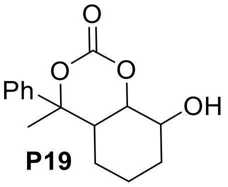
O
O
O
Ph
OH

P19

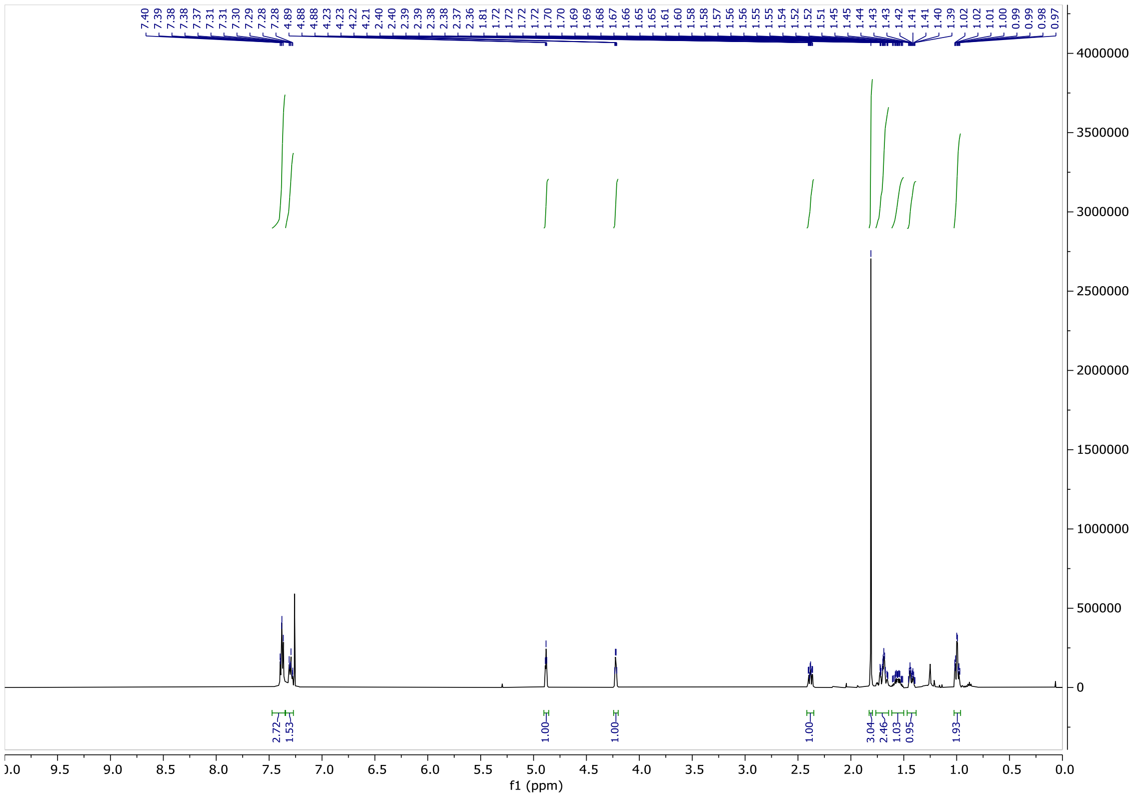
7.40 7.39 7.38 7.38 7.37 7.31 7.31 7.30 7.29 7.28 7.28 4.89 4.88 4.88 4.23 4.23 4.22 4.21 2.40 2.40 2.39 2.39 2.38 2.38 2.37 2.36 1.81 1.72 1.72 1.72 1.72 1.70 1.69 1.69 1.68 1.67 1.66 1.65 1.65 1.61 1.60 1.58 1.58 1.57 1.56 1.56 1.55 1.55 1.54 1.52 1.52 1.51 1.45 1.45 1.44 1.43 1.43 1.42 1.41 1.40 1.39 1.02 1.02 1.01 1.00 0.99 0.99 0.98 0.97
2.72 1.53 1.00 1.00 1.00 3.04 2.46 1.03 0.95 1.93
f1 (ppm)

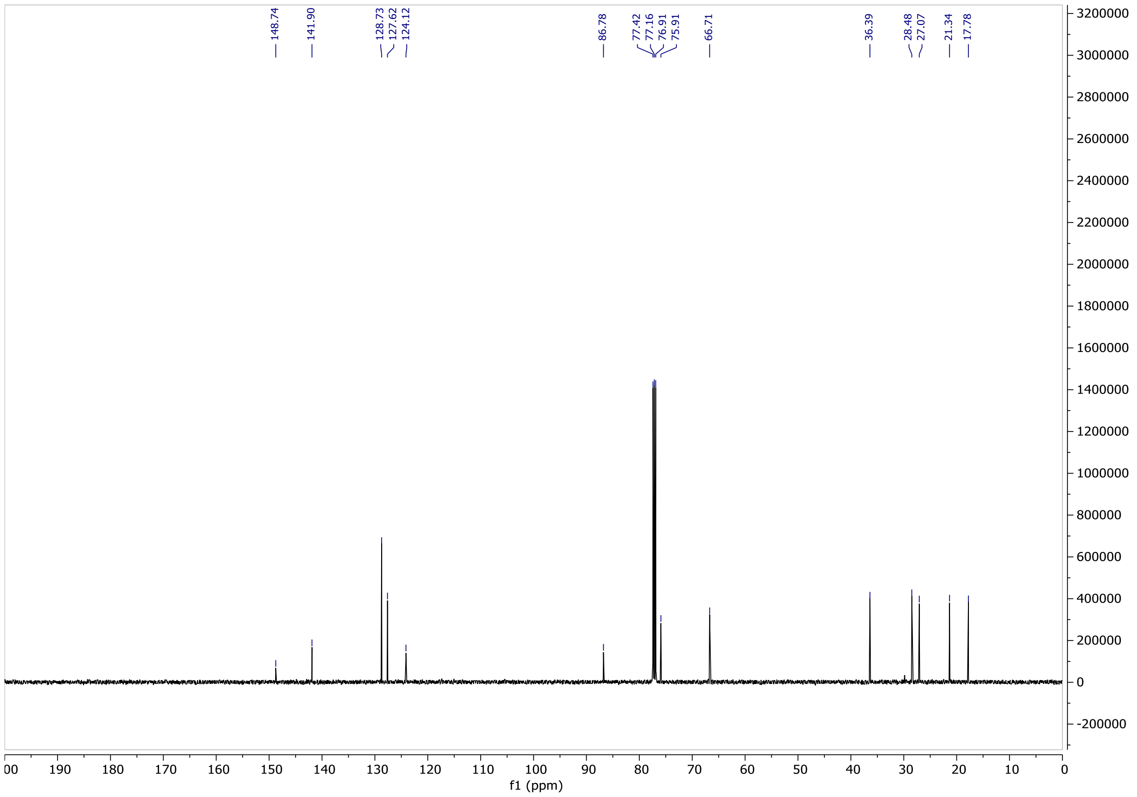
148.74 141.90 128.73 127.62 124.12 86.78 77.42 77.16 76.91 75.91 66.71 36.39 28.48 27.07 21.34 17.78
f1 (ppm)

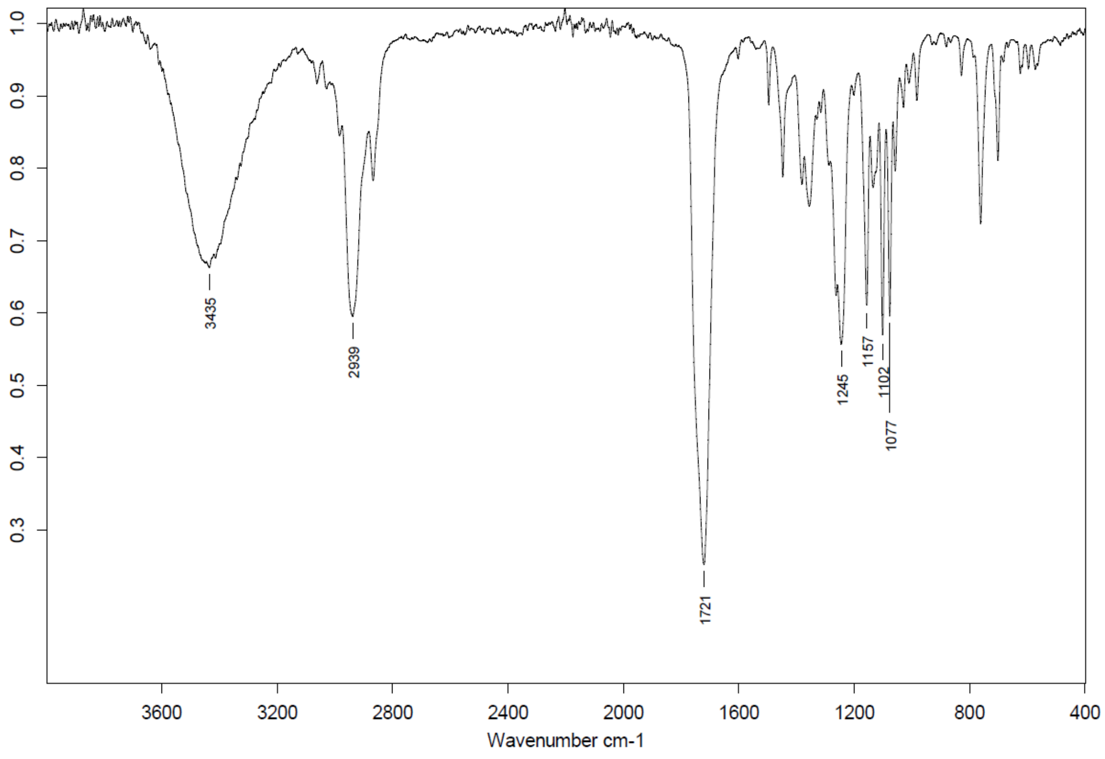
1.0
0.9
0.8
0.7
0.6
0.5
0.4
0.3
3435
2939
1721
1245
1157
1102
1077
3600
3200
2800
2400
2000
1600
1200
800
400
Wavenumber cm-1

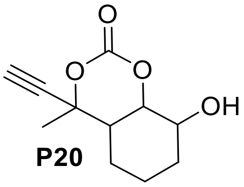

P20

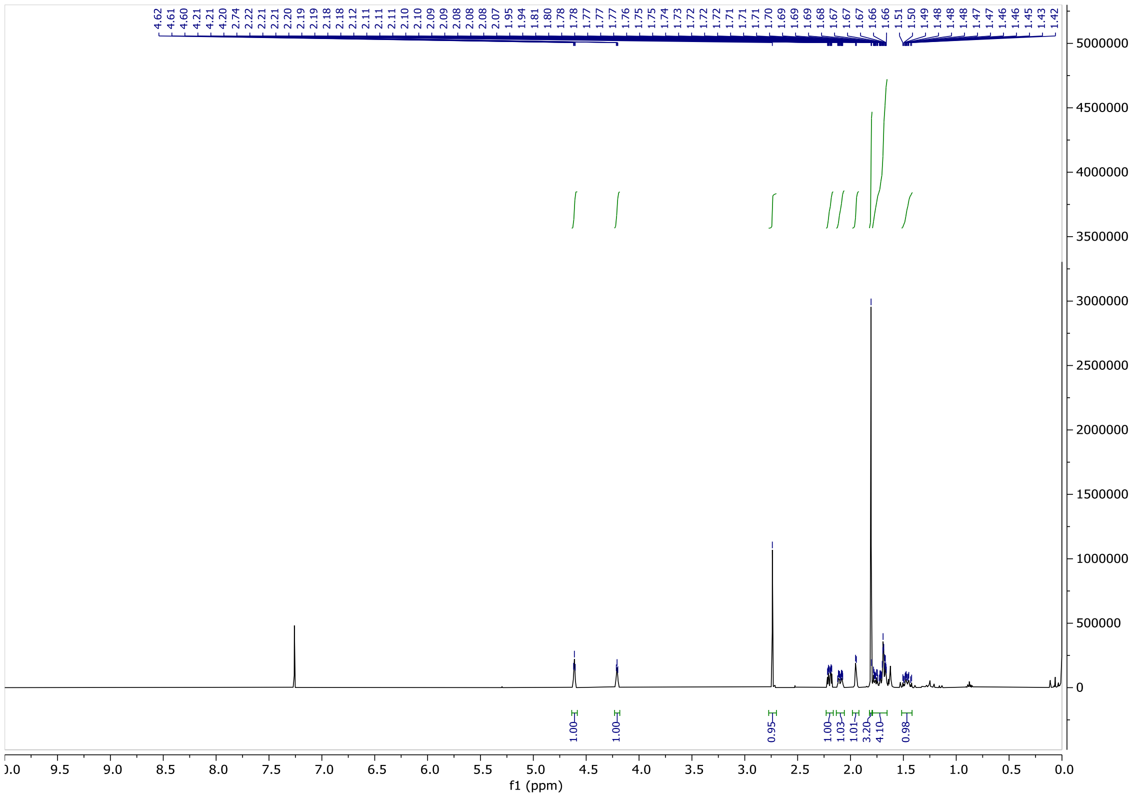

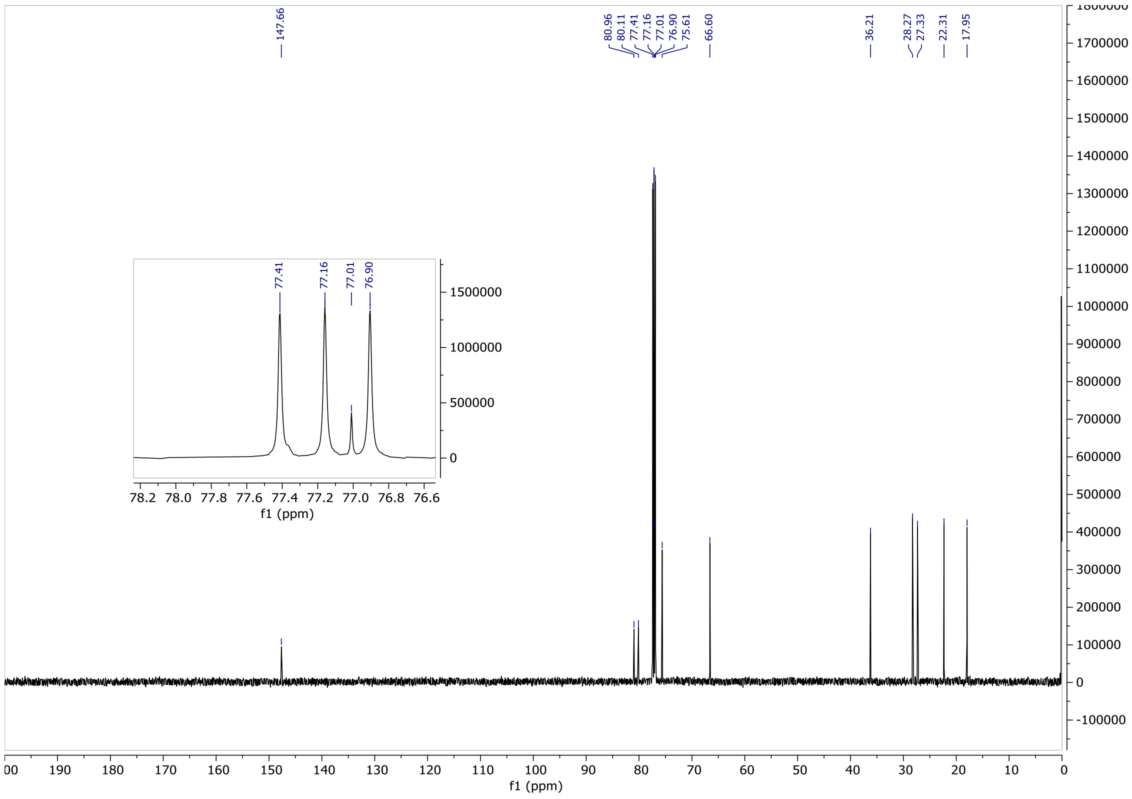

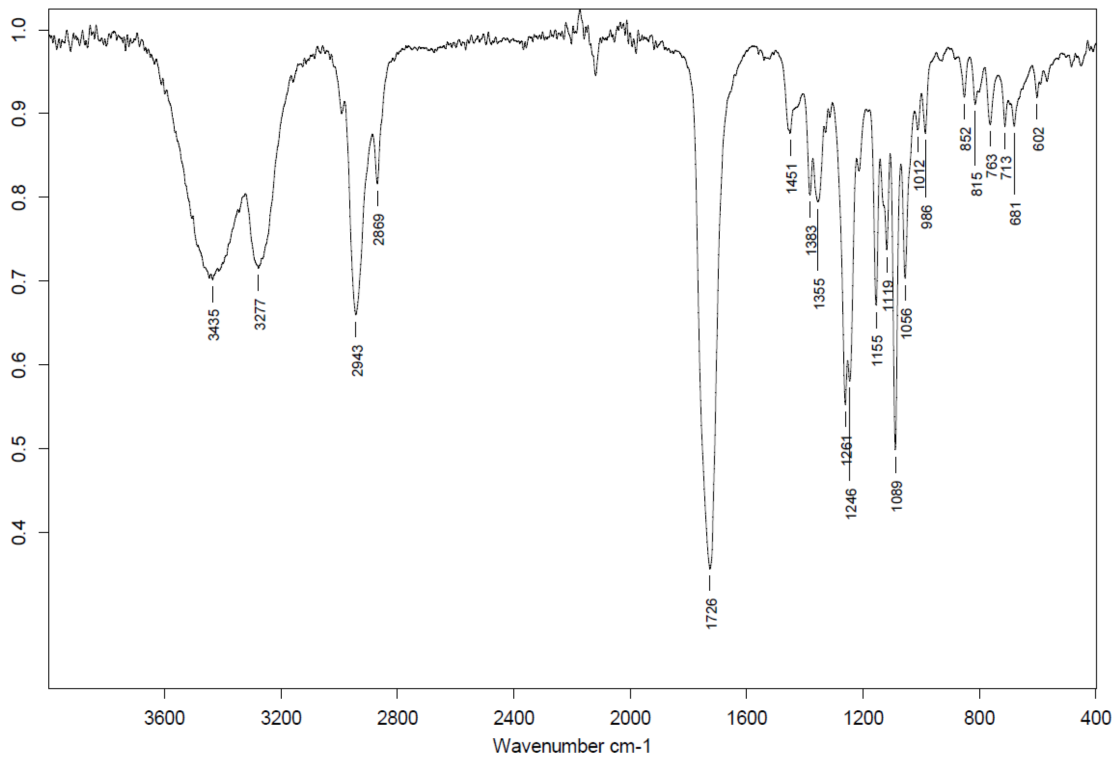
1.0
0.9
0.8
0.7
0.6
0.5
0.4
3435
3277
2943
2869
1726
1451
1383
1355
1261
1246
1155
1119
1089
1056
1012
986
852
815
763
713
681
602
3600
3200
2800
2400
2000
1600
1200
800
400
Wavenumber cm-1

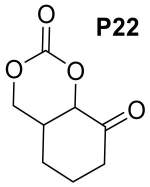
O
O
O
O

P22

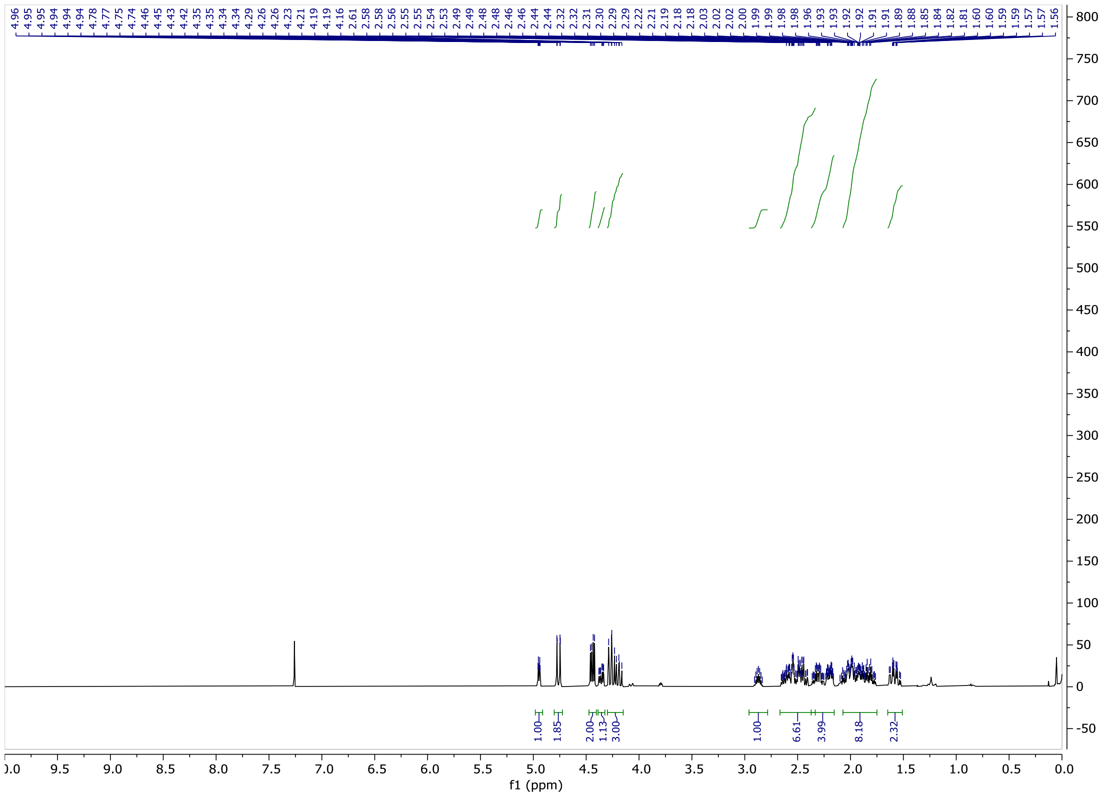
4.96 4.95 4.95 4.94 4.94 4.94 4.78 4.77 4.75 4.74 4.46 4.45 4.43 4.42 4.35 4.35 4.34 4.34 4.29 4.26 4.26 4.23 4.21 4.19 4.19 4.16 2.61 2.58 2.56 2.55 2.54 2.53 2.49 2.49 2.48 2.48 2.46 2.46 2.44 2.44 2.32 2.32 2.31 2.30 2.29 2.29 2.22 2.21 2.19 2.18 2.18 2.03 2.02 2.02 2.00 1.99 1.99 1.98 1.98 1.96 1.93 1.93 1.92 1.92 1.91 1.91 1.89 1.88 1.85 1.84 1.82 1.81 1.60 1.60 1.59 1.59 1.57 1.56
1.00
1.85
2.00
1.13
3.00
1.00
6.61
3.99
8.18
2.32
f1 (ppm)

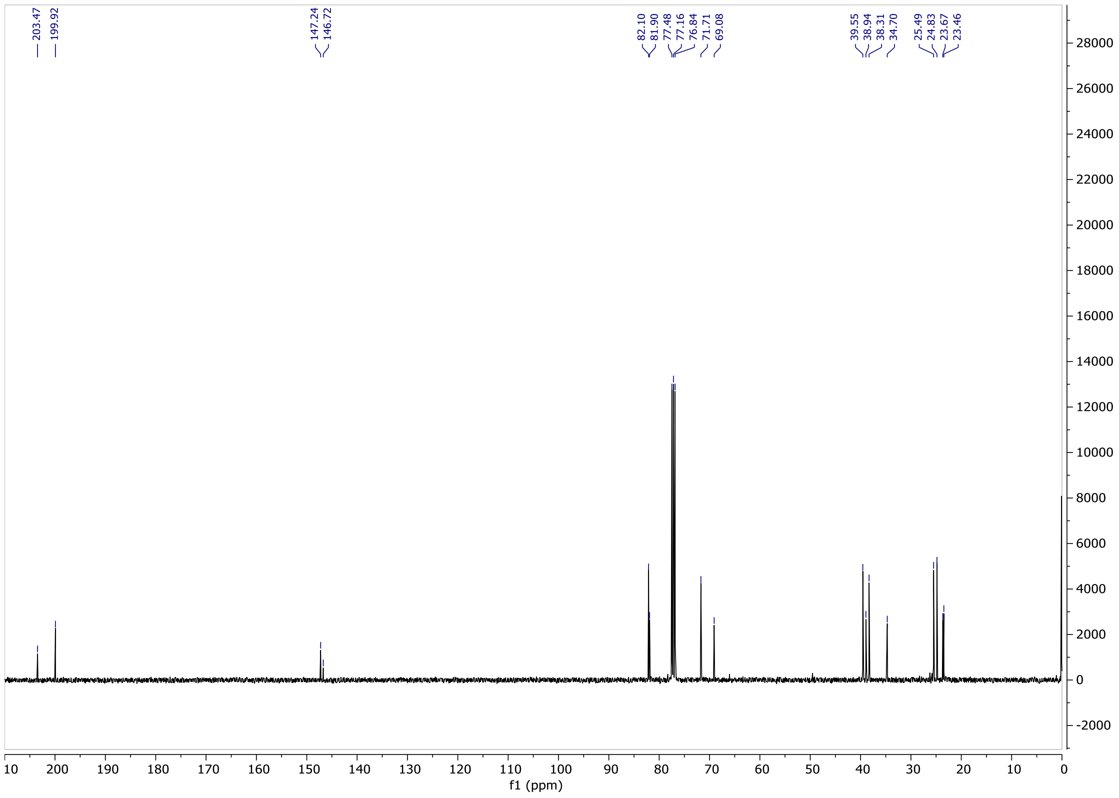
203.47
199.92
147.24
146.72
82.10
81.90
77.48
77.16
76.84
71.71
69.08
39.55
38.94
38.31
34.70
25.49
24.83
23.67
23.46
f1 (ppm)

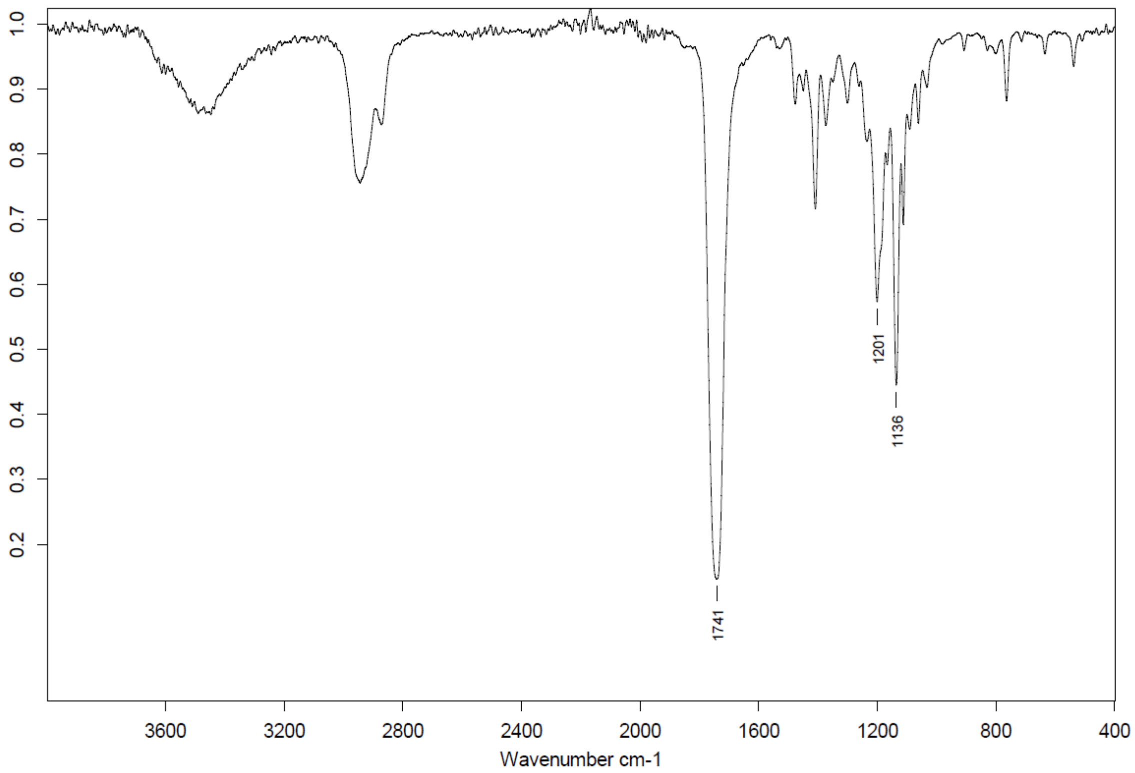
1.0
0.9
0.8
0.7
0.6
0.5
0.4
0.3
0.2
1741
1201
1136
3600
3200
2800
2400
2000
1600
1200
800
400
Wavenumber cm-1

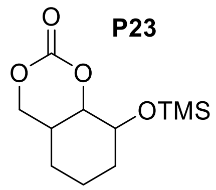
O
O
O
OTMS

P23

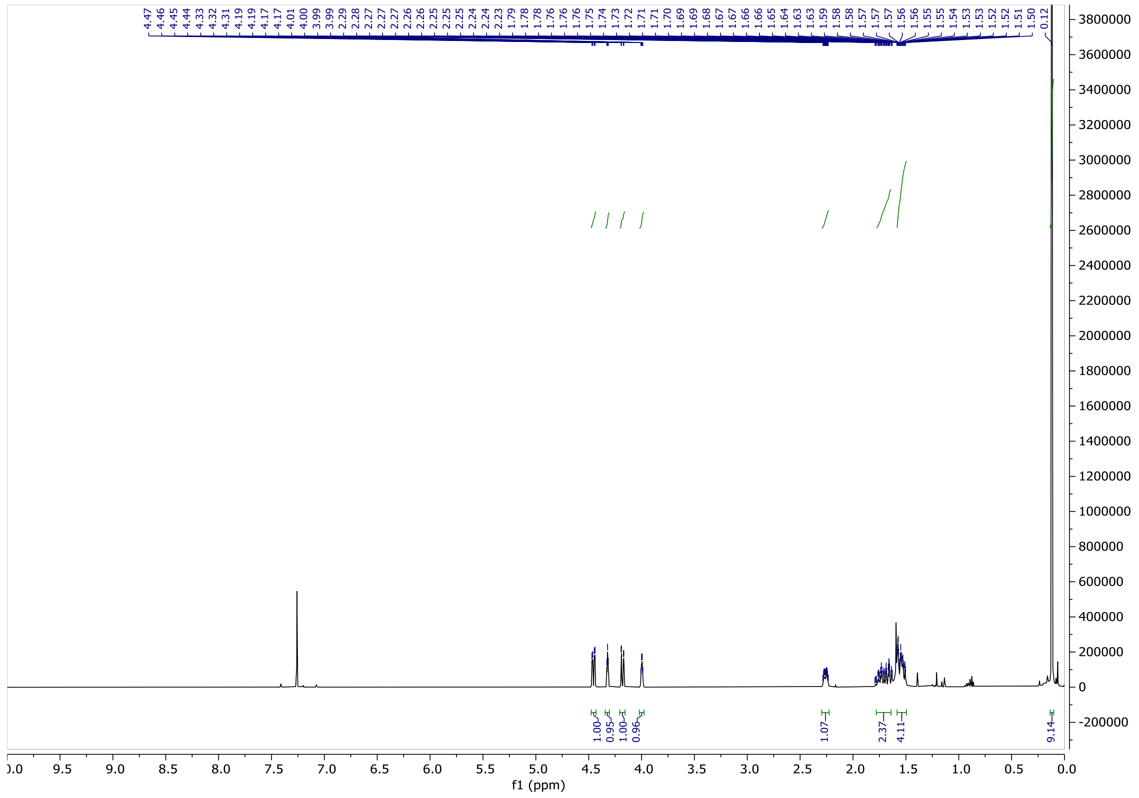
f1 (ppm)

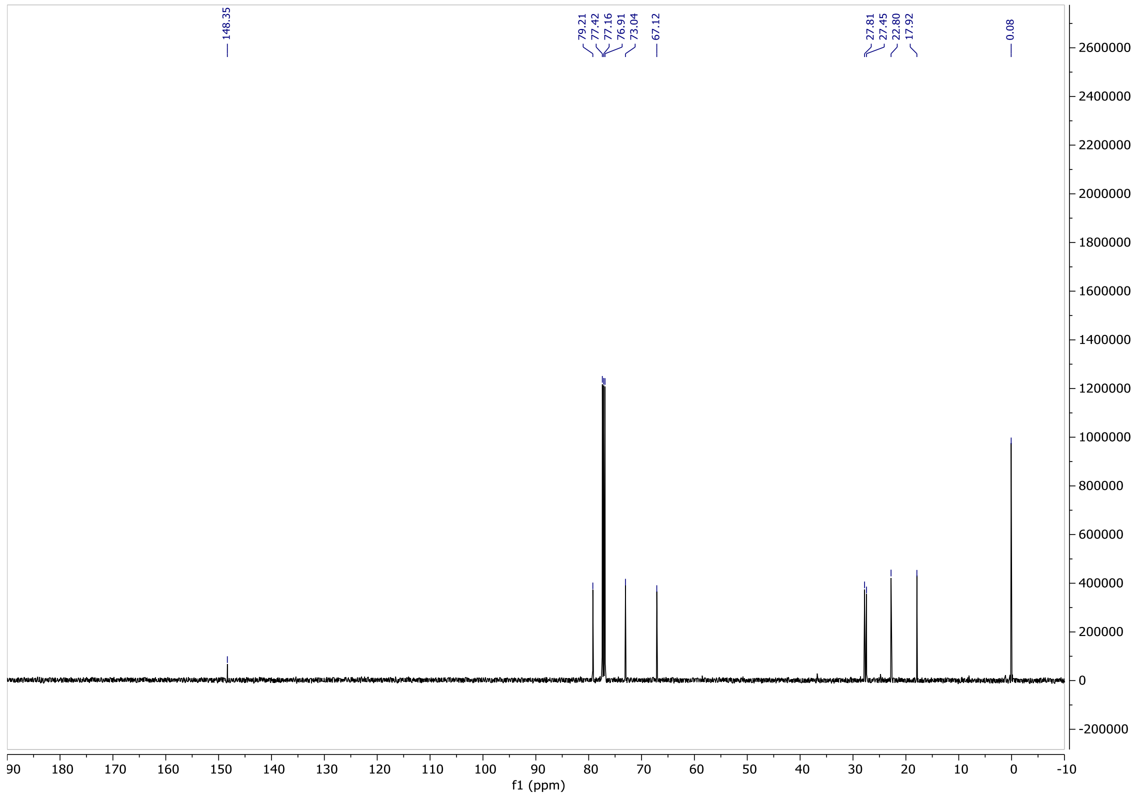
148.35
79.21
77.42
77.16
76.91
73.04
67.12
27.81
27.45
22.80
17.92
0.08
f1 (ppm)

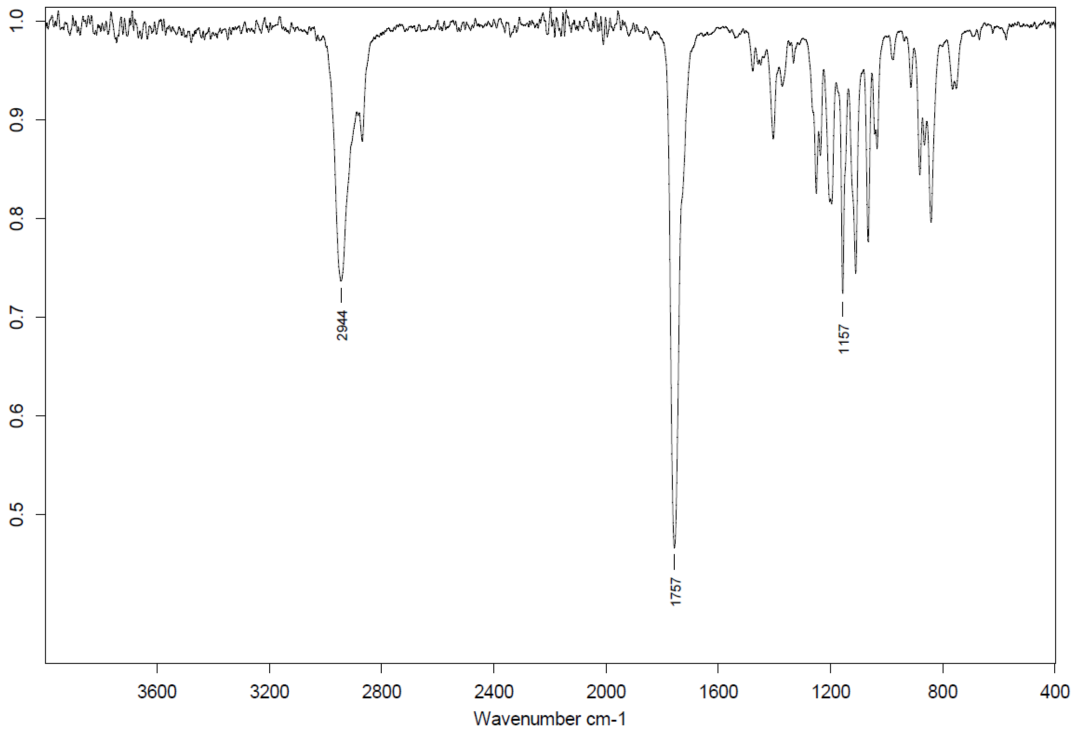

1.0
0.9
0.8
0.7
0.6
0.5
2944
1757
1157
3600
3200
2800
2400
2000
1600
1200
800
400
Wavenumber cm-1

P24

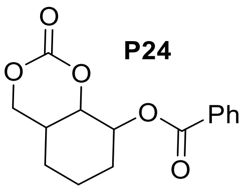

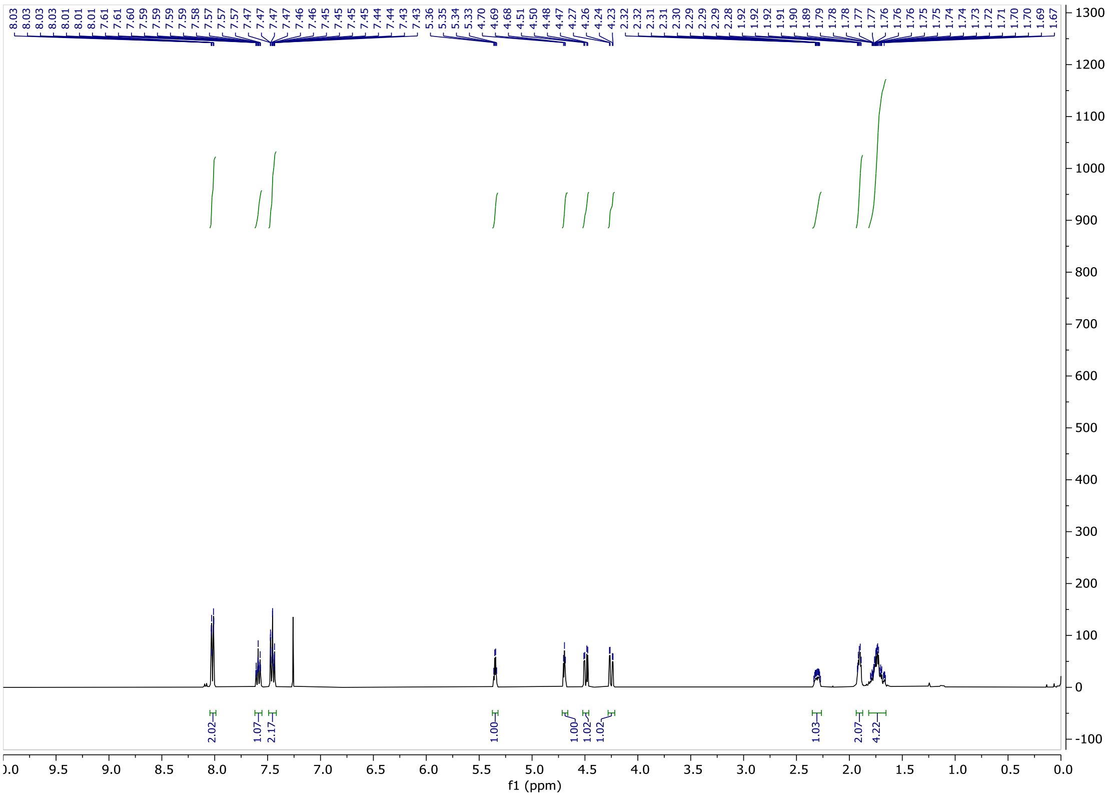
8.03 8.03 8.03 8.03 8.01 8.01 8.01 7.61 7.60 7.59 7.59 7.59 7.59 7.58 7.57 7.57 7.57 7.47 7.47 7.47 7.47 7.46 7.45 7.45 7.45 7.45 7.44 7.44 7.43 5.36 5.35 5.34 5.33 4.70 4.69 4.68 4.51 4.50 4.48 4.47 4.27 4.26 4.24 4.23 2.32 2.31 2.31 2.30 2.29 2.29 2.29 2.28 1.92 1.92 1.92 1.91 1.90 1.89 1.79 1.78 1.78 1.77 1.77 1.76 1.76 1.75 1.75 1.74 1.74 1.73 1.72 1.71 1.70 1.69 1.67
2.02
1.07
2.17
1.00
1.00
1.02
1.02
1.03
2.07
4.22
f1 (ppm)

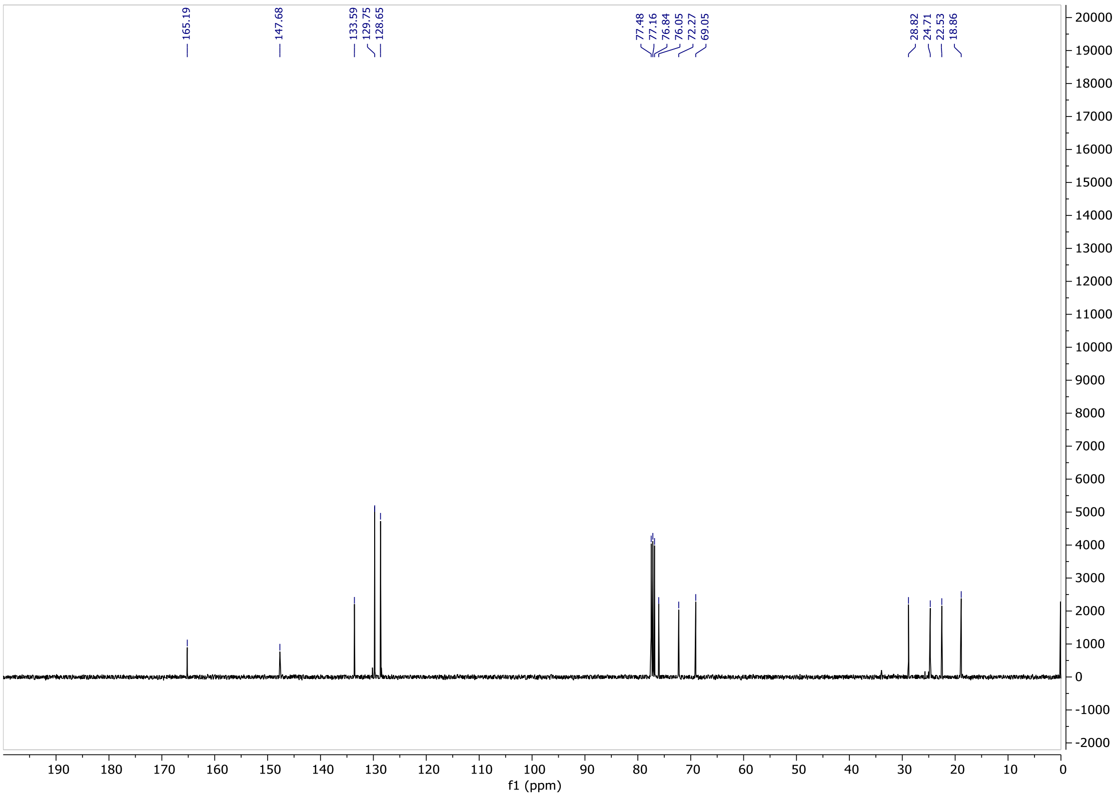
165.19
147.68
133.59
129.75
128.65
77.48
77.16
76.84
76.05
72.27
69.05
28.82
24.71
22.53
18.86
f1 (ppm)

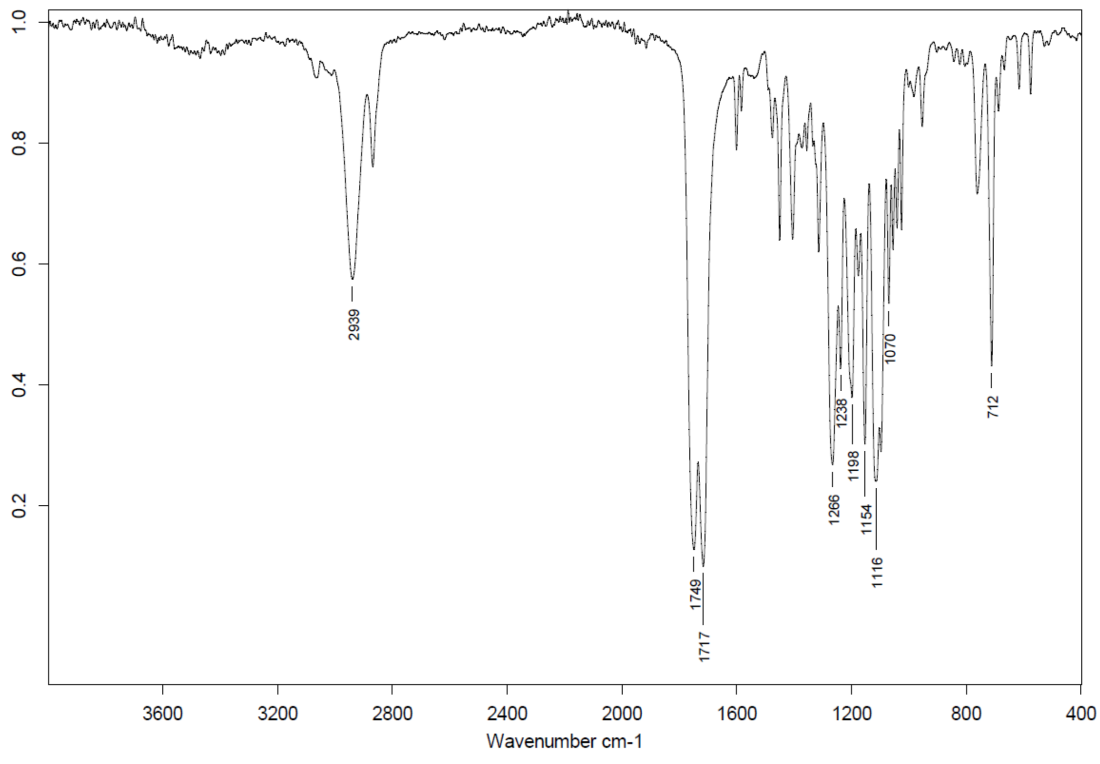

1.0
0.8
0.6
0.4
0.2
2939
1749
1717
1266
1238
1198
1154
1116
1070
712
3600
3200
2800
2400
2000
1600
1200
800
400
Wavenumber cm-1

P24

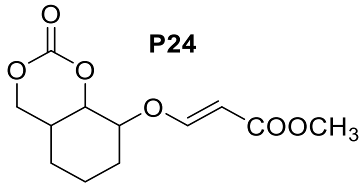

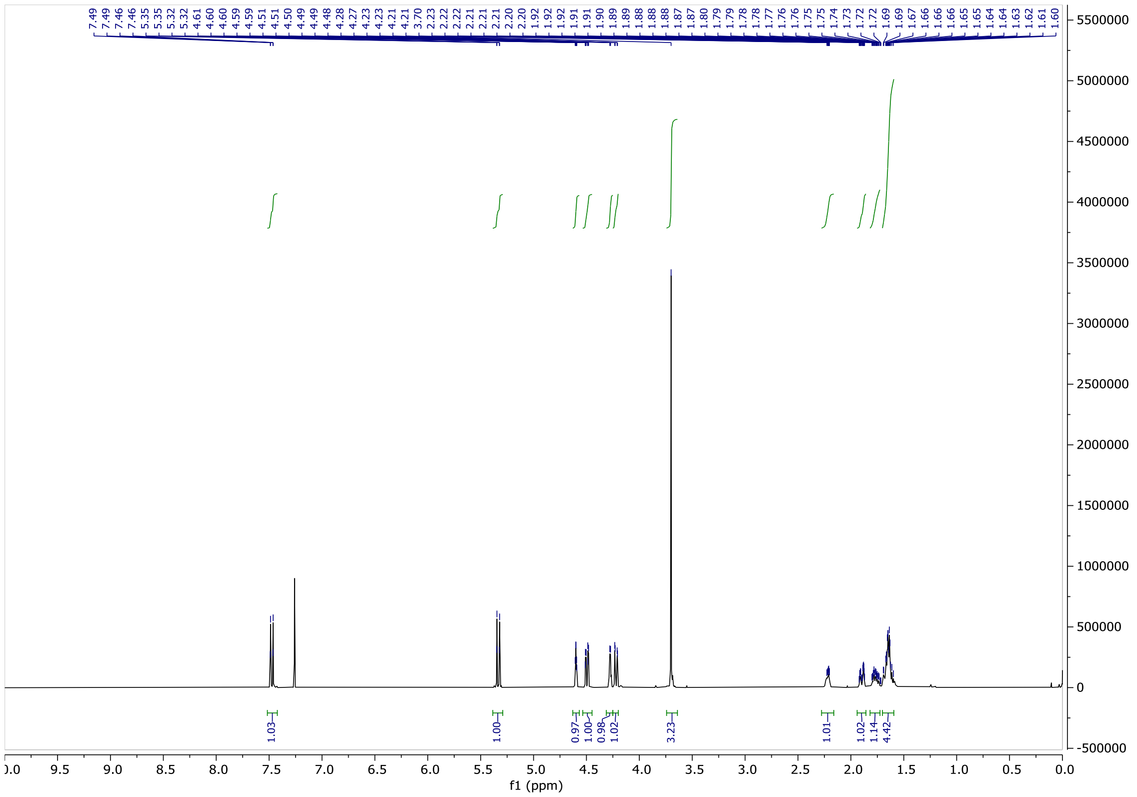

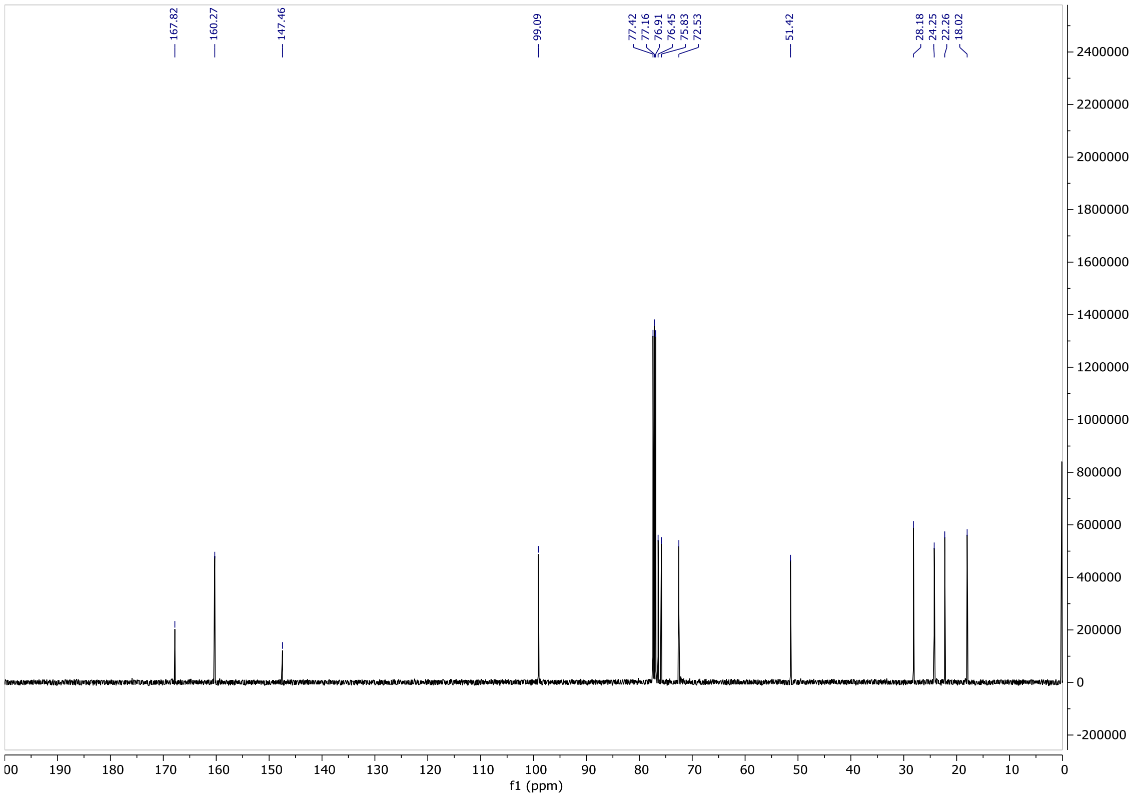

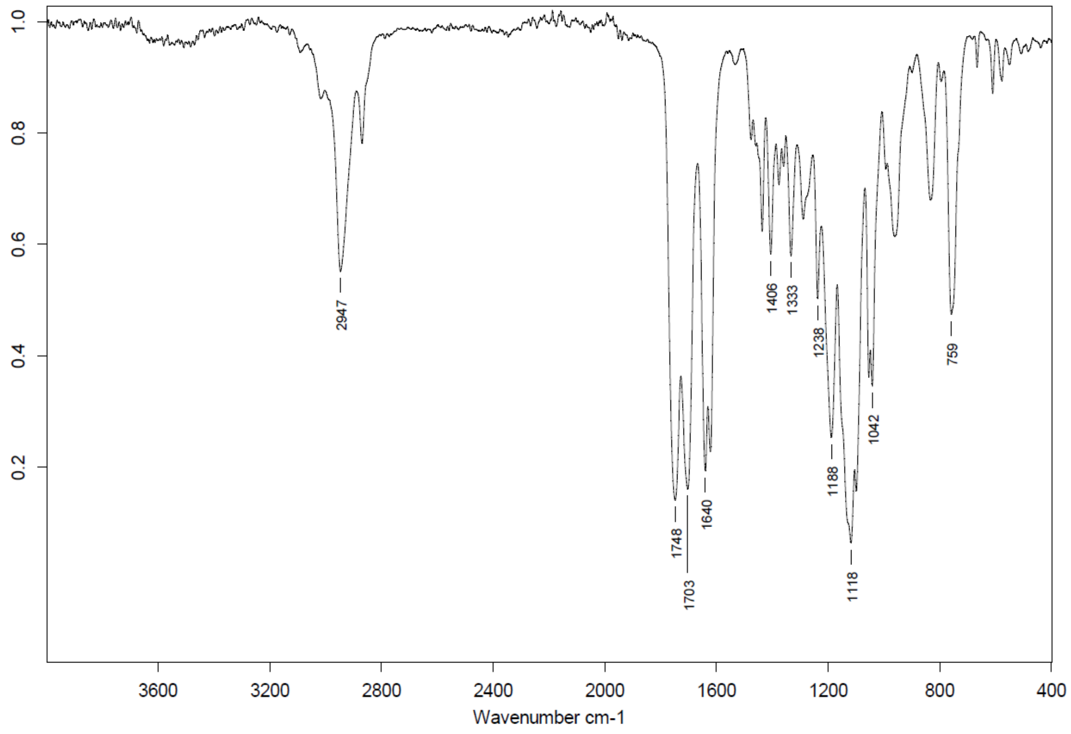

1.0
0.8
0.6
0.4
0.2
2947
1748
1703
1640
1406
1333
1238
1188
1118
1042
759
3600
3200
2800
2400
2000
1600
1200
800
400
Wavenumber cm-1

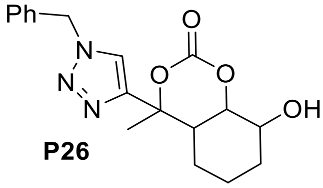
Ph
N
N
N
O
O
O
OH

P26

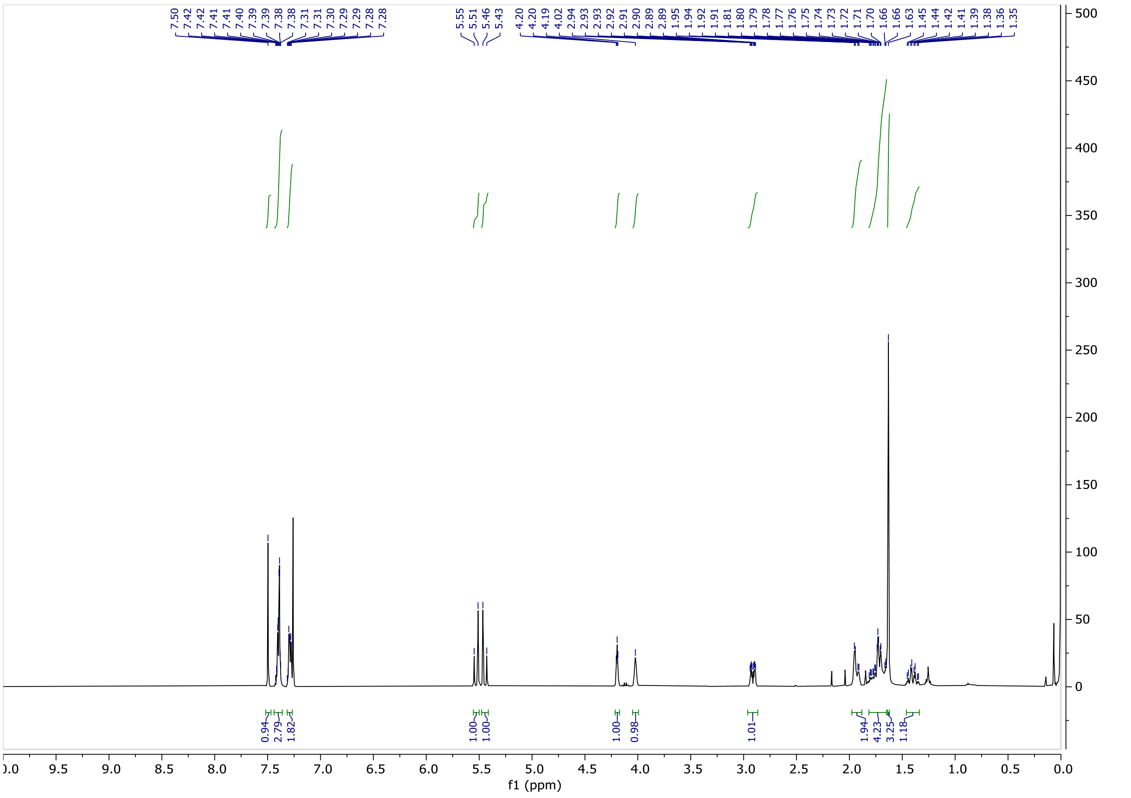
7.50
7.42
7.42
7.41
7.40
7.39
7.38
7.38
7.31
7.31
7.30
7.29
7.29
7.28
7.28
5.55
5.51
5.46
5.43
4.20
4.20
4.19
4.02
2.94
2.93
2.93
2.92
2.91
2.90
2.89
2.89
1.95
1.94
1.92
1.91
1.81
1.80
1.79
1.78
1.77
1.76
1.75
1.74
1.73
1.72
1.71
1.70
1.66
1.63
1.45
1.44
1.42
1.41
1.39
1.38
1.36
1.35
0.94
2.79
1.82
1.00
1.00
1.00
0.98
1.01
1.94
4.23
3.25
1.18
f1 (ppm)

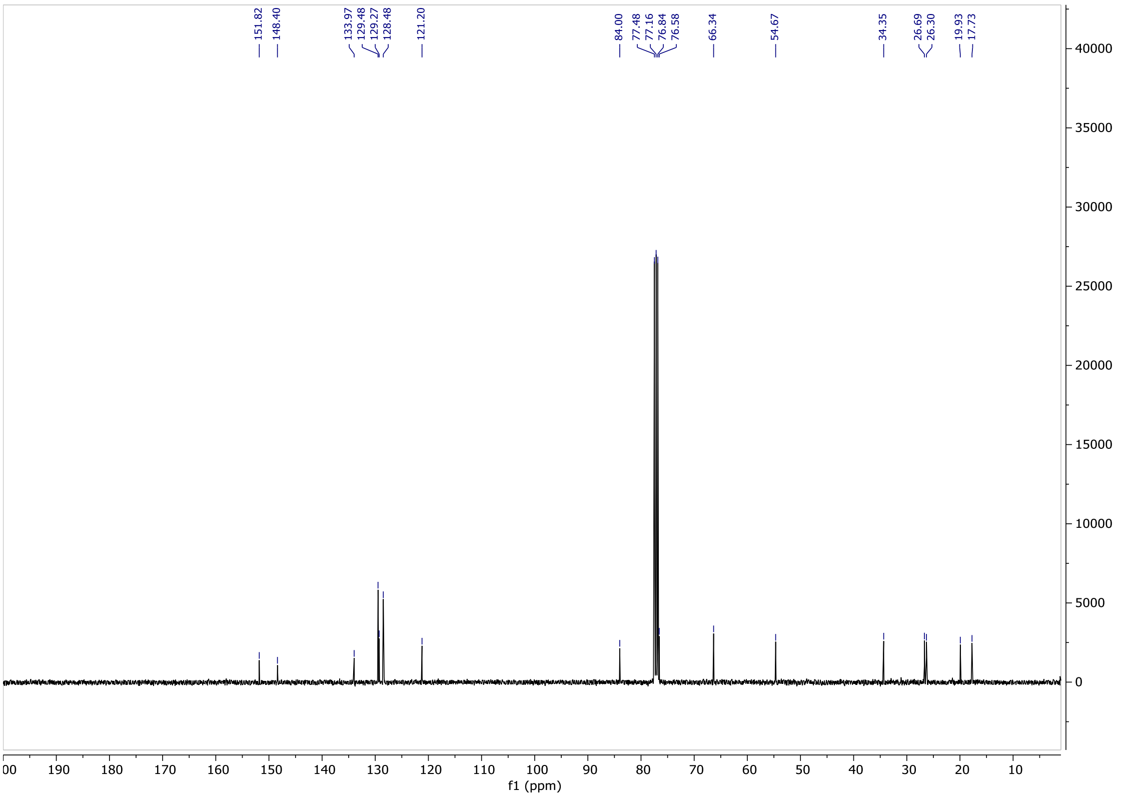
151.82
148.40
133.97
129.48
129.27
128.48
121.20
84.00
77.48
77.16
76.84
76.58
66.34
54.67
34.35
26.69
26.30
19.93
17.73
f1 (ppm)

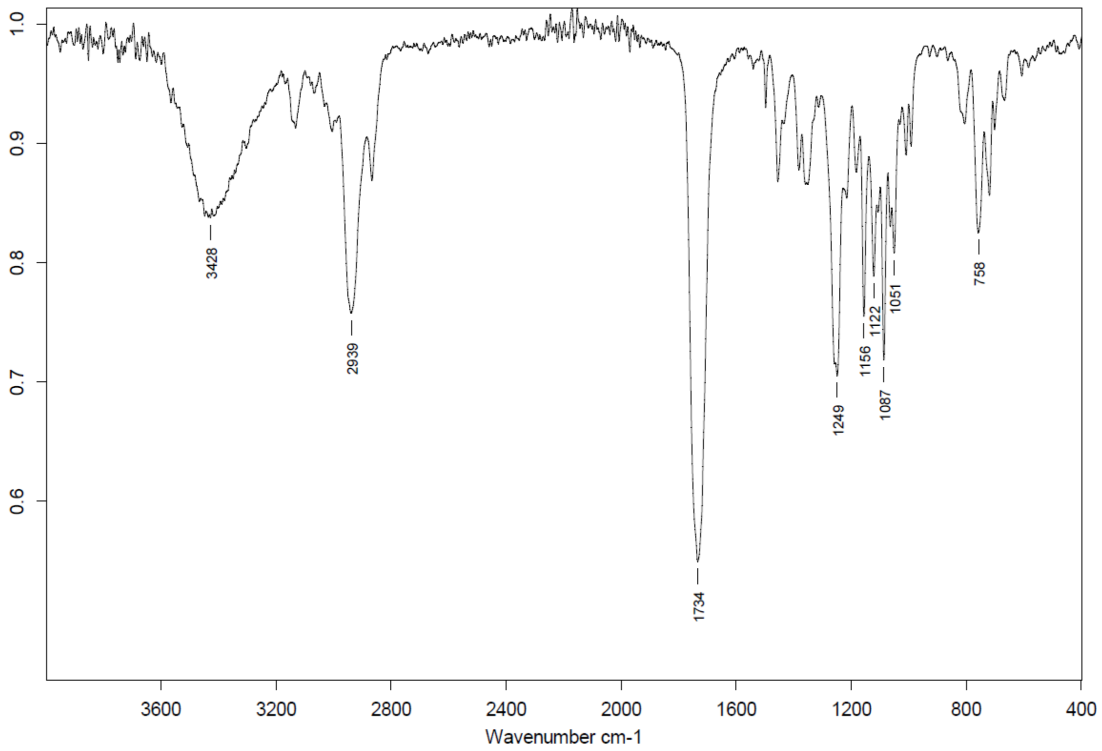
1.0
0.9
0.8
0.7
0.6
3428
2939
1734
1249
1156
1122
1087
1051
758
3600
3200
2800
2400
2000
1600
1200
800
400
Wavenumber cm-1

Intramolecular isomerization product

P21

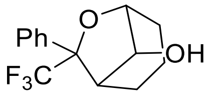

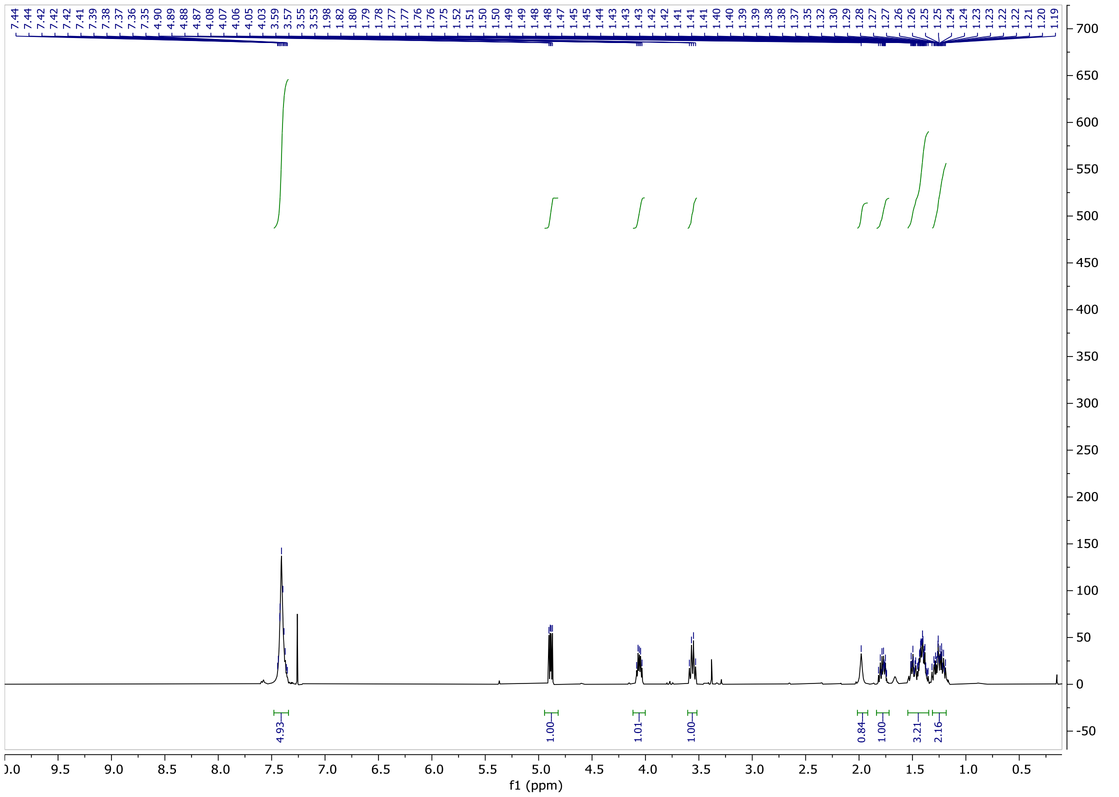

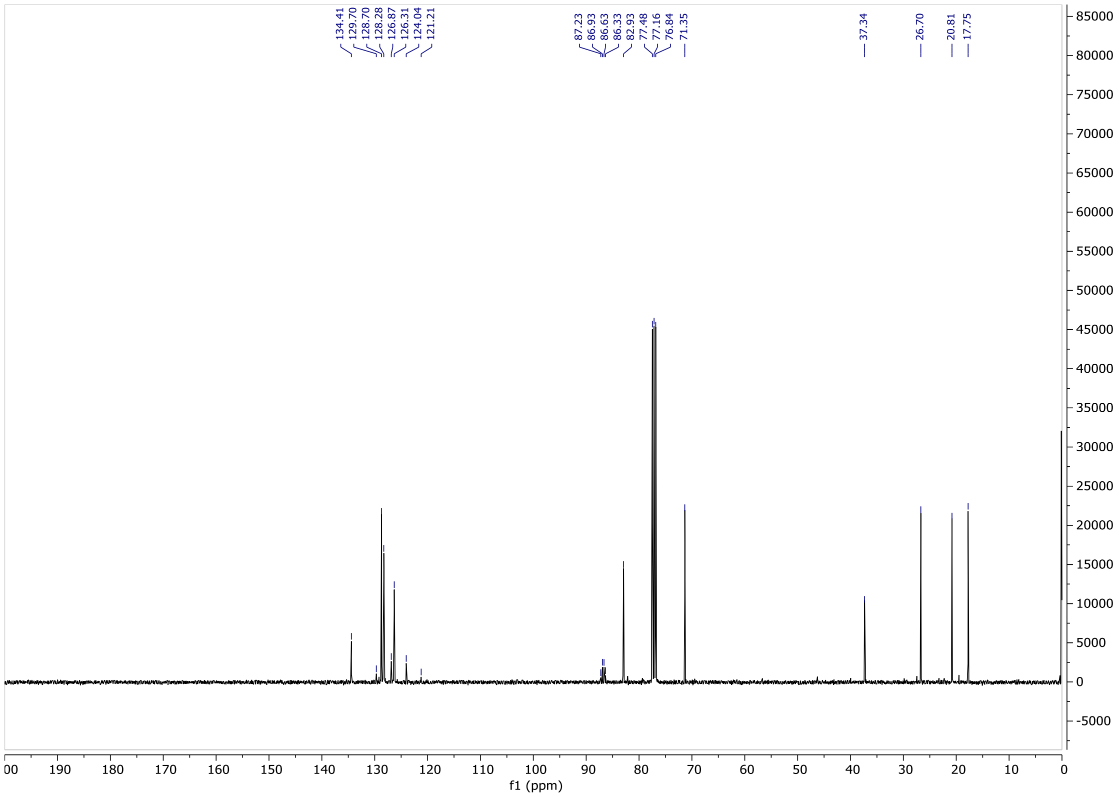

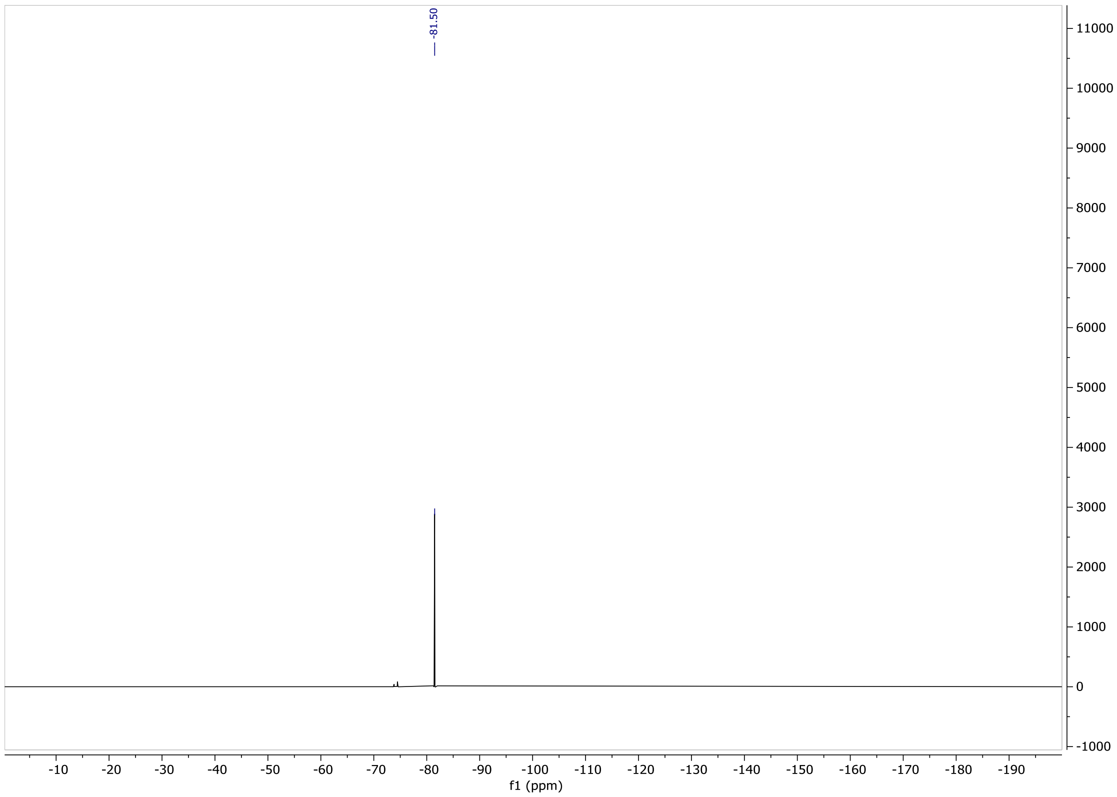

-81.50
11000
10000
9000
8000
7000
6000
5000
4000
3000
2000
1000
0
-1000
-10
-20
-30
-40
-50
-60
-70
-80
-90
-100
-110
-120
-130
-140
-150
-160
-170
-180
-190
f1 (ppm)

P15[b]

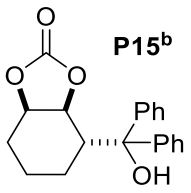
O
O
O
Ph
Ph
OH

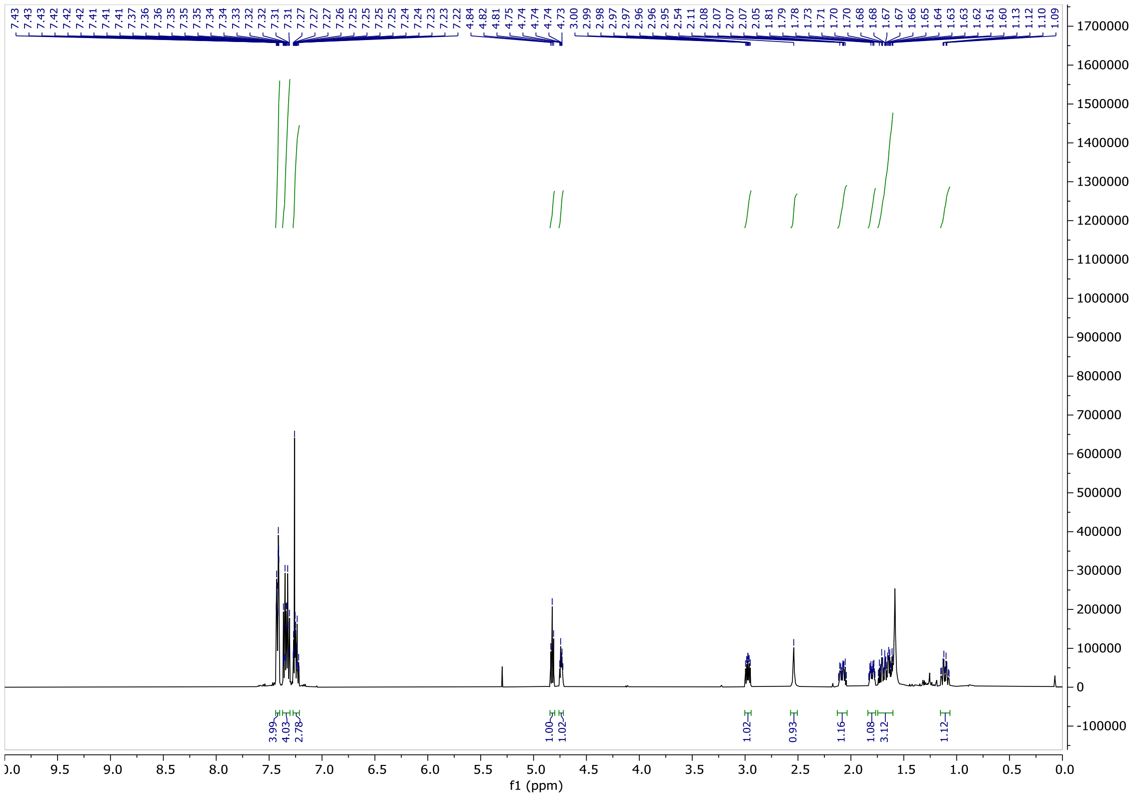
7.43
7.43
7.42
7.42
7.42
7.41
7.41
7.41
7.37
7.36
7.36
7.35
7.35
7.35
7.34
7.34
7.33
7.32
7.32
7.31
7.31
7.27
7.27
7.27
7.26
7.25
7.25
7.25
7.25
7.24
7.24
7.23
7.23
7.22
4.84
4.82
4.81
4.75
4.74
4.74
4.74
4.73
3.00
2.99
2.98
2.97
2.97
2.96
2.96
2.95
2.54
2.11
2.08
2.07
2.07
2.07
2.05
1.81
1.79
1.78
1.73
1.71
1.70
1.70
1.68
1.68
1.67
1.67
1.66
1.65
1.64
1.63
1.63
1.62
1.61
1.60
1.13
1.12
1.10
1.09
3.99
4.03
2.78
1.00
1.02
1.02
0.93
1.16
1.08
3.12
1.12
f1 (ppm)

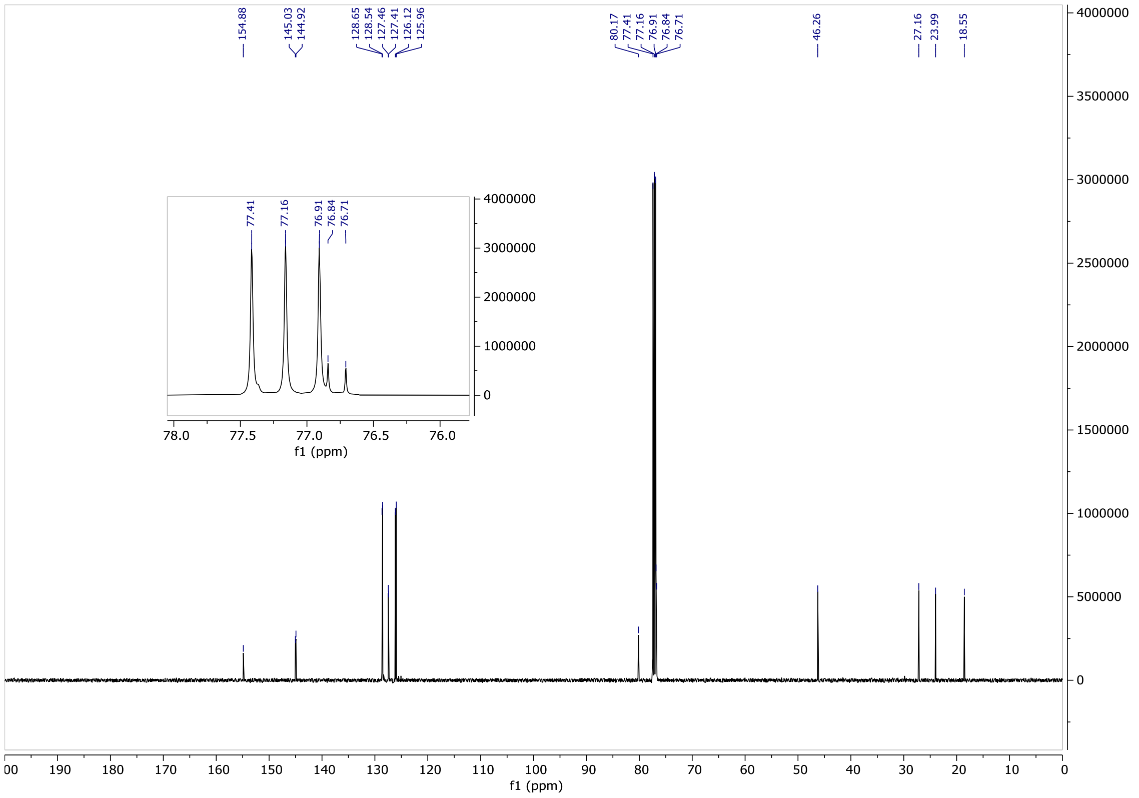
154.88
145.03
144.92
128.65
128.54
127.46
127.41
126.12
125.96
80.17
77.41
77.16
76.91
76.84
76.71
46.26
27.16
23.99
18.55
f1 (ppm)

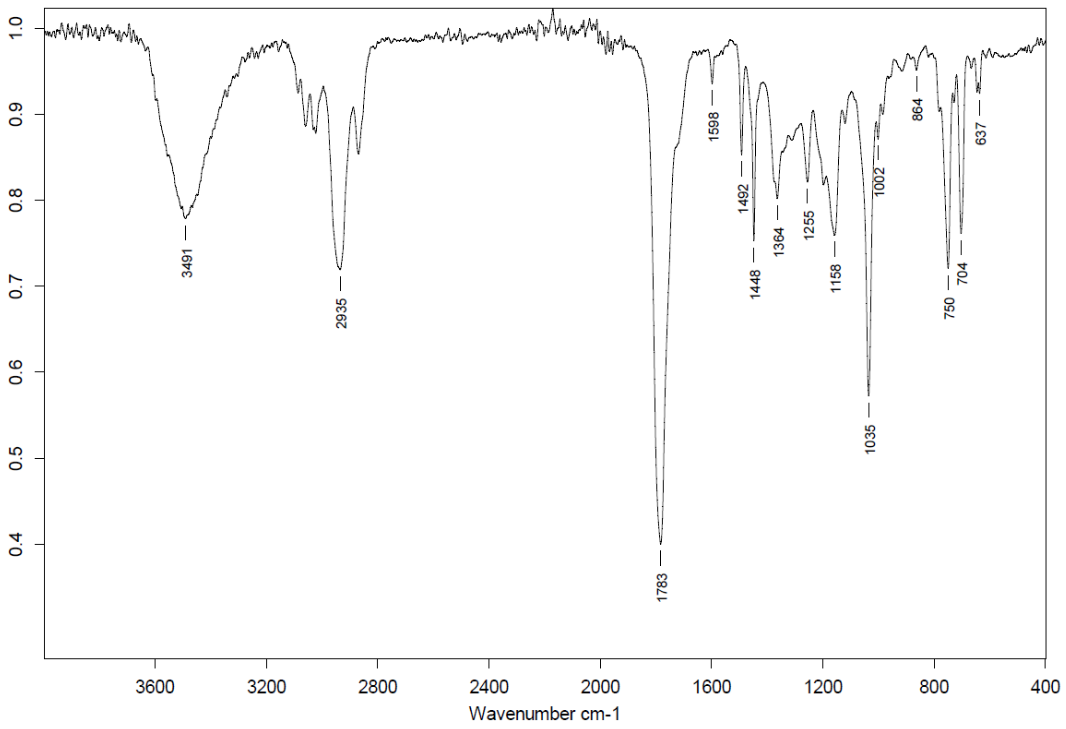
1.0
0.9
0.8
0.7
0.6
0.5
0.4
3491
2935
1783
1598
1492
1448
1364
1255
1158
1035
1002
864
750
704
637
3600
3200
2800
2400
2000
1600
1200
800
400
Wavenumber cm-1

$^1H/^{13}C$ NMR and IR spectra for products of *syn* and *anti* 5-membered carbonates

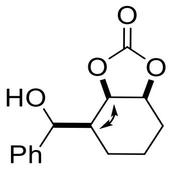

P1[a] *syn*

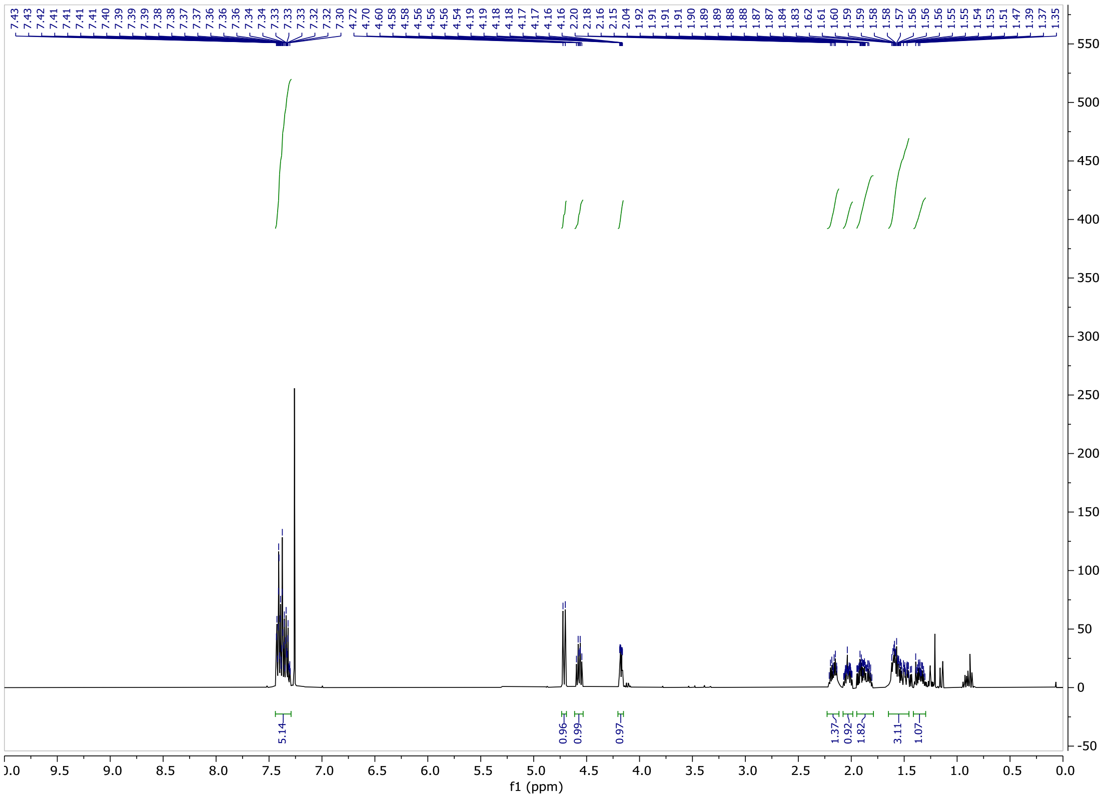

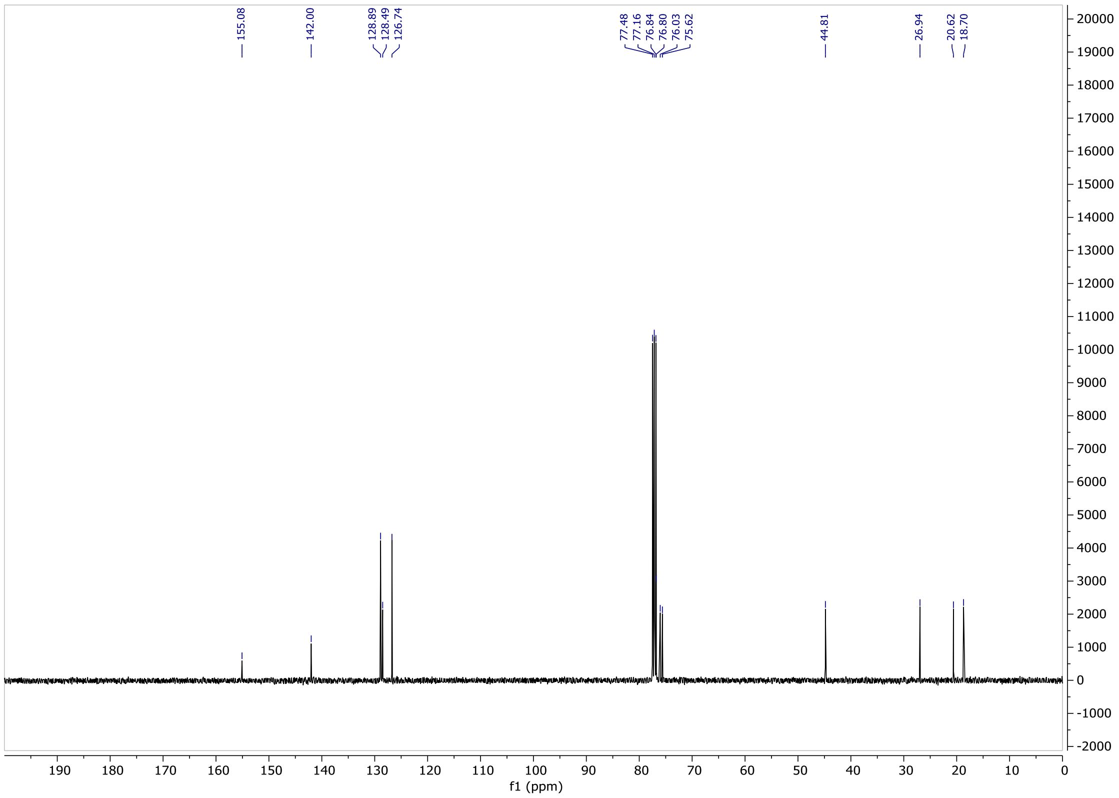
155.08
142.00
128.89
128.49
126.74
77.48
77.16
76.84
76.80
76.03
75.62
44.81
26.94
20.62
18.70
f1 (ppm)

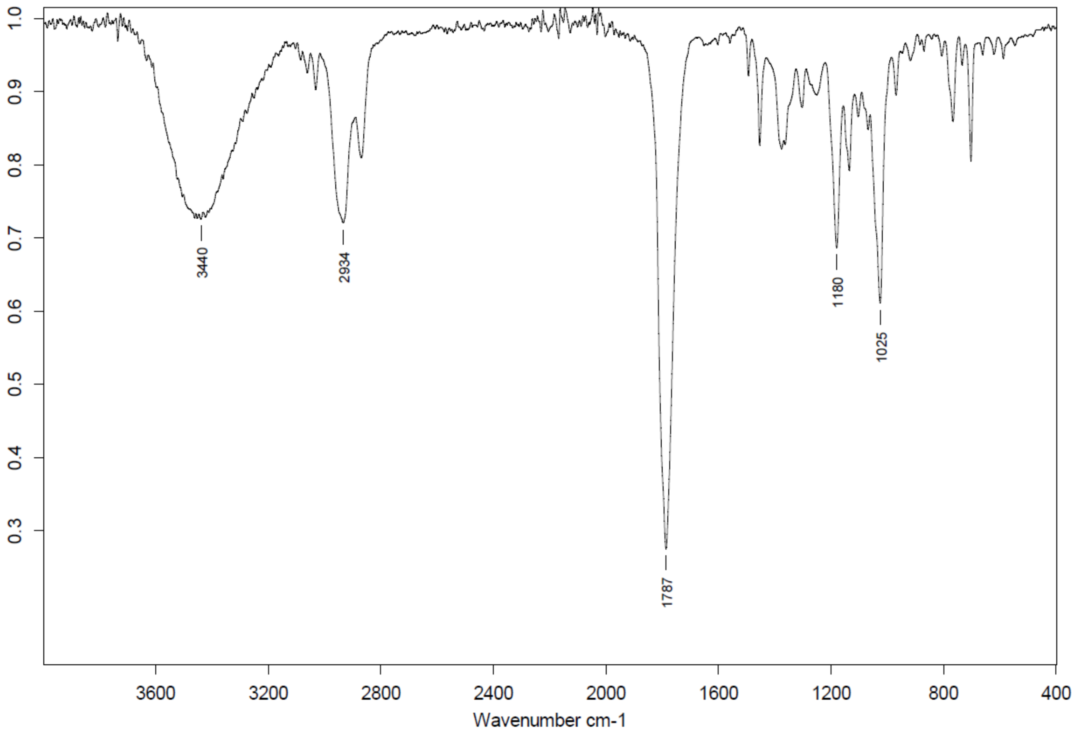
3440
2934
1787
1180
1025
Wavenumber cm-1

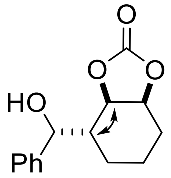

P1[b] *anti*

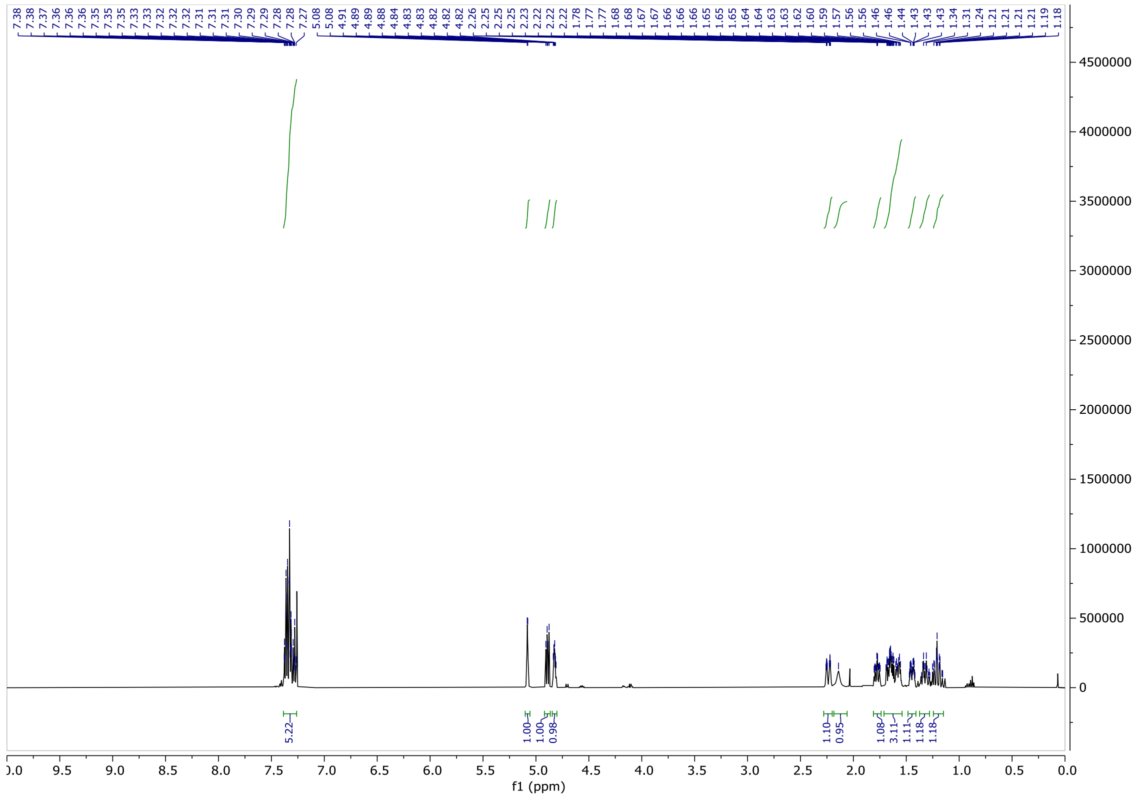

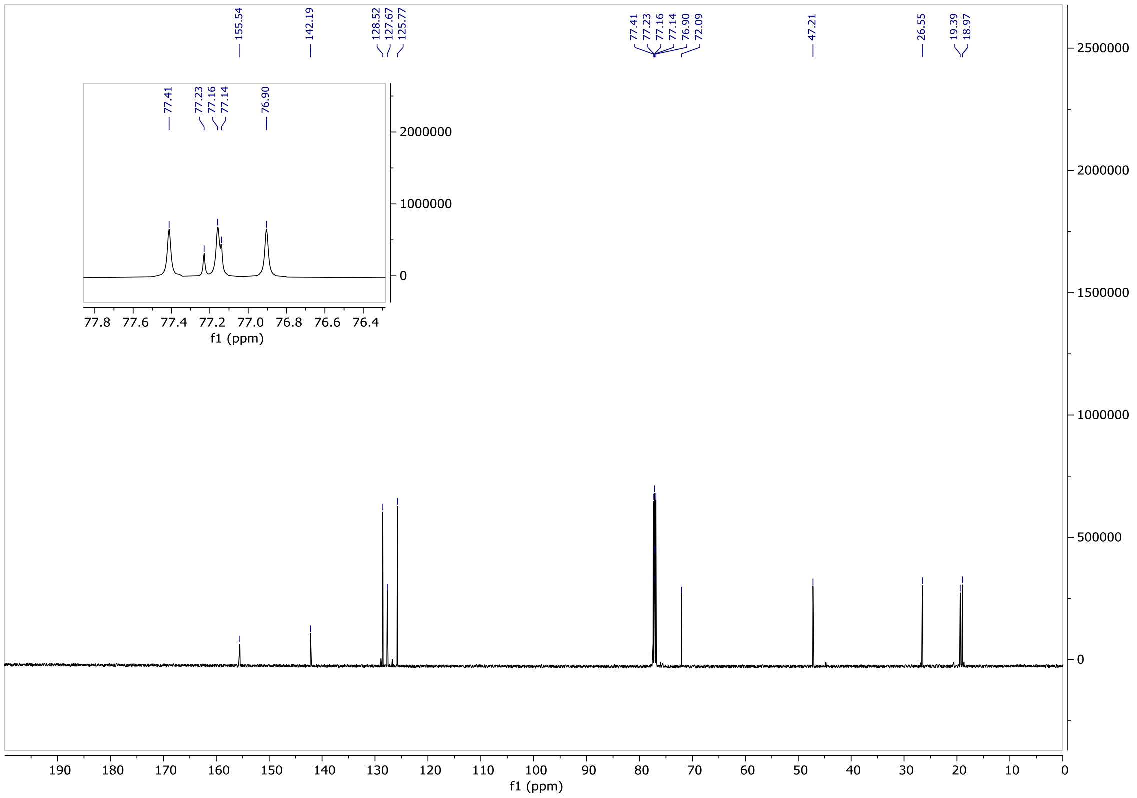
155.54
142.19
128.52
127.67
125.77
77.41
77.23
77.16
77.14
76.90
72.09
47.21
26.55
19.39
18.97
2500000
2000000
1500000
1000000
500000
0
f1 (ppm)

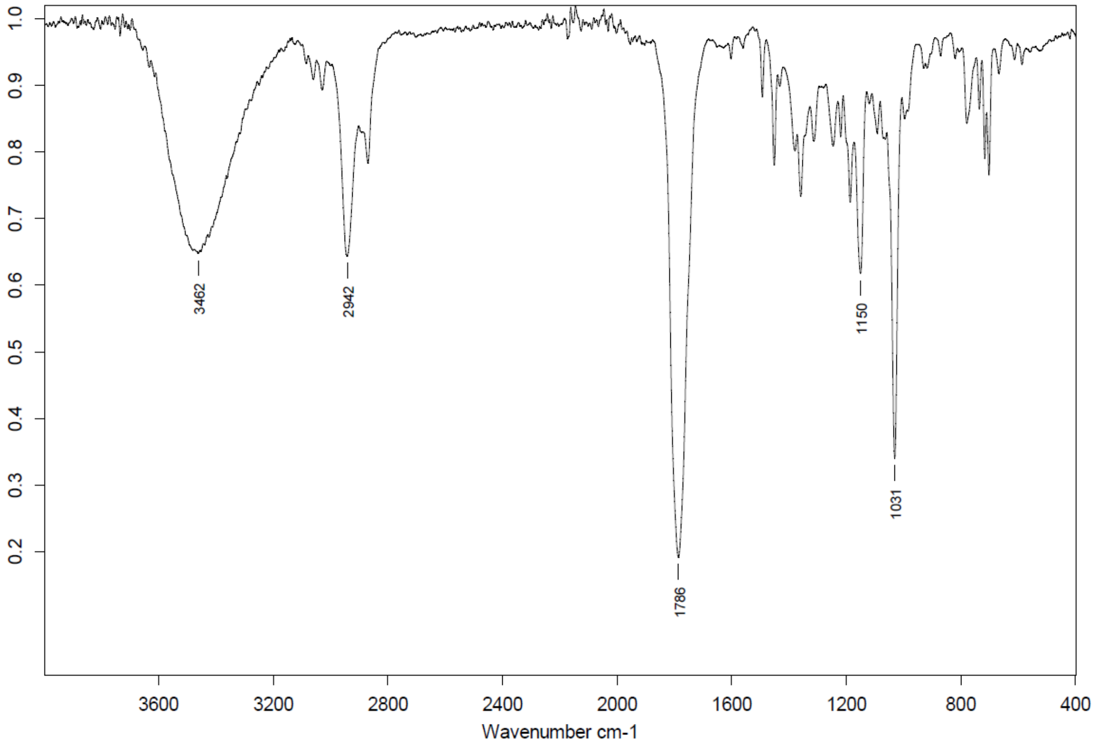
3462
2942
1786
1150
1031
Wavenumber cm-1

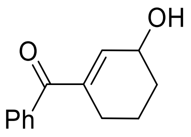
OH
O
Ph

ByP1

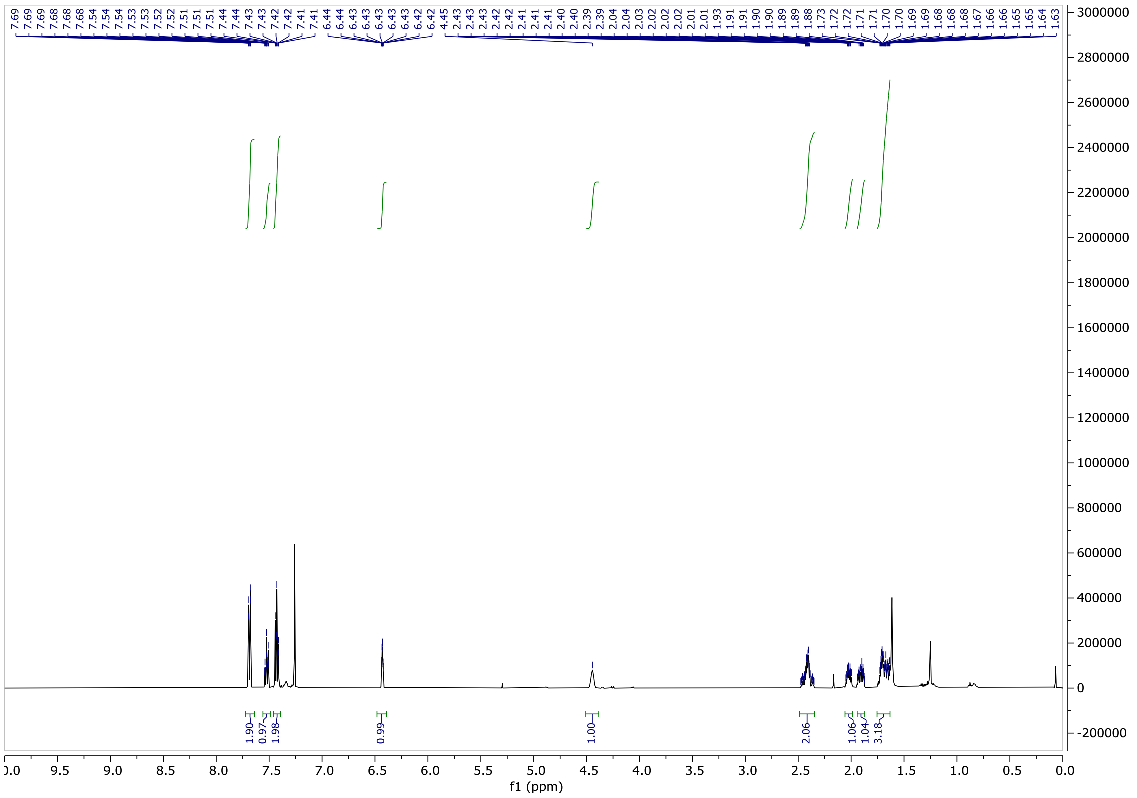
7.69
7.69
7.69
7.68
7.68
7.68
7.54
7.54
7.53
7.53
7.52
7.52
7.51
7.51
7.51
7.44
7.44
7.43
7.43
7.42
7.42
7.41
7.41
6.44
6.44
6.43
6.43
6.43
6.43
6.43
6.42
6.42
4.45
2.43
2.43
2.43
2.42
2.42
2.41
2.41
2.41
2.40
2.40
2.39
2.39
2.04
2.04
2.03
2.02
2.02
2.01
2.01
1.93
1.91
1.91
1.90
1.90
1.89
1.89
1.88
1.73
1.72
1.72
1.71
1.71
1.70
1.70
1.69
1.69
1.68
1.68
1.68
1.67
1.66
1.65
1.64
1.63
1.90
0.97
1.98
0.99
1.00
2.06
1.06
1.04
3.18
f1 (ppm)

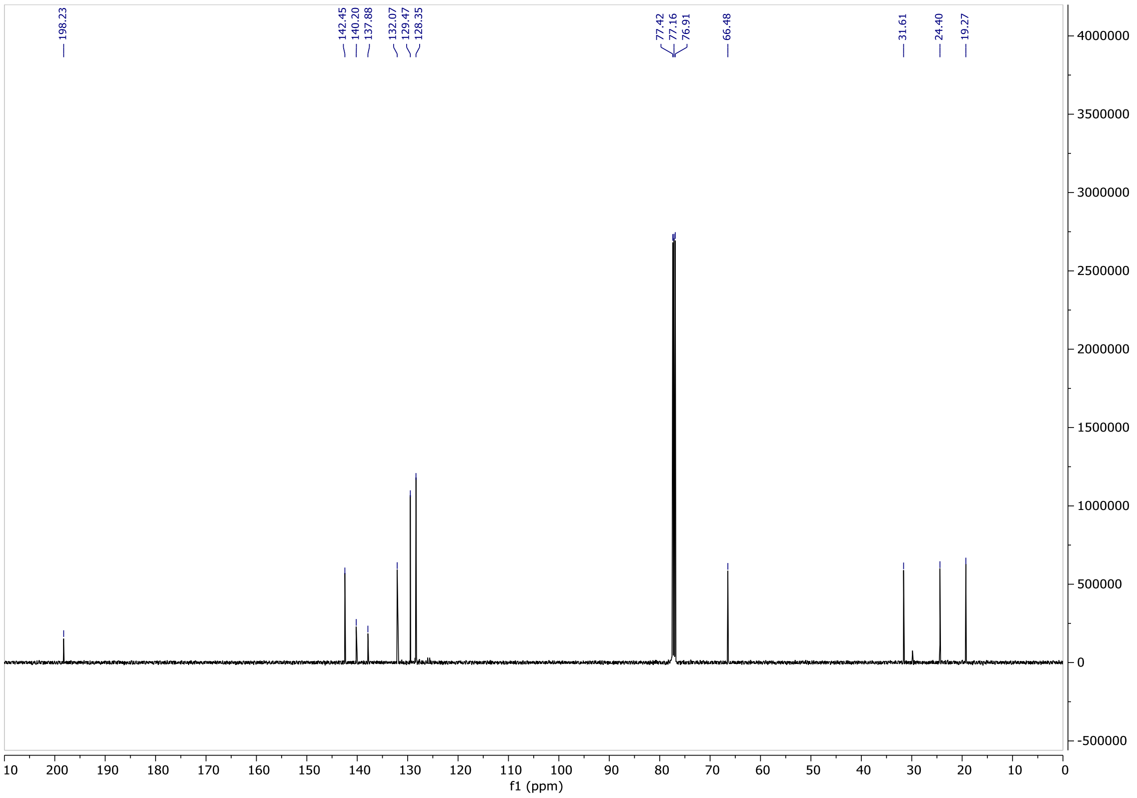
198.23
142.45
140.20
137.88
132.07
129.47
128.35
77.42
77.16
76.91
66.48
31.61
24.40
19.27
f1 (ppm)

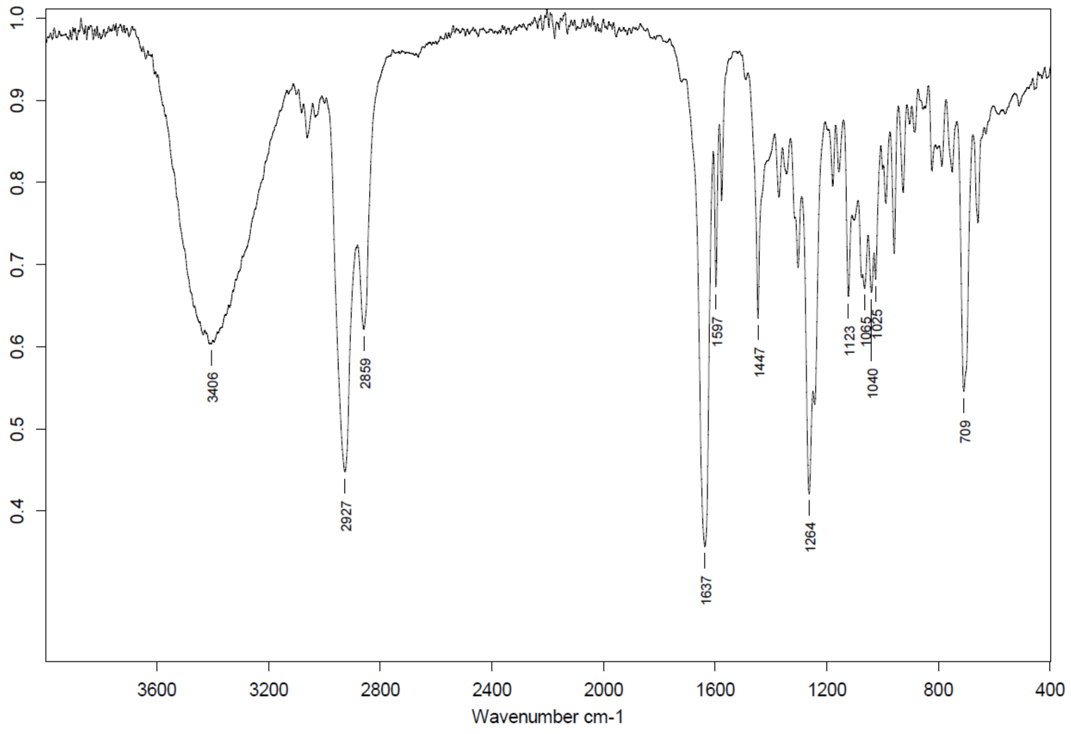

3406
2927
2859
1637
1597
1447
1264
1123
1065
1040
1025
709
Wavenumber cm-1

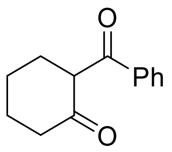

ByP2

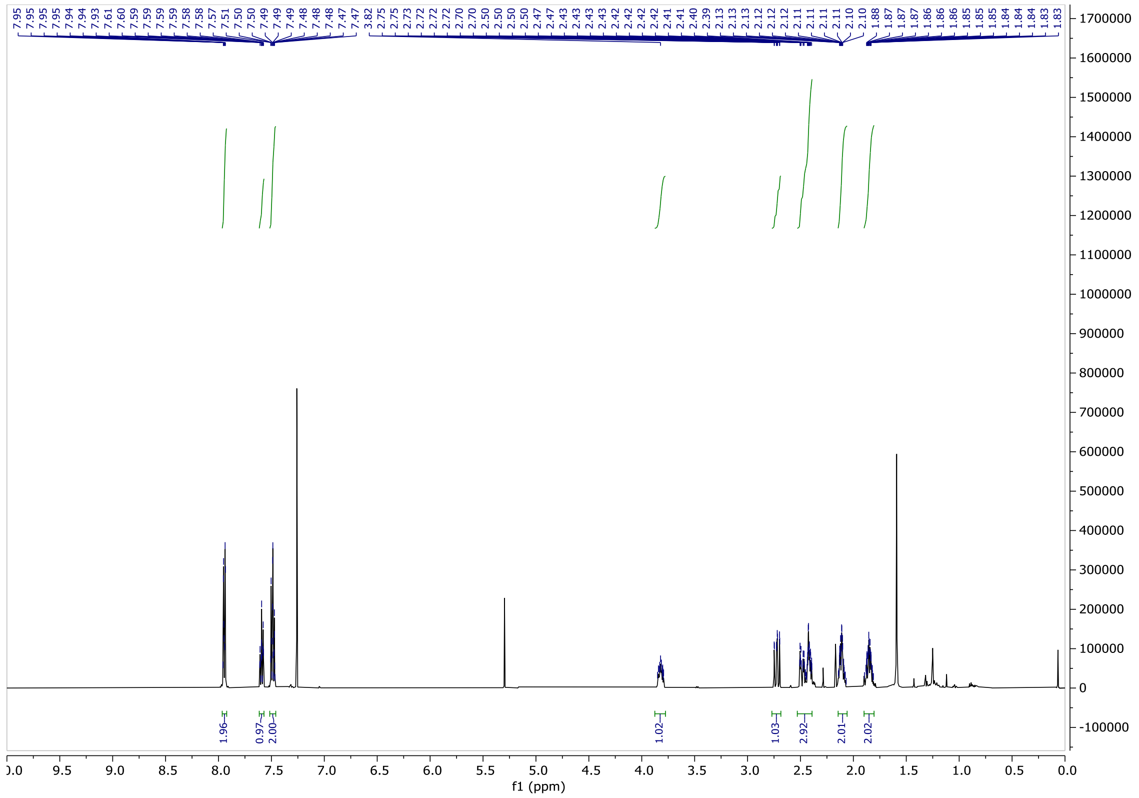

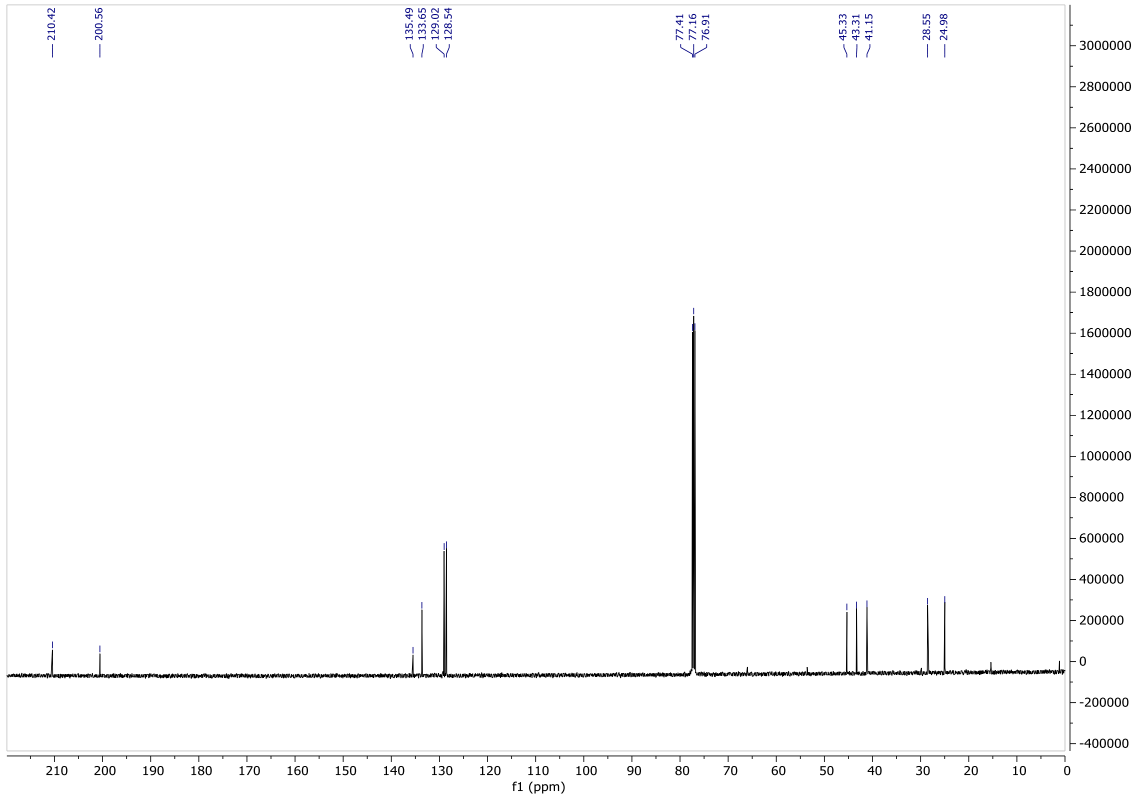

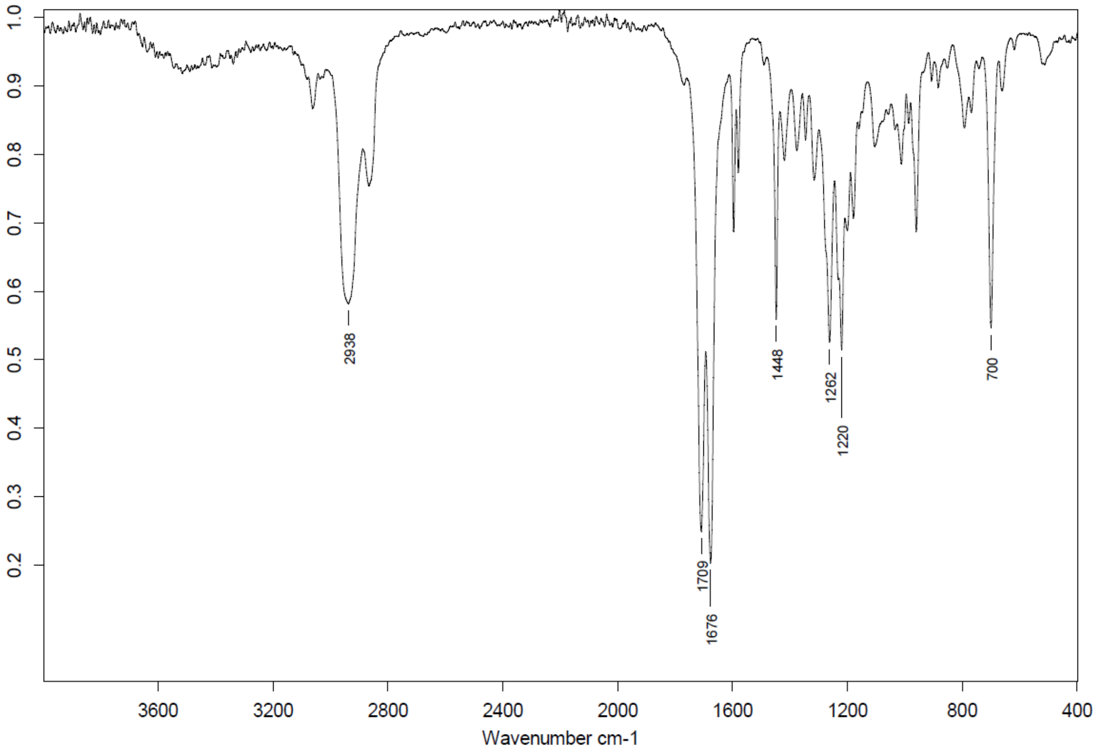

1.0
0.9
0.8
0.7
0.6
0.5
0.4
0.3
0.2
2938
1709
1676
1448
1262
1220
700
3600
3200
2800
2400
2000
1600
1200
800
400
Wavenumber cm-1

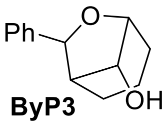

ByP3

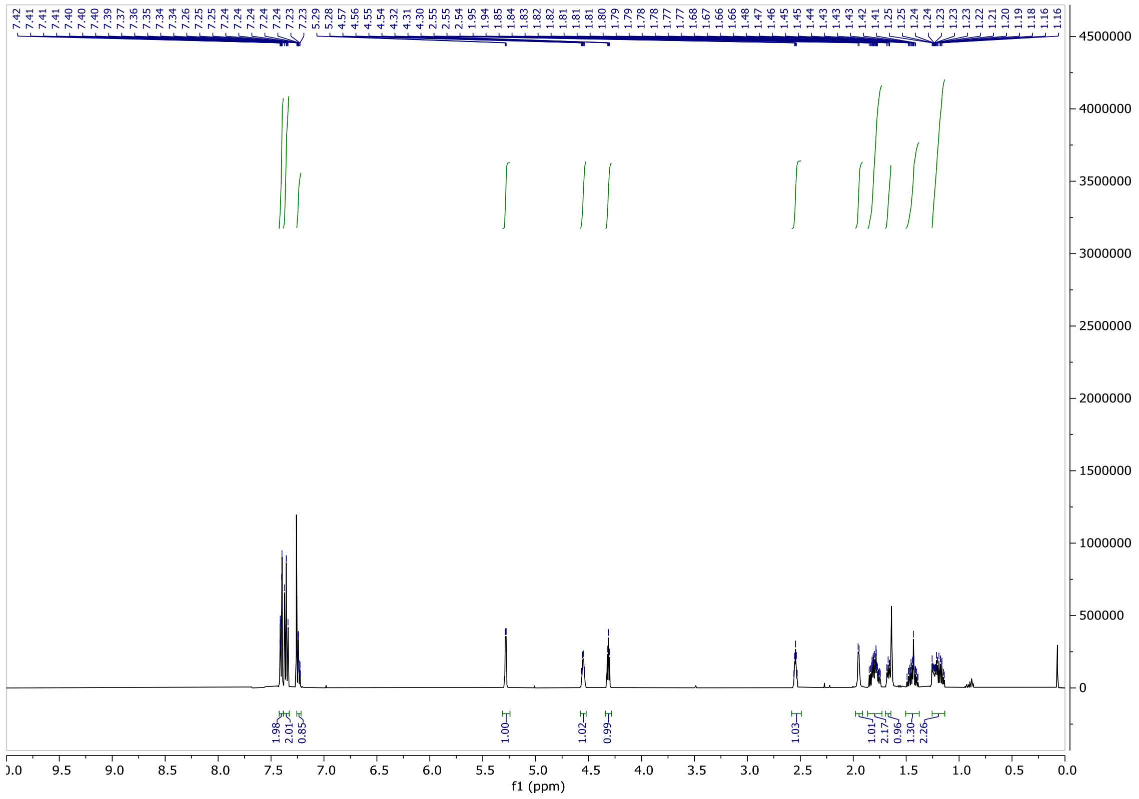

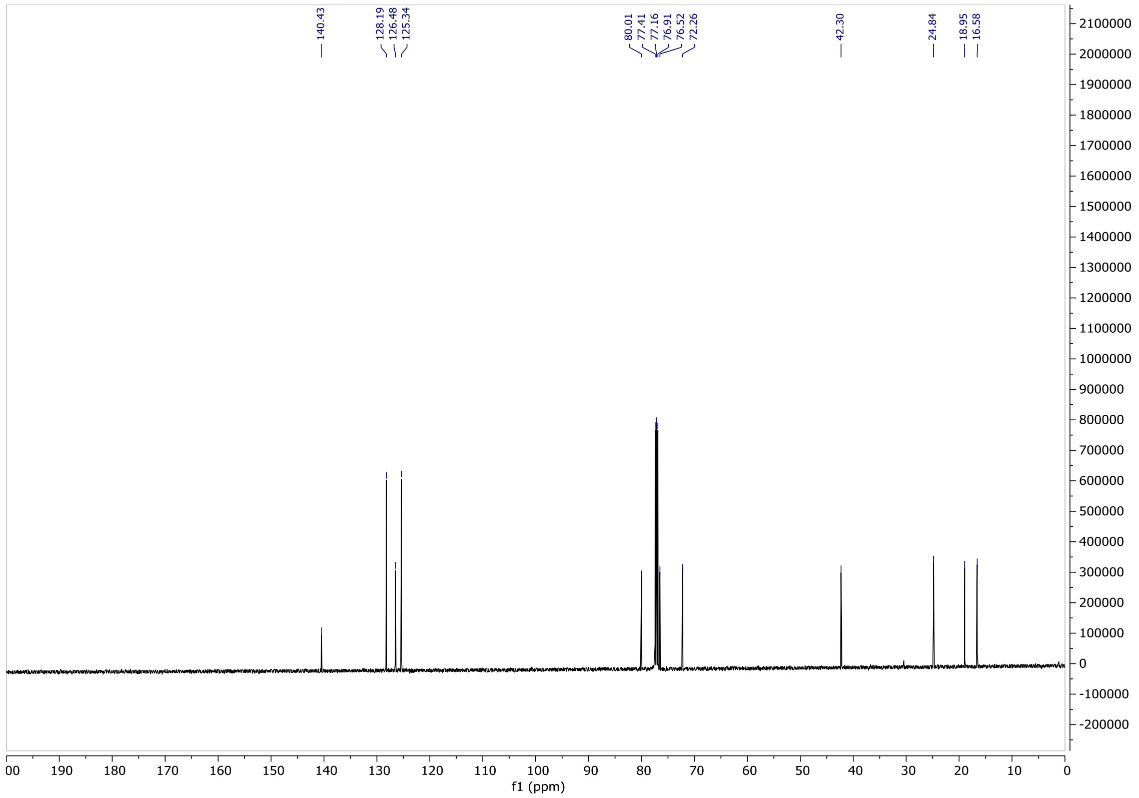

ByP4

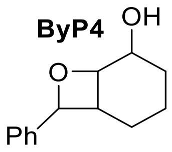
OH
O
Ph

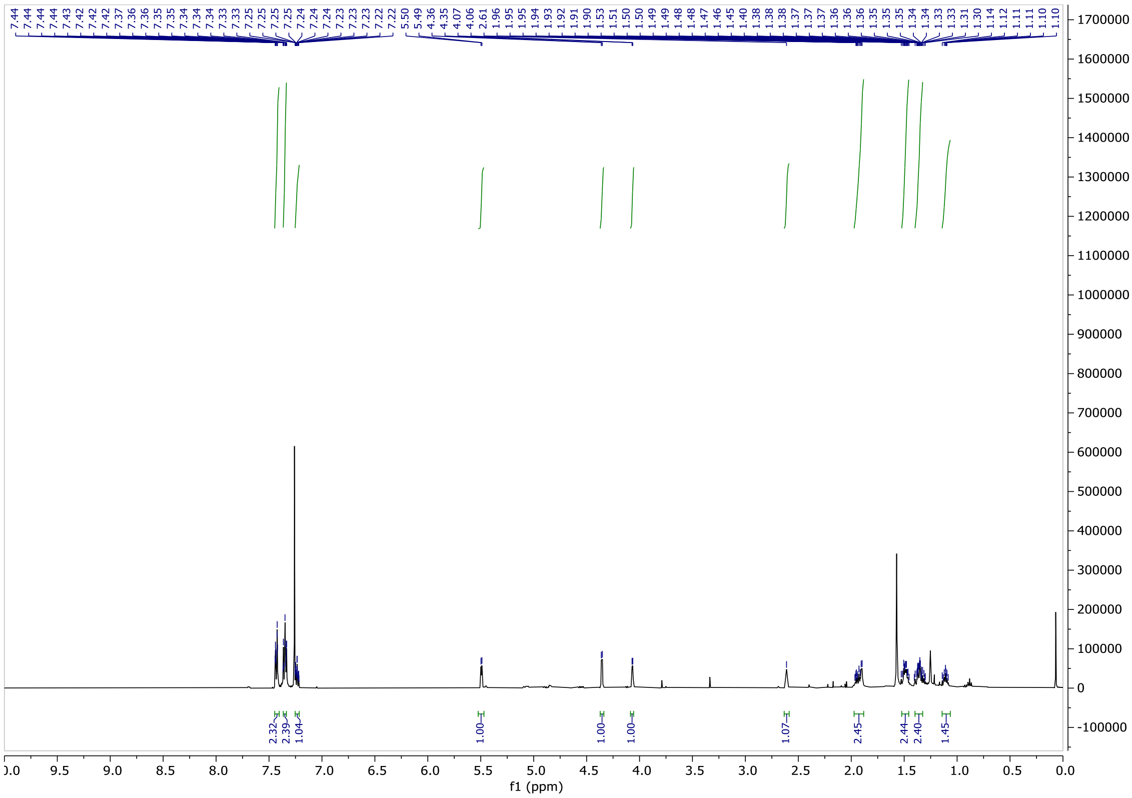
7.44
7.44
7.44
7.44
7.43
7.42
7.42
7.42
7.37
7.36
7.36
7.35
7.35
7.34
7.34
7.34
7.33
7.33
7.25
7.25
7.25
7.24
7.24
7.24
7.24
7.23
7.23
7.23
7.22
7.22
5.50
5.49
4.36
4.35
4.07
4.06
2.61
1.96
1.95
1.95
1.94
1.93
1.92
1.91
1.90
1.53
1.51
1.50
1.50
1.49
1.49
1.48
1.48
1.47
1.46
1.45
1.40
1.38
1.38
1.38
1.38
1.37
1.37
1.37
1.36
1.36
1.36
1.35
1.35
1.35
1.34
1.34
1.33
1.33
1.31
1.30
1.14
1.12
1.11
1.11
1.10
1.10
2.32
2.39
1.04
1.00
1.00
1.00
1.07
2.45
2.44
2.40
1.45
f1 (ppm)

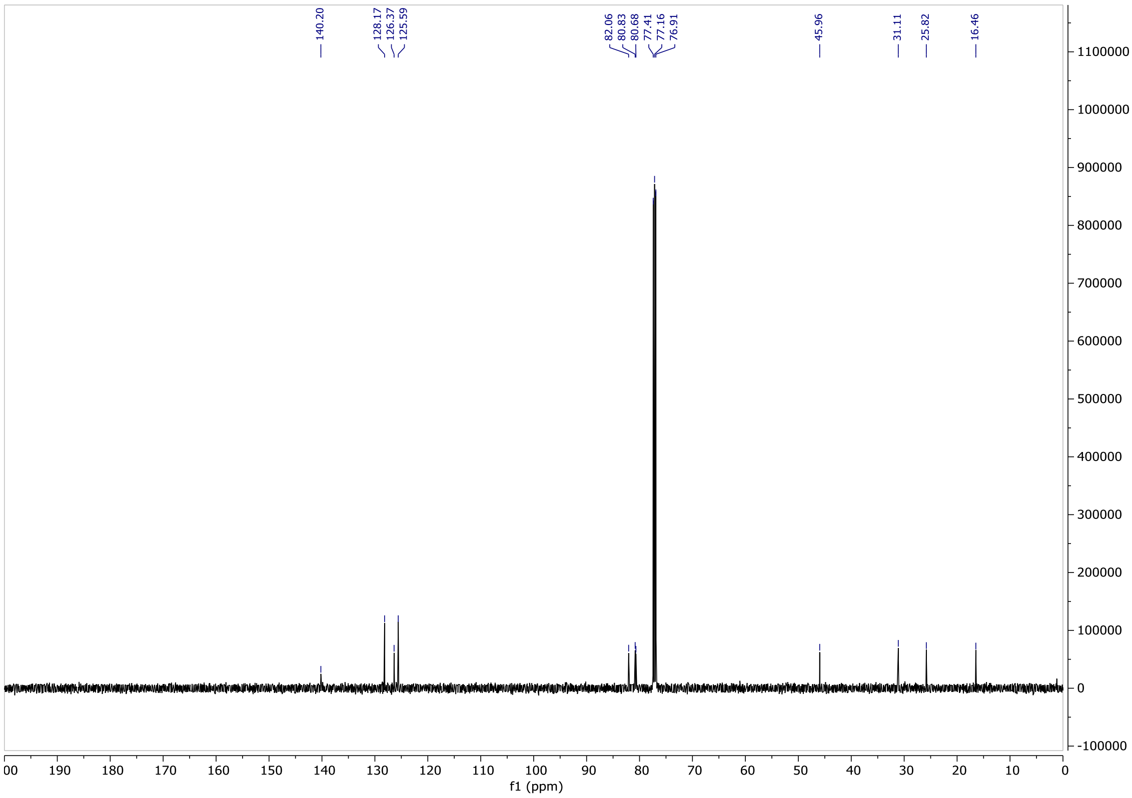
140.20
128.17
126.37
125.59
82.06
80.83
80.68
77.41
77.16
76.91
45.96
31.11
25.82
16.46
f1 (ppm)

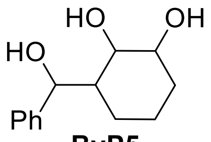

ByP5

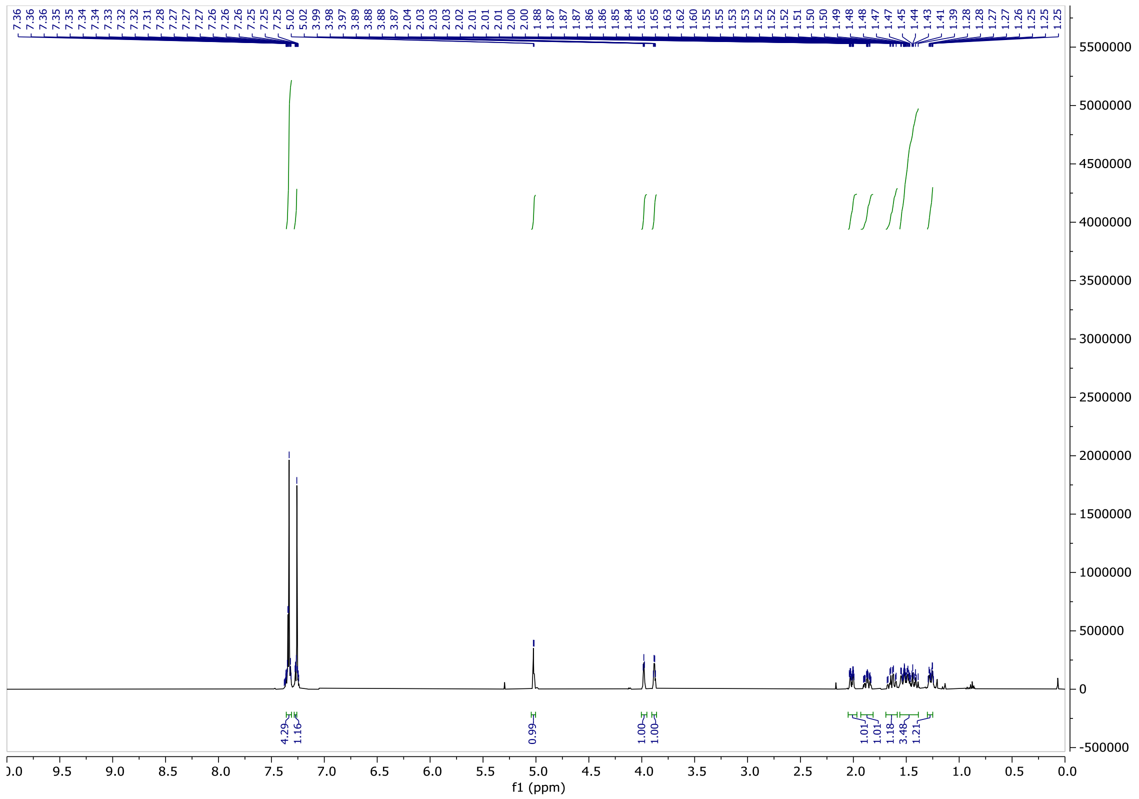

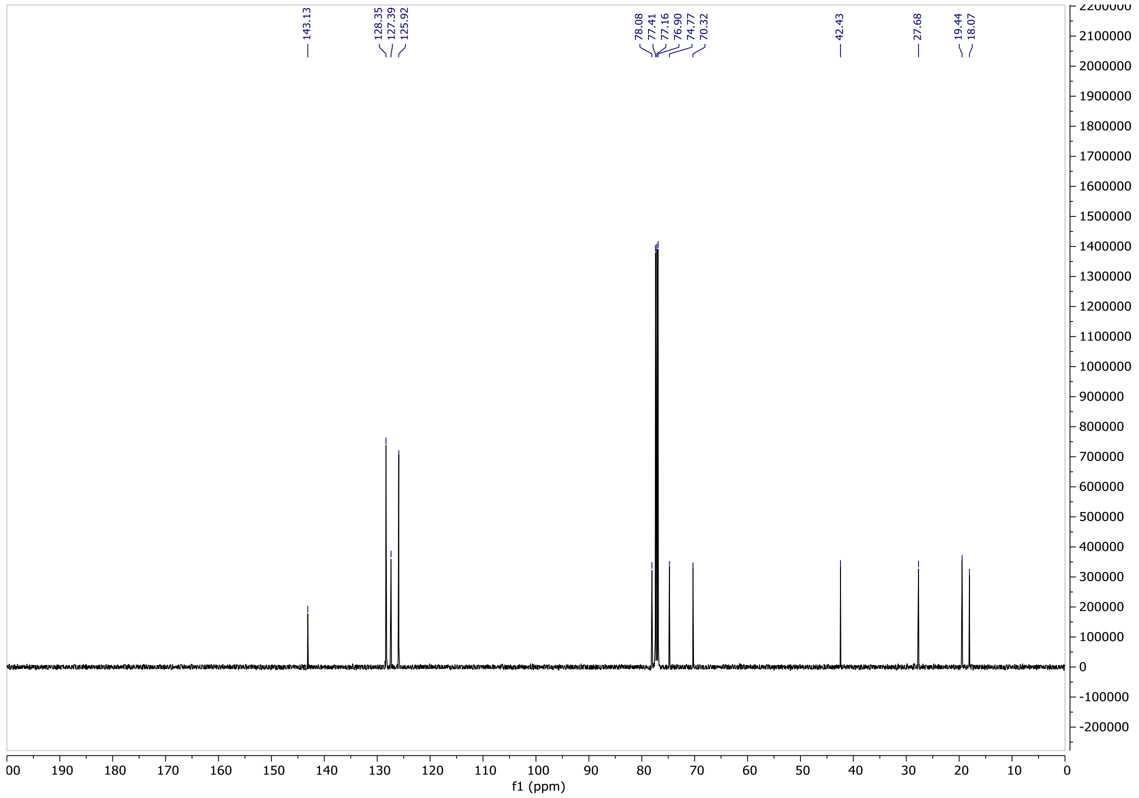

S127. ROP studies

General considerations: All water-sensitive operations were carried out under a nitrogen atmosphere using an MBraun glovebox, standard vacuum line, and Schlenk techniques. Solvents were purchased from Sigma-Aldrich (HPLC grade) and dried using an MBraun MBSPS800 purification system. Benzyl alcohol was dried over CaH_2 and distilled under reduced pressure. TBD and the 6-membered cyclic carbonate monomer were dissolved in DCM and THF separately, stirred over calcium hydride, filtered and dried in vacuum prior to use. Differential scanning calorimetry (DSC) analyses for determination of the glass transition temperatures (T_g) were measured under a N_2 atmosphere using a Mettler Toledo equipment (model DSC822e). Samples were weighed into 40 μL aluminum crucibles and subjected to two heating cycles at a heating rate of 10 °C/min. Thermogravimetric analyses (TGA) were recorded under air atmosphere using Mettler Toledo equipment (model TGA/SDTA851). Samples were weighed into 40 µL aluminum crucibles and heated to 600 ºC at a heating rate of 10 °C/min. Gel permeation chromatography (GPC) measurements were performed using an Agilent 1200 series HPLC system, equipped with PSS SDV Analytical linear M GPC column (8 × 300 mm; 5 μm particle size) in tetrahydrofuran at 30 °C at a flow rate of 1 $mL\cdot min^{-1}$. Samples were analyzed at a concentration of 1 $mg\cdot mL^{-1}$ after filtration through a 0.45 μm pore-size membrane. M_n, M_w, and $Đ$ data were derived from the RI signal by a calibration curve based on polystyrene standards (PS from Polymer Standards Service) for the analysis of the polymers.

Matrix-assisted laser desorption/ionization time-of-flight mass spectrometry (MALDI-TOF) was performed using a BRUKER Autoflex spectrometer under the following conditions for the sample preparation: 1 mg of polymer was dissolved in 1 mL of CH_2Cl_2 (1 mg mL^{-1}). 5 µL of this solution was added to a solution of dithranol in CH_2Cl_2 as a matrix (25 µL, 10 mg mL^{-1}) and CF_3COONa in THF as an additive (1 µL, 1 mg mL^{-1}).

Typical ROP procedure:

In a glovebox, monomer **P23** (20 mg, 82 μmol) was introduced into a vial equipped with a magnetic stirrer. Then, benzyl alcohol (27 *μ*L from a 59.2 mM stock solution in toluene, 1.62 μmol, 2.0 mol%) and TBD (55 *μ*L of a 29.5 mM stock solution in toluene, 1.62 μmol, 2 mol%) were added in this sequence. Once out of the glovebox, the vial was sealed with electric insulator tape. After stirring for 20 h, the reaction mixture was quenched with benzoic acid (30 *μ*L of a 83.5 mM stock solution in toluene, 1.22 *μ*mol) and a sample was analyzed by ^{1}H NMR ($CDCl_3$) to determine the monomer conversion. The polymer product was collected by precipitation from DCM/MeOH following filtration. The conversion of **P23** under these conditions was 96%. M_n (GPC) = 5.9 kDa, $Đ$ = 1.32 (Table 2, entry 3).

See the following pages for analytical details

Copies of NMR, IR, TGA, DSC and MALDI-MS spectra for the polycarbonate related to entry 4, Table 2

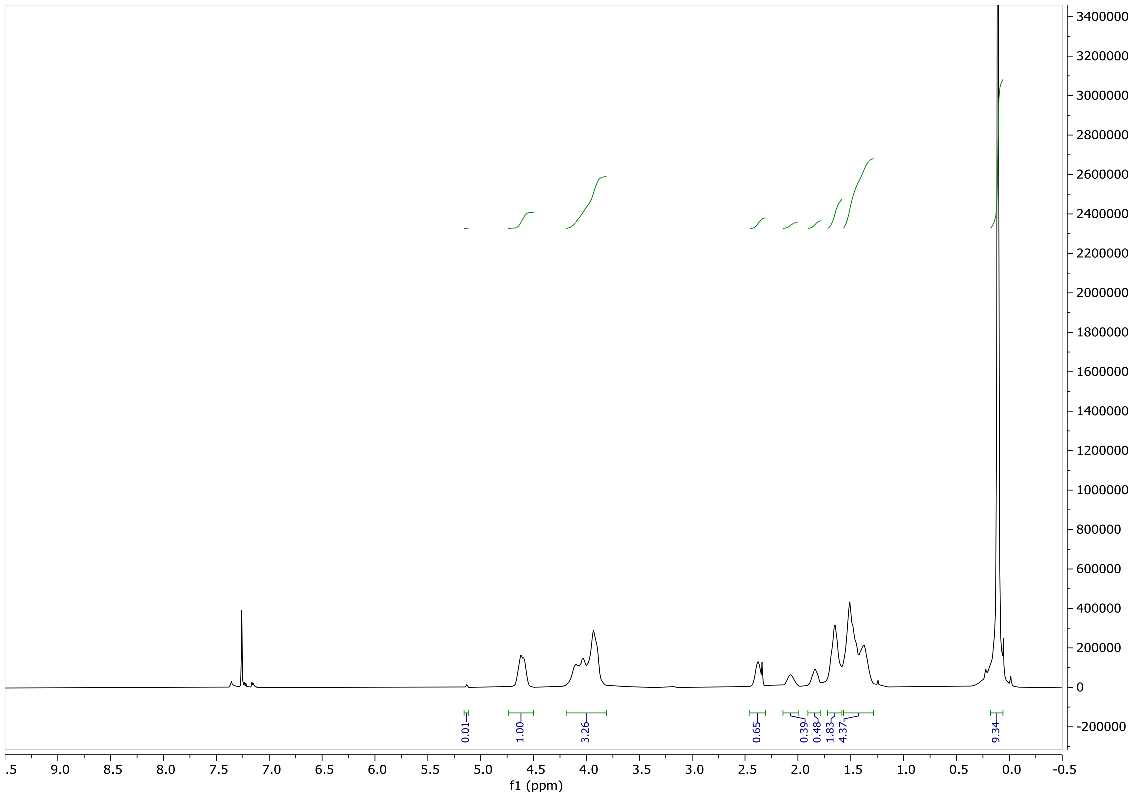

^{1}H NMR spectrum (500 MHz, rt, $CDCl_3$)

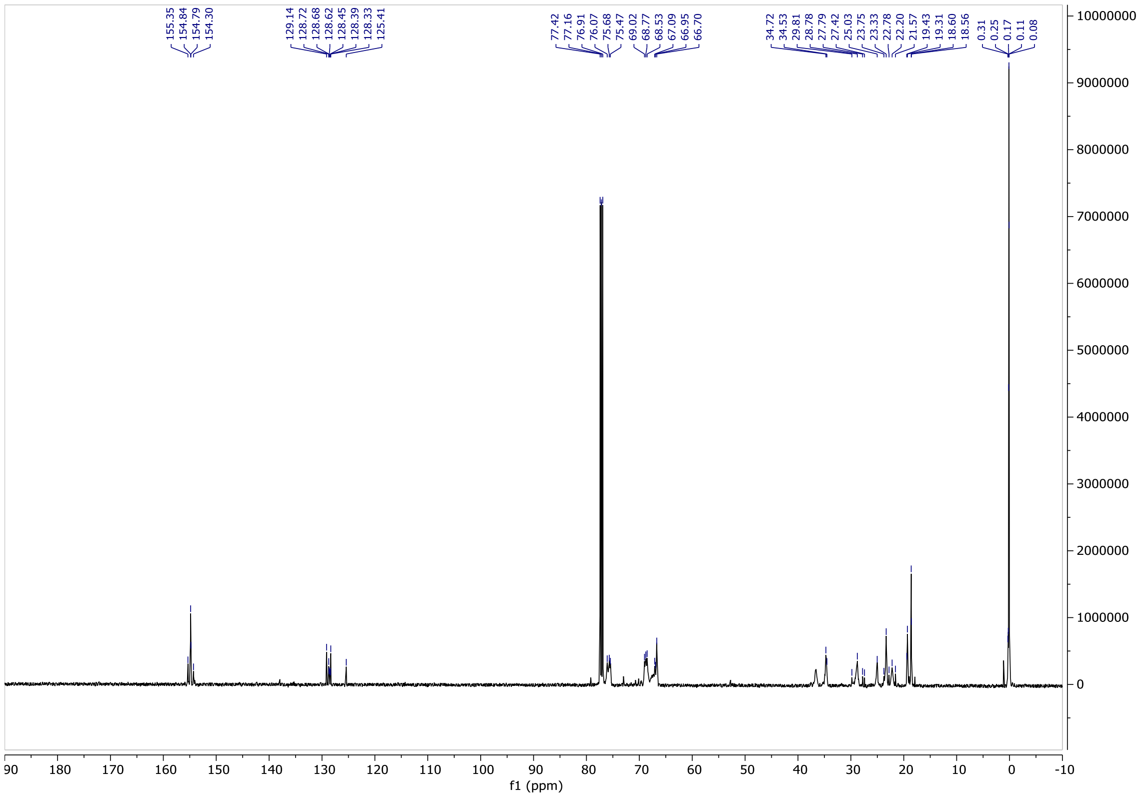

^{13}C NMR spectrum (500 MHz, rt, $CDCl_3$)

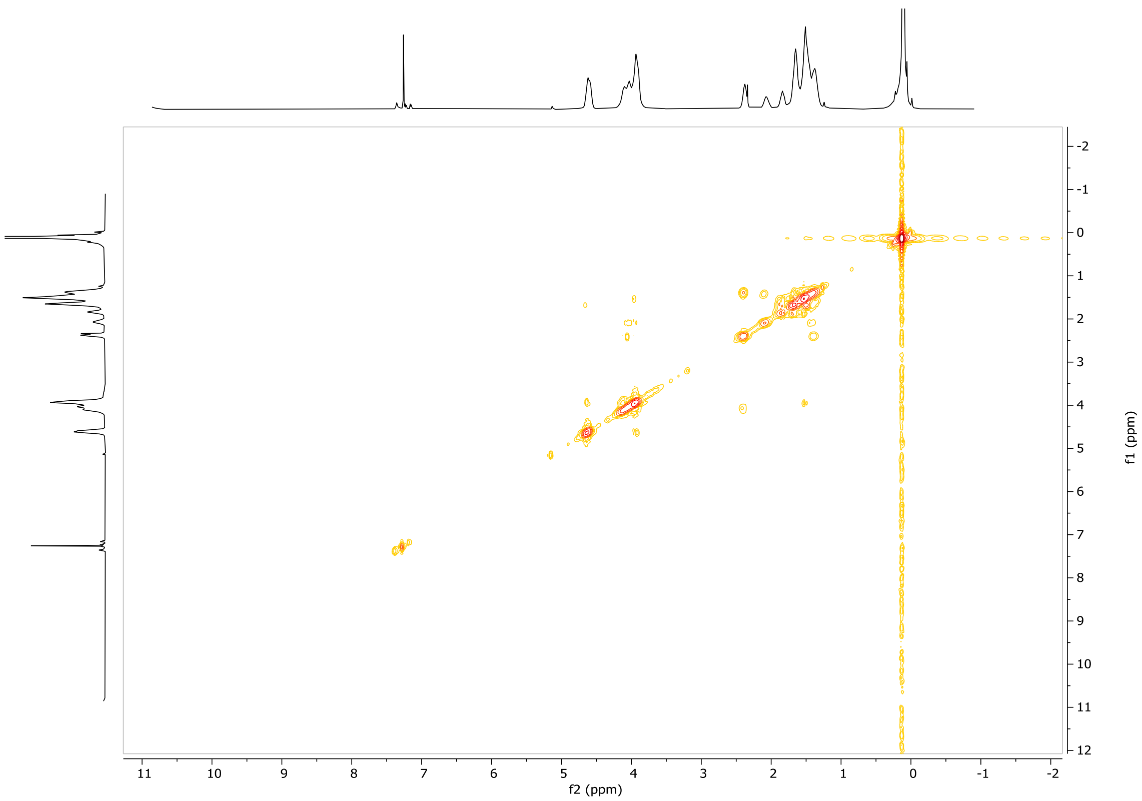

^{1}H-^{1}H COSY NMR spectrum (500 MHz, rt, $CDCl_3$)

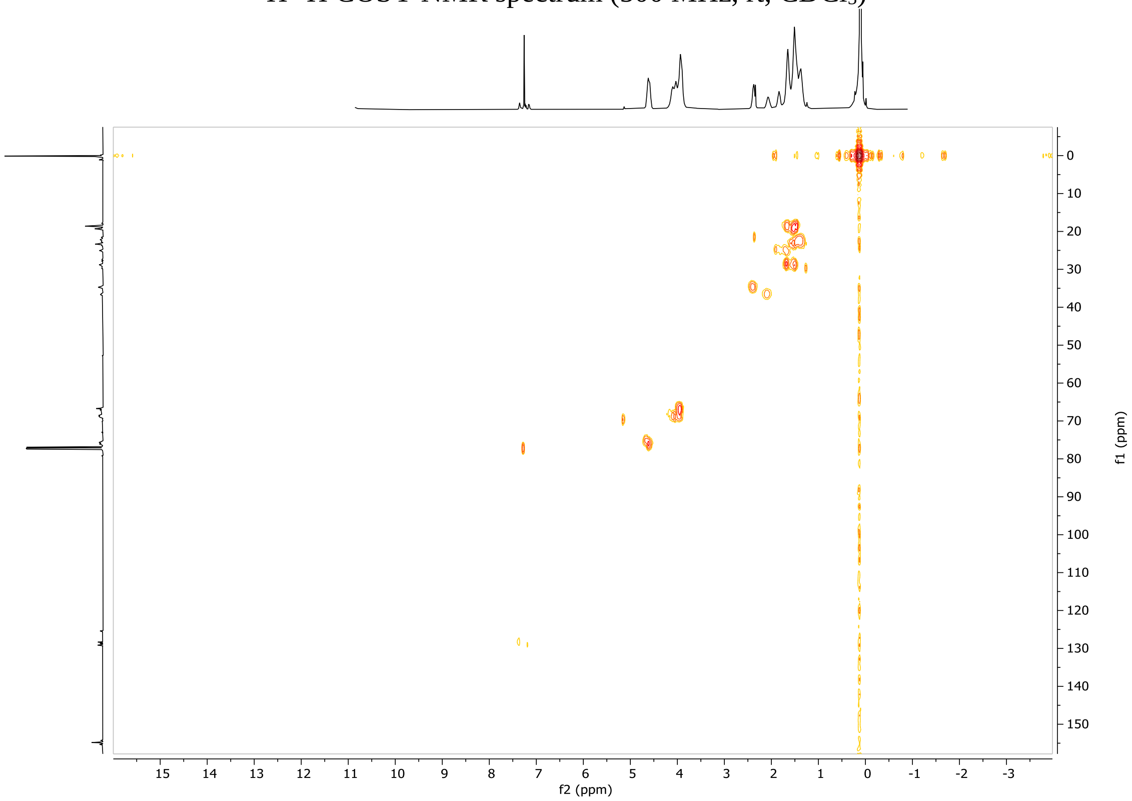

^{1}H-^{13}C HMBC NMR spectrum (500 MHz, rt, $CDCl_3$)

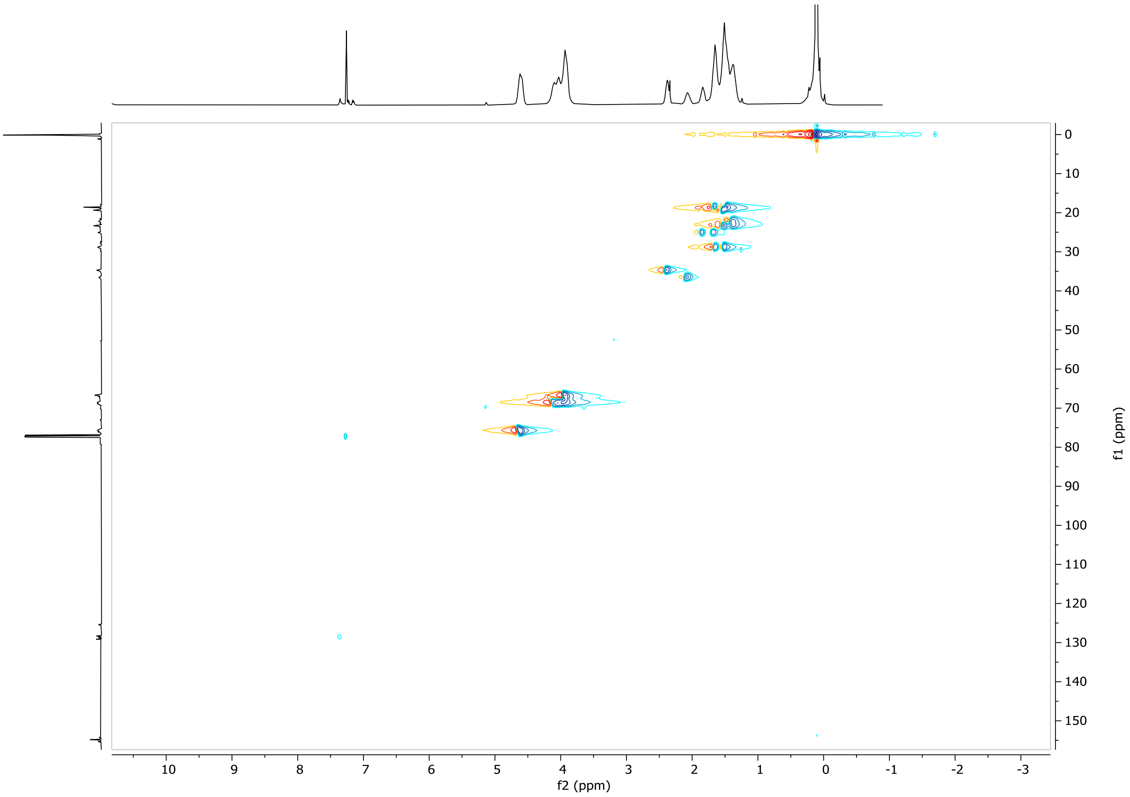

^{1}H-^{13}C HSQC NMR spectrum (500 MHz, rt, $CDCl_3$)

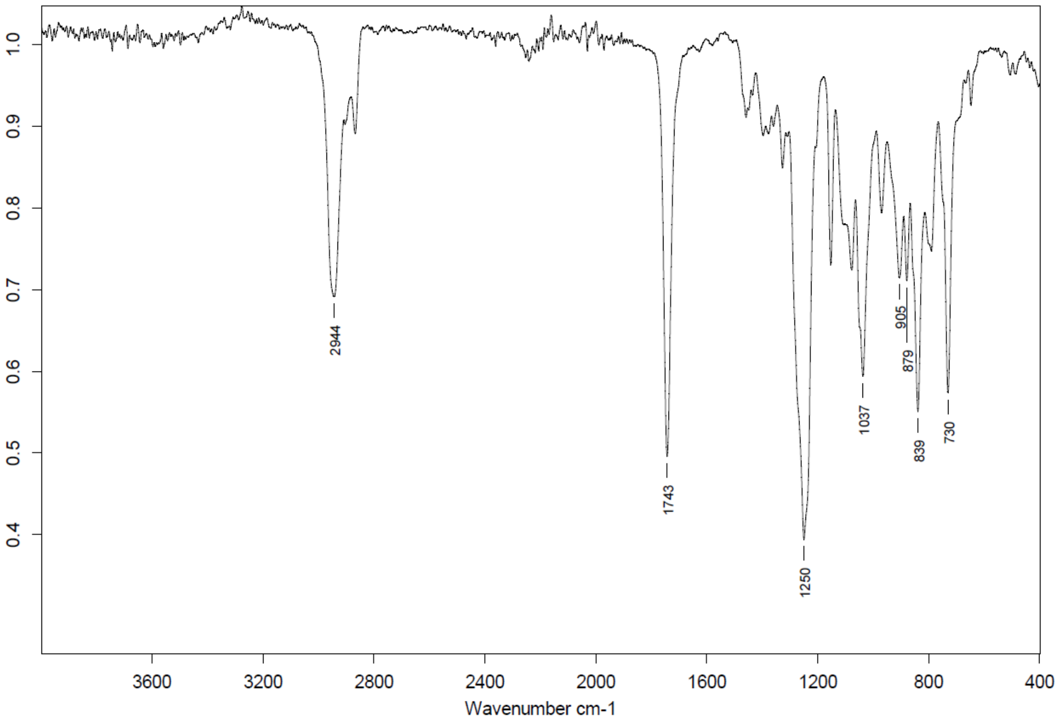

IR spectrum (neat)

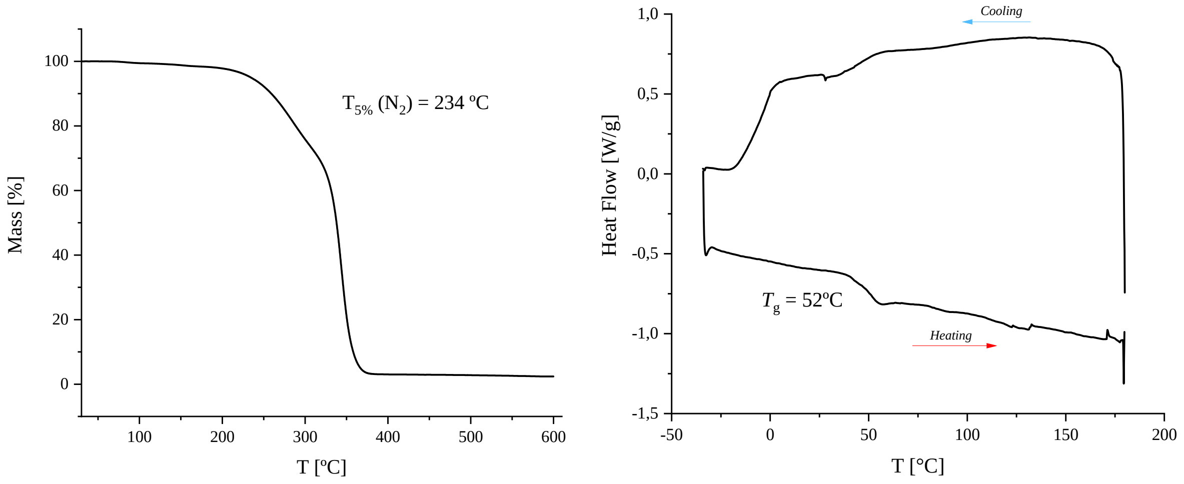

TGA thermogram (left) and **DSC** analysis (right)

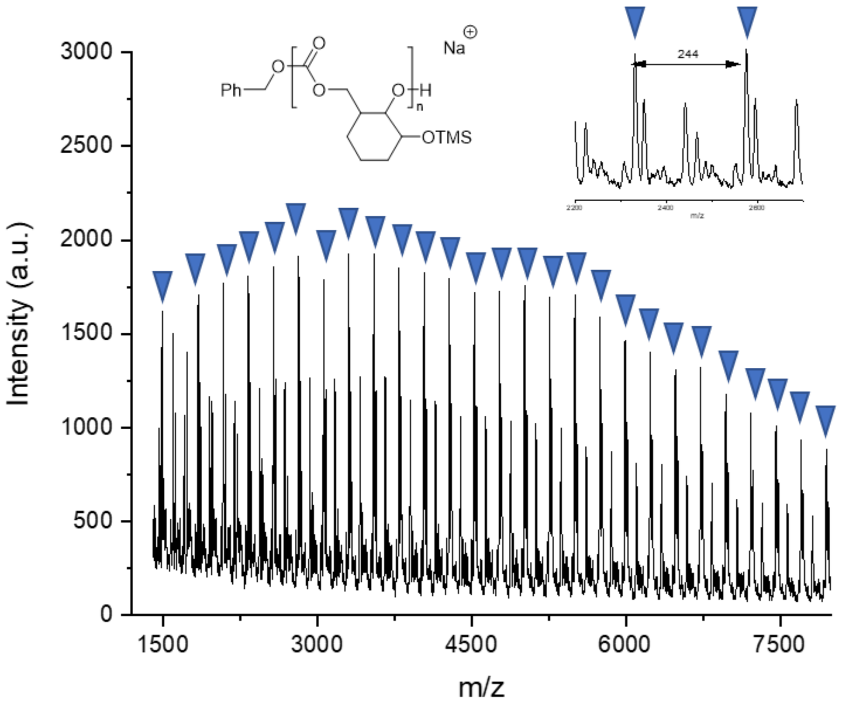

MALDI-MS spectrum

Copies of GPC traces:

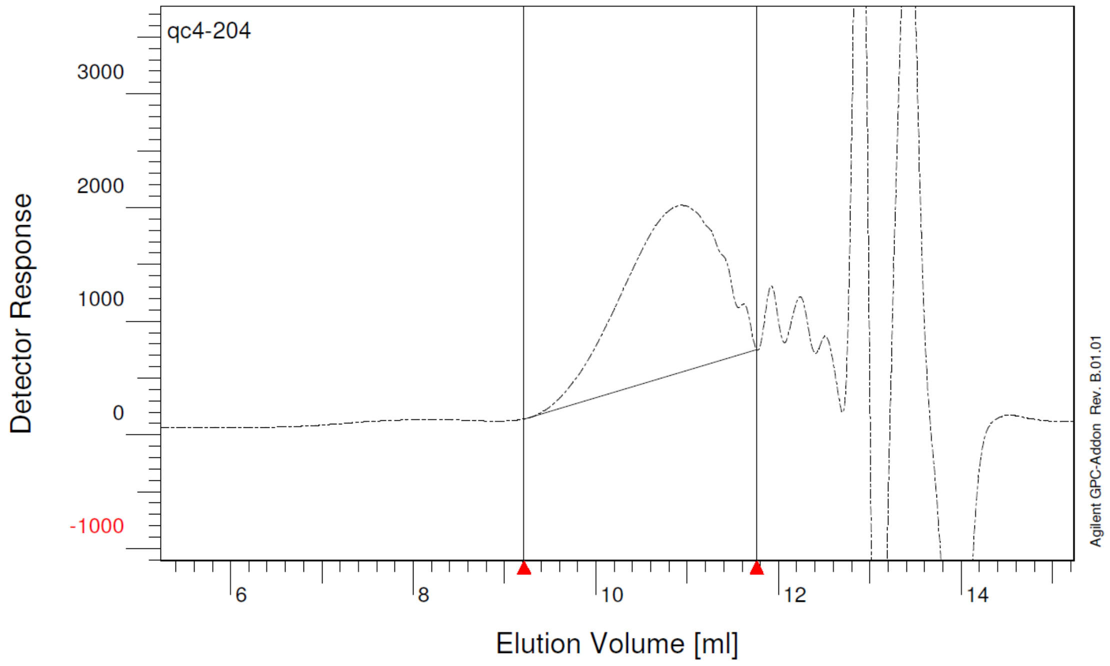

M_n (Da)	M_w (Da)	M_z (Da)	M_p (Da)	*Đ*
$1.6995 \cdot 10^3$	$2.5037 \cdot 10^3$	$3.6020 \cdot 10^3$	$1.8712 \cdot 10^3$	1.4732

Note: GPC trace related to entry 1, Table 2

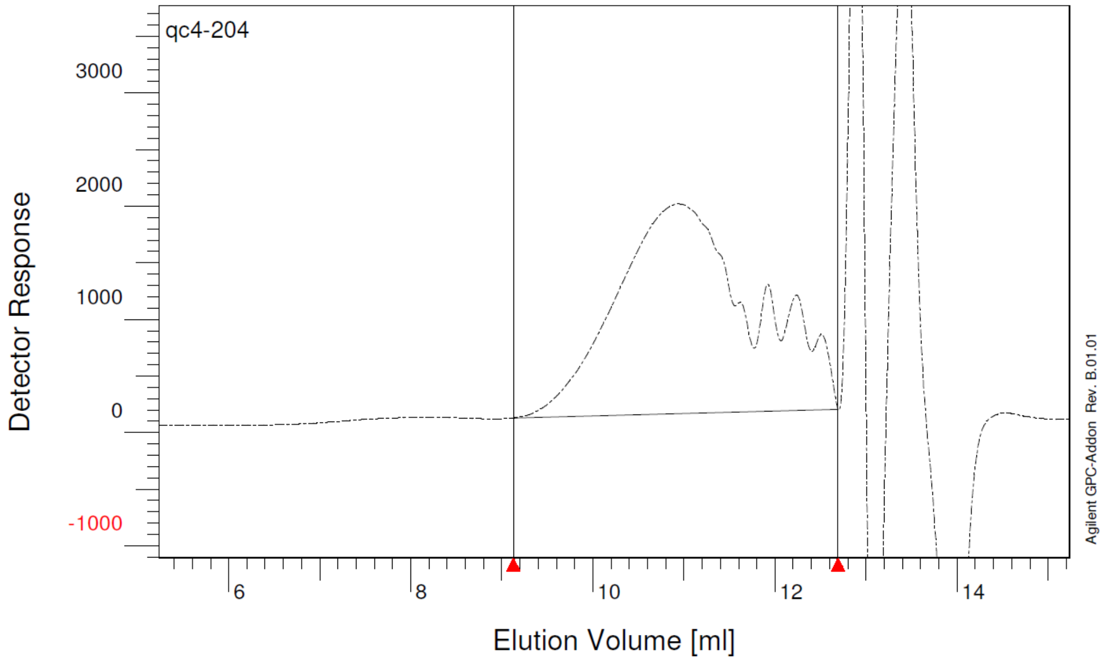

M_n (Da)	M_w (Da)	M_z (Da)	M_p (Da)	*Đ*
$5.2863 \cdot 10^2$	$2.0263 \cdot 10^3$	$3.8824 \cdot 10^3$	$1.7528 \cdot 10^3$	3.8332

Note: GPC trace related to entry 2, Table 2

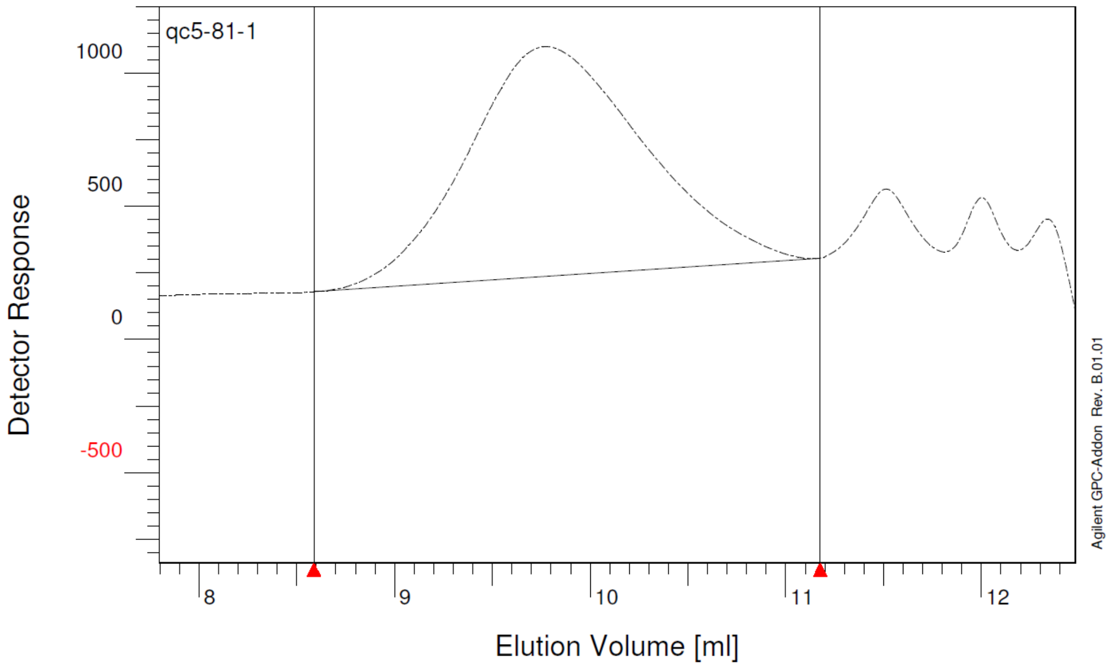

M_n (Da)	M_w (Da)	M_z (Da)	M_p (Da)	*Đ*
$5.8889 \cdot 10^3$	$7.9033 \cdot 10^3$	$1.0436 \cdot 10^4$	$7.5619 \cdot 10^3$	1.3421

Note: GPC trace related to entry 3, Table 2

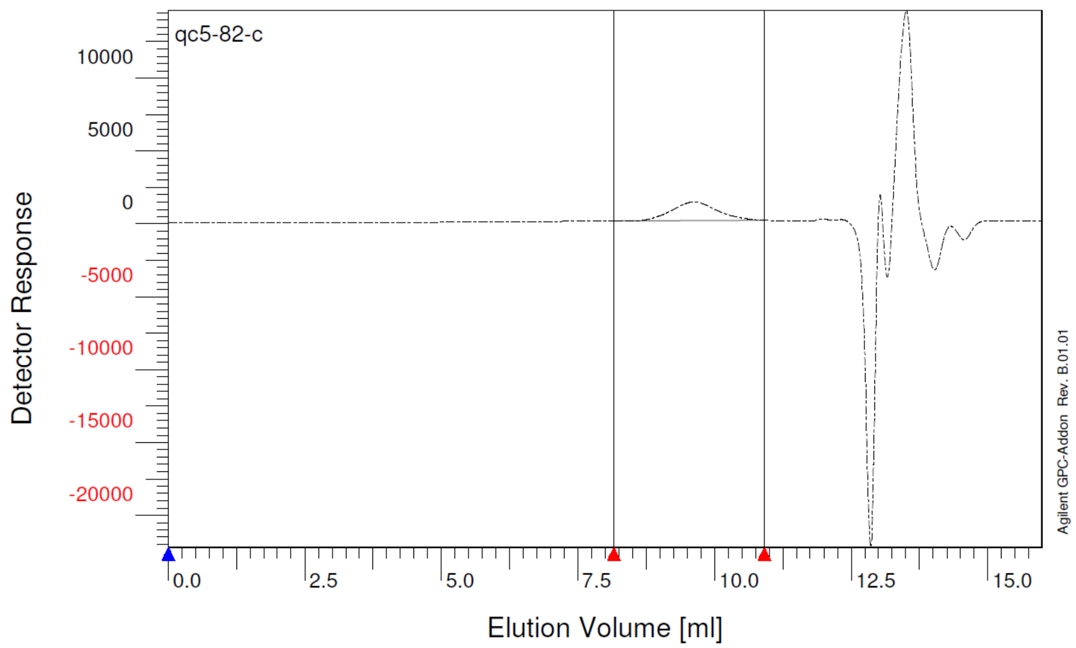

M_n (Da)	M_w (Da)	M_z (Da)	M_p (Da)	$Đ$
$7.7719 \cdot 10^3$	$1.0278 \cdot 10^3$	$1.3424 \cdot 10^4$	$9.1887 \cdot 10^3$	1.3225

Note: GPC trace related to entry 4, Table 2

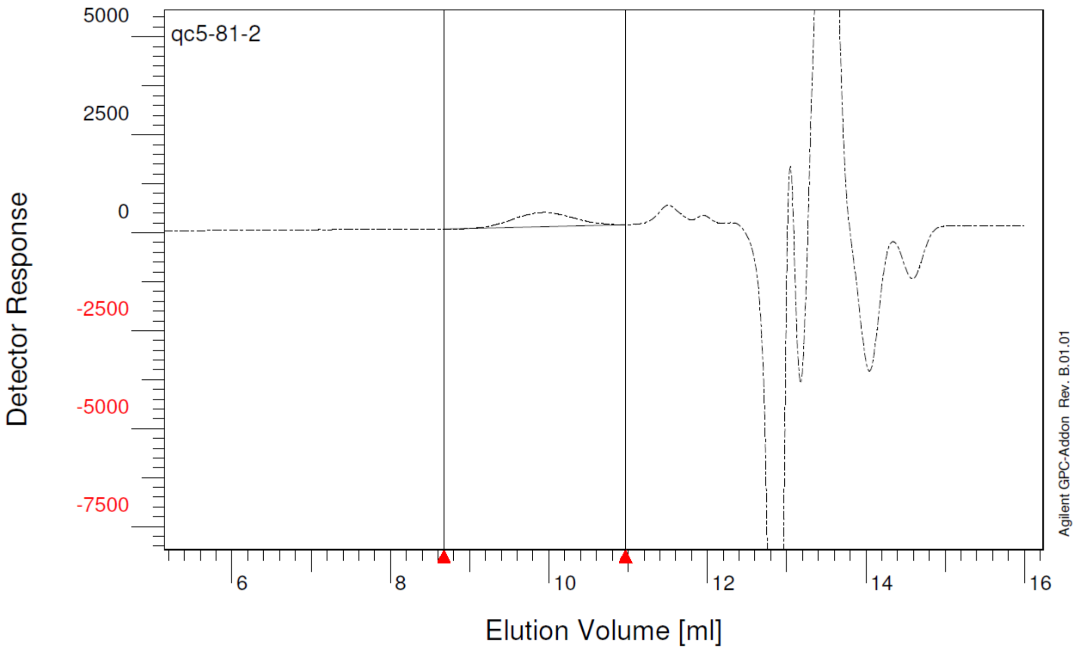

M_n (Da)	M_w (Da)	M_z (Da)	M_p (Da)	*Đ*
$5.5397 \cdot 10^3$	$6.6688 \cdot 10^3$	$8.0104 \cdot 10^4$	$6.1413 \cdot 10^3$	1.2038

Note: GPC trace related to entry 5, Table 2

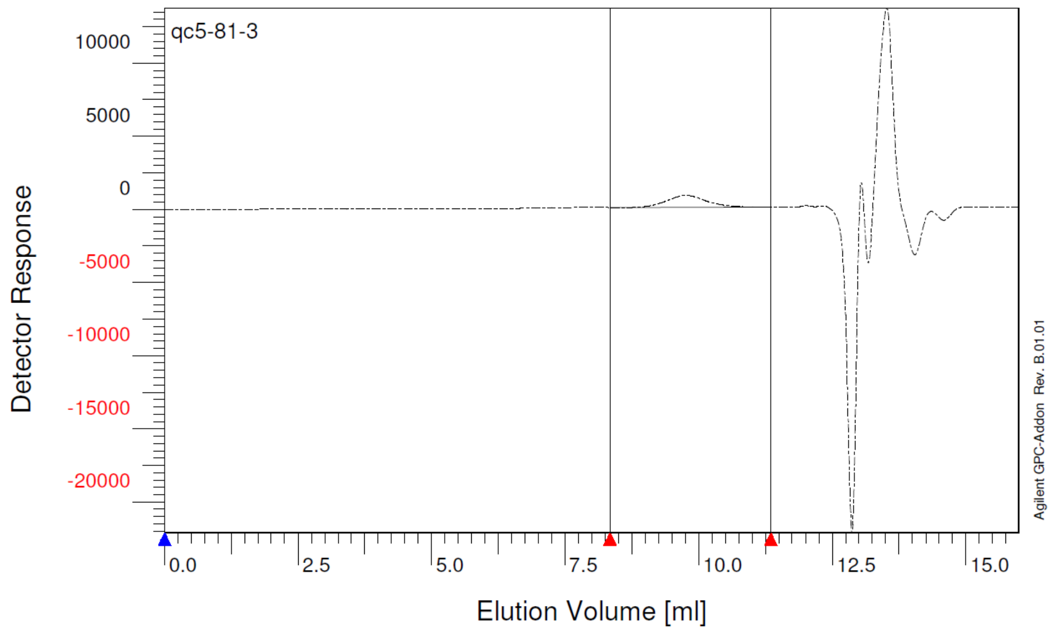

M_n (Da)	M_w (Da)	M_z (Da)	M_p (Da)	$Đ$
$6.3854 \cdot 10^3$	$8.1276 \cdot 10^3$	$1.0087 \cdot 10^4$	$7.5619 \cdot 10^3$	1.2728

Note: GPC trace related to entry 6, Table 2

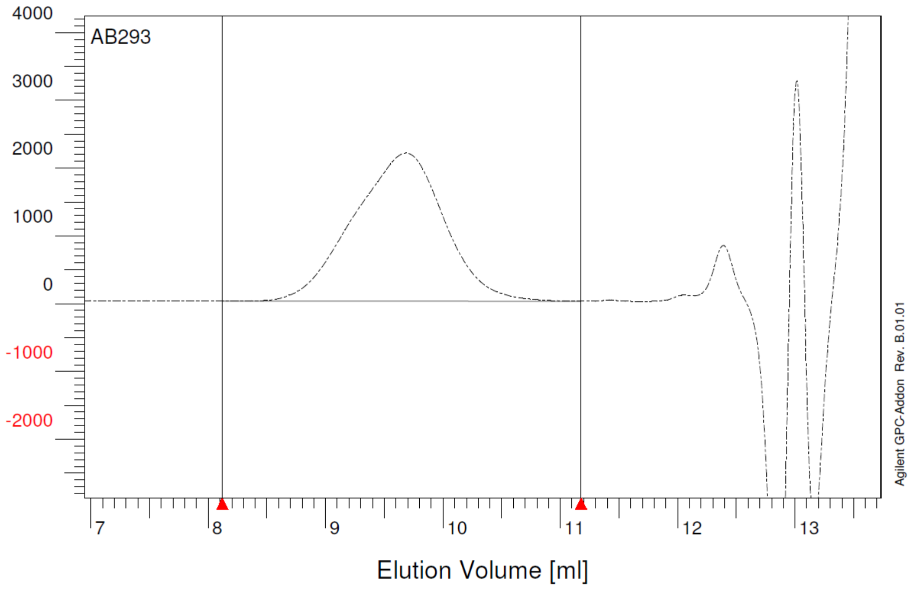

M_n (Da)	M_w (Da)	M_z (Da)	M_p (Da)	$Đ$
$8.6347 \cdot 10^3$	$1.1007 \cdot 10^4$	$1.4064 \cdot 10^4$	$8.8069 \cdot 10^3$	1.2748

Note: GPC trace related to entry 7, Table 2

S140. X-ray molecular structures

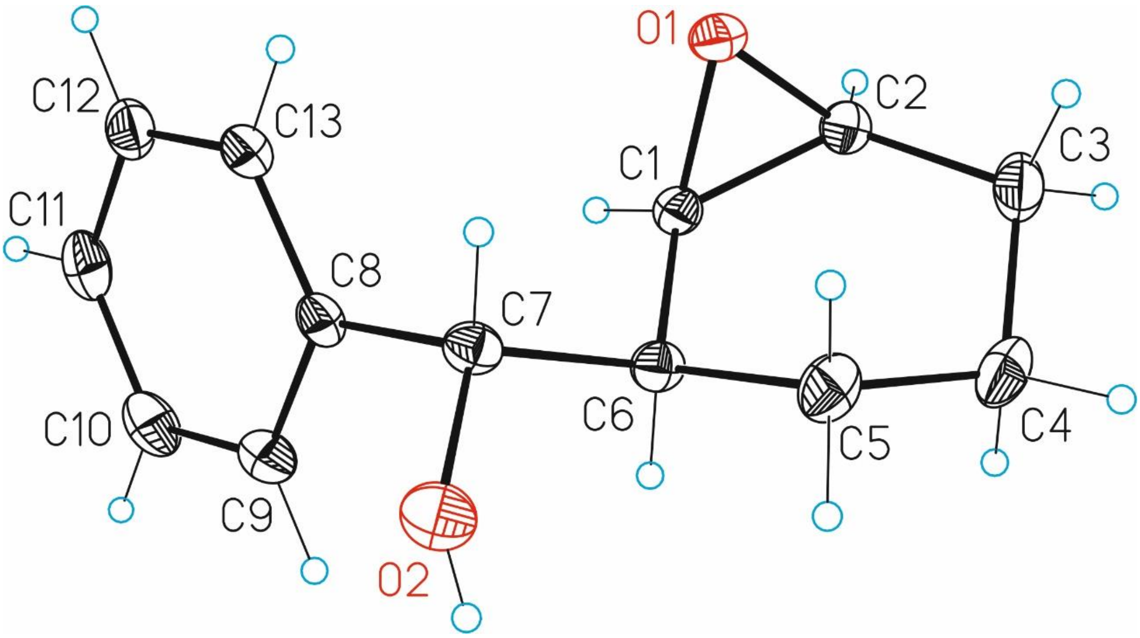

X-ray molecular structure for *syn*-**1a** (CCDC-2157245)

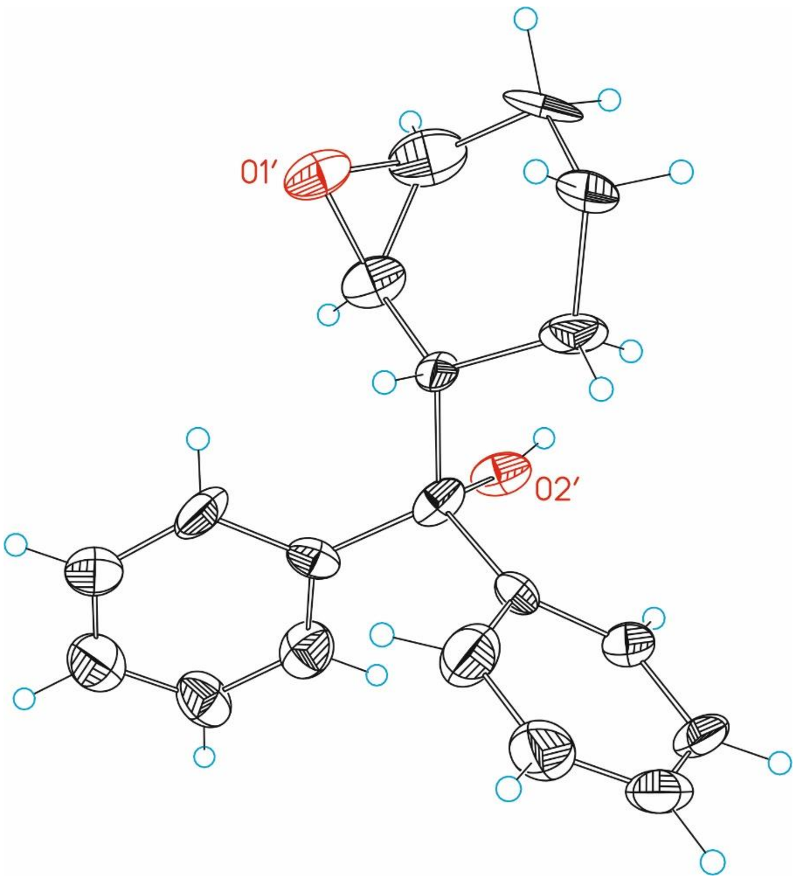

X-ray molecular structure for *anti*-**1o** (CCDC-2157247)

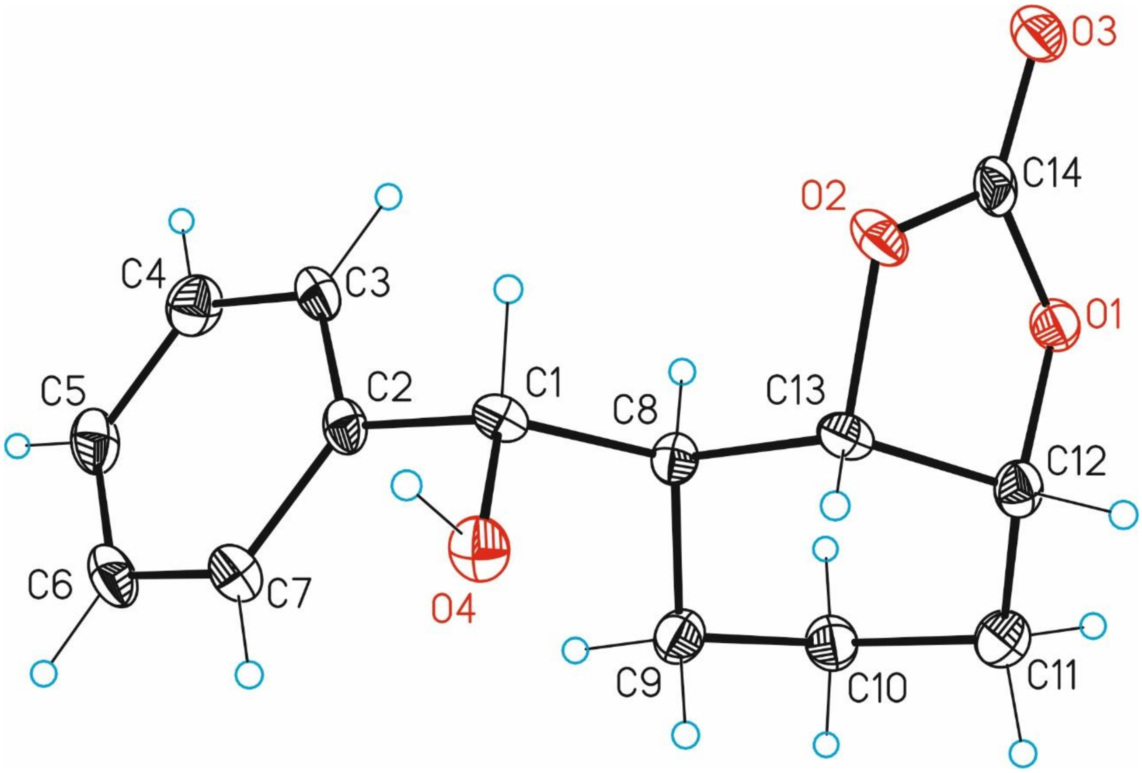

X-ray molecular structure for **P1**[b] (CCDC-2157244)

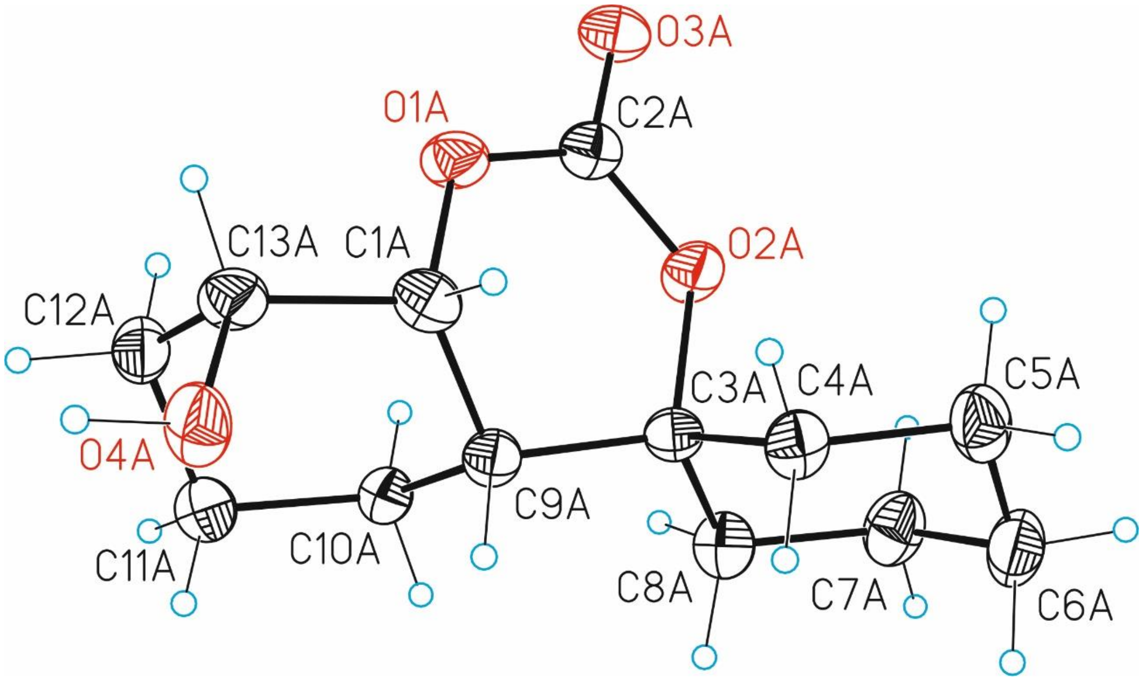

X-ray molecular structure for **P13** (CCDC-2157246)
